# Amphiphilic Iodine(III) Reagents for the Lipophilization of Peptides in Water

**DOI:** 10.1002/anie.202106458

**Published:** 2021-07-12

**Authors:** Abhaya Kumar Mishra, Romain Tessier, Durga Prasad Hari, Jerome Waser

**Affiliations:** ^1^ Laboratory of Catalysis and Organic Synthesis Ecole Polytechnique Fédérale de Lausanne EPFL SB ISIC LCSO, BCH 4306 1015 Lausanne Switzerland; ^2^ Present address: Department of Chemical Biology Max Planck Institute of Molecular Physiology Otto-Hahn-Strasse 11 44227 Dortmund Germany

**Keywords:** amphiphilic reagents, hypervalent iodine, lipidation, lipopeptide, ubiquitin

## Abstract

We report the functionalization of cysteine residues with lipophilic alkynes bearing a silyl group or an alkyl chain using amphiphilic ethynylbenziodoxolone reagents (EBXs). The reactions were carried out in buffer (pH 6 to 9), without organic co‐solvent or removal of oxygen, either at 37 °C or room temperature. The transformation led to a significant increase of peptide lipophilicity and worked for aromatic thiols, homocysteine, cysteine, and peptides containing 4 to 18 amino acids. His_6_‐Cys‐Ubiquitin was also alkynylated under physiological conditions. Under acidic conditions, the thioalkynes were converted into thioesters, which could be cleaved in the presence of hydroxylamine.

## Graphical abstract

Amphiphilic hypervalent iodine reagents enable the alkynylation of cysteine residues in peptides with lipophilic groups in water. The reaction proceeds in neutral standard buffers with high efficiency for a broad range of peptides and one protein. The thioalkynes can be converted into cleavable thioesters under acidic conditions.

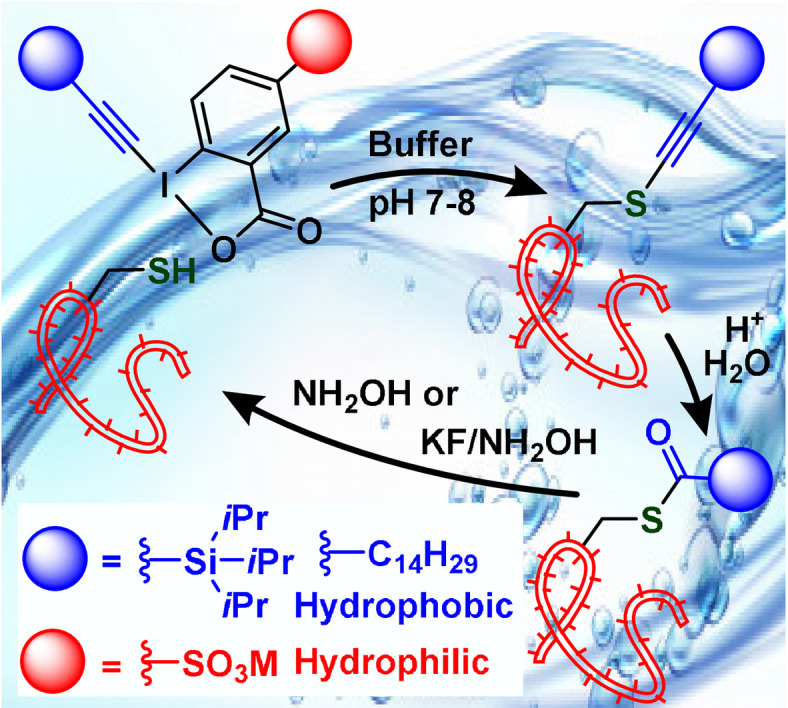

Since the use of insulin in the treatment of diabetes,^[1]^ the importance of peptide‐based drugs has constantly increased.^[2]^ However, the high polarity and low stability of natural peptides result in unfavorable pharmacological properties, requiring chemical modifications.^[3]^ An adequate lipophilicity is essential to control the ADMET properties (absorption, distribution, metabolism, elimination and toxicology),^[4]^ and the lipidation of peptides has proved to be effective in this regard.^[5]^ The lipidation of proteins, through post‐translational modifications (PTMs), is an essential process to control the properties and localization of biomolecules in the cell.^[6]^ Lipopeptides have also found numerous applications in material sciences.^[7]^

Peptides and proteins containing nucleophilic residues are often functionalized with electrophilic reagents.^[8]^ Considering their low abundance and high nucleophilicity, cysteines are targets of choice.^[9]^ To achieve lipidation, naturally occurring palmitoylation and prenylation have been the focus of most research.^[10]^ Due to the low stability of thioesters and the importance of permanent lipidation in multiple applications, chemists have recently focused on more stable natural lipids (Figure 1 a).[^4^, ^5^] Brimble and co‐workers reported the photoinitiated coupling of cysteines with vinyl palmitate **A** via a thiol–ene process.^[11]^ Breinbauer and co‐workers developed a palladium‐catalyzed geranylation of cysteine residues using carbonate reagent **B**.^[12]^ Reports on stable non‐natural modifications of cysteine under mild physiological conditions remain scarce (Figure 1 b).^[9d]^ Müller, Wessig, and co‐workers used maleimide derivatives **C** and **D** for the recruitment of thiol‐containing peptides into the cell membrane.^[13]^ Many reported lipidation processes use highly reactive reagents together with organic co‐solvents, due to the low solubility of the lipophilic reagents, which can be an issue in presence of sensitive biomolecules. There are only few reagents for cysteine functionalization that are fully water‐soluble,^[14]^ and only reagent **E** was reported for the specific case of lipidation, based on the formation of labile disulfide bridges.^[14a]^

**Figure 1:**
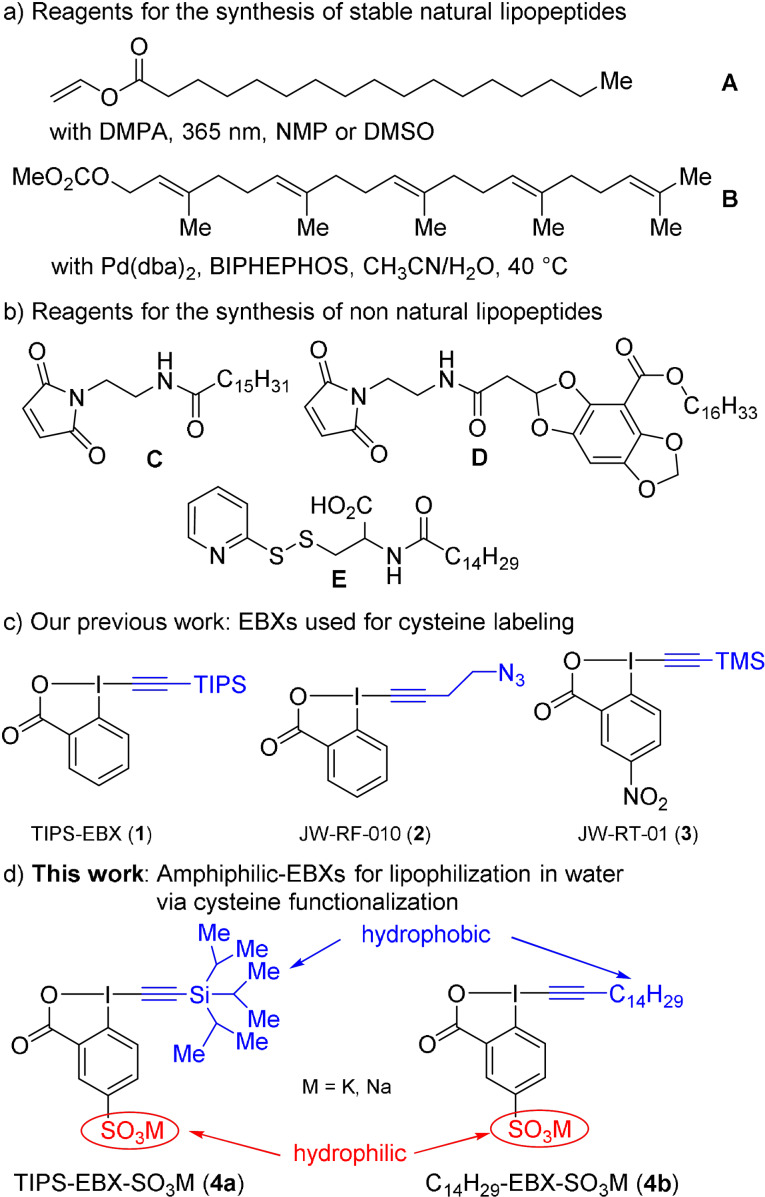
a) Chemical methods for stable natural lipidation. b) Reported reagents for non‐natural lipidation. c) EBX reagents for cysteine labeling. d) This work: amphiphilic reagents for non‐natural lipidation under physiological conditions. DMPA=2,2‐dimethoxy‐2‐phenylacetophenone, NMP=N‐methyl‐2‐pyrrolidone, BIPHEPHOS=6,6′‐[(3,3′‐di‐tert‐butyl‐5,5′‐dimethoxy‐1,1′‐biphenyl‐2,2′‐diyl)bis(oxy)]bis(di‐benzo[d,f][1,3,2]dioxaphosphepin), TIPS=tri*iso*propylsilyl, TMS=trimethylsilyl.

Our group previously investigated ethynylbenziodoxolone (EBX) reagents for the alkynylation of thiols (Figure 1 c).^[15]^ The non‐water‐soluble reagent tri*iso*propylsilylethynyl‐benziodoxolone (TIPS‐EBX, **1**) was used in organic solvents.[^15a^, ^16^] Less lipophilic alkyl reagents, such as JW‐RF‐010 (**2**) gave alkynylated thiols, hypervalent iodine vinylbenziodoxolone (VBX) addition products or a mixture of both depending on the cysteine reactivity and the reaction conditions.[^15b^, ^15c^, ^15d^] Finally, trimethysilylated reagents such as JW‐RT‐01 (**3**) were deprotected under physiological conditions.^[15e]^

The importance of lipo‐peptides and ‐proteins, combined with the lack of lipidation methods in absence of organic solvents and the reactivity of EBXs towards thiols, motivated us to design water‐soluble amphiphilic‐EBXs. Herein, we report the synthesis of sulfonylated EBXs **4 a** and **4 b** and their application for the lipophilization of unprotected peptides and proteins under physiological conditions (Figure 1 d). Under acidic conditions, the obtained thiolakynes could be converted to cleavable thioesters.

Among the approaches to access water‐soluble hypervalent iodine reagents,^[17]^ we focused on sulfonylated derivatives^[18]^ because the iodine precursor 2‐iodo‐5‐potassium sulfonate (**5 a**) is easily accessible (see the Supporting Information)^[19]^ and the sulfonate group ensures good solubility in water at a broad range of pHs. **5 a** was oxidized to **5 b** by NaIO_4_ in 85 % yield. Installation of the alkyne on **5 b** was achieved by using a large excess of Lewis acid. We first prepared TIPS‐EBX‐SO_3_M (**4 a**),^[20]^ as silylated reagents are known to exclusively give alkynylation products (Scheme 1).^[15]^ A slightly modified procedure (BF_3_⋅OEt_2_ instead of TMSOTf as Lewis acid) then allowed us to access the alkyl reagent C_14_H_29_‐EBX‐SO_3_M (**4 b**; Scheme 1). Gratifyingly, **4 a** displayed a more than fifty fold increased solubility in water compared to TIPS‐EBX (**1**) (0.46 for **4 a** vs. <10 mg mL^−1^ for **1**) and **4 b** was also well soluble (0.45 g mL^−1^).

**Scheme 1:**
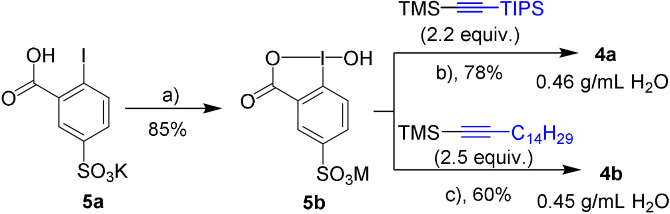
Synthesis of TIPS‐EBX‐SO_3_M (**4 a**) and C_14_H_29_‐EBX‐SO_3_M (**4 b**). a) NaIO_4_ (1.05 equiv), 30 % aq. AcOH (v/v), reflux, 4 h; b) TMSOTf (3.0 equiv), pyridine (6.0 equiv), DCE, 40 °C, 22 h; c) BF_3_⋅Et_2_O (3.0 equiv), pyridine (1.1 equiv), CH_3_CN, rt, 24 h.

We then examined the alkynylation of glutathione (**6**, GSH) in buffer (Table 1). In 10 mM Tris buffer at pH 7.4 at rt,^[15]^ S‐alkynylated product **6 a** was obtained in 47 % yield in 6 h (entry 1).^[21]^ After 16 h, the yield was improved to 83 % (entry 2). At 37 °C, 95 % yield could be obtained in 6 h (entry 3). With gradual increase of the buffer concentration, the yield decreased from 83 % to 24 % (entries 4 to 6). Surprisingly, the yield was almost unchanged from pH 7.0 to 9.0 (entries 7–9). Even at pH 6.0, 54 % of the product was obtained (entry 10). This is unusual for cysteine functionalization, which proceeds normally better under basic conditions. Other buffers led to lower yields (see SI). The reaction proceeded also in pure water (entry 11). The reaction conditions had to be optimized again for reagent **4 b**, due to increased formation of side products (see SI, Table S2). Best results were obtained with a 200 mM Tris buffer at pH 8.0 (Scheme 2). According to our experience with alkyl‐EBXs, we expected to obtain VBX product **6 c**.^[15d]^ However, a mixture of alkynylated (**6 b**), as major product, and VBX (**6 c**), as minor product, was obtained.

**Scheme 2:**
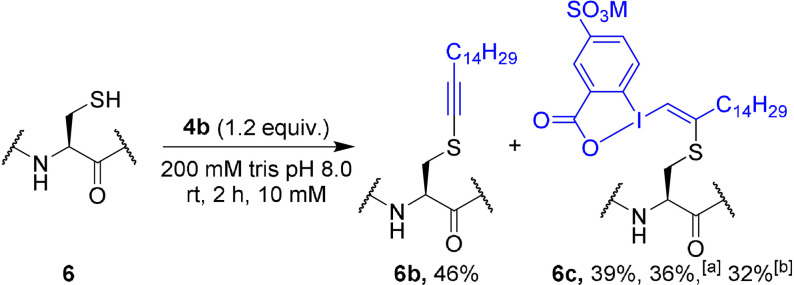
Optimized conditions for the reaction of **4 b** with glutathione (**6**). HPLC‐MS yield is indicated. [a] Isolated yield. [b] Calibrated yield.

**Table 1:** Optimization of the reaction conditions with TIPS‐EBX‐SO_3_K (**4 a**) and glutathione (**6**). 
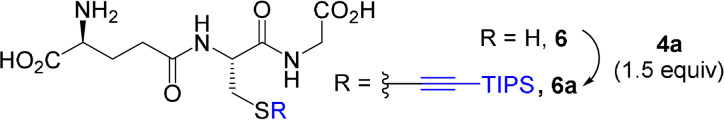

Entry	Reaction conditions^[a]^	**6 a** Yield [%]^[b]^
**1**	10 mM Tris, pH 7.4, rt, 6 h	47
**2**	10 mM Tris, pH 7.4, rt, 16 h	83
**3**	10 mM Tris, pH 7.4, 37 °C, 6 h	95
**4**	40 mM Tris, pH 7.4, 37 °C, 6 h	84
**5**	80 mM Tris, pH 7.4, 37 °C, 6 h	48
**6**	200 mM Tris, pH 7.4, 37 °C, 6 h	24
**7**	10 mM Tris, pH 7.0, 37 °C, 6	93
**8**	10 mM Tris, pH 8.2, 37 °C, 6 h	94
**9**	10 mM Tris, pH 9.0, 37 °C, 6 h	93
**10**	10 mM Tris, pH 6.0, 37 °C, 6 h	54
**11**	Water, 37 °C, 6 h	90

[a] Labeling condition: 16.0 μmol scale in 1.6 mL of non‐degassed buffer. [b] Relative ratio of **6 a** and disulfide based on HPLC‐UV at 214 nm.

With 4‐bromothiophenol (**7**), alkynylation product **7 a** was obtained in 72 % isolated yield with **4 a** (Scheme 3 a). Naphthalene‐2‐thiol (**8**) gave 68 % yield of **8 a**. Both aromatic thiols **7** and **8** did not convert to alkynes **7 b** and **8 b** using **4 b**. Nevertheless, **8 b** could be obtained in 29 % yield using a 100 mM PB buffer. Homocysteine (**9**) gave **9 a** in 95 % HPLC‐MS yield. Reaction with **4 b** gave 66 % of alkyne **9 b** together with 34 % of VBX **9 c**. The reaction of unprotected cysteine (**10**) with **4 a** and **4 b** also proceeded well. After 6 h with **4 b**, only **10 b** was observed. With *N*‐acylated peptide **11**, the alkynylation gave 82 % of **11 a** (79 % calibrated yield and 35 % isolated, Scheme 3 b).^[21]^ Using EBX **4 b**, **11 b** was obtained in 82 % yield and 18 % VBX **11 c** was observed by HPLC. *N*‐terminus unprotected hexapeptides **12** and **13** underwent alkynylation efficiently with both reagents. The reaction was selective for cysteine in presence of other nucleophilic amino acids such as serine, threonine, aspartic acid or lysine (peptides **14**–**18**). Larger peptides **19**–**21** (15 to 18‐mer) were then investigated (Scheme 3 c). Peptide **19** gave **19 a** in 76 % yield. Alkyne **19 b** was obtained in 50 % yield when using a 100 mM PB buffer. Both reagents **4 a** and **4 b** worked well with peptides **20** and **21** bearing nucleophilic side chains such as lysine, tryptophan, tyrosine, serine, threonine or glutamic acid.

**Scheme 3:**
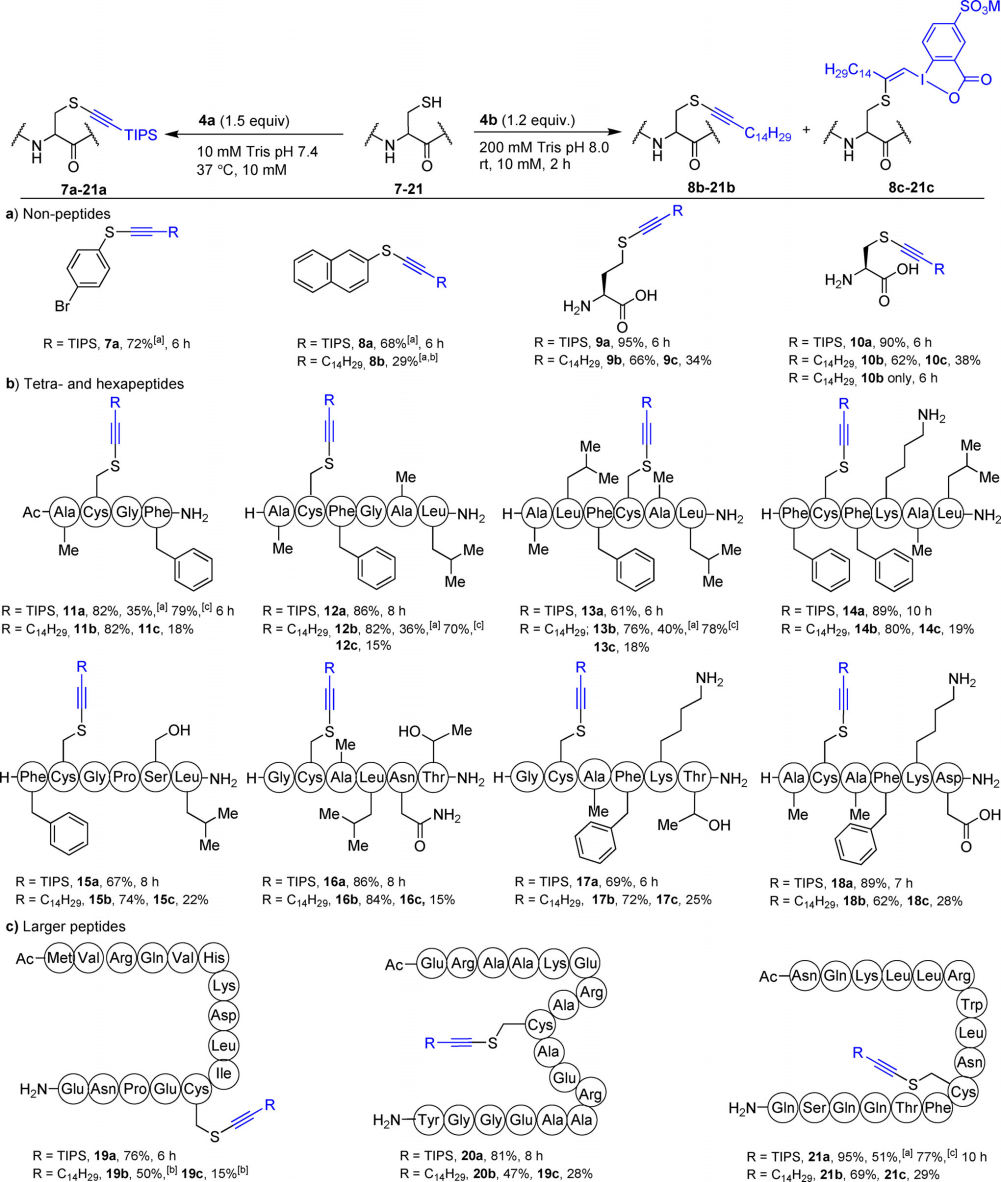
Scope of the alkynylation reaction for a) non‐peptides, b) tetra‐ and hexa‐peptides and c) larger peptides. All the reactions were performed in 0.5 to 64.0 μmol scale at 10 mM concentration. Freshly prepared buffer was used without removing oxygen. Yields: relative ratio based on reverse phase HPLC‐MS chromatogram unless stated otherwise. [a] Isolated yield. [b] Reactions were performed in 100 mM PB buffer at pH 8.0 at rt. [c] Calibrated yield.

We then turned our attention to biologically relevant peptides (Scheme 4): Leu_55_‐His_63_ fragment **22** derived from human serum albumin, Trp_554_‐Ala_566_ fragment **23** derived from the hepatitis C virus (HCV) envelope glycoprotein E2^[22]^ and Phe_32_‐Thr_40_ fragment **24** derived from the human immunodeficiency virus (HIV) tat protein.^[23]^ Peptides **22** and **23** were successfully alkynylated with both reagents. With peptide **24** containing two cysteines, bisalkynylated product **24 a** was obtained in 57 % yield with 6 equivalents of **4 a**. Cysteine‐containing modified His_6_‐Cys‐ubiquitin (**25**)^[15d]^ was also alkynylated efficiently with **4 a**. However, the use of reagent **4 b** led to a complex mixture of products.

**Scheme 4:**
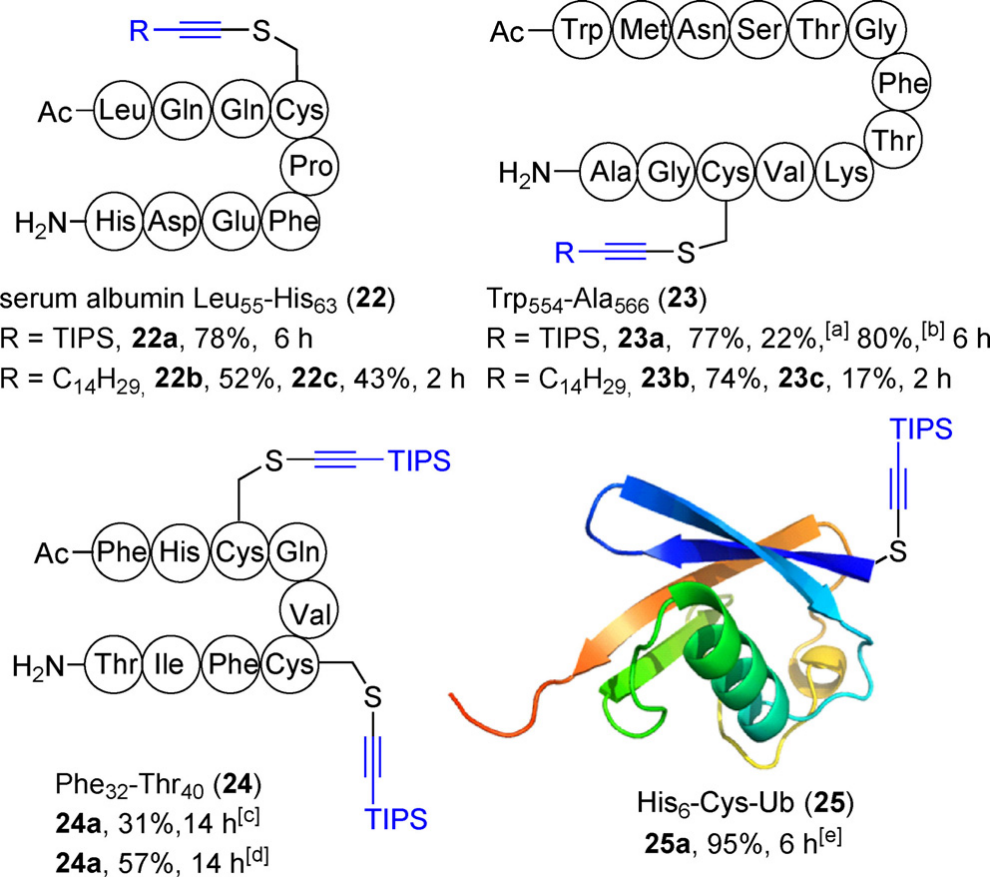
Alkynylation of peptides **22**–**24** and His_6_‐Cys‐ubiquitin (**25**). Reaction conditions: for **22 a** and **23 a**: **4 a** (1.5 equiv), 10 mM Tris pH 7.4, 37 °C, for **22 b** and **23 b**: **4 b** (1.2 equiv), 200 mM Tris buffer pH 8.0, rt. [a] Isolated yield. [b] Calibrated yield. [c] 4 equiv and [d] 6 equiv of **4 a** was used in 10 mM Tris pH 7.4 at 37 °C, 5 mM. [e] Reaction was performed at 300 μM concentration.

A significant increase of retention time in RP‐HPLC was observed for all alkynylated peptides, indicating qualitatively higher lipophilicity. For example, the retention time of peptide **11** shifted from 5.8 to 13.6 min for **11 a** and 20.2 min for **11 b** (See SI). The partition coefficient (LogP) of product **11 a** was determined to be 1.53 compared with −1.43 for **11**.^[24]^

The obtained thioalkynes constitute a new type of lipophilic compounds lacking the electrophilic carbonyl group present in natural palmitoylated peptides, which is required for their hydrolysis. In presence of trifluoroacetic acid (TFA), clean hydration to give thioesters was observed (Scheme 5 a). This hydration can be performed in a one pot protocol with silylated and alkylated alkynes on both small and larger peptides to give thioesters such as **11 aa**–**bb**, **17 aa**–**bb**, **18 aa** and **23 aa**–**bb** in 58–82 % yield. **11 bb**, **17 bb** and **23 bb** are then natural palmitoylated products. The VBX products **11 c** and **23 c** did not react under these acidic conditions. Numerous enzymatic and chemical methods have been reported for the cleavage of palmitoyl groups on cysteine.^[25]^ Indeed, when peptide **17 bb** was submitted to a 1 M solution of hydroxylamine, quantitative cleavage of the thioester was observed (Scheme 5 b). In contrast, the silyl substituted thioester **18 aa** reacts only very slowly under these conditions, probably due to the sterically hindered TIPS group. Nevertheless, treatment with a KF solution followed by hydroxylamine also allowed to cleave this thioester with 90 % conversion. Taken together, our work therefore gives access to lipophilic peptide derivatives modifiable/cleavable under different conditions, which can be exploited depending on the desired application.

**Scheme 5:**
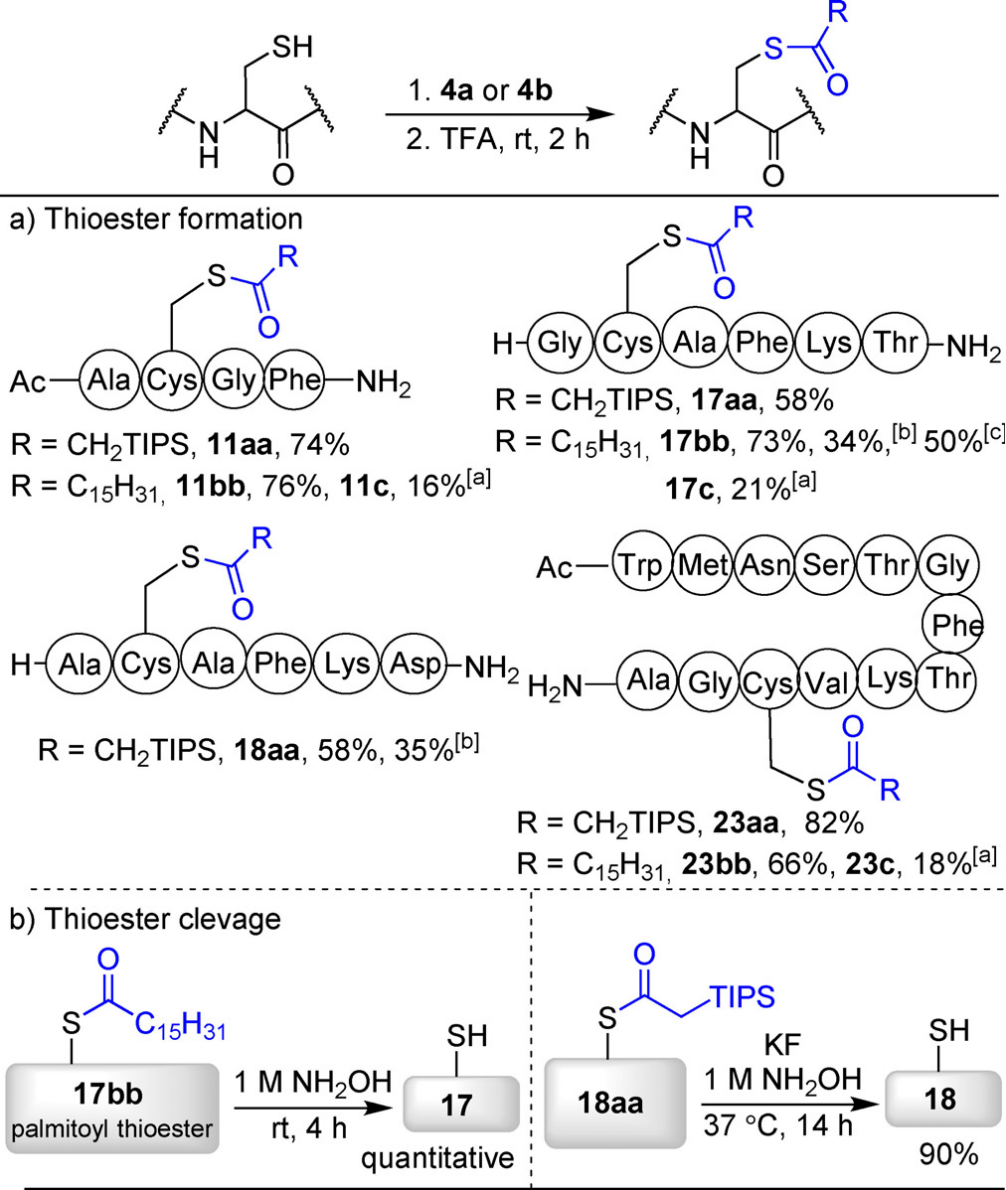
a) Access to thioesters from unprotected peptides in one pot via thioalkynes and b) Cleavage of the thioesters. See Supporting Information for detailed reaction conditions. [a] VBXs remain untouched under these reaction conditions. [b] Isolated yield. [c] Calibrated yield.

In summary, we have synthesized amphiphilic EBX hypervalent iodine reagents, which were employed for the selective lipophilization of cysteine under physiological conditions (pH 7.4–8.0, from room temperature to 37 °C in buffers without organic co‐solvents). Aromatic thiols, homocysteine, cysteine and unprotected tetra‐ and hexapeptides were successfully alkynylated. Larger peptides (15–18‐mers) and one protein (His_6_‐Cys‐Ubiquitin (**25**)) could also be selectively functionalized. Both retention time in reverse phase HPLC and LogP determination showed a significant increase of lipophilicity for the modified peptides, and the obtained thioalkynes could be converted into thioesters under acidic conditions. The thioesters could be easily cleaved using either hydroxylamine or a fluoride/hydroxylamine mixture.

## Conflict of interest

The authors declare no conflict of interest.

## Supporting information

As a service to our authors and readers, this journal provides supporting information supplied by the authors. Such materials are peer reviewed and may be re‐organized for online delivery, but are not copy‐edited or typeset. Technical support issues arising from supporting information (other than missing files) should be addressed to the authors.

Supporting Information
