# Supplementary material for: Amphiphilic Iodine(III) Reagents for the Lipophilization of Peptides in Water
Source: Angew Chem Int Ed Engl. 2021 Jul 12;60(33):17963–8. doi: 10.1002/anie.202106458 (PMC8456932; doi:10.1002/anie.202106458)
Supplement: Supplementary file 1 — Supporting Information [file ANIE-60-17963-s001.pdf]

Angewandte Chemie
Eine Zeitschrift der Gesellschaft Deutscher Chemiker

Supporting Information

Amphiphilic Iodine(III) Reagents for the Lipophilization of Peptides in Water

Abhaya Kumar Mishra[+], *Romain Tessier*[+], *Durga Prasad Hari, and Jerome Waser**

anie_202106458_sm_miscellaneous_information.pdf

SUPPORTING INFORMATION

Table of Contents

1 **General procedure**………………………………………………………………………………… S2

2 **Analytical HPLC and preparative HPLC information**………………………………………… S3

3 **Peptide preparation**……………………………………………………………………………… S3-S12

Peptides purification and analyses

4 **Preparation of amphiphilic reagents 4a and 4b**………………………………………………… S13-S15

4.1. Preparation of **5a**

4.2. Preparation of **5b**

4.3. Preparation of TIPS-EBX-SO_3M (**4a**)

4.4. Preparation of $C_{14}H_{29}$-EBX-SO_3M (**4b**)

4.5. Solubility of **4a** and **4b**

5 **Optimization of reaction conditions**……………………………………………………………… S16-S23

5.1. Experimental procedure and optimization table for **4a**

5.2. Experimental procedure and optimization table for **4b**

5.3. Preparation and isolation of **6c**

5.4. Contrast in reactivity between **1**, **4a** and **4b** with glutathione (**6**) in buffer

5.5. Calibration curve of **6c**

5.6 Stability of **6c** at 37 °C

6 **Yield calculation and Substrate scope for small molecules**…………………………………… S23-S29

6.1. Yield calculation for final products obtained from homocysteine, cysteine, and peptides and general procedure for General reaction procedure for alkynylation with **4a** and **4b**

6.2. Preparation of **7a**

6.3. Preparation of **8a**

6.4. Preparation of **8b**

6.5. Reaction procedure for alkynylation of homocysteine **9** using **4a**

6.6. Reaction procedure for alkynylation of homocysteine **9** using **4b**

7 **Substrate scope for Tetra and hexapeptides** …………………………………………………… S30-S50

7.1. Procedure for preparation of **11a**

7.2. Calibration of **11a**

7.3. Calculation of LogP for **11a**

8 **Substrate scope for long peptides**……………………………………………………………… S51-S57

9 **Substrate scope for bio-active fragments and His_6-Cys-Ubiquitin**………………………… S58-S64

10 **Synthesis of thioester in one-pot**………………………………………………………………… S65-S72

11 **Thioester cleavage** ………………………………………………………………………………… S73-S74

11 **NMR spectra of isolated products**……………………………………………………………… S75-S90

SUPPORTING INFORMATION

1. General procedures

All reactions using anhydrous conditions were performed with oven-dried glassware, under an atmosphere of nitrogen, unless stated otherwise. Anhydrous acetonitrile was purchased from Sigma-Aldrich. All the Fmoc-protected amino acids, Rink Amide MBHA resin and 2-chlorotrityl chloride resin were purchased from GL Biochem. O-Benzotriazole-N,N,N',N'-etramethyluronium-hexafluoro-phosphate (HBTU, GL Biotech), N,N-di*iso*propylethylamine (DIPEA, Iris Biotech GmbH) and hydroxybenzotriazole (HOBt, GL Biotech) were used as received. All the other reagents were purchased from ABCR, Acros, Aldrich, AlfaAesar, Apollo Scientific, Fluorochem, Fluka, Roth and TCI and were used without additional purification. Melting points were measured on a Büchi B-540 melting point apparatus using open glass capillaries. The data is uncorrected. ^{1}H-NMR spectra were recorded on a Brucker DPX-400 400 MHz spectrometer in $CDCl_3$, DMSO-d_6 or D_2O. All signals are reported in ppm with the internal $CHCl_3$ signal at 7.26 ppm, the internal DMSO signal at 2.50 ppm or the internal H_2O signal at 4.79 ppm, MeOD at 4.35 as standard. The data is being reported as: s= singlet, d = doublet, t = triplet, q = quadruplet, qi = quintet, m = multiplet or unresolved, br = broad signal, app = apparent, coupling constant(s) in Hz, integration, interpretation.^{13}C-NMR spectra were recorded with ^{1}H-decoupling on a Brucker DPX-400 100 MHz spectrometer in $CDCl_3$, DMSO-d_6 or D_2O. All signals are reported in ppm with the internal $CHCl_3$ signal at 77.0 ppm or the internal DMSO signal at 39.5 ppm as standard. Infrared spectra were recorded on a JASCO FT-IR B4100 spectrophotometer with an ATR PRO410-S and a ZnSe prisma and are reported as cm-1 (w = weak, m = medium, s = strong, br = broad). High-resolution mass spectrometric measurements were performed by the mass spectrometry service of ISIC at the EPFL on a MICROMASS (ESI) Q-TOF Ultima API.

All reactions related to the peptide/protein alkynylation process were set up on the benchtop and carried out in 1.5 mL vial without oxygen exclusion. Buffers were not degassed and prepared with milliQ water. All the reactions were replicated three times and the reported yield is an average of these replicates.

2. Analytical HPLC and preparative HPLC information

a. Analytical

HPLC-MS measurements were performed on an Agilent 1290 Infinity HPLC system with a G4226A 1290 Autosampler, a G4220A 1290 Bin Pump and a G4212A 1290 DAD detector, connected to a 6130 Quadrupole LC/MS, coupled with a Waters XBridge C18 column (250 x 4.6 mm, 5 µm). Water:acetonitrile 95:5 + 0.1% formic acid (solvent A), water:acetonitrile 5:95 + 0.1% formic acid (solvent B) were used as the mobile phase, at a flow rate of 0.6 mL/min. The column temperature was set up to 25 °C. Low resolution mass spectrometric measurements were acquired using the following parameters: positive electrospray ionization (ESI), temperature of drying gas = 350 °C, flow rate of drying gas = 12 L min^{-1}, pressure of nebulizer gas = 60 psi, capillary voltage = 2500 V and fragmentor voltage = 70 V. To obtain high-resolution mass spectrometric measurements, the desired fraction was recovered after separation on a Waters XBridge C18 column (250 x 4.6 mm, 5 µm) and submitted to the mass spectrometry service of ISIC at the EPFL that uses a MICROMASS (ESI) Q-TOF Ultima API.

Method 1: 100% A to 100% B 0-20 minutes, then 100% B 20 – 30 minutes.

Method 2: 100% A for 5 minutes isocratic, 100% A to 100% B 0-20 minutes, then 100% B 20 – 30 minutes.

Method 3: 100% A to 50% A in 5 minutes, then 50% A for 5-30 minutes.

Method 4: 100% A for 2 minutes, then 100% A to 30% A for 2-32 minutes, then 30% A to 100% B for 32-38 minutes, 100% B to 100% A for 38-40 minutes.

b. Preparative HPLC

Preparative RP-HPLC were performed on an Agilent 1260 HPLC system with a G2260A 1260 Prep ALS Autosampler, a G1361A 1260 Prep Pump, a G1365C 1260 MWD detector and a G1364B 1260 FC-PS collector, coupled with a Waters XBridge semi-preparative C18 column (19 x 150 mm, 5 µm). Water + 0.1% TFA (solvent C), water: acetonitrile 5:95 + 0.1% TFA (solvent D), water (solvent E) or water:acetonitrile 5:95 (solvent F) were used as the mobile phase at a flow rate of 20 $mL.min^{-1}$. Following methods were used.

Method 5: 100% C to 100% D in 0-20, then 100% B 20-30 minutes.

Method 6: 100% C to 50% C for 5 min, then 50% C to 50% D in 5 - 30 minutes.

Method 7: 100% E to 100% F in 30 minutes.

3. Peptide preparation

Solid-Phase Peptide Synthesis (SPPS): Peptides were synthesized on an Advanced ChemTech 348-Ω parallel peptide synthesizer (AAPPTec) using standard Fmoc SPPS-chemistry and Rink Amide MBHA resin (0.26 mmol/g resin, 0.05 mmol scale) for C-terminal amide. The coupling was carried out by shaking the resin with a Fmoc-protected monomer (4.0 equiv.), 1-[Bis(dimethylamino)methylene]-1H-1,2,3-triazolo[4,5-b]pyridinium 3-oxide hexafluorophosphate (HATU, 4.0 equiv.), 4-Methylmorpholine (NMM, 6.0 equiv.), in dimethylformamide (1.3 mL), at 400 rpm, over 30 minutes. This step was accomplished twice. Capping was performed at the end of each coupling using mixture of Ac_2O:2,6-lutidine:DMF (5:6:89),

followed by dimethylformamide wash (4 x 3 mL). Fmoc groups were then removed by shaking the resin with 20% v/v piperidine in dimethylformamide at 400 rpm, over 5 minutes. This step was carried out twice. Next, washing steps were achieved with dimethylformamide (5 x 3 mL). Finally, resin was dried with dichloromethane (5 x 3 mL).

Peptide cleavage and deprotection: Peptides were deprotected and cleaved from the resin under reducing conditions, by treatment with 2.5% v/v water and 2.5% v/v triisopropylsilane (TIPS) in neat trifluoroacetic acid (5 mL). The resulting mixture was shaken for 2 hours at 400 rpm, at room temperature. The resin was removed by filtration and peptides were precipitated in cold diethyl ether (20 mL), followed by a 2 hours incubation at -20 °C. Peptides were pelleted by centrifugation at 4000 rpm, at 4 °C, for 5 minutes. Finally, the mother liquors were carefully removed and crude peptides were dried under vacuum.

Peptide purification and analyses: Peptides were dissolved in water with a minimum amount of organic co-solvent (acetonitrile, dimethylformamide or dimethyl sulfoxide). Peptides were then purified on preparative RPHPLC using method A. Fractions containing the desired peptide were lyophilized. The purity was assessed by analyzing a 10-20 mM peptide solution by RP-HPLC (HPLC gradient: 100% A to 100% B in 30 minutes. At the same time, low-resolution mass spectrometric measurements were also acquired.

Ac-Ala-Cys-Gly-Phe-NH_2 (**11**)

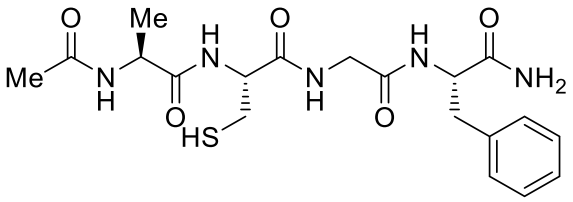

LRMS (ESI) m/z: $[M + H]^+$ Calcd for $C_{19}H_{28}N_5O_5S^+$ 438.18; Found 438.4. HPLC gradient: Method 1.

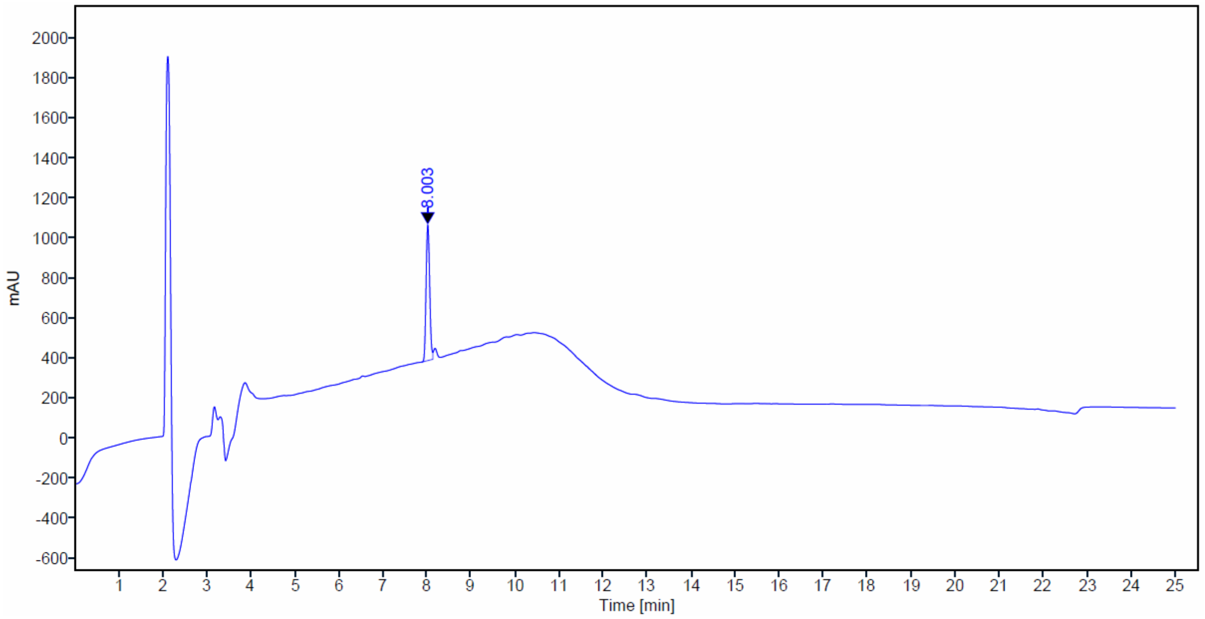

H-Ala-Cys-Phe-Gly-Ala-Leu-NH_2 (**12**)

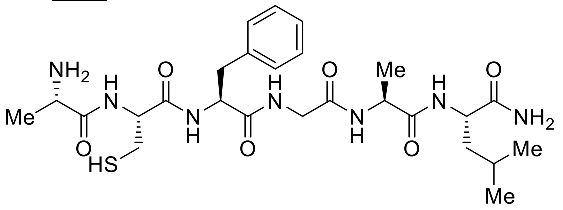

LRMS (ESI) m/z: $[M + H]^+$ Calcd for $C_{26}H_{42}N_7O_6S^+$ 580.29; Found 580.30. HPLC gradient: Method 1.

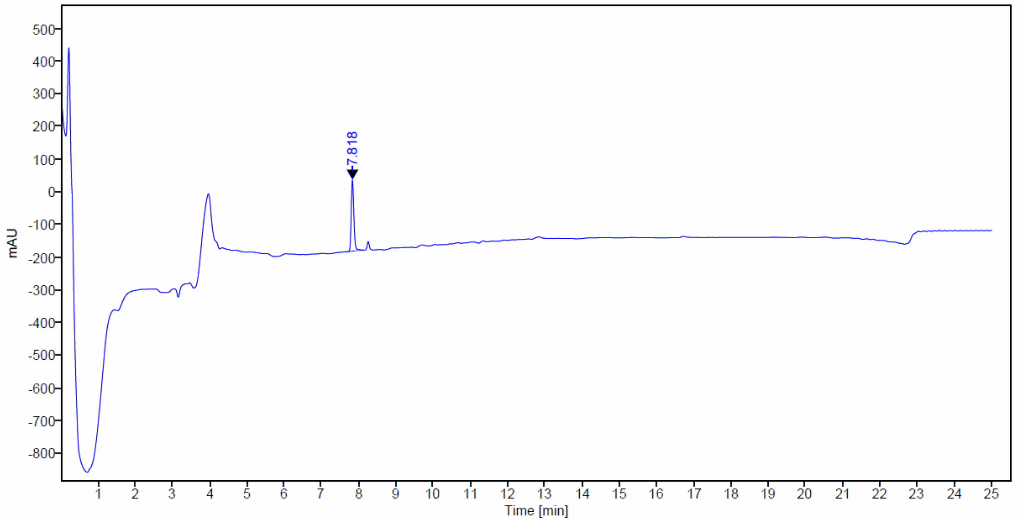

H-Ala-Leu-Phe-Cys-Ala-Leu-NH_2 (**13**)

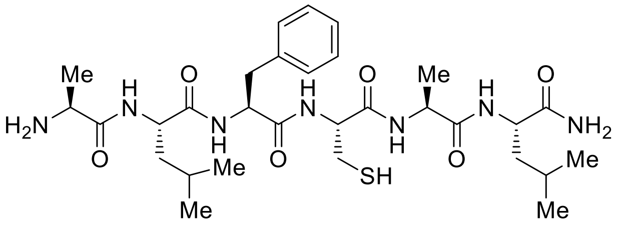

LRMS (ESI) m/z: $[M + H]^+$ Calcd for $C_{30}H_{50}N_7O_6S^+$ 636.35; Found 636.50. HPLC gradient: Method 1.

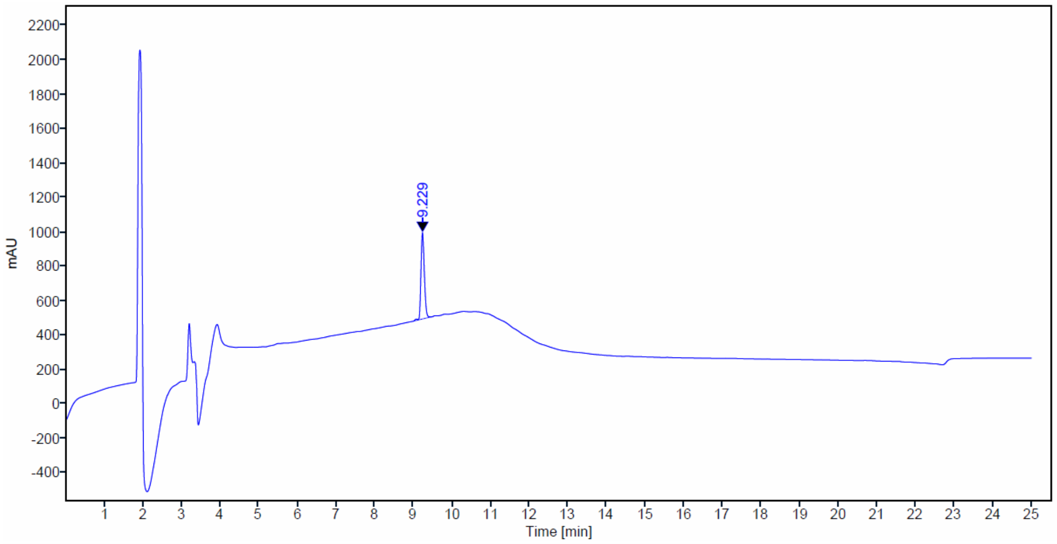

SUPPORTING INFORMATION

H-Phe-Cys-Phe-Lys-Ala-Leu-NH2 (**14**)

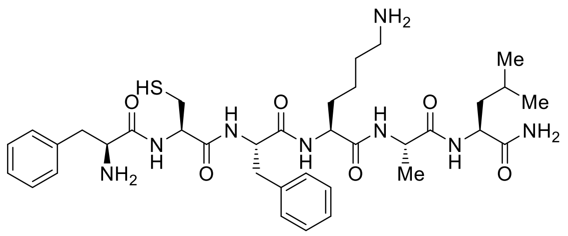

LRMS (ESI) m/z: [M + H]$^+$ Calcd for $C_{36}H_{55}N_8O_6S^+$ 726.39; Found 726.60. HPLC gradient: Method 1.

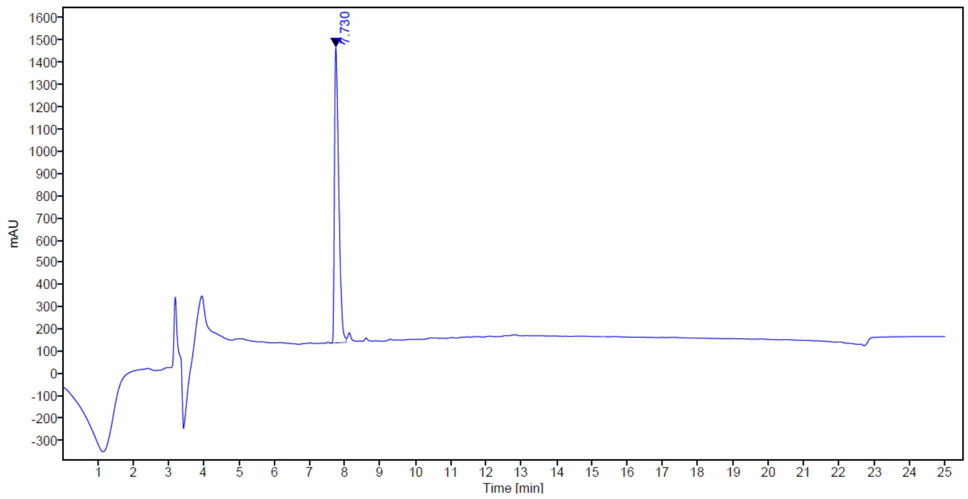

H-Phe-Cys-Gly-Pro-Ser-Leu-NH2 (**15**)

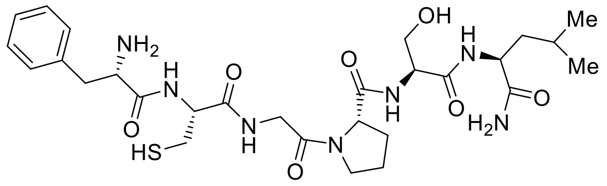

LRMS (ESI) m/z: [M + H]$^+$ Calcd for $C_{28}H_{44}N_7O_7S^+$ 622.30; Found 622.50. HPLC gradient: Method 1.

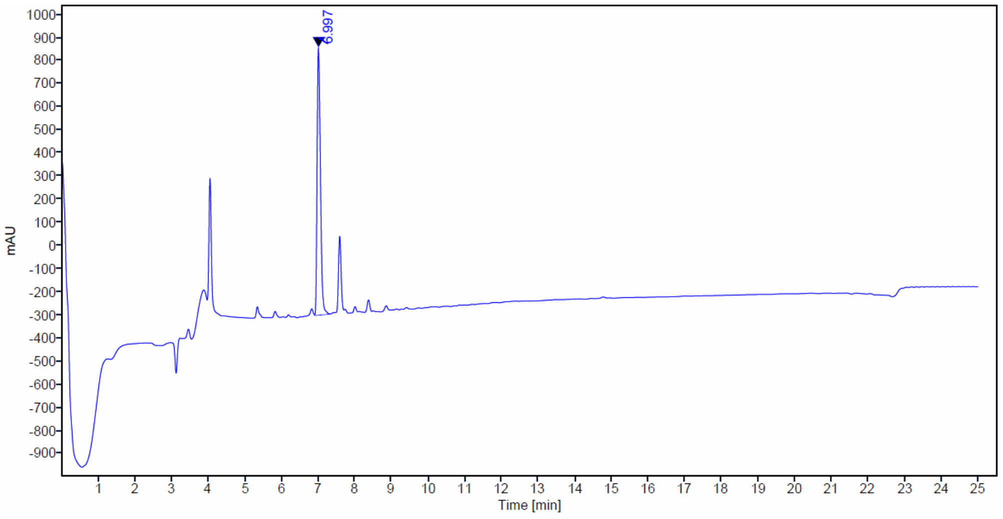

SUPPORTING INFORMATION

H-Gly-Cys-Ala-Leu-Asn-Thr-NH2 (**16**)

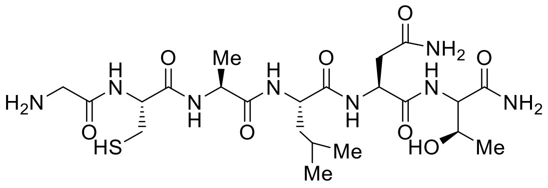

LRMS (ESI) m/z: $[M + H]^+$ Calcd for $C_{22}H_{41}N_8O_8S^+$ 577.27; Found 577.40. HPLC gradient: Method 1.

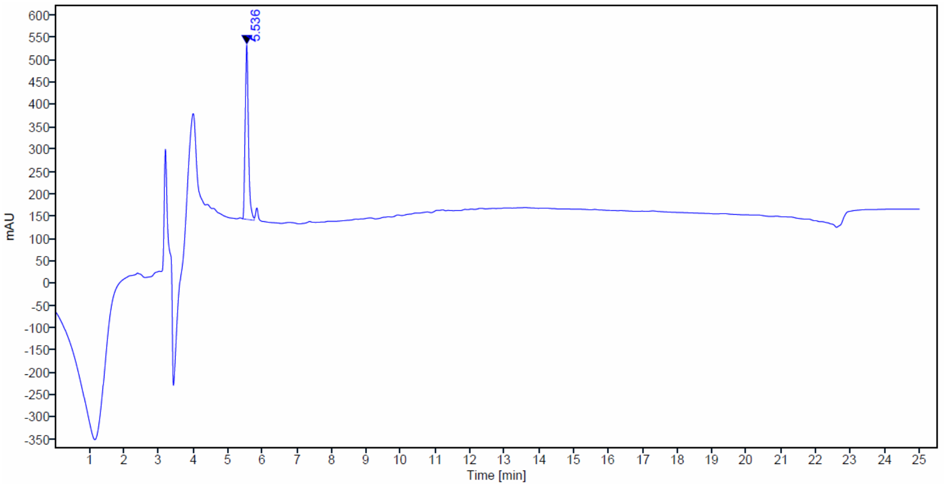

H-Gly-Cys-Ala-Phe-Lys-Thr-NH2 (**17**)

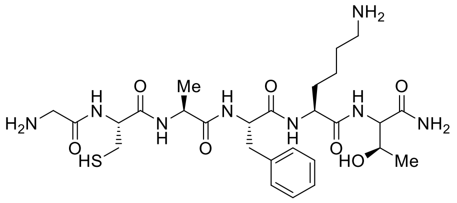

LRMS (ESI) m/z: $[M + H]^+$ Calcd for $C_{27}H_{45}N_8O_7S^+$ 625.31; Found 625.40. HPLC gradient: Method 2.

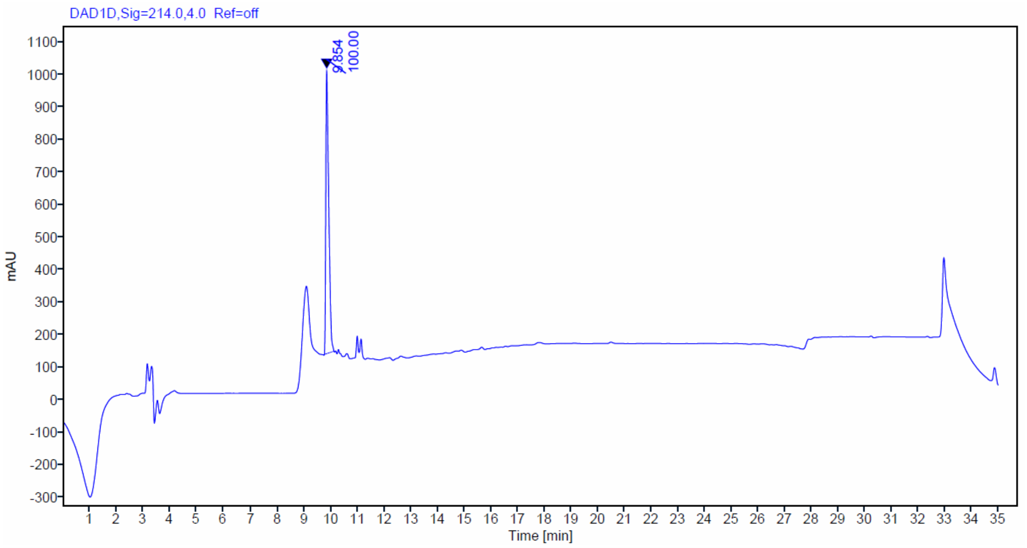

H-Ala-Cys-Ala-Phe-Lys-Asp-NH_2 (**18**)

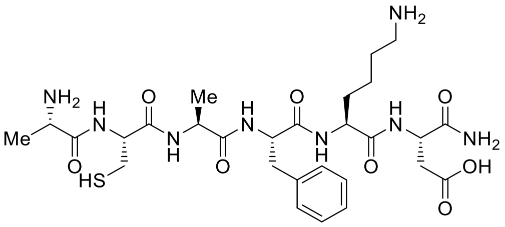

LRMS (ESI) m/z: $[M + H]^+$ Calcd for $C_{28}H_{45}N_8O_8S^+$ 653.30; Found 653.40. HPLC gradient: Method 2.

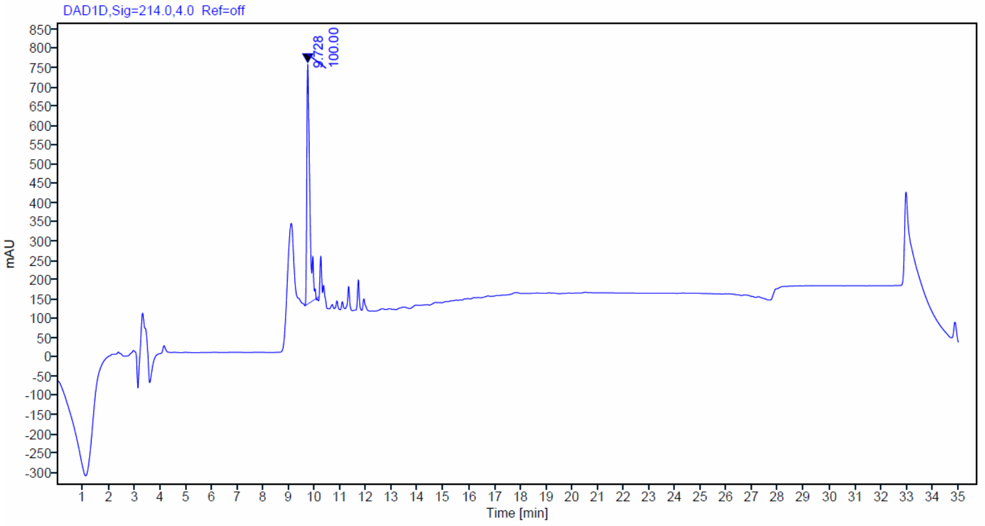

Ac-Met-Val-Arg-Gln-Val-His-Lys-Asp-Leu-Ile-Cys-Glu-Pro-Asn-Glu-NH_2 (**19**)

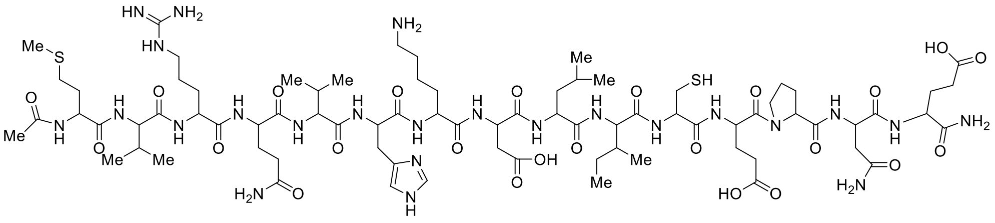

LRMS (ESI) m/z: $[M + 2H]^{2+}$ Calcd for $C_{78}H_{132}N_{24}O_{24}S_2^{2+}$ 927.46; Found 927.70. HPLC gradient: Method 1.

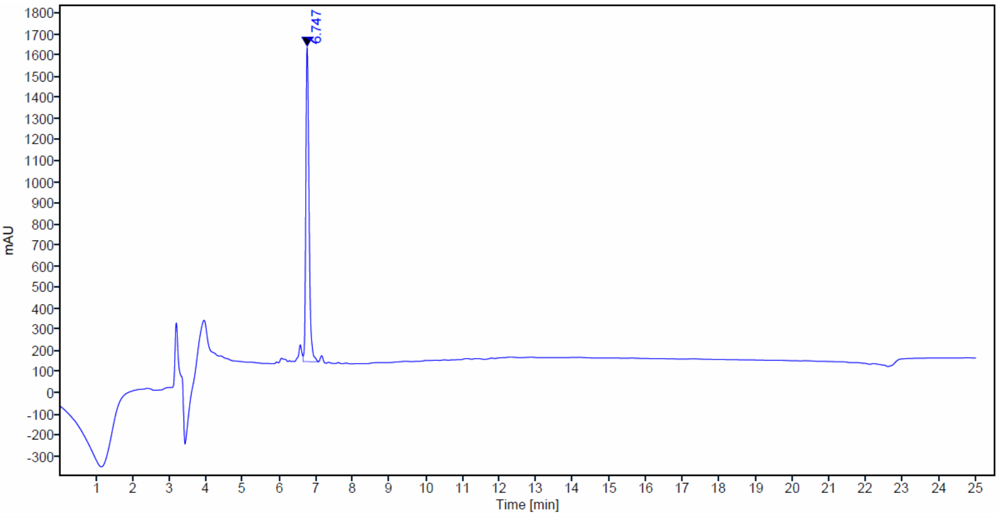

SUPPORTING INFORMATION

Ac-Glu-Arg-Ala-Ala-Lys-Glu-Arg-Ala-Cys-Ala-Glu-Arg-Ala-Ala-Glu-Gly-Gly-Tyr-NH_2 (**20**)

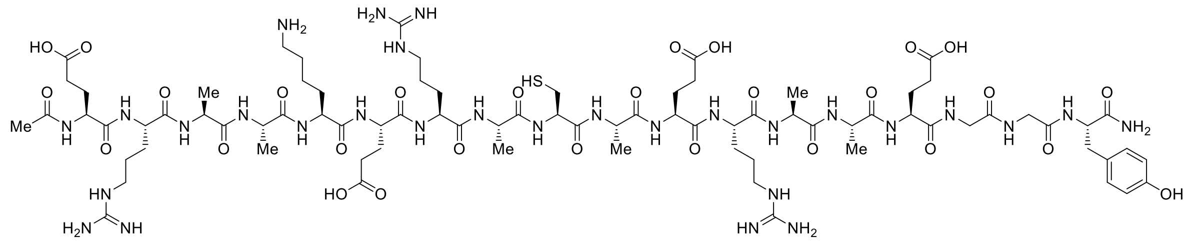

LRMS (ESI) m/z: $[M + 2H]^{2+}$ Calcd for $C_{80}H_{133}N_{29}O_{28}S^{2+}$ 990.97; Found 990.80. HPLC gradient: Method 1.

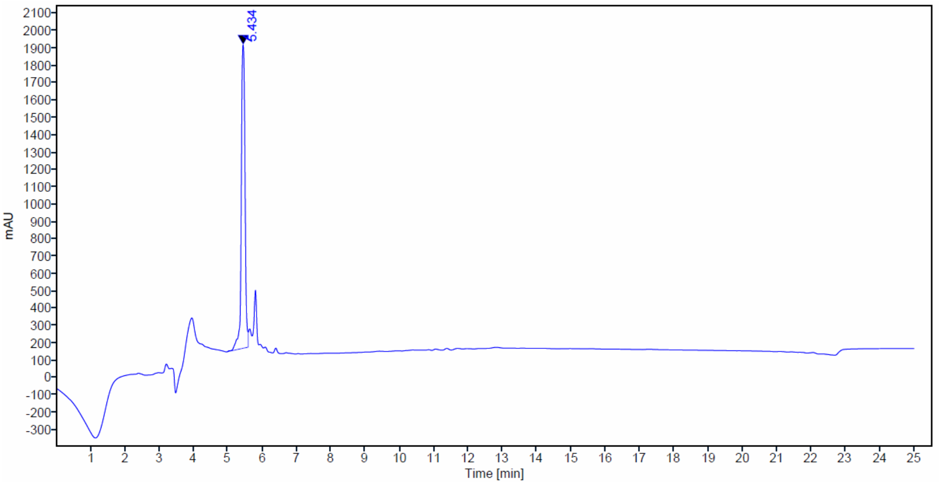

Ac-Asn-Gln-Lys-Leu-Leu-Arg-Trp-Leu-Asn-Cys-Phe-Thr-Gln-Gln-Ser-Gln-NH_2 (**21**)

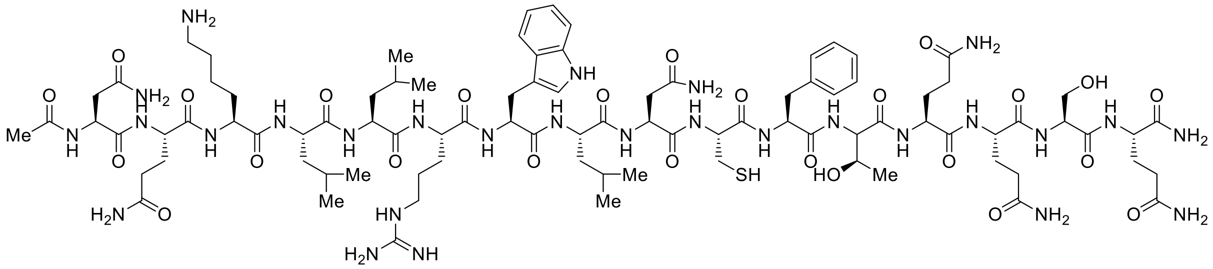

LRMS (ESI) m/z: $[M + 2H]^{2+}$ Calcd for $C_{90}H_{144}N_{28}O_{25}S^{2+}$ 1025.52; Found 1025.20. HPLC gradient: Method 1.

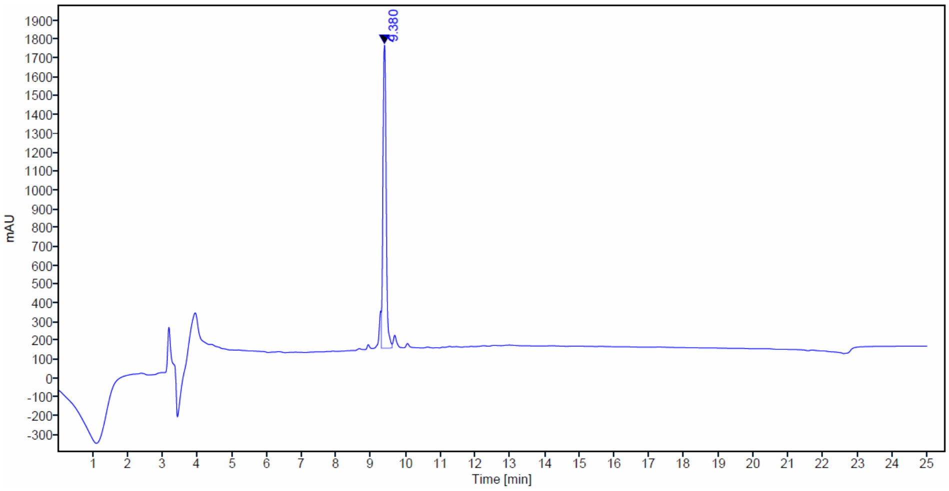

Human Serum Albumin (Leu55-His63): Ac-Leu-Gln-Gln-Cys-Pro-Phe-Glu-Asp-His-NH2 (**22**)

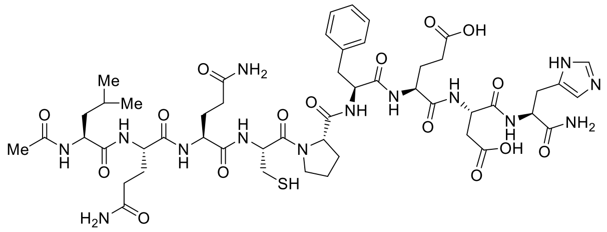

LRMS (ESI) m/z: [M + 2H] $^{2+}$ Calcd for $C_{50}H_{74}N_{14}O_{16}S^{2+}$ 580.25; Found 580.30. HPLC gradient: Method 1.

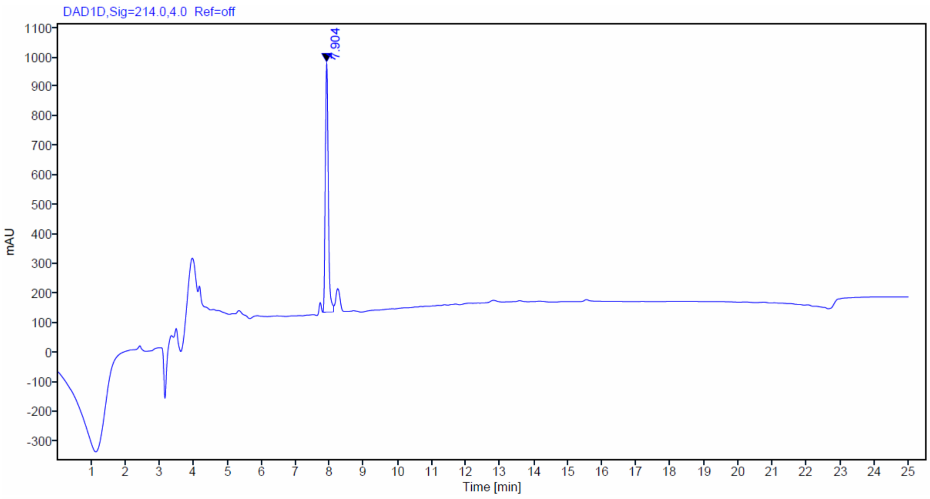

Ac-Trp-Met-Asn-Ser-Thr-Gly-Phe-Thr-Lys-Val-Cys-Gly-Ala-NH2 (**23**)

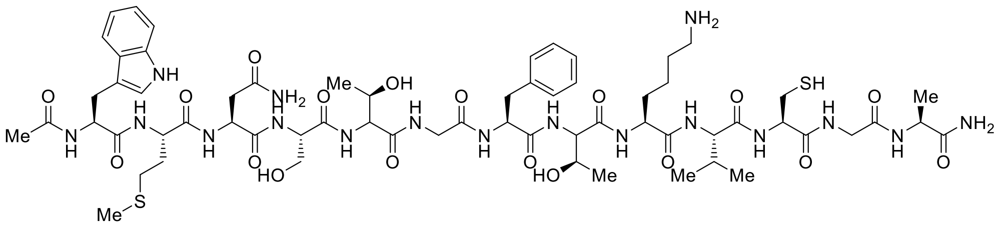

LRMS (ESI) m/z: [M + 2H] $^{2+}$ Calcd for $C_{63}H_{97}N_{17}O_{18}S_2^{2+}$722.83; Found 722.50. HPLC gradient: Method 1.

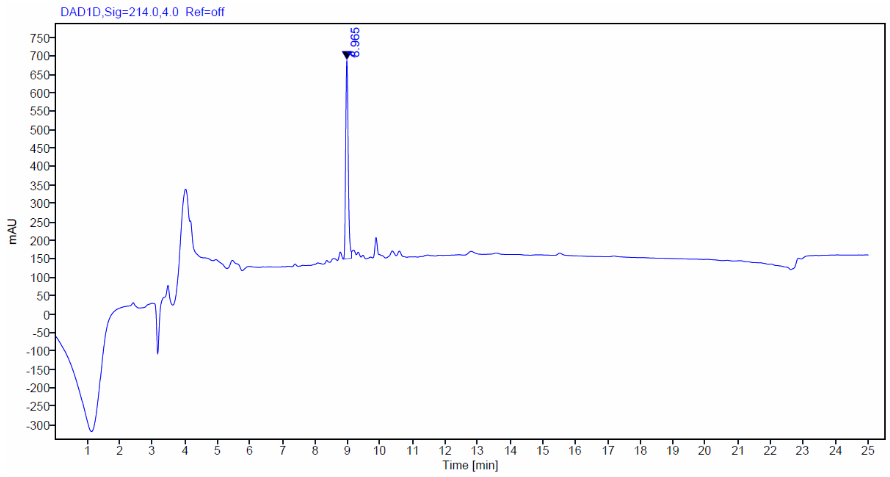

SUPPORTING INFORMATION

TAT-HIV (Phe32-Thr40) Ac-Phe-His-Cys-Gln-Val-Cys-Phe-Ile-Thr-NH2 (**24**)

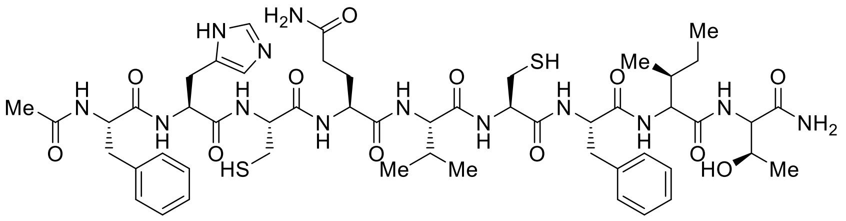

LRMS (ESI) m/z: $[M + H]^+$ Calcd for $C_{52}H_{76}N_{13}O_{12}S_2^+$; 1138.52, Found 1138.4. HPLC gradient: Method 1.

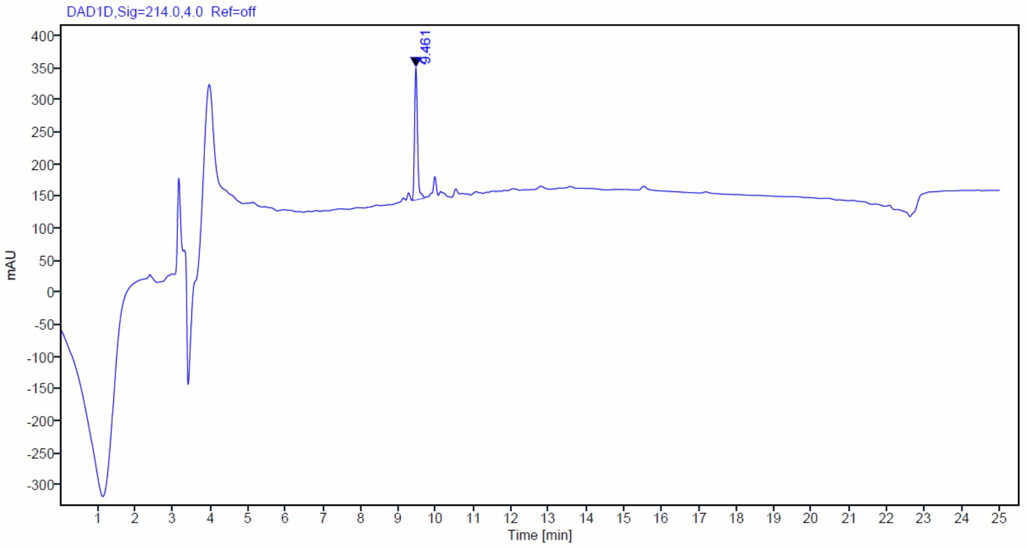

Ubiquitin (**24**)

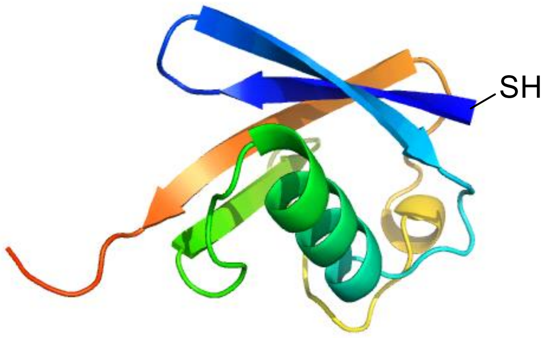

LRMS (ESI) m/z: [M + 15H]$^{15+}$ Calcd for His$_6$-Cys-Ub 714.3 Found 714.5.

HPLC gradient: Method 4.

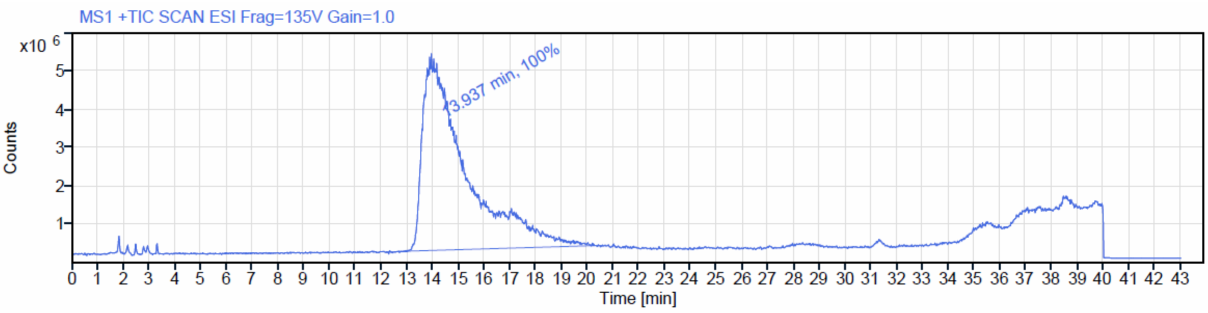

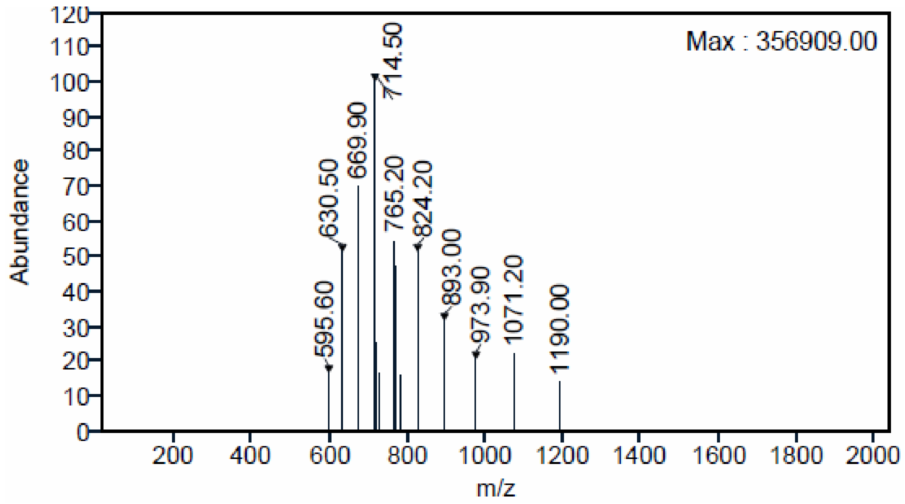

4. Preparation of amphiphilic reagents

Preparation of (**5a**)

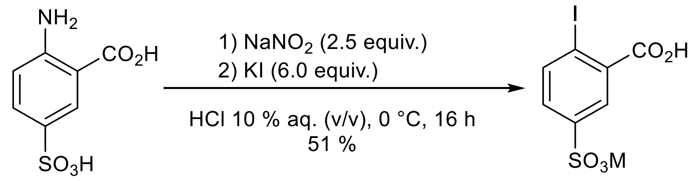

5a M = K, Na

Following a reported procedure,[1] 2-amino-5-sulfobenzoic acid (4.34 g, 20.0 mmol, 1.0 equiv.) was suspended in a 10% aqueous hydrochloric acid solution (100 mL) and cooled to 0 °C. A cooled solution of sodium nitrite ($NaNO_2$, 3.45 g, 50.0 mmol, 2.5 equiv.) in water (18 mL) was slowly added over a period of 45 minutes. After an additional 30 minutes stirring at this temperature, a cooled solution of potassium iodide (KI, 19.9 g, 120 mmol, 6.0 equiv.) in water (75 mL) was slowly added over a period of 1 hour at 0 °C. The resulting dark solution was allowed to warm to room temperature and stirred for 16 hours. Then, the reaction was slowly quenched by small portions of sodium bisulfite (around 14 g) until the solution persistently turned as a light-yellow[2] suspension. The resulting suspension was filtered, washed with acetone (3 x 100 mL) and dichloromethane (50 mL) to afford a yellow pale solid. The collected solid was then recrystallized from water and washed with cold water (2 x 50 mL), acetone (2 x 50 mL) and dichloromethane (2 x 50 mL) to yield pure **5a** (3.71 g, 10.1 mmol, 51% yield) as a pale-yellow solid.

^{1}H NMR (400 MHz, DMSO-d_6) δ 7.95 (d, J = 8.1 Hz, 1H, ArH), 7.90 (d, J = 2.0 Hz, 1H, ArH), 7.41 (dd, J = 8.1, 2.1 Hz, 1H, ArH).

^{13}C NMR (101 MHz, DMSO-d_6) δ 167.9, 147.8, 140.5, 136.4, 129.5, 127.3, 94.8.

Spectra data was consistent with the values reported in literature.[1]

[1] A. Kommreddy, M. S. Bowsher, M. R. Gunna, K. Botha, T. K. Vinod,*Tetrahedron Lett.* **2008**, *49*, 4378.
[2] a) T. Harschneck, S. Hummel, S. Kirsch, P. Klahn, *Chem. Eur. J.* **2012**, *18*, 1187; b) A. Bredenkamp, F. Mohr, S. Kirsch, *Synthesis*, **2015**, *47*, 1937.

Preparation of **5b**

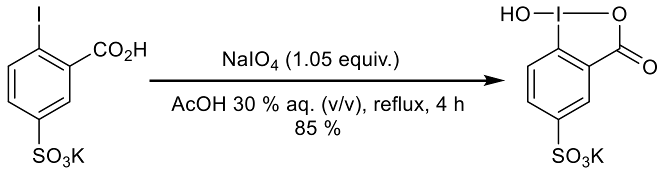

5a **5b**

Following a modified reported procedure, **5a** (1.75 g, 8.17 mmol, 1.00 equiv.) and sodium periodate ($NaIO_4$, 2.85 g, 7.78 mmol, 1.05 equiv.) were suspended in 30% aqueous acetic acid solution (14 mL). The vigorously stirred mixture was heated and refluxed under air for 4 h. The reaction mixture was allowed to cool to room temperature and placed under vacuum. The resulting precipitate was filtered and washed with acetone (3 x 100 mL) and dichloromethane (100 mL). The collected solid was dissolved in methanol, filtered and concentrated under pressure to afford pure potassium 2-iodosyl-5-sulfobenzoate, **5b** (2.52 g, 6.59 mmol, 85% yield) as a white solid.

m.p.: 299 – 300 °C.

^{1}H NMR (400 MHz, DMSO-d_6) δ 8.18 (d, J = 1.8 Hz, 1H, ArH), 8.12 (dd, J = 8.3, 1.9 Hz, 1H, ArH), 7.80 (d, J = 8.3 Hz, 1H, ArH).

^{13}C NMR (101 MHz, DMSO-d_6) δ 167.5 (C=O), 151.1 (ArC), 132.1 (ArC), 130.7 (ArC), 128.5 (ArC), 126.3 (ArC), 119.1 (ArC).

IR ν_{max} 1648 (*m*), 1618 (*m*), 1205 (*s*), 1095 (*m*), 1041 (*m*), 1011 (*m*).

HRMS (ESI/QTOF) m/z: $[M-K]^-$ Calcd for $C_7H_4IO_6S^-$ 342.8779; Found 342.8779.

Preparation of TIPS-EBX-SO_3M (**4a**)

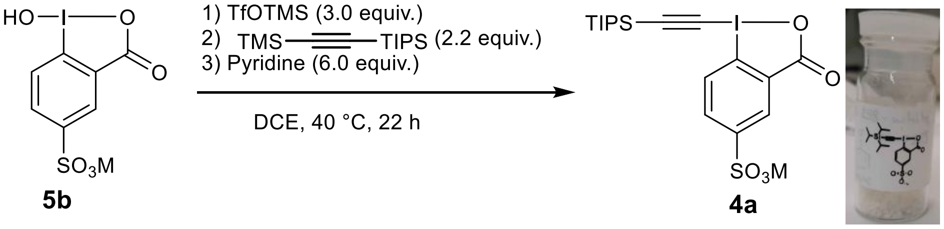

5b **4a**

Trimethylsilyl trifluoromethanesulfonate (TfOTMS, 2.55 mL, 14.1 mmol, 3.0 equiv.) was added dropwise to a stirred suspension of potassium 2-iodosyl-5-sulfobenzoate (1.80 g, 4.71 mmol, 1.0 equiv.) in dichloroethane (157 mL) at 40 °C. After 2 h stirring at this temperature, triisopropyl((trimethylsilyl)ethynyl)silane (2.64 g, 10.4 mmol, 2.2 equiv.) was slowly added to the solution. The reaction mixture was stirred for another 18 h and pyridine (2.29 mL, 28.3 mmol, 6.0 equiv.) was added. After 2 additional hours stirring, the mixture was diluted with dichloromethane (200 mL), washed with a 0.5 N aqueous sodium bicarbonate solution (150 mL) and a 0.5 N aqueous hydrochloric acid solution (150 mL). The organic layer was dried over magnesium sulfate, filtered and the volatiles were removed *in vacuo*. The crude orange oil was purified by column chromatography (SiO_2, Dichloromethane:Methanol gradient from 9:1 to 8:2) to yield pure K/Na-5-sulfonate TIPS-EBX-SO_3M (**4a**, 2.00 g, 3.66 mmol, 78% yield) as a white solid. Yield for **4a** calculated based on K salt.

R_f 0.50 (Dichloromethane:Methanol, 4:1).

m.p.: 325 – 326 °C,

Solubility in water (**4a)**: 0.46 g/mL

^{1}H NMR (400 MHz, DMSO-d_6) δ 8.30 (d, *J* = 2.0 Hz, 1H, Ar*H*), 8.26 (d, *J* = 8.5 Hz, 1H, Ar*H*), 7.98 (dd, *J* = 8.5, 2.1 Hz, 1H, Ar*H*), 1.24 – 1.00 (m, 21H, TIPS).

^{13}C NMR (101 MHz, DMSO-d_6) δ 165.9 (C=O), 151.7 (ArC), 132.2 (ArC), 131.2 (ArC), 128.3 (ArC), 126.6 (ArC), 115.4 (ArC), 110.7 (CC), 67.1 (CC), 18.4 (CH_3), 10.7 (CH).

IR ν_{max} 2952 (*w*), 2866 (*w*), 2372 (*w*), 2347 (*w*), 2325 (*w*), 1634 (*s*), 1238 (*s*), 1169 (*s*), 1102 (*m*), 1036 (*s*), 994 (*m*), 882 (*m*).

HRMS (ESI/QTOF) m/z: $[M-K]^-$ Calcd for $C_{18}H_{24}IO_5SSi^-$ 507.0164; Found 507.0165.

ICP-MS 53.66 µg/mg Na, 5.15 µg/mg K.

Preparation of $C_{14}H_{29}$-EBX-SO_3M (**4b**)

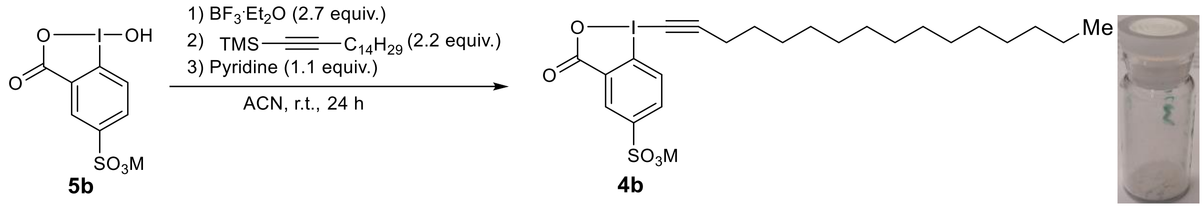

A flame-dried 25 mL round-bottomed flask under nitrogen was charged with potassium **5b** (0.50 g, 1.30 mmol, 1.0 equiv.) and acetonitrile (10.0 mL). A cooled solution of boron trifluoride etherate ($BF_3 \cdot Et_2O$, 0.43 mL, 3.5 mmol, 2.7 equiv.) was added dropwise at room temperature and the reaction was stirred for 2 h. Hexadec-1-yn-1-yltrimethylsilane (0.843 g, 2.86 mmol, 2.2 equiv.) was then slowly added and the resulting mixture was stirred for an additional 18 h. Then, pyridine (115 µL, 1.43 mmol, 1.1 equiv.) was added dropwise and the reaction mixture was stirred for 2 h. The resulting precipitate was filtered and washed with acetonitrile (3 x 15 mL) and pentane (3 x 10 mL). The crude mixture was purified by RP-HPLC using C18 column (gradient: 100 H_2O to 95% ACN/H_20 for 30 mins) to obtain 60% pure **4b** (0.45 g, 0.78 mmol,) as a white solid. HPLC-Gradient: Method 7. Yield for **4b** calculated based on K salt.

R_f 0.30 (dichloromethane:methanol, 9:1).

m.p.: 184 – 185 °C.

^{1}H NMR (400 MHz, DMSO-d_6) δ 8.29 (d, *J* = 2.1 Hz, 1H, ArH), 8.18 (d, *J* = 8.5 Hz, 1H, ArH), 8.02 (dd, *J* = 8.4, 2.1 Hz, 1H, ArH), 2.67 (t, *J* = 7.0 Hz, 2H, CH_2), 1.59 (q, *J* = 7.1 Hz, 2H, CH_2), 1.49 – 1.12 (m, 22H, 11×CH_2), 0.85 (t, *J* = 8.0 Hz , 3H, CH_3).

^{13}C NMR (101 MHz, DMSO-d_6) *δ* 166.3 (C=O), 152.0 (ArC), 132.5 (ArC), 132.0 (ArC), 128.6 (ArC), 127.3 (ArC), 115.7 (CC), 108.6 (CC), 31.7 (CH_2), 29.5 (5×CH_2), 29.4 (CH_2), 29.2 (CH_2), 28.9 (CH_2), 28.7 (CH_2), 28.1 (CH_2), 22.5 (CH_2), 20.1 (CH_2), 14.4 (CH_3).

IR ν_{max} 2918 (*s*), 2850 (*s*), 2176 (*w*), 1636 (*s*), 1469 (*m*), 1234 (*s*), 1190 (*s*), 1099 (*m*), 1039 (*s*), 998 (*s*).

HRMS (ESI/QTOF) m/z: $[M-K]^-$ Calcd for $C_{23}H_{32}IO_5S^-$ 547.1021; Found 547.1019.

ICP-MS 46.01 µg/mg Na, 2.91 µg/mg K.

Solubility in water (**4b)** 0.45 g/mL

5. Optimization of reaction conditions

Experimental procedure for optimization of **4a**

A solution of glutathione (**6**, GSH) in non-degassed 10 mM Tris buffer pH 7.4 was prepared. Then, a solution of **4a** in non-degassed 10 mM Tris buffer pH 7.4 was added to the solution of glutathione **6** in a 1.5 mL vial. The mixture was then stirred at room temperature under "open-flask" conditions. After 6 hours, the labeling furnished a remarkable 47% yield of the alkynylated glutathione **6a** (Table S1, Entry 1). Notably, we did not observe any formation of VBX derivatives. A similar procedure adopted for the other entries of the optimization, Table S1, entry 2-20.

Table 1. Reaction optimization of glutathione with **4a**

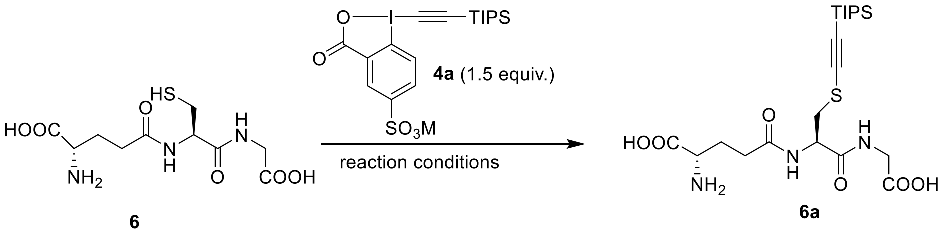

Entry	Buffer	Buffer conc. (mM)	Buffer pH	Temp (°C)	Time (h)	Yield (%)
1	Tris	10	7.4	rt	6	47
2	Tris	10	7.4	rt	16	83
3	Tris	10	7.4	37	6	83
4	Tris	20	7.4	37	6	81
5	Tris	10	7.4	37	6	95
6	Tris	40	7.4	37	6	84
7	Tris	60	7.4	37	6	78
8	Tris	80	7.4	37	6	48
9	Tris	100	7.4	37	6	33
10	Tris	200	7.4	37	6	24
11	Tris	10	7.8	37	6	94
12	Tris	10	8.2	37	6	94
13	Tris	10	8.6	37	6	95
14	Tris	10	9.0	37	6	93
15	Tris	10	7.0	37	6	93
16	PB	10	7.4	rt	6	17
17	PBS	10	7.4	rt	6	13
18	DPBS	10	7.4	rt	6	19
19	HEPES	10	7.4	rt	6	28
20	water	10	-	37	6	90

[a] Labeling condition: 16.0 µmol scale in 1.6 mL of non-degassed buffer. Equivalent of **4a** calculated based on K salt. [b] Yields were determined by relative integration based on HPLC at 214 nm.

Experimental procedure for optimization of **4b**

A 1.5 mL vial was charged with glutathione (**6**, 0.50 mg, 1.6 µmol) and **4b** (1.1 mg, 1.9 µmol) in 163 µL 10 mM Tris pH 7.4 with small magnetic stirring bar, and the reaction mixture was stirred at rt. After 2 h, a 25 µL aliquot of the reaction mixture was diluted with 25 µL acetonitrile to make a clear solution. Then the solution was submitted to HPLC. According to HPLC-MS chromatogram, 25% alkynylated product (**6b**), 7% VBX (**6c**) and 50% disulfide were observed (Table S2, entry 1). There was a large difference in integral ration observed in HPLC-UV and HPLC-MS chromatograms due to difference in absorption of products **6b** and **6c**, therefore the yields reported in the table S2 and manuscript are based on HPLC-MS chromatogram, which is more accurate. A similar experimental procedure was followed for the other entries of Table S2, entries 2-12.

Table S2. Reaction optimization of glutathione with **4b**

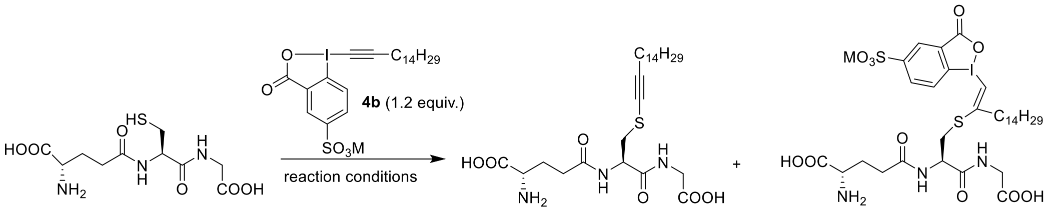

Entry	Reaction conditions	Yield %[a]		
		6b	6c	disulfide
1	10 mM Tris pH 7.4, rt, 2h	25[b]	7[b]	50
2	100 mM Tris, pH 8.0, rt, 2h	45 (8)	41 (92)	14
3	100 mM Tris, pH 8.0, 37, 2h	35 (7)	49 (93)	16
4	200 mM Tris, pH 8.0, rt, 2h	46 (9) [b]	39 (91)	4
5	200 mM Tris pH 8.0, rt, 22h	25 (6) [b]	35 (94)	5
6	200 mM Tris, pH 8.0, 37, 22h	34 (7) [b]	28 (88)	4
7	100 mM PB, pH 8.0, rt, 2h	32 (5)	60 (95)	8
8	100 mM PB, pH 8.0, 37, 2h	21 (2)	46 (98)	33
9	100 mM PB, pH 6.9, rt, 2h	22 (2)	56 (57)	22
10	100 mM PB pH 6.9, 37, 2h	14 (8)	49 (92)	37
11	100 mM HEPES, pH 8.0, rt, 2h	32 (3)	47 (97)	17
12	Water, rt, 2h	30[b]	5[b]	49

[a] Relative ratio of alkynylated (**6b**), VBX (**6c**) and disulfide based on HPLC-MS chromatogram. Equivalent of **4b** calculated based on K salt. Yields in parentheses refers to relative ratio of **6b** and **6c** based on HPLC-UV chromatogram. [b] By-product observed.

SUPPORTING INFORMATION

Comparison of reactivity between **1**, **4a** and **4b** with glutathione (**6**) in buffer

Reaction of TIPS-EBX (1) and glutathione (6). A 1.5 mL vial was charged with glutathione (**6**, 1.0 mg, 3.2 µmol) and **1** (2.3 mg, 5.3 µmol) in 326 µL 10 mM Tris pH 7.4 with small magnetic stirring bar, and the reaction mixture was stirred at 37 °C for 5 h. After 5 h, a 20 µL aliquot of the reaction mixture was diluted with 30 µL acetonitrile to make a clear solution. Then the solution was submitted to HPLC.

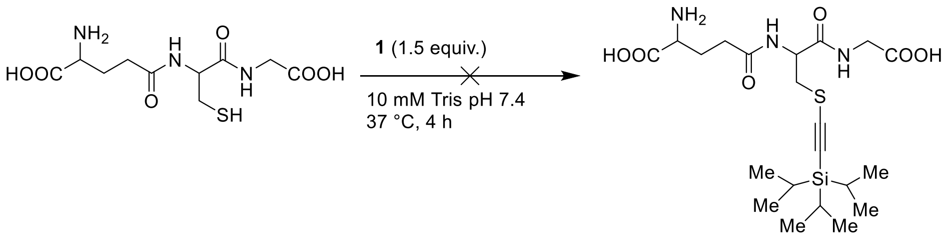

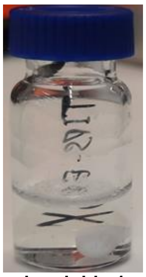

insoluble 1

HPLC-MS chromatogram of the reaction mixture

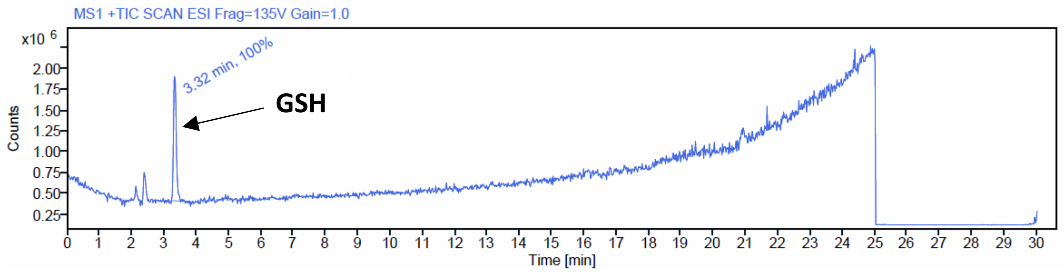

Reaction of 4a and glutathione (6). The same procedure was followed as for the reaction of TIPS-EBX (**1**) and glutathione (**6**).

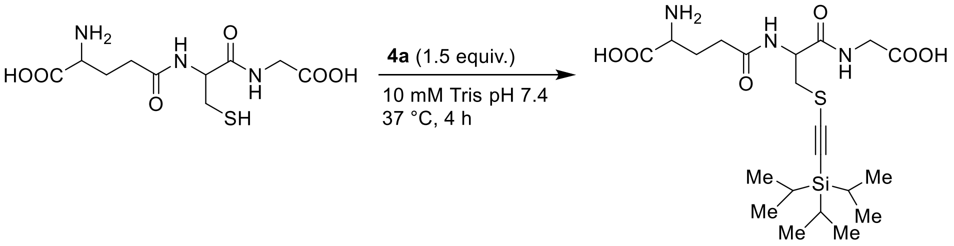

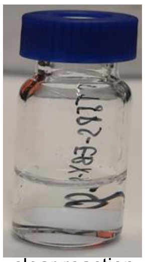

clear reaction mixture

HPLC-MS chromatogram of the reaction mixture

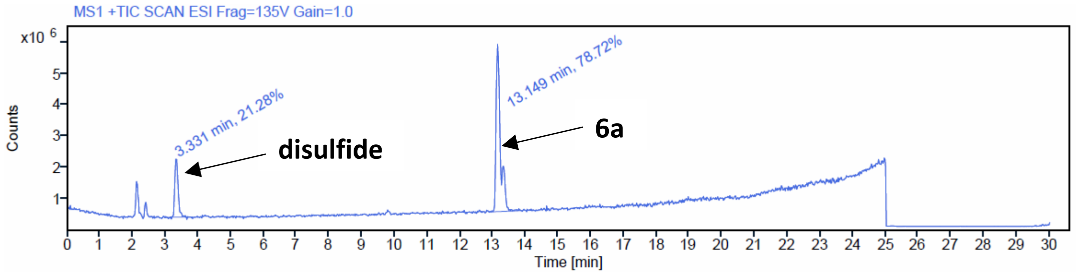

Reaction of 4b and glutathione (6). The same procedure was followed as for the reaction of TIPS-EBX (**1**) and glutathione (**6**).

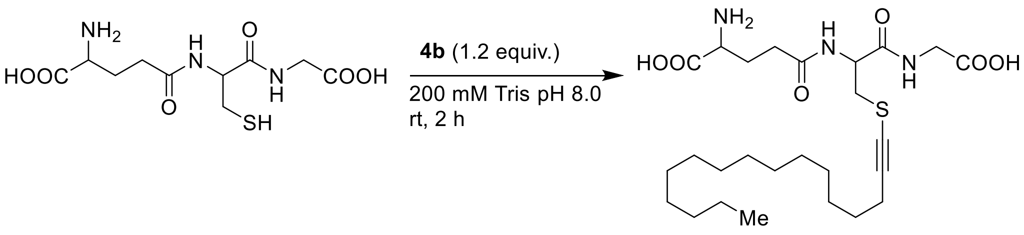

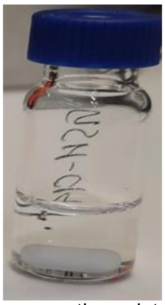

clear reaction mixture

HPLC-MS chromatogram of the reaction mixture

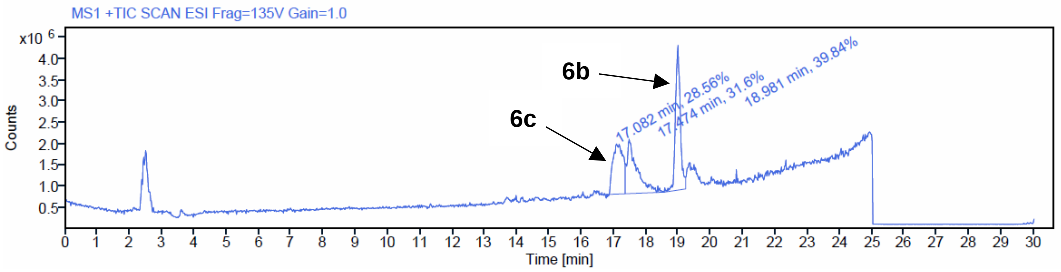

Reaction of **1** and **6** in biphasic solvents (buffer :DCM)

The same procedure was followed as for the reaction of TIPS-EBX (**1**) and glutathione (**6**), but using a 1:1 buffer/DCM mixture instead of pure buffer.

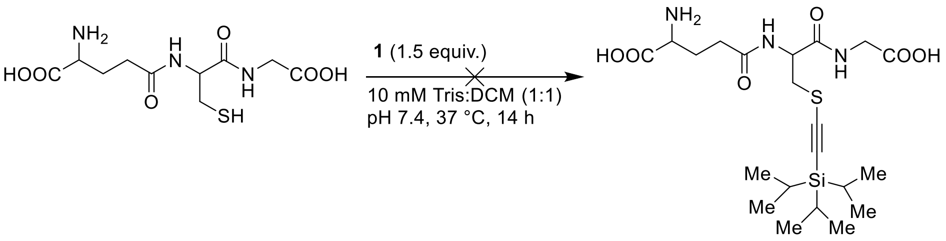

HPLC-MS chromatogram of reaction mixture of water and DCM layer

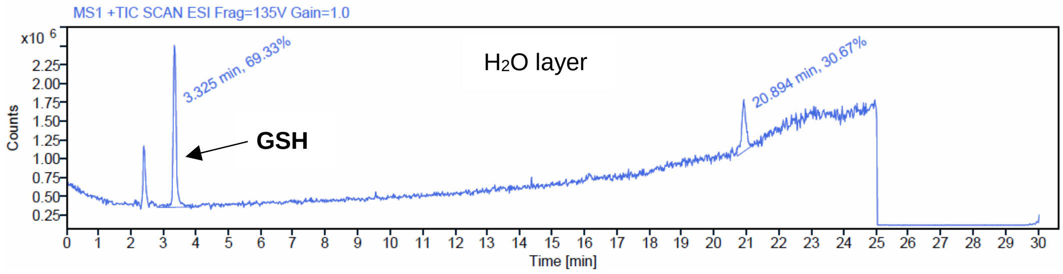

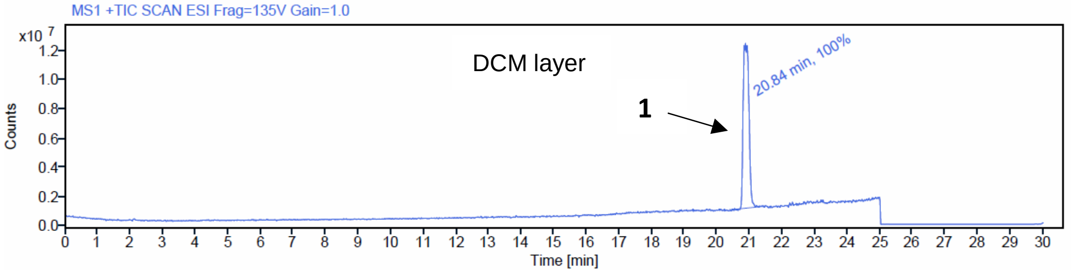

Preparation and isolation of **6c**

A 5 mL vial charged with glutathione (20.0 mg, 65.1 µmol) and **4b** (45.9 mg, 78.0 µmol) dissolved in 3 mL of 100 mM PB pH 8.0. The phosphate buffer was used without degassing. The reaction mixture was stirred at room temperature for two hours. After two hours a 20 µL aliquot of the reaction mixture was diluted 10 times with 50% CH_3CN: H_2O mixture to make a clear solution. The progress of the reaction was monitored by reverse phase HPLC-MS. HPLC-MS revealed a 32:60 ratio of **6b** and **6c** respectively. HPLC gradient Method 5 was used to obtained 36% (23.1 mg, 23.4 mmol) pure white solid of **6c** by preparative reverse phase HPLC. Calibrated yield 32% (retention time = 15.9).

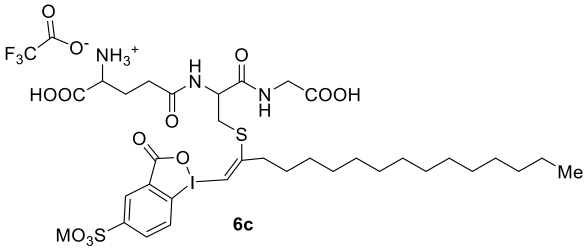

6c

M = Na, K

Analytical HPLC-UV and HPLC-MS chromatogram of reaction mixture

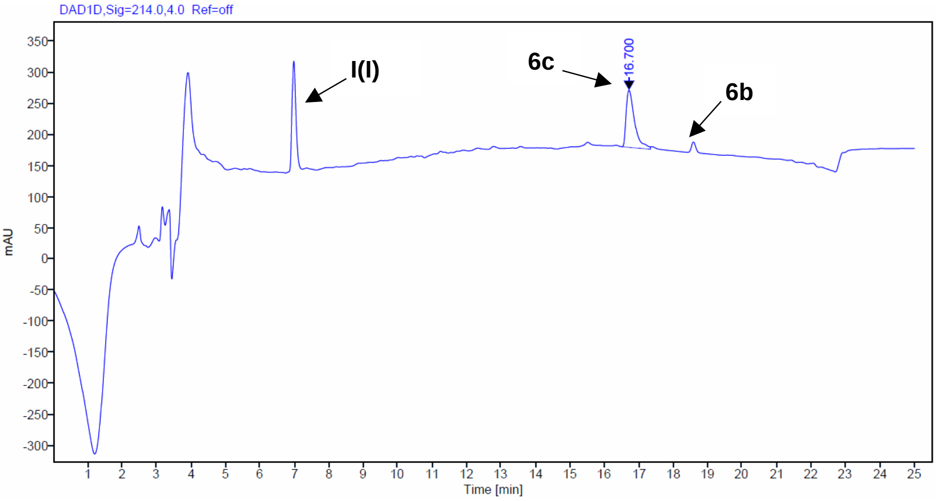

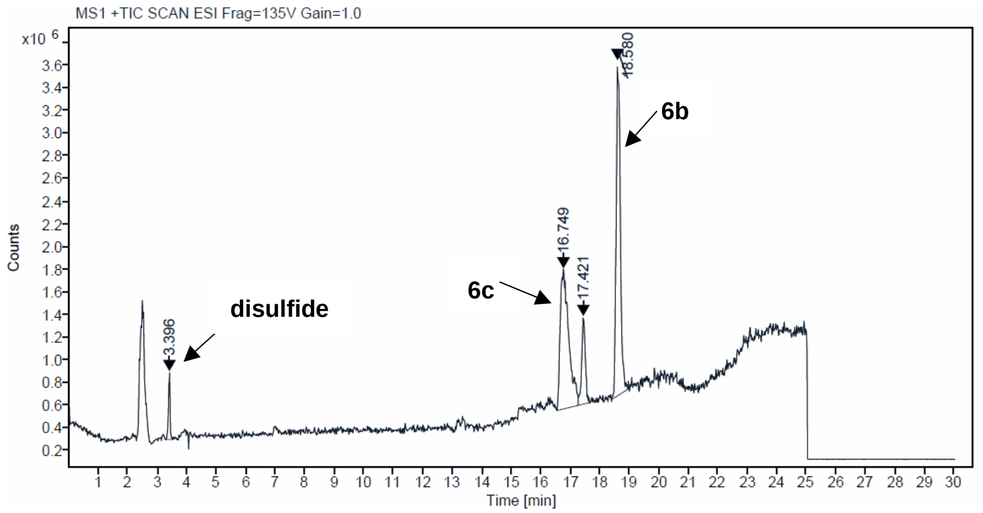

^{1}H NMR (400 MHz, MeOD) δ 8.74 (d, *J* = 2.1 Hz, 1H, ArH), 8.13 (dd, *J* = 8.5, 2.2 Hz, 1H, ArH), 7.71 (d, *J* = 8.5 Hz, 1H, ArH), 7.04 (s, 1H, vinylic-CH), 4.49 (dd, *J* = 10.0, 3.8 Hz, 1H, CHN), 4.03 (t, *J* = 6.4 Hz, 1H, NH), 3.93 (d, *J* = 3.4 Hz, 2H, CH_2N), 3.63 – 3.49 (m, 1H, CHN), 3.12 – 2.82 (m, 3H, CH_2 and NH), 2.51 – 2.19 (m, 2H, CH_2), 2.08 – 2.01 (m, 2H, CH_2), 1.83 – 1.72 (m, 2H, CH_2), 1.35 (d, *J* = 12.1 Hz, 24H, 12×CH_2), 0.93 (t, *J* = 6.4 Hz, 3H, CH_3).

^{19}F NMR (376 MHz, MeOD) δ -77.2.

^{13}C NMR (101 MHz, MeOD) δ 172.7 (C=O), 171.2 (C=O), 170.8 (C=O), 170.0 (C=O), 168.1 (C=O), 164.2 (ArC), 148.2 (ArC), 132.4 (ArC), 131.5 (ArC), 129.6 (ArC), 128.0 (ArC), 127.9 (ArC), 113.9 (C=C), 98.2 (vinylic-CH), 53.0 (CH), 51.9 (CH), 40.3 (CH_2), 36.3 (CH_2), 33.2 (CH_2), 31.6 (CH_2), 30.4 (CH_2), 29.4 (3×CH_2), 29.3 (3×CH_2), 29.1 (CH_2) 29.0 (CH_2), 28.7 (CH_2), 28.6 (CH_2), 25.3 (CH_2), 22.3 (CH_2), 13.0 (CH_3).

HRMS (ESI/QTOF) m/z: $[M+H]^+$ Calcd for $C_{33}H_{51}IN_3O_{11}S_2^+$ 856.2009; Found 856.2023.

Calibration **6c**

Calibration of **6a** was achieved through the preparation of several samples of different concentrations and their analysis on RP HPLC. In order to obtain average curve each analysis was performed 3 times. The following linear regression was obtained: y = 1204x + 9.8942, and R = 0.9967, where axis X is the concentration in millimolar (mM) of **6c** and Y the absorbance area of the peak at 214 nm.

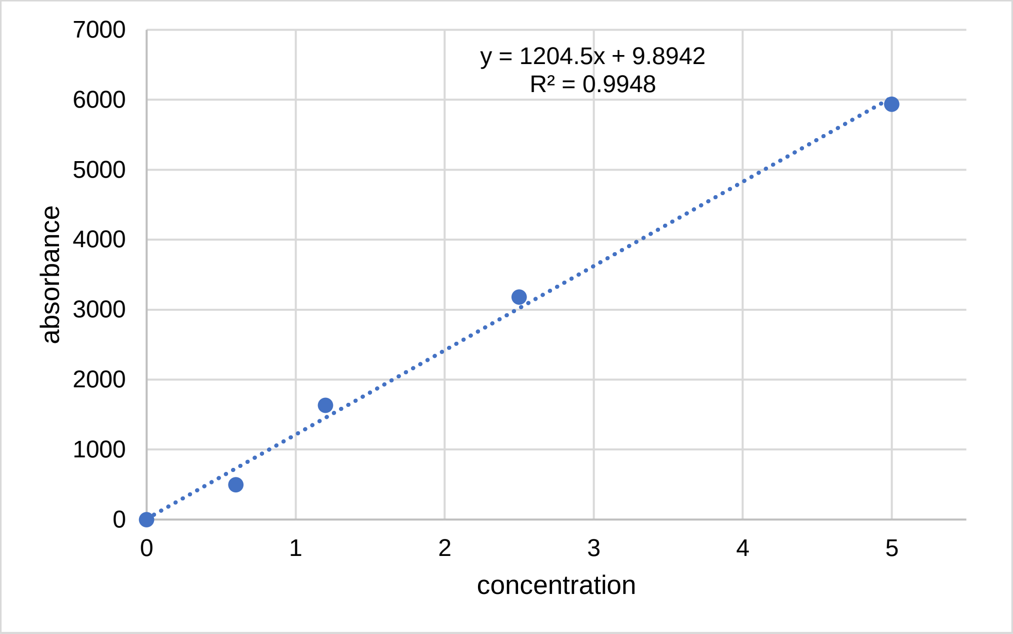

Figure S1: Calibration curve of **6c**.

Stability of 6c at 37 °C. Isolated **6c** was dissolved in 0.1M Tris buffer pH 8.0 and stirred for 20 h at 37 °C. After 20 h HPLC-MS reveled the 14% **6b**. It means that **6c** converted in to **6b**, but rate of was very slow.

HPLC gradient: Method 1.

<u>HPLC-MS chromatogram of isolated **6c**</u>

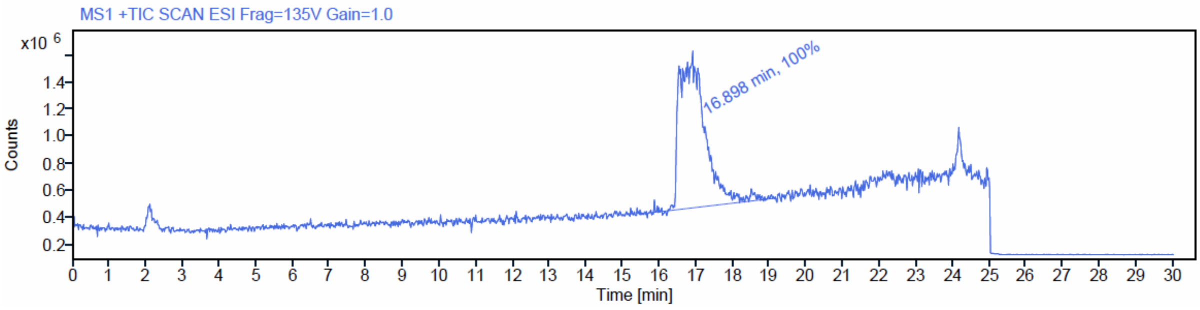

<u>HPLC-MS chromatogram of **6c** after stirring at 37 °C for 20 h</u>

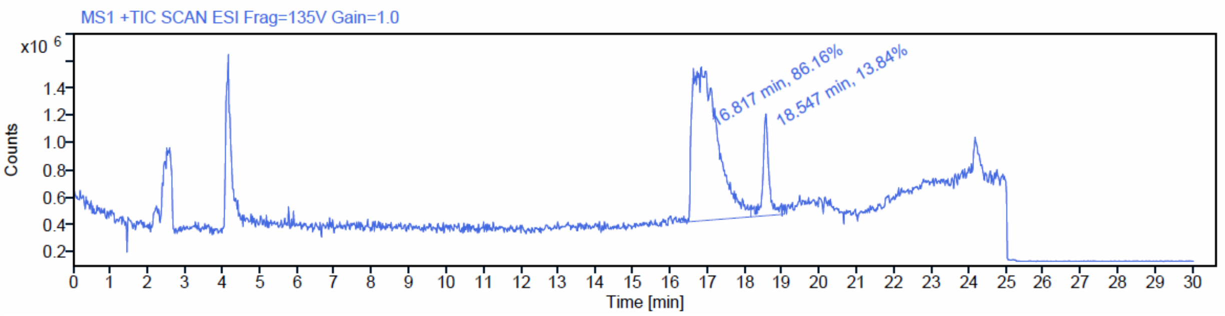

Yield calculation for final products obtained from homocysteine, cysteine, and peptides

The peak areas for all-relevant peptide-containing species on the chromatogram were integrated and the yield was determined using a slightly modified equation introduced by Li *et al.*:[3] yield % = I product/(Istarting + Iproduct + I*oxidation* + Iside product), where Istarting, Iproduct, I*oxidation* and Iside product respectively represent the average ion counts of the remaining starting material, product, oxidized starting material and side product, if any.

General reaction procedure for alkynylation with **4a**

A 1.5 mL vial was charged with thiols (1.0 equiv., concentration maintained 10 mM) and **4a** (1.5 equiv.) in 10 mM Tris pH 7.4 with small magnetic stirring bar. The reaction mixture was stirred at 37 °C for 6 h. The buffer was used directly from freshly prepared solution without degassing. After 6 h, an aliquot of 25 µL of the reaction mixture was diluted with 25 µL acetonitrile to give a clear solution. The solution was submitted to HPLC. The yields were calculated based on HPLC-MS chromatogram, unless otherwise stated.

[3] N. Li, R. Lim, S. Edwardraja, Q. Lin, *J. Am. Chem. Soc.* **2011**, *133*, 15316.

General reaction procedure for alkynylation with **4b**

A 1.5 mL vial was charged with thiols (1.0 equiv., concentration maintained 10 mM) and **4b** (1.2 equiv.) in 200 mM Tris pH 8.0 (unless otherwise stated) with small magnetic stirring bar. The reaction mixture was stirred at rt for 2 h. The buffer was used directly from freshly prepared solution without degassing. After 2 h, an aliquot of 25 µL of the reaction mixture was diluted with 25 µL acetonitrile to give a clear solution. The solution was submitted to HPLC. The reported yields were calculated based on HPLC-MS chromatogram.

6. Substrate scope for small molecules

Preparation of triisopropyl((4-bromophenyl)ethynyl)silane 7a. A 5 mL vial was charged with 4-bromobenzene thiol (10.0 mg, 52.8 µmol, 1.0 equiv.), **4a** (43.5 mg, 79.3 µmol, 1.5 equiv.) and a stirring bar. Tris buffer (10 mM, pH 7.4, 3 mL) was then added and the resulting mixture stirred vigorously over 6 h at 37 °C. After 6 h, the reaction mixture was then diluted with 10 mL DCM and the organic layer was collected using a separating funnel. The solvent was evaporated and the crude residue was purified by column chromatography on Biotage (Büchi flashpure cartridge 12 g, Pentane) to afford pure **7a**. A low melting pure **7a** was obtained in 72% (14.0 mg, 38.0 µmol) yield.

Triisopropyl((4-bromophenyl)ethynyl)silane, **7a**

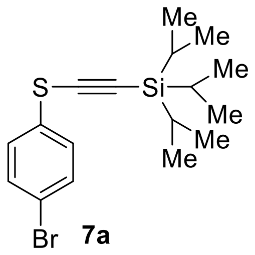

7a

^{1}H NMR (400 MHz, $CDCl_3$) δ 7.46 (d, *J* = 6.9 Hz, 2H, ArH), 7.31 (d, *J* = 8.5 Hz, 2H, ArH), 1.20 – 1.00 (m, 21H, TIPS).

^{13}C NMR (101 MHz, $CDCl_3$) δ 132.1 (ArC), 127.5 (ArC), 120.0 (ArC), 104.1 (CC), 90.2 (CC), 18.6 (CH_3), 11.3 (CH).

IR (ν_{max}, cm^{-1}) 2942 (s), 2926 (s), 2865 (s), 2094 (s), 1472 (s), 1388 (m), 1083 (s), 1070 (s), 1008 (s), 997 (m), 882 (s), 859 (s), 809 (s), 743 (s), 679 (s), 660 (s), 605 (s).

HRMS (APCI/QTOF) m/z: $[M]^+$ Calcd for $C_{17}H_{25}BrSSi^+$ 368.0624; Found 368.0499, 370.0487 $[M + 2]^+$.

Preparation of triisopropyl((naphthalen-2-ylthio)ethynyl)silane, 8a. A 5 mL vial was charged with naphthalene-2-thiol (10.0 mg, 62.4 µmol, 1.0 equiv.), **4a** (51.3 mg, 93.6 µmol, 1.5 equiv.) and as stirring bar. Tris buffer (10 mM, pH 7.4, 3 mL) was then added and the resulting mixture was stirred vigorously at 37 °C for 6 h. The buffer was used directly from freshly prepared bottle without degassing. Although the reaction solution was not clear due to the hydrophobic nature of naphthalene-2-thiol, no effect was observed on the rate of the reaction. After 6 h, the reaction mixture was diluted with 10 mL DCM and the organic layer was extracted using separating funnel. The organic layer dried over Na_2SO_4.The solvent was evaporated and the crude residue was purified by column chromatography on Biotage (Büchi flashpure cartridge 12 g, Pentane) to afford pure **8a** as low melting solid, yield 68% (14.5 mg, 42.5 µmol).

Triisopropyl((naphthalen-2-ylthio)ethynyl)silane, **8a**

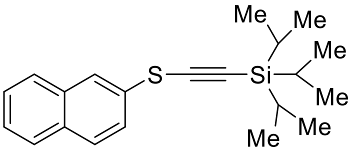

8a

.

^{1}**H NMR** (400 MHz, $CDCl_3$) δ 7.96 (d, *J* = 2.3 Hz, 1H, ArH), 7.81 (d, *J* = 8.8 Hz, 2H, ArH), 7.73 (d, *J* = 8.0 Hz, 1H, ArH), 7.52 – 7.42 (m, 3H, ArH), 1.21 – 1.10 (m, 21H, TIPS).

^{13}**C NMR** (101 MHz, $CDCl_3$) δ 133.8 (ArC), 131.9 (ArC), 130.1 (ArC), 128.8 (ArC), 127.8 (ArC), 127.0 (ArC), 126.8 (ArC), 125.8 (ArC), 124.3 (ArC), 123.9 (ArC), 103.6 (CC), 91.1 (CC), 18.7 (CH_3), 11.4 (CH).

IR (ν_{max}, cm^{-1}) 3056 (s), 2943 (s), 2865 (s), 2092 (s), 1626 (s), 1591 (s), 1503 (s), 1462 (s), 1071 (s), 996 (s), 882 (s), 859 (s), 851 (s), 742 (s), 680 (s), 661 (s), 604 (s).

HRMS $(APPI)^+$ m/z: $[M]^+$ Calcd for $C_{21}H_{28}SSi^+$ 340.1681; Found 340.1673.

Preparation of hexadec-1-yn-1-yl(naphthalen-2-yl)sulfane, 8b. A 5 mL vial was charged with naphthalene-2-thiol, **8** (10.0 mg, 62.4 µmol, 1.0 equiv.), **4b** (44.1 mg, 74.8 µmol, 1.2 equiv.) and magnetic bar. Then 3 mL 100 mM PB buffer pH 8.0 was then added and reaction mixture was stirred vigorously at rt for 2 h. The buffer was used directly from freshly prepared bottle without degassing. Although the reaction solution was not clear due to the hydrophobic nature of naphthalene-2-thiol, no effect was observed on the rate of the reaction. After 6 h, the reaction mixture was diluted with 20 mL diethyl ether and the organic layer was extracted using separating funnel. Then organic layer dried over Na_2SO_4, and diethyl ether evaporated in rotary evaporator under reduced pressure. The crude residue was purified by column chromatography on Biotage (Büchi flashpure cartridge 12 g, Pentane) to afford pure **8b** as colorless liquid, yield 29% (7.0 mg, 18 µmol).

Hexadec-1-yn-1-yl(naphthalen-2-yl)sulfane, **8b**

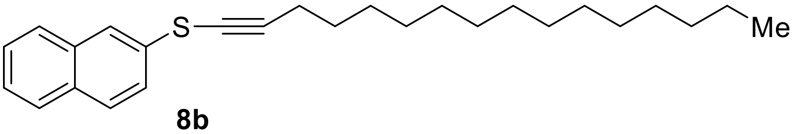

8b

^{1}**H NMR** (400 MHz, $CDCl_3$) δ 7.87 (brs, 1H, ArH), 7.83 – 7.71 (m, 3H, ArH), 7.53 – 7.37 (m, 3H, ArH), 2.50 (t, *J* = 6.3 Hz, 2H, CH_2), 1.70 – 1.60 (m, 2H, CH_2), 1.54 – 1.20 (m, 22H, 11×CH_2), 0.88 (t, *J* = 7.4, 3H, CH_3).

^{13}**C NMR** (101 MHz, $CDCl_3$) δ 133.7 (ArC), 131.8 (ArC), 131.20 (ArC), 128.6 (ArC), 127.7 (ArC), 127.0 (ArC), 126.7 (ArC), 125.6 (ArC), 123.9 (ArC), 123.8 (ArC), 100.3 (CC), 64.5 (CC), 31.9 (CH_2), 29.6 (5×CH_2), 29.5 (CH_2), 29.3 (CH_2), 29.1 (CH_2), 28.9 (CH_2), 28.6 (CH_2), 22.6 (CH_2), 20.3 (CH_2), 14.1 (CH_3).

HRMS (APCI/QTOF) m/z: $[M + H]^+$ Calcd for $C_{26}H_{37}S^+$ 381.2610; Found 381.2613.

IR (ν_{max}, cm^{-1}) 3154 (m), 3058 (m), 2927 (m), 2859 (m), 2253 (m), 1793 (m), 1458 (m), 1380 (m), 903 (s), 722 (s).

Reaction procedure for alkynylation of homocysteine **9** using **4a**

SUPPORTING INFORMATION

A 1.5 mL vial was charged with homocysteine (**9**, 0.50 mg, 3.9 µmol, 1.0 equiv.) and **4a** (3.0 mg, 5.5 µmol, 1.5 equiv.) in 726 µL 10 mM Tris pH 7.4 with small magnetic stirring bar. The reaction mixture was stirred at 37 °C for 6 h. After 2 h, an aliquot of 25 µL of the reaction mixture was diluted with 25 µL acetonitrile to give a clear solution. The solution was submitted to HPLC. According to HPLC-MS chromatogram 95% alkynylated product (**9a**) and 5% disulfide were obtained.

S-((triisopropylsilyl)ethynyl)homocysteine, **9a**

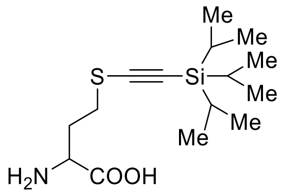

9a

HRMS (ESI/QTOF) m/z: $[M+H]^+$ Calcd for $C_{15}H_{30}NO_2SSi^+$ 316.1761; Found 316.1753.

HPLC gradient: Method 2.

HPLC-UV and HPLC-MS chromatogram

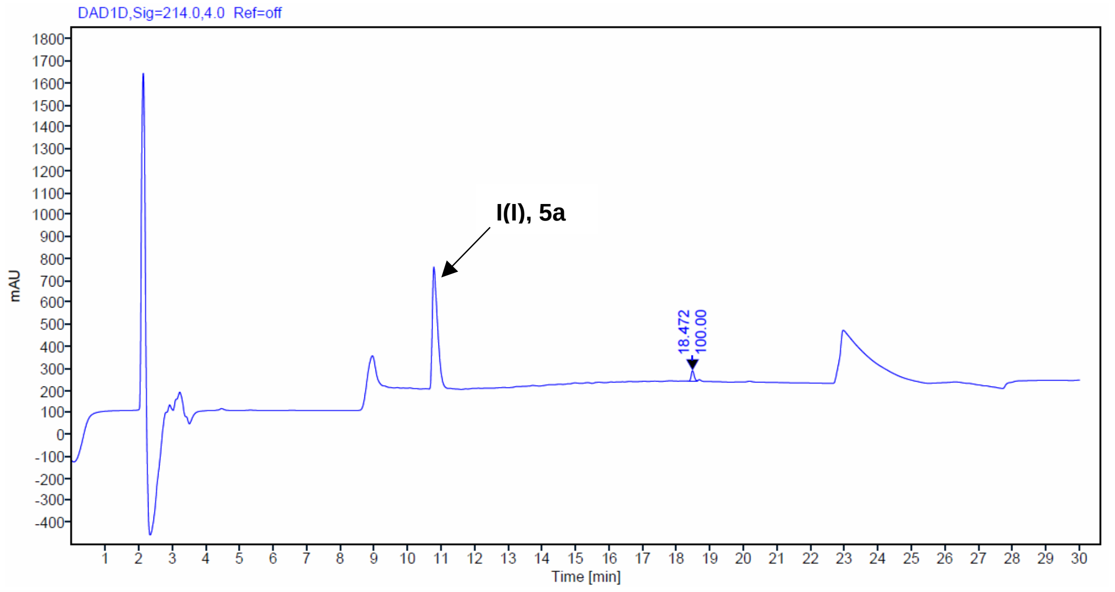

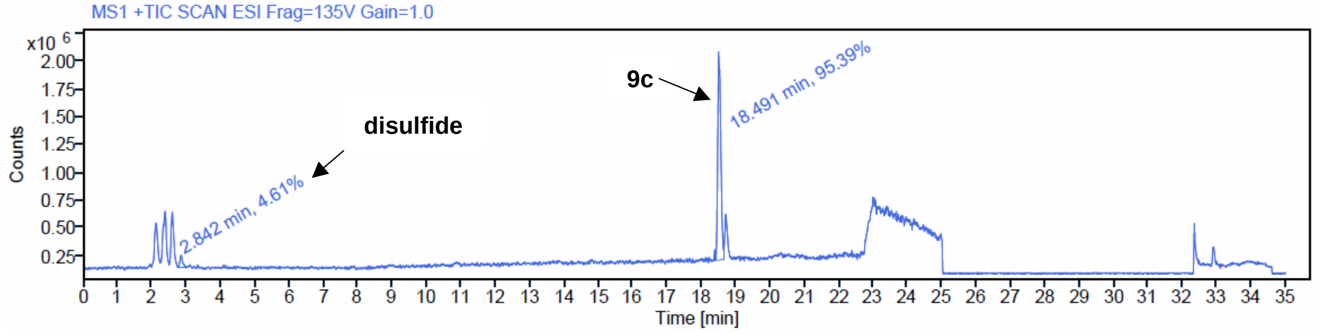

Reaction procedure for alkynylation of homocysteine **9** using **4b**

SUPPORTING INFORMATION

A 1.5 mL vial was charged with homocysteine (**9**, 0.50 mg, 3.9 µmol, 1.0 equiv.) and **4b** (2.6 mg, 4.4 µmol, 1.2 equiv.) in 726 µL 200 mM Tris pH 8.0 with small magnetic stirring bar. The reaction mixture was stirred at rt for 2 h. After 2 h, an aliquot of 25 µL of the reaction mixture was diluted with 25 µL acetonitrile to give a clear solution. The solution was submitted to HPLC. According to HPLC-MS chromatogram 66% alkynylated product **9b** (retention time = 19.0) and 34% VBX **9c** (retention time = 16.1 were obtained.

S-(hexadec-1-yn-1-yl)homocysteine, **9b** and VBX of homocysteine, **9c**

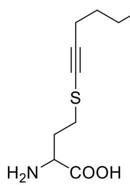

9b

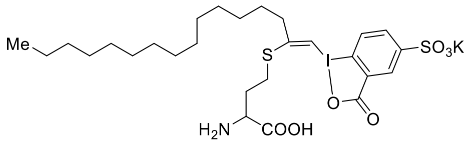

9c

HPLC gradient: Method 1.

HRMS (ESI/QTOF) m/z for **9b**: $[M + H]^+$ Calcd for **9b** $C_{20}H_{38}NO_2S^+$ 356.2618; Found 356.2622.

HRMS (ESI/QTOF) m/z: $[M]^-$ Calcd for **9c** $C_{27}H_{41}INO_7S_2^-$ 682.1375; Found 682.1370.

HPLC-UV and HPLC-MS chromatogram

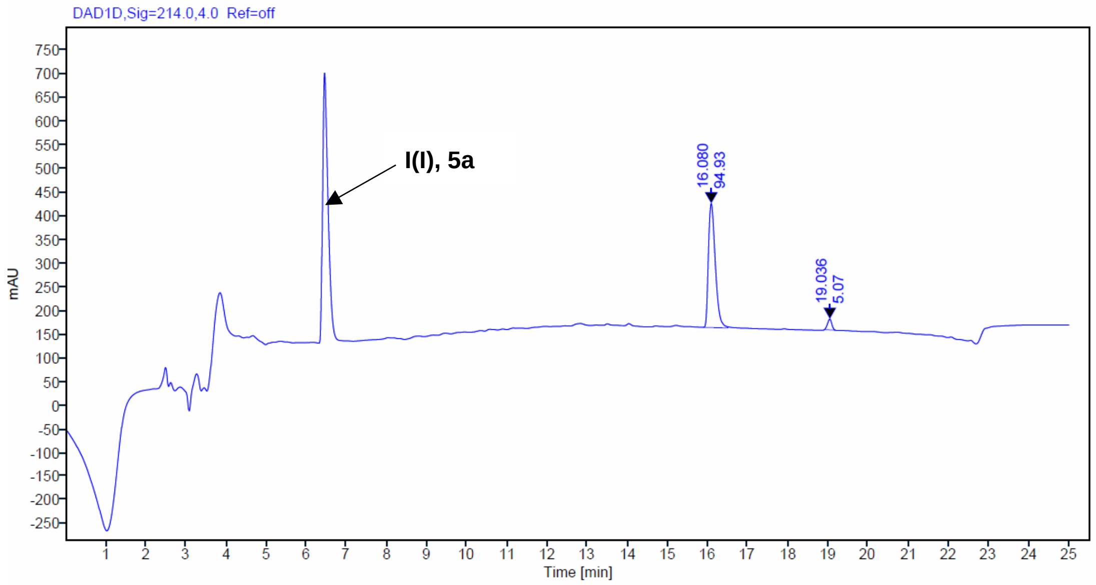

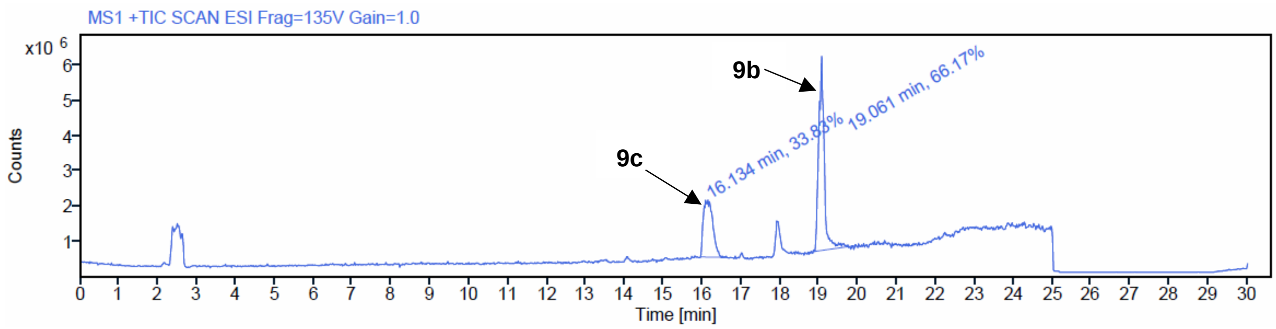

SUPPORTING INFORMATION

S-((triisopropylsilyl)ethynyl)cysteine, **10a**

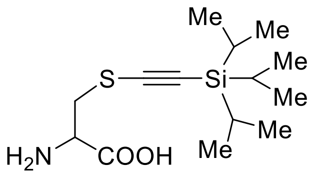

10a

cysteine **10** (0.20 mg, 1.6 µmol) and **4a** (1.3 mg, 2.6 µmol) in 165 µL 10 mM Tris pH 7.4. Adopted General reaction procedure for **4a**. Yield 90% (retention time = 13.53)

HRMS (ESI/QTOF) m/z: $[M+H]^+$ Calcd for $C_{14}H_{28}NO_2SSi^+$ 302.1605; Found 302.1597.

HPLC gradient: Method 1.

HPLC-UV and HPLC-MS chromatogram

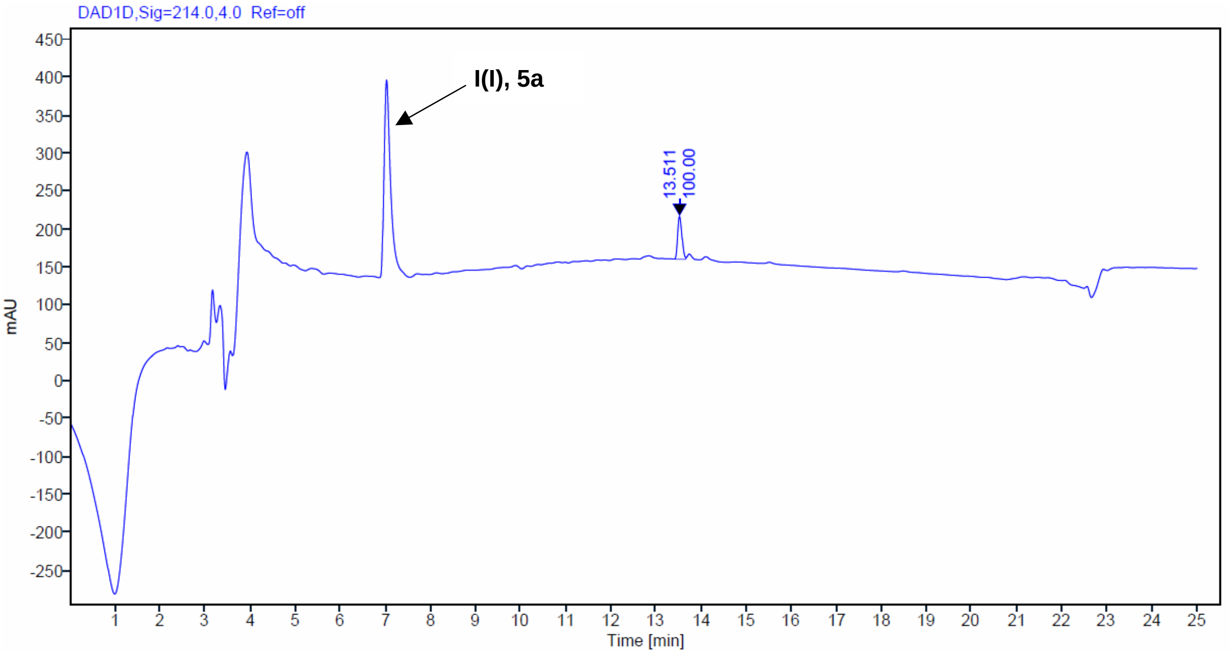

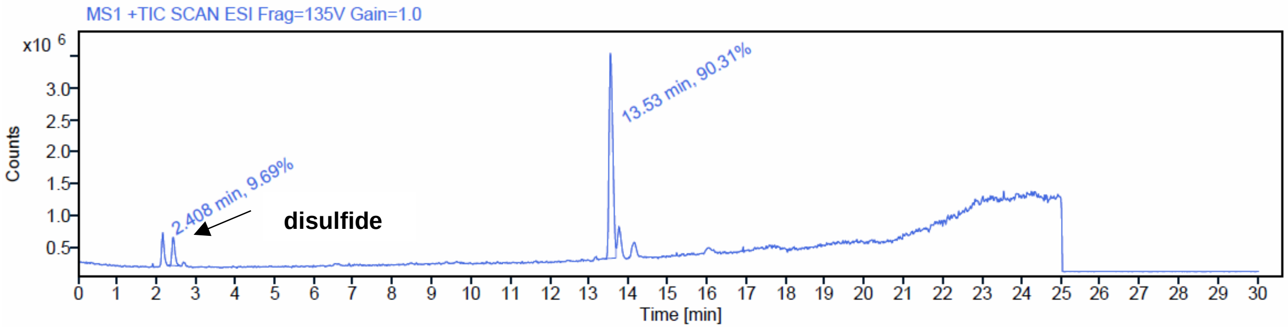

S-(heptadec-1-yn-1-yl)cysteine, **10b**, VBX of cysteine, **10c**

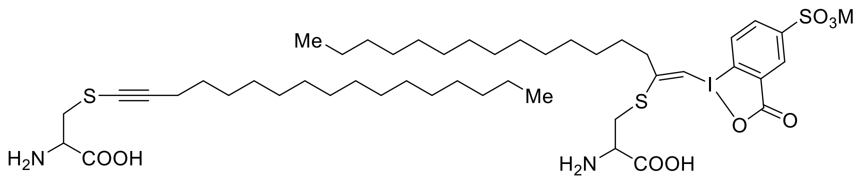

10b **10c**

Cysteine **10** (0.20 mg, 1.6 µmol) and **4b** (1.2 mg, 1.9 µmol) in 165 µL 10 mM Tris pH 7.4. Adapted General reaction procedure. After 2 h; Yield for **10b** 62% (retention time = 19.4) and **10c,** 38% (retention time = 16.9).

HRMS (ESI/QTOF) m/: [M + H]$^+$ Calcd for **10b**, $C_{19}H_{36}NO_2S^+$ 342.2461; Found 342.2478.

HRMS (ESI/QTOF) m/z: [M]$^-$ Calcd for **10c** $C_{26}H_{39}INO_7S_2^-$ 668.1218; Found 668.1210.

HPLC-MS chromatogram 2 h

HPLC gradient: Method 1

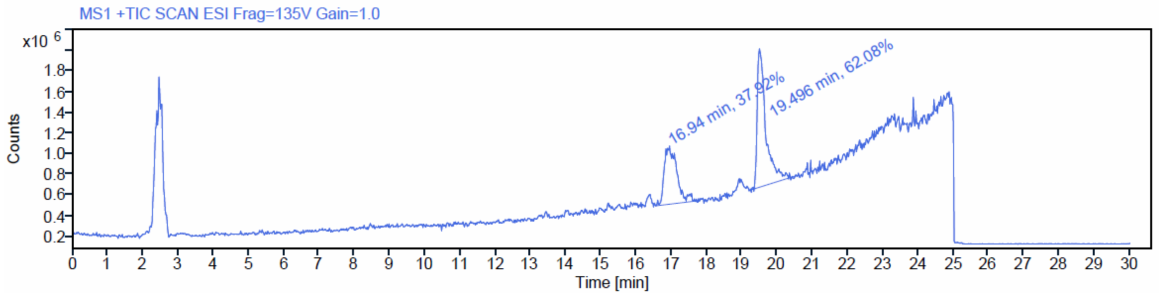

HPLC-MS chromatogram 6 h

HPLC gradient: Method 2.

After 6 h; quantitative yield observed for **10b** (retention time = 24.5).

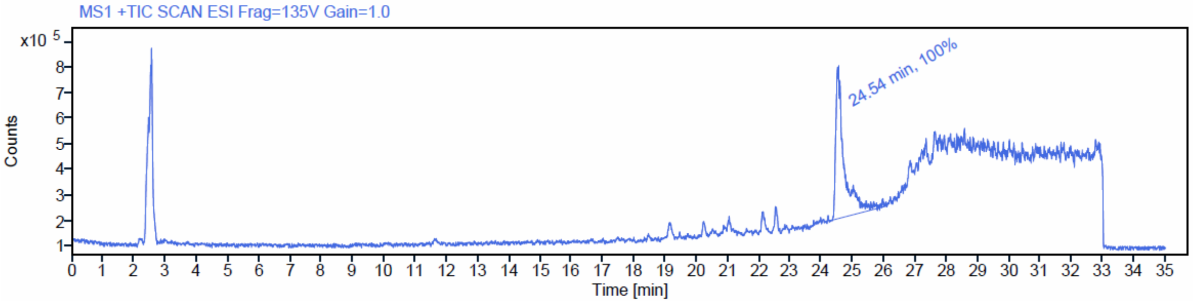

7. Substrate scope for tetra and hexapeptides

Procedure for preparation of **11a**

A 5 mL vial was charged with **11** (15.0 mg, 34.2 µmol, 1.0 equiv.) and **4a** (28.2 mg, 51.4 µmol, 1.5 equiv.) and magnetic bar. Then in 3.3 mL of Tris buffer (10 mM, pH 7.4) added, followed by reaction mixture was stirred at 37 °C for 10 h. No effort was made to exclude oxygen. The reaction mixture was diluted was directly submitted to HPLC-MS as it was clear solution. On the basis of HPLC-MS 82% (retention time = 13.7) yield was observed for **11a**. Pure **11a** was isolated by prep RP-HPLC using Method 6. The pure fraction was collected and lyophilized for two days. A white solid obtained with 36% yield (7.5 mg, 12 µmol, retention time = 14.5 min), 79% calibrated yield.

Ac-Cys-Gly-Phe-NH_2 (**11a**)

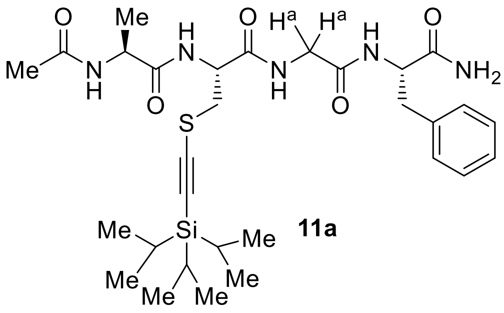

11a

HPLC Gradient: Method 3 (analytical HPLC), Method 6 (preparative HPLC).

HPLC-UV and HPLC-MS chromatogram of **11a**

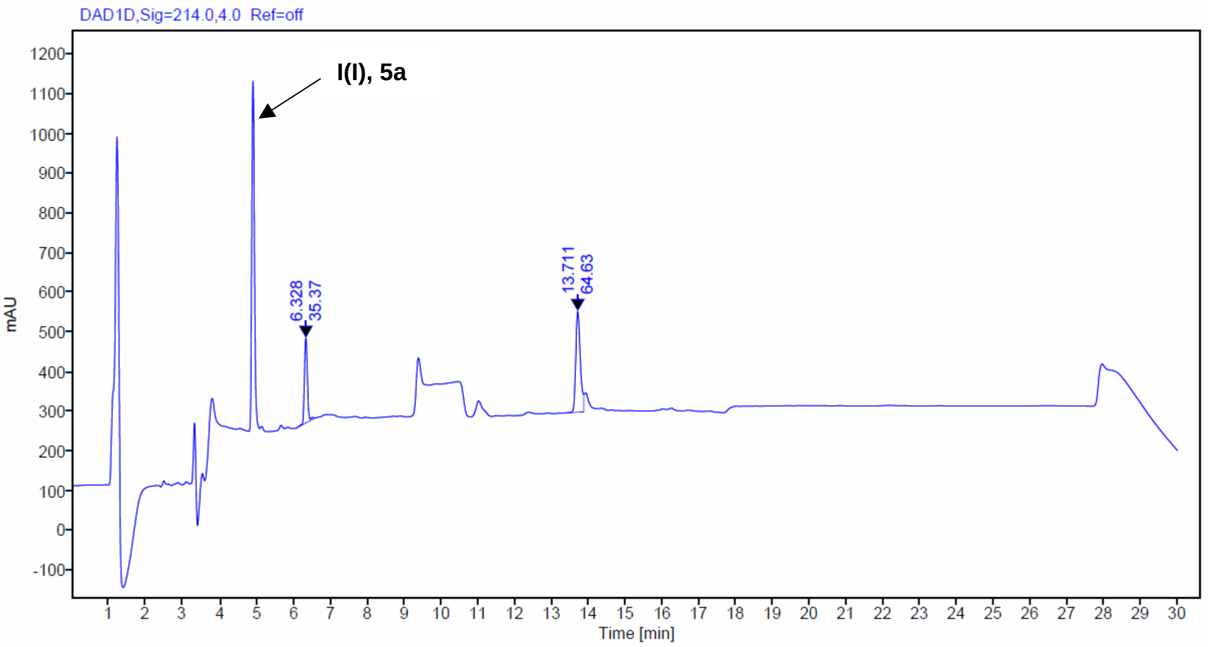

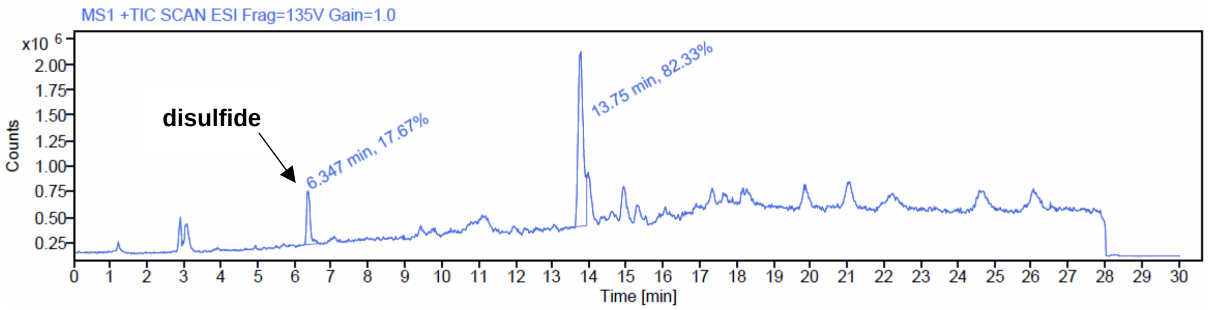

^{1}H NMR (400 MHz, MeOD) δ 7.26 – 6.98 (m, 5H, ArH), 4.61 – 4.50 (m, 2H, CH_2), 4.30 – 4.15 (m, 1H, CH), 3.96 (d, *J* = 16.7 Hz, 1H, CH[a]), 3.65 (d, *J* = 16.7 Hz, 1H, CH[a]), 3.45 (dd, *J* = 13.3, 4.6 Hz, 1H, CH), 3.20 (dd, *J* = 14.0, 5.0 Hz, 1H, CH), 3.06 – 2.90 (m, 2H, CH_2), 1.89 (s, 3H, CH_3), 1.29 (d, *J* = 7.2 Hz, 3H, CH_3), 0.99 (d, *J* = 3.0 Hz, 21H, TIPS).

^{19}F NMR (376 MHz, MeOD) δ -77.7 (trace amount of CF_3COOH).

^{13}C NMR (101 MHz, MeOD) δ 175.4 (C=O), 175.3 (C=O), 173.7 (C=O), 171.9 (C=O), 170.7 (C=O), 137.9 (ArC), 129.6 (ArC), 128.8 (ArC), 127.0 (ArC), 98.2 (CC), 95.4 (CC), 55.4 (CH), 53.5 (CH), 50.8 (CH), 43.0 (CH_2), 38.0 (CH_2), 37.2 (CH_2), 21.9 (CH_3), 18.4 (CH_3), 16.9 (CH), 11.8 (CH_3).

HRMS (nanochip-ESI/LTQ-Orbitrap) m/z: $[M + H]^+$ Calcd for $C_{30}H_{48}N_5O_5SSi^+$ 618.3140; Found 618.3115.

HRMS (ESI/QTOF) m/z: $[M + Na]^+$ Calcd for $C_{30}H_{47}N_5NaO_5SSi^+$ 640.2959; Found 640.2961.

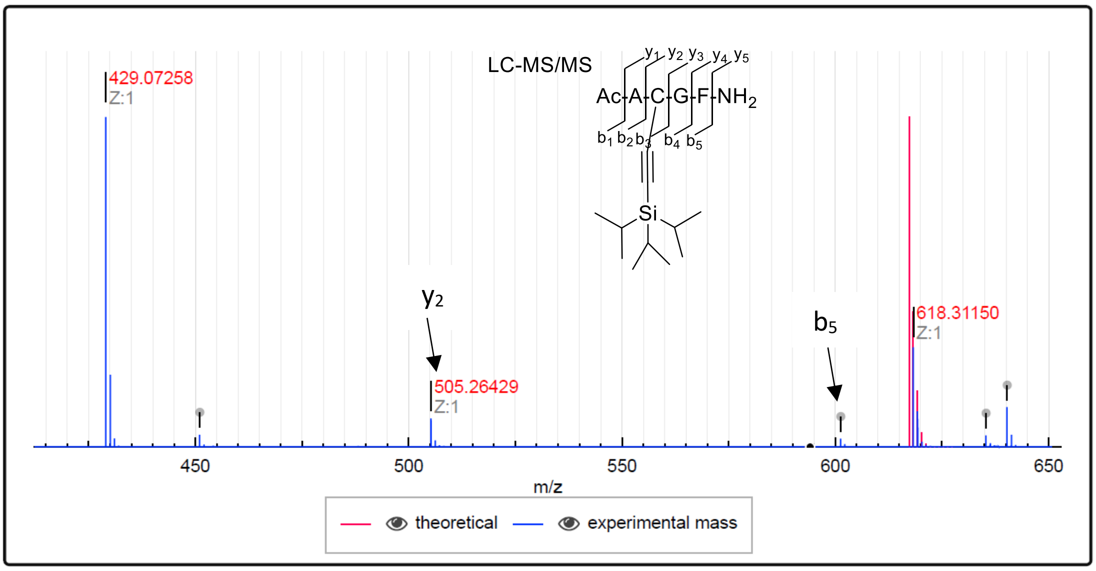

Figure S2. LC-MS/MS of **11a**.

Calibration curve of **11a**

Calibration with **11a** was achieved through the preparation of several samples of different concentrations and their analysis on RP HPLC. These analyses were repeated three times in order to obtain an average curve of calibration. The following linear regression was obtained: Y = 470.12 X + 284.02 and R = 0.9887, where Y is the absorption in $mAUs^{-1}$ at 214 nm and X the concentration of **11a** in mM.

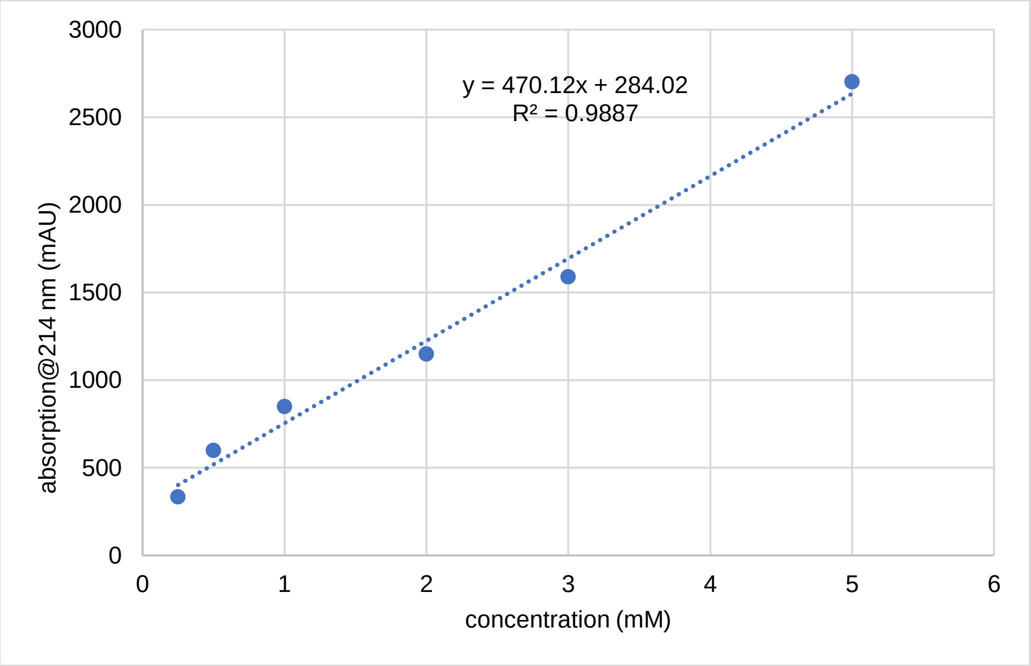

Figure S3. calibration curve of **11a.**

Calculation of Partition coefficient (LogP)[4]

2.5 mg (4.0 µmol) **11a** was dissolved in 1.7 mL octanol-H_2O mixture (0.85 mL 1-Octanol + 0.85 mL milli-Q H_2O) in a 5 mL vial. In order to mix the layers, the vial was shaken on vertex for 1 min. Then the mixture was transferred to a separating funnel. The separating funnel was closed and left standing for 3 h to separate the organic and the H_2O layer. Both layers were taken separately and submitted tonreverse phase HPLC. The concentration of both layers was determined by HPLC-UV (absorption 214 nm).

Amount of **11a** in 1-octanol = 2.41 mg/mL

Amount of **11a** in H_2O = 0.09 mg/mL

Partition coefficient (P) = amount of **11a** in 1-octanol/ amount of **11a** in H_2O

P = (2.41mg/mL)/(0.09mg/mL)

P = 26.78   or   LogP = Log(26.78)

LogP = **1.43**

Following the same procedure for starting material **11**: LogP = -1.53

[4] J. R. Espinosa, C. R. Wand, C. Vega, E. Sanz, D. Frenkel, *J. Chem. Phys*. **2018**, *149*, 224501.

SUPPORTING INFORMATION

Alkynylated Ac-Ala-Cys-Gly-Phe-NH_2 (**11b**)

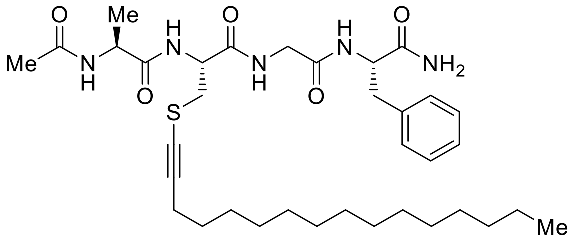

11b

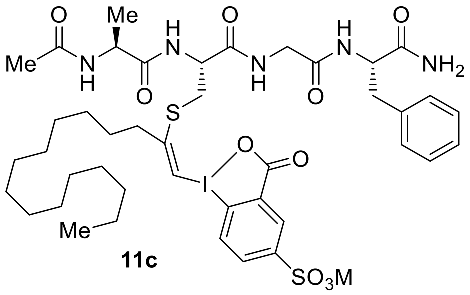

11c

11 (1.0 mg, 2.3 µmol) and **4b** (1.6 mg, 2.7 µmol) in 115 µL 200 mM Tris pH 7.4. Adapted General reaction procedure for **4b**. Yield for **11b** 82% (retention time = 20.2), **11c** 18% (retention time = 11.54)

HRMS (ESI/QTOF) m/z: $[M + H]^+$ Calcd for **11b** $C_{14}H_{28}NO_2SSi^+$ 302.1605; Found 302.1597.

HRMS (ESI/QTOF) m/z: $[M]^-$ Calcd for **11c** $C_{42}H_{59}IN_5O_{10}S_2^-$ 984.2754; Found 984.2731.

HPLC gradient: Method 3.

HPLC-UV and HPLC-MS chromatogram

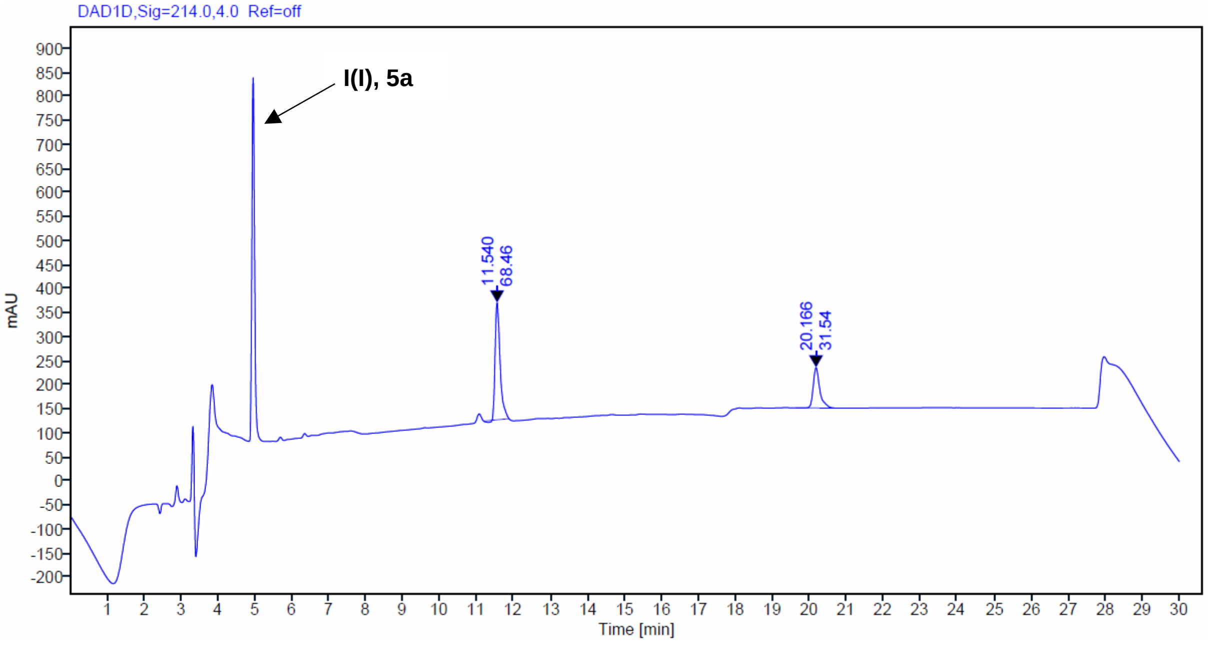

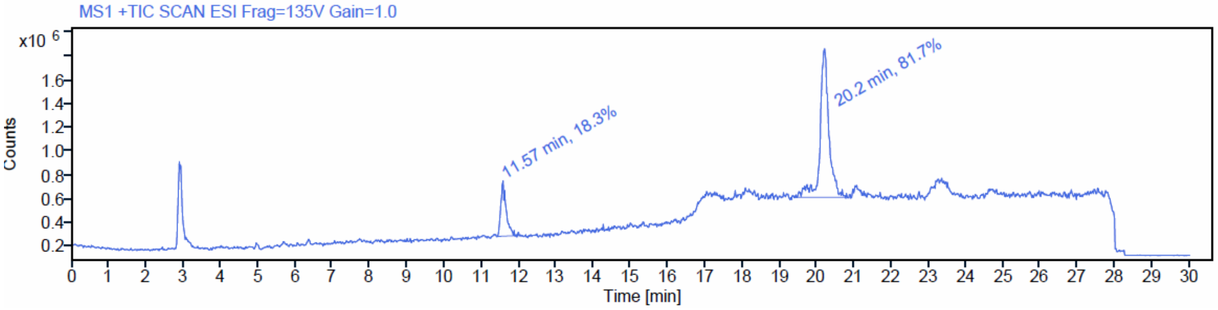

Alkynylated H-Ala-Cys-Phe-Gly-Ala-Leu-NH_2 with (**12a**)

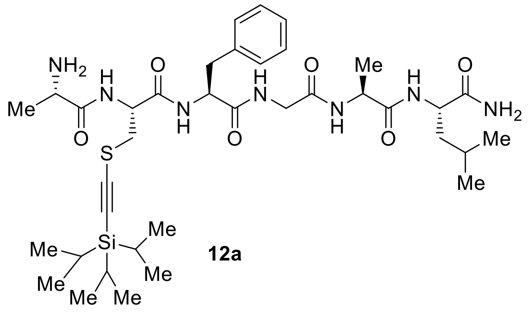

12a

12 (0.50 mg, 0.86 µmol) and **4a** (0.70 mg, 1.3 µmol) in 86 µL 10 mM Tris pH 7.4. Adapted General reaction procedure for **4a**. Yield for **12a** 86% (retention time = 13.9)

HRMS (ESI/QTOF) m/z: $[M + H]^+$ Calcd for **12a** $C_{37}H_{62}N_7O_6SSi^+$ 760.4246; Found 760.4253.

HPLC gradient: Method 1.

HPLC-UV and HPLC-MS chromatogram

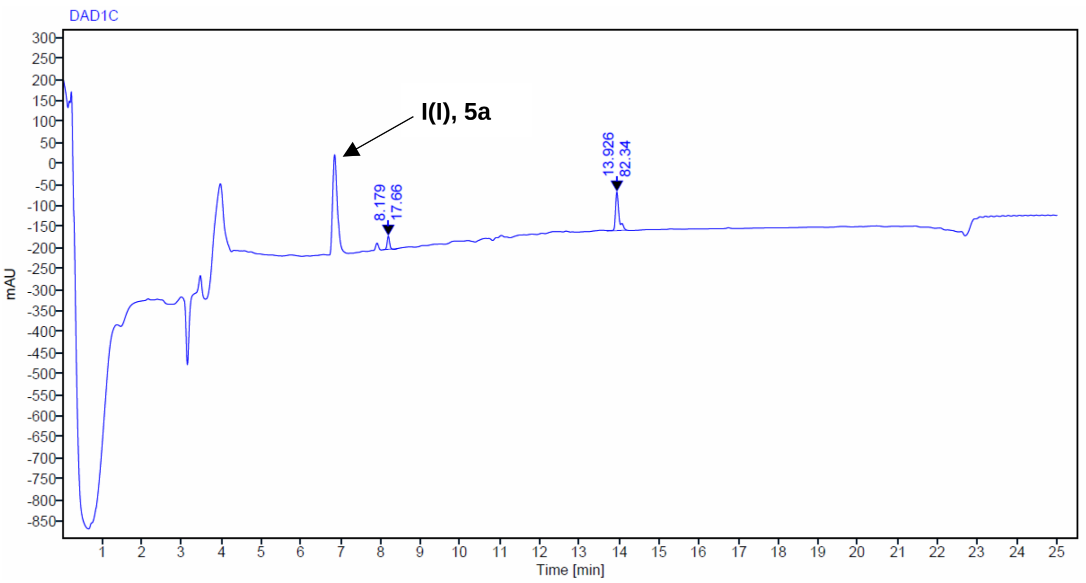

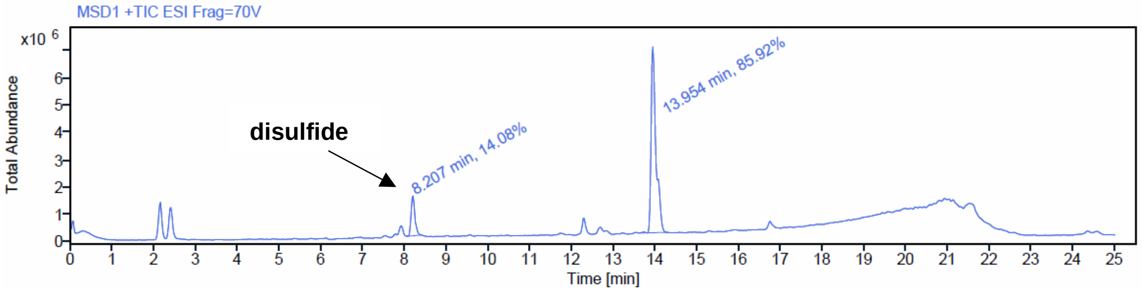

Alkynylated H-Ala-Cys-Phe-Gly-Ala-Leu-NH_2 with (**12b**) and corresponding VBX (**12c**)

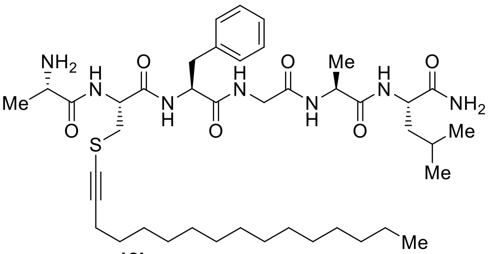
12b

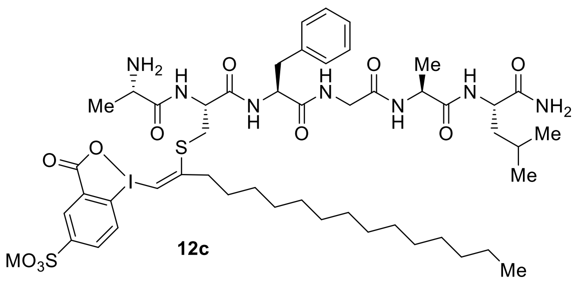
12c

12 (5.0 mg, 8.0 µmol) and **4b** (6.1 mg, 10.0 µmol) in 0.85 mL 200 mM Tris pH 8.0. Adapted general reaction procedure for **4b**. Yield for **12b** 82% (retention time = 17.2), **12c** 15 % (retention time = 16.8). Isolated yield of **12b**: 36% (2.5 mg, 2.8 µmol, retention time 17-19 min).

HRMS (ESI/QTOF) m/z: [M + H]$^+$ Calcd for **12b** $C_{42}H_{70}N_7O_6S^+$ 800.5103; Found 800.5098.

HRMS (ESI/QTOF) m/z: [M]$^-$ Calcd for **12c** $C_{49}H_{73}IN_7O_{11}S_2^-$ 1126.3860; Found 1126.3880.

HPLC gradient: Method 1. **Prep HPLC gradient**: Method 7.

HPLC-UV and HPLC-MS chromatogram

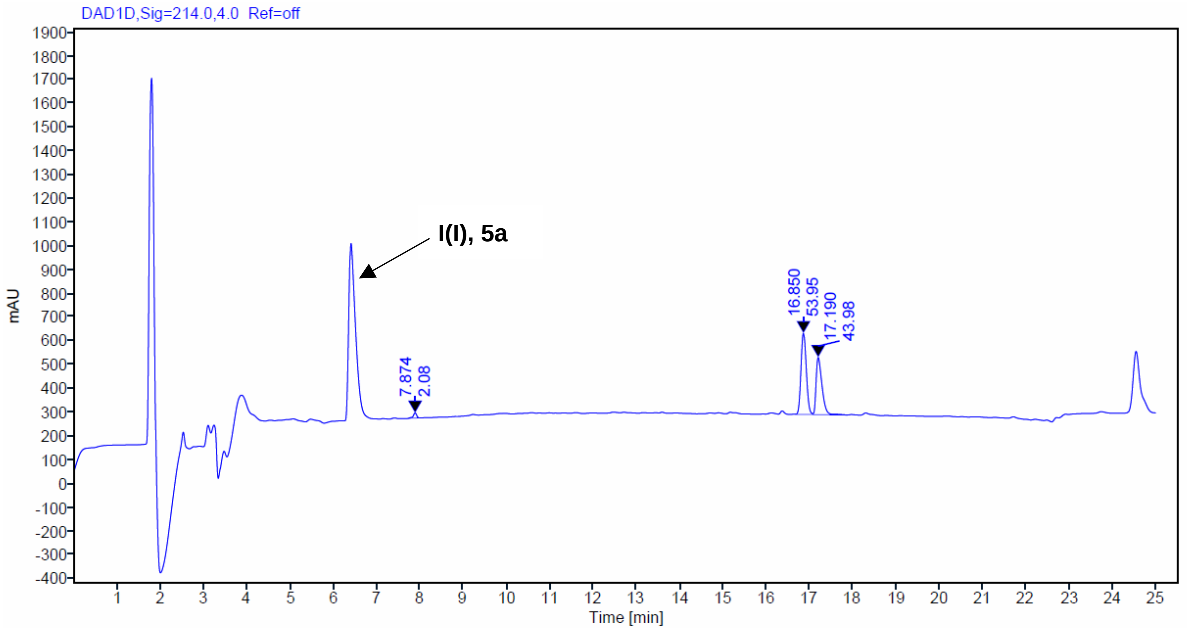

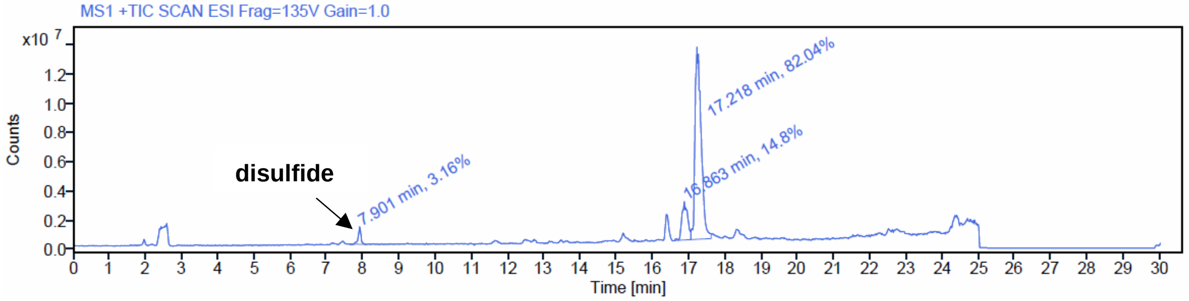

Calibration curve of **12b**

Preparation of stock solution. Pure **12b** (0.5 mg) dissolved in 183 uL of CH_3CN: H_2O (1:1). Stock solution was diluted separately and submitted for RP-HPLC. Calibrated yield 70% (based on HPLC-UV), 90% (based on HPLC-MS).

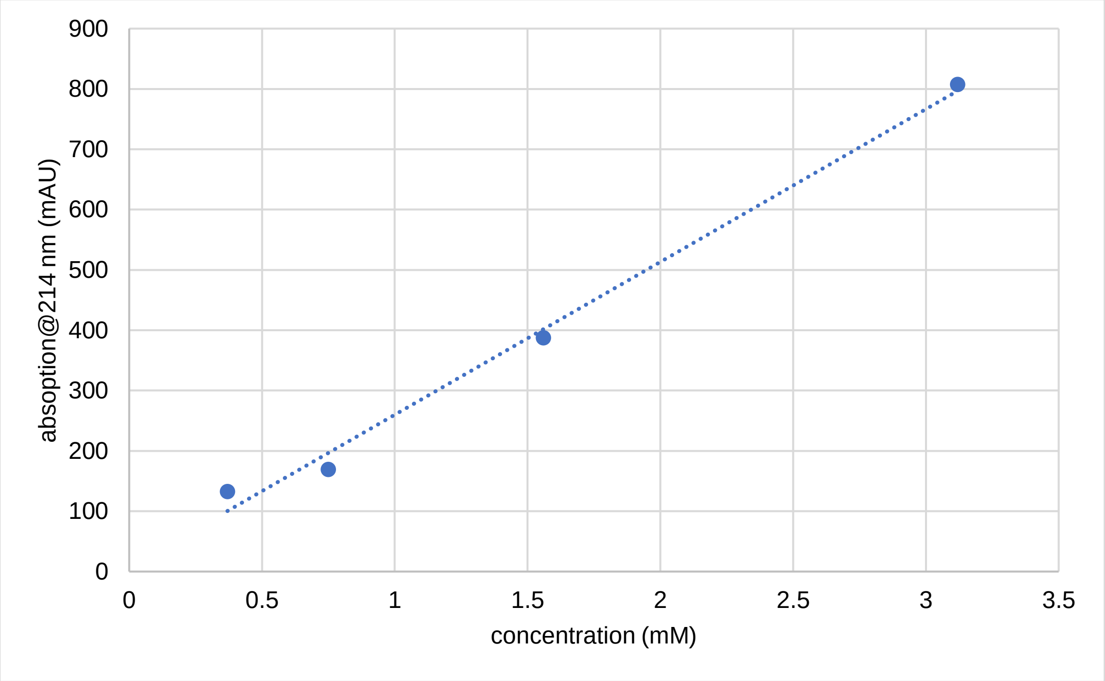

Figure S4. calibration curve of **12b** based on absorption at 214 nm.

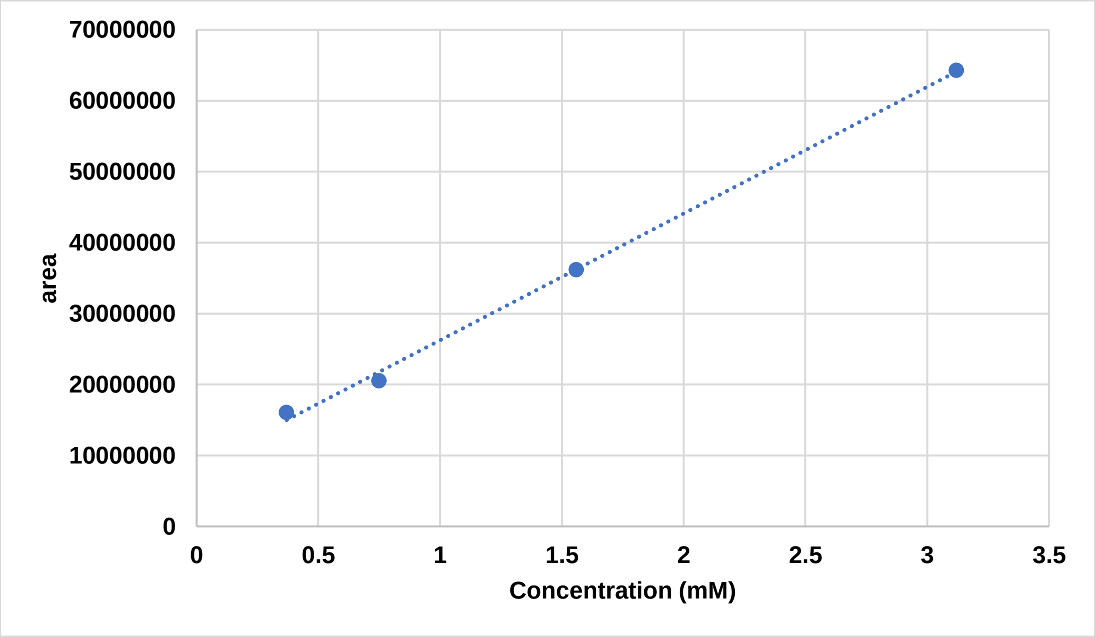

Figure S5. calibration curve of **12b** based on HPLC-MS.

SUPPORTING INFORMATION

Alkynylated H-Ala-Leu-Phe-Cys-Ala-Leu-NH_2 (**13a**)

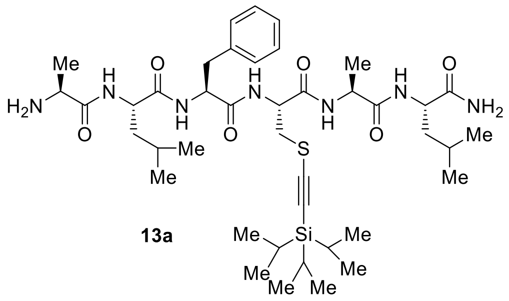

13a

13 (0.50 mg, 0.78 µmol) and **4a** (0.64 mg, 1.2 µmol) in 79 µL 10 mM Tris pH 7.4. Adapted general reaction procedure for **4a**. Yield for **13a** 61% (retention time = 14.7)

HRMS (ESI/QTOF) m/z: $[M + H]^+$ Calcd for **13a** $C_{41}H_{70}N_7O_6SSi^+$ 816.4872; Found 816.4877.

HPLC gradient: Method 1.

HPLC-UV and HPLC-MS chromatogram

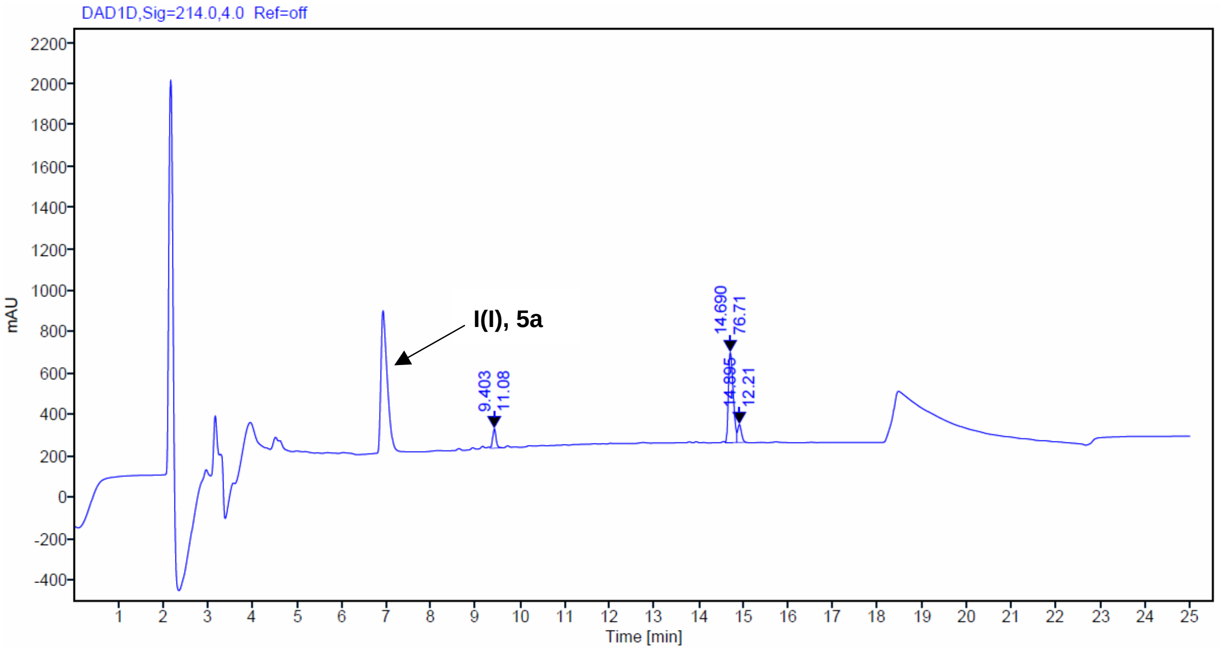

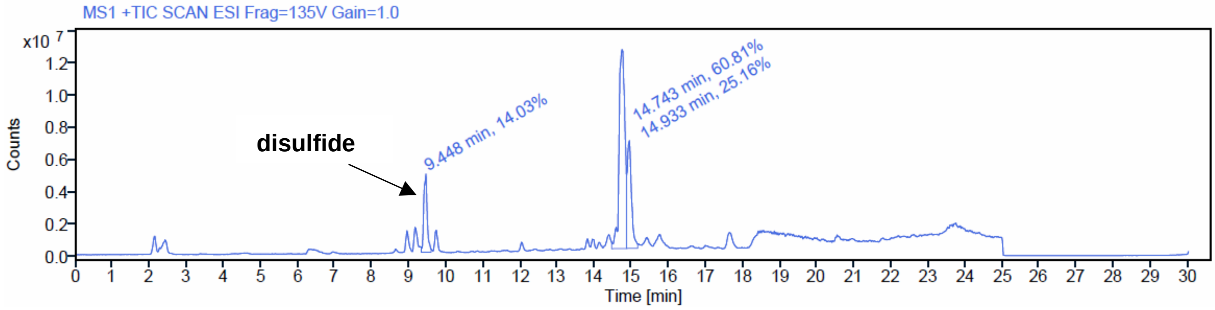

Alkynylated H-Ala-Leu-Phe-Cys-Ala-Leu-NH_2 (**13b**) and corresponding VBX (**13c**)

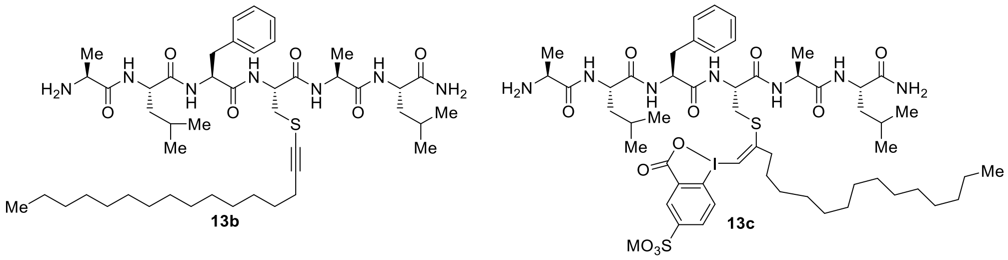

13 (15.0 mg, 23.5 µmol) and **4b** (11.1 mg, 18.8 µmol) in 1.5 mL 200 mM Tris pH 8.0. Adapted general reaction procedure for **4b**. Yield for **13b** 76% (retention time = 18.1), **13c** 18% (retention time = 17.2). Isolated yield of **13b**: 40% (7.8 mg, 9.4 µmol retention time = 16-18 min.)

HRMS (ESI/QTOF) m/z: $[M + H]^+$ Calcd for **13b** $C_{46}H_{78}N_7O_6S^+$ 856.5729; Found 856.5723.

HRMS (nanochip-ESI/LTQ-Orbitrap) m/z: $[M]^-$ for **13c** Calcd for $C_{53}H_{81}IN_7O_{11}S_2^-$ 1182.4486; Found 1182.4523.

HPLC gradient: Method 1. **Prep HPLC gradient**: Method 7.

HPLC-UV and HPLC-MS chromatogram

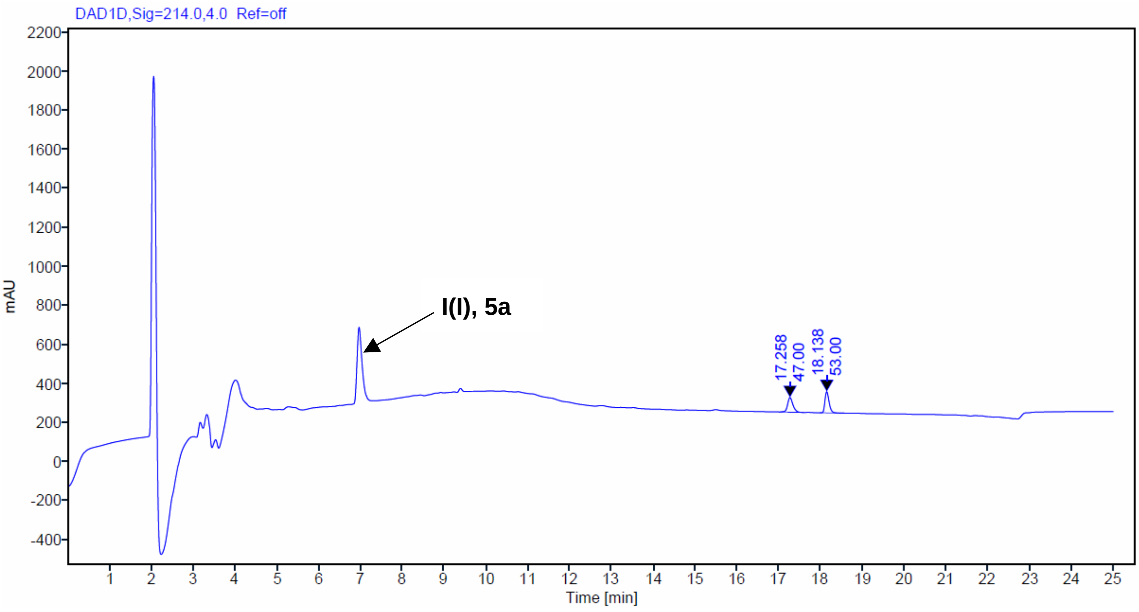

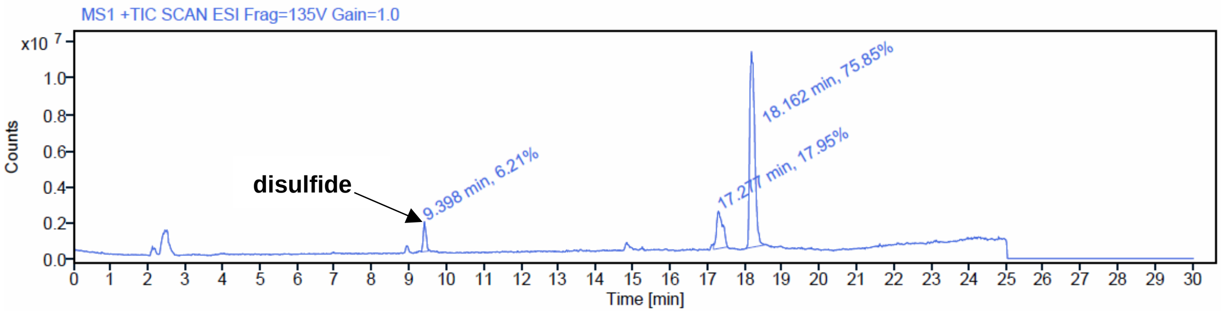

SUPPORTING INFORMATION

Calibration curve of **13b**

Preparation of stock solution. Pure **13b** (0.5 mg) dissolved in 73 uL of CH_3CN: H_2O (1:1). Stock solution was diluted separately and submitted for RP-HPLC. Calibrated yield 78% (based on HPLC-UV), 69% (based on HPLC-MS).

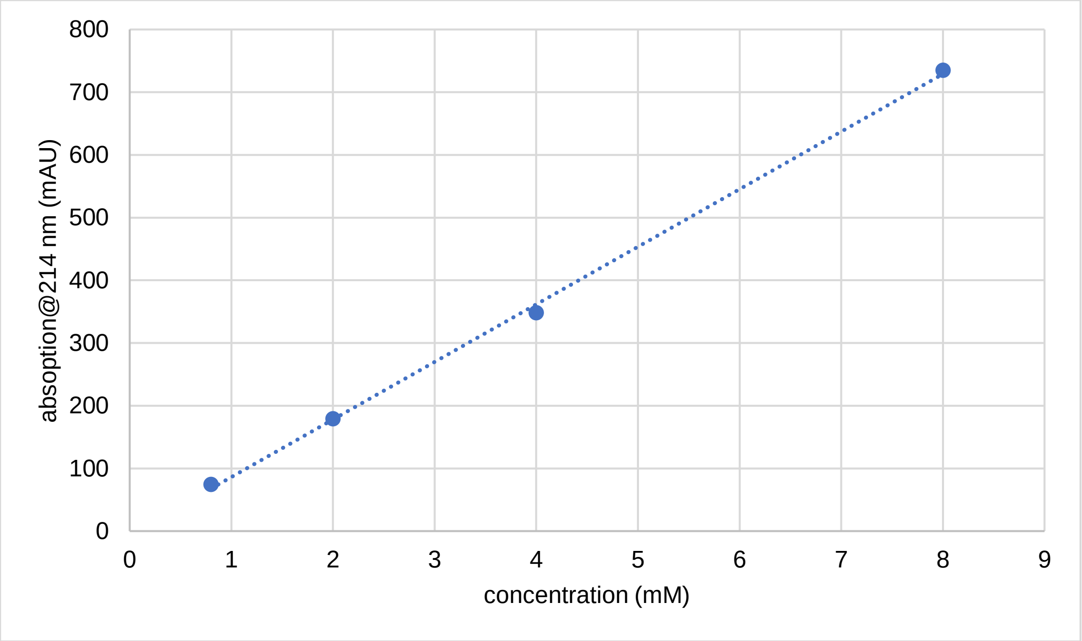

Figure S6. calibration curve of **13b** based HPLC-UV absorption at 214 nm.

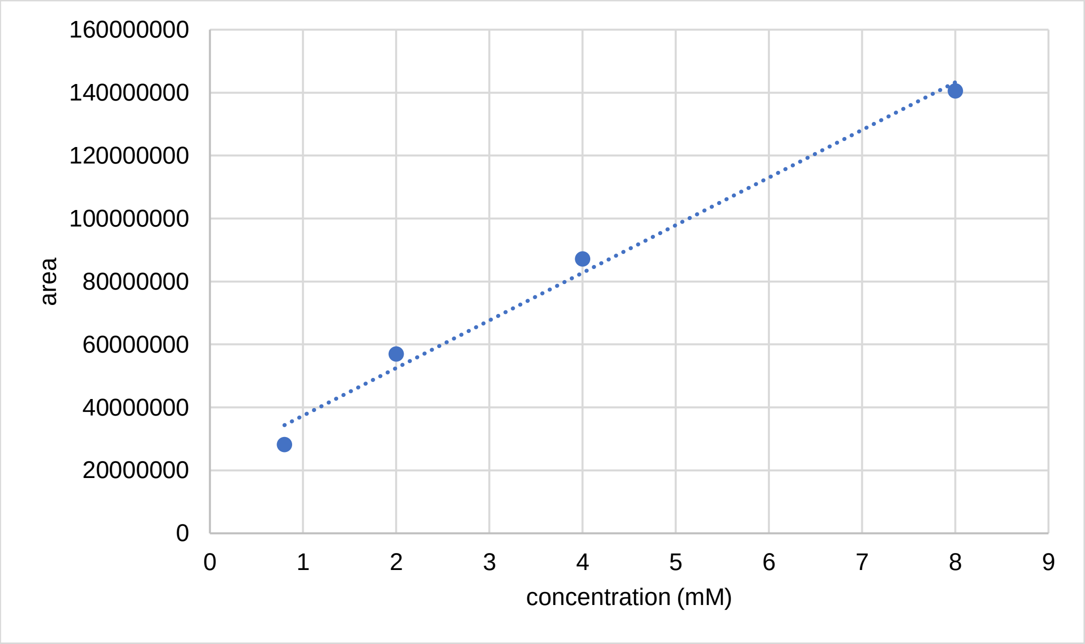

Figure S7. calibration curve of **13b** based HPLC-MS.

SUPPORTING INFORMATION

Alkynylated H-Phe-Cys-Phe-Lys-Ala-Leu-NH_2 (**14a**)

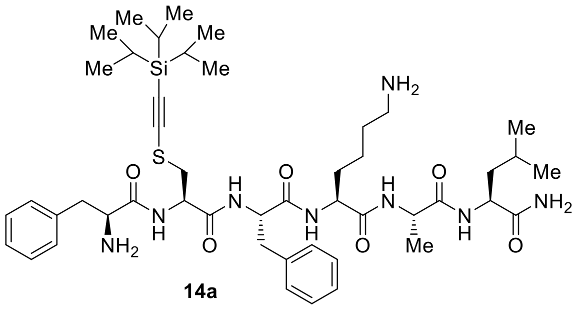

14a

14 (1.0 mg, 1.4 µmol) and **4a** (1.1 mg, 2.0 µmol) in 137 µL 10 mM Tris pH 7.4. Adapted general reaction procedure for **4a**. Yield for **14a** 89% (retention time = 12.0)

HRMS (ESI/QTOF) m/z: $[M + H]^+$ Calcd for **14a** $C_{47}H_{75}N_8O_6SSi^+$ 907.5294; Found 907.5328.

HPLC gradient: Method 1.

HPLC-UV and HPLC-MS chromatogram

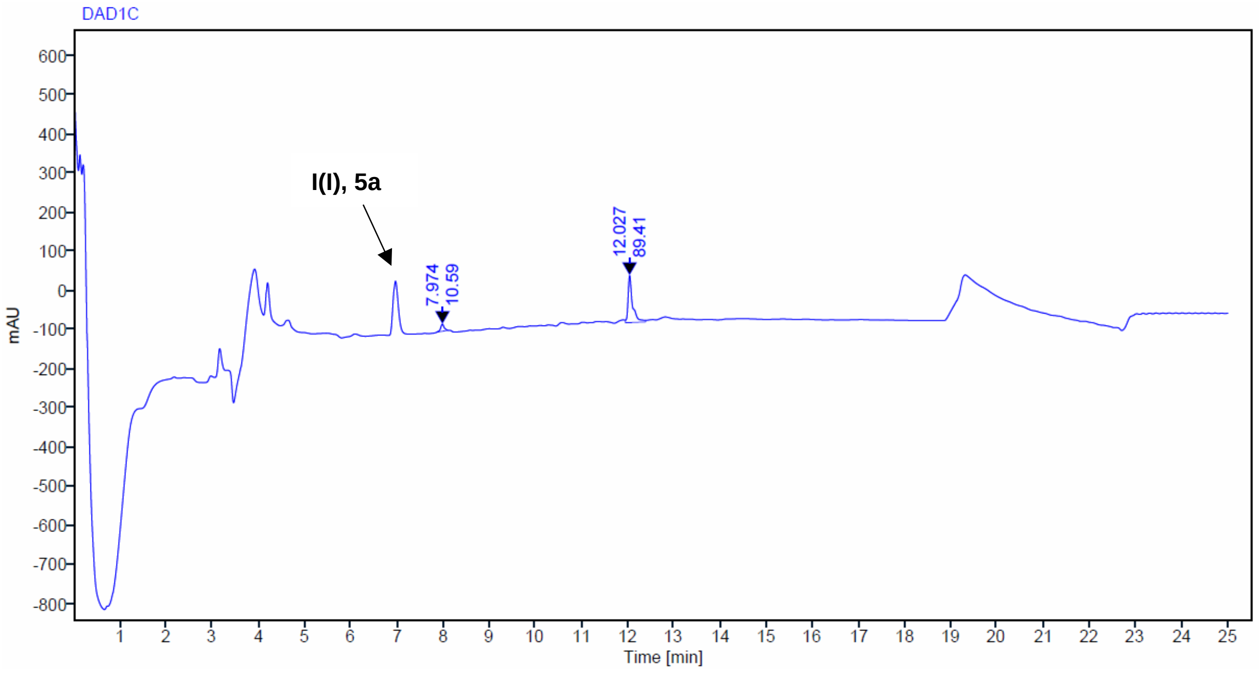

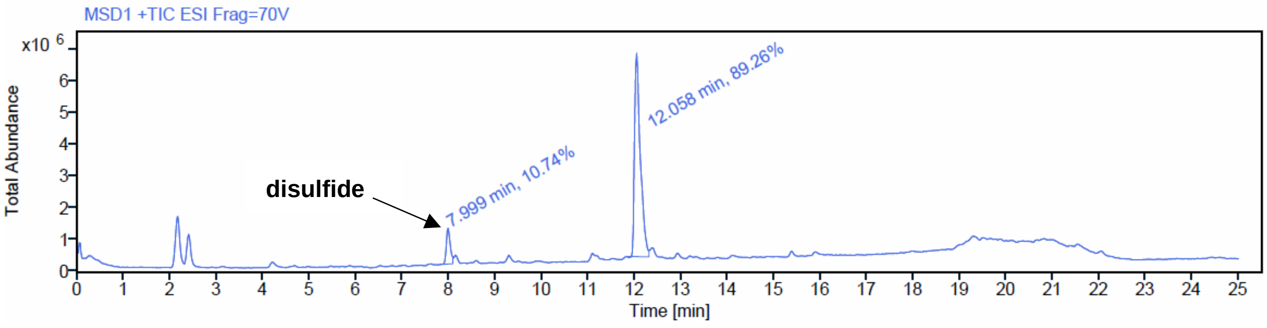

SUPPORTING INFORMATION

Alkynylated H-Phe-Cys-Phe-Lys-Ala-Leu-NH_2 (**14b**) and corresponding VBX (**14c**)

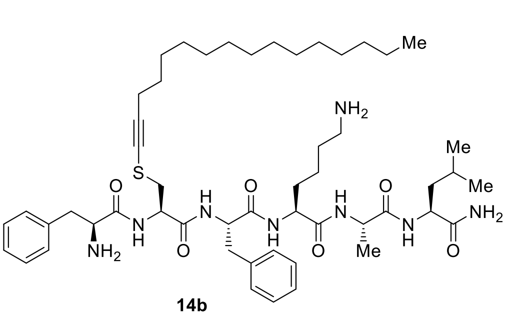

14b

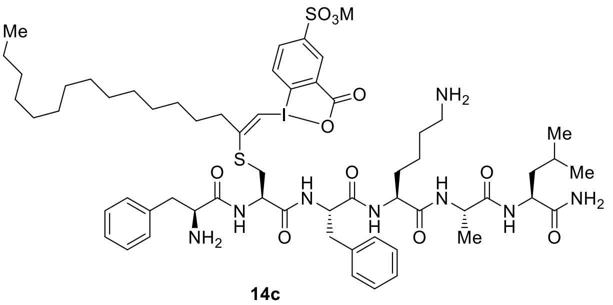

14c

14 (0.50 mg, 0.68 µmol) and **4b** (0.48 mg, 0.82 µmol) in 68 µL 200 mM Tris pH 8.0. Adapted general reaction procedure for **4b**. Yield for **14b** 80% (retention time = 14.2), **14c** 19% (retention time = 13.5).

HRMS (ESI/QTOF) m/z: $[M + H]^+$ Calcd for **14b** $C_{52}H_{83}N_8O_6S^+$ 947.6151; Found 947.6159.

HRMS (ESI/QTOF) m/z: $[M]^-$ Calcd for **14c** $C_{59}H_{86}IN_8O_{11}S_2^-$ 1273.4908; Found 1273.4963.

HPLC gradient: Method 1.

HPLC-UV and HPLC-MS chromatogram

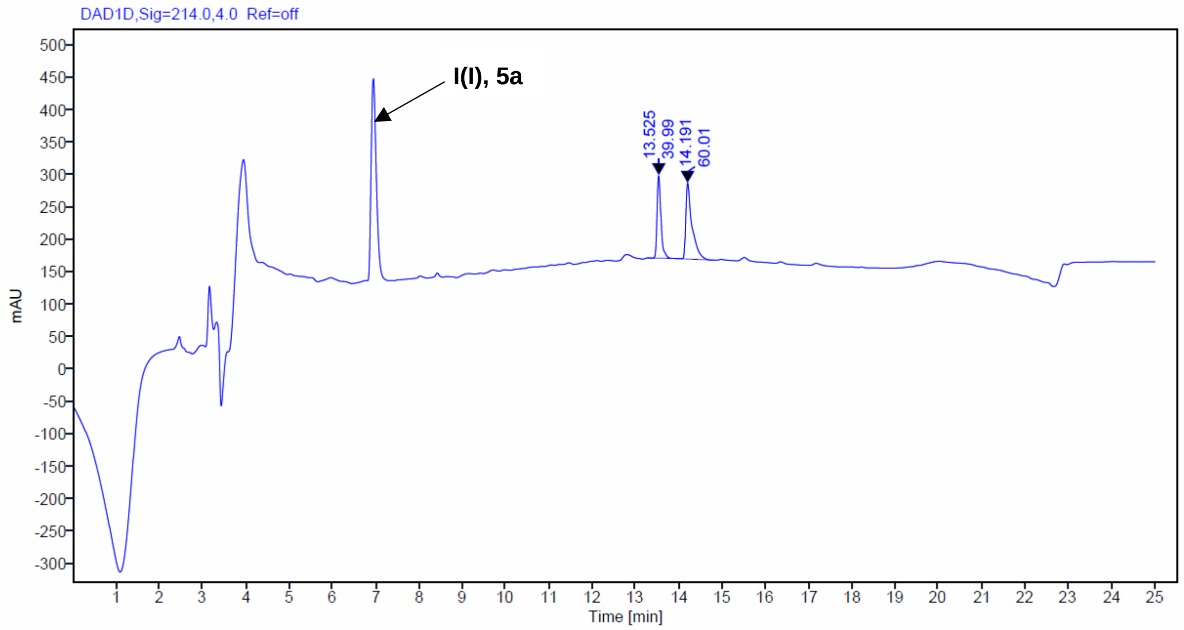

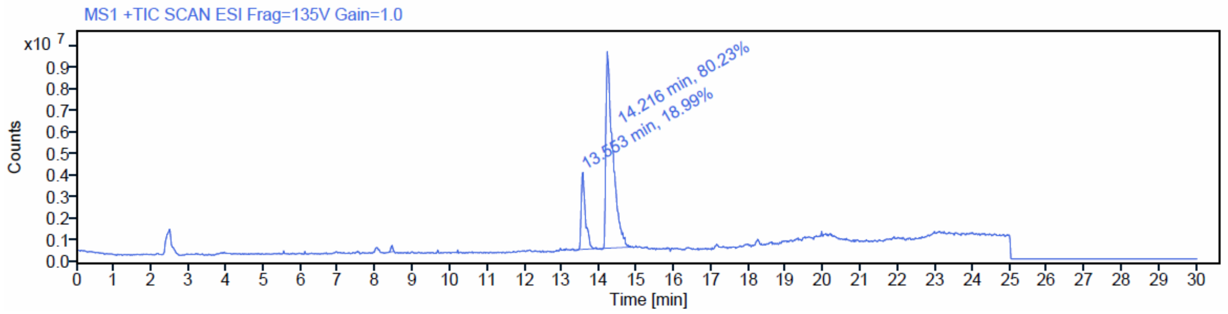

Alkynylated H-Phe-Cys-Gly-Pro-Ser-Leu-NH_2 (**15a**)

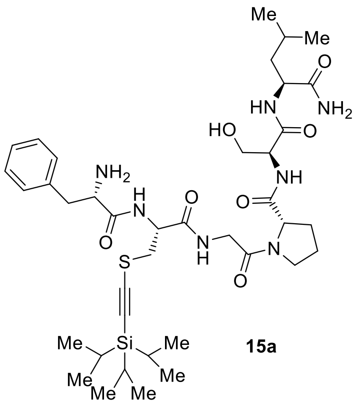

15a

15 (0.50 mg, 0.80 µmol) and **4a** (0.60 mg, 1.2 µmol) in 80 µL 10 mM Tris pH 7.4. Adapted general reaction procedure for **4a**. Yield for **15a** 67% (retention time = 13.3).

HRMS (ESI/QTOF) m/z: $[M + H]^+$ Calcd for **15a** $C_{39}H_{64}N_7O_7SSi^+$ 802.4352; Found 802.4318.

HPLC gradient: Method 1.

HPLC-UV and HPLC-MS chromatogram

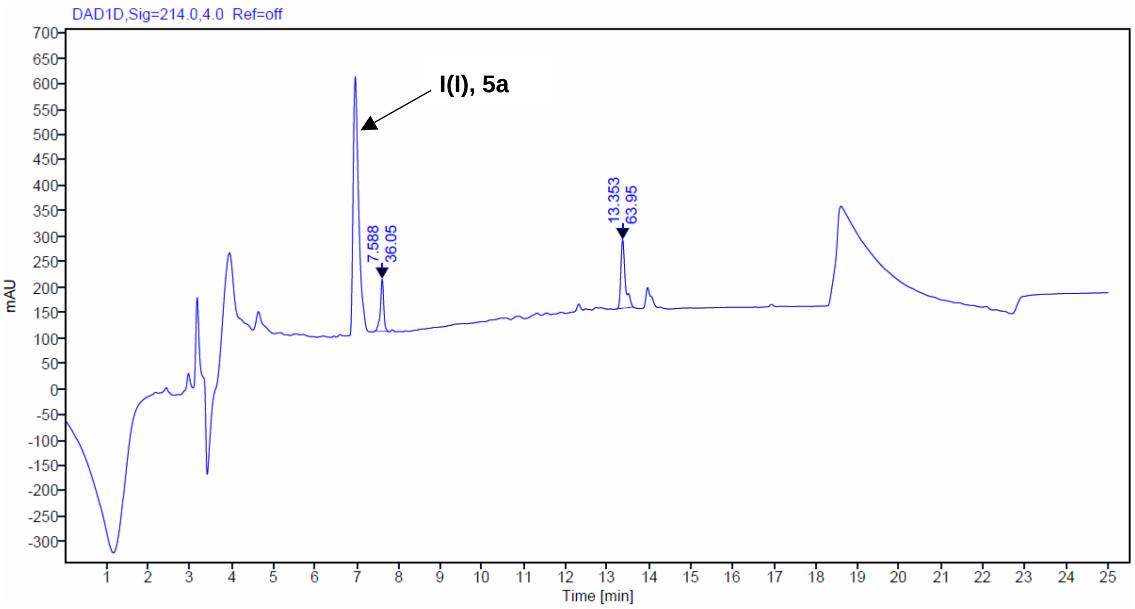

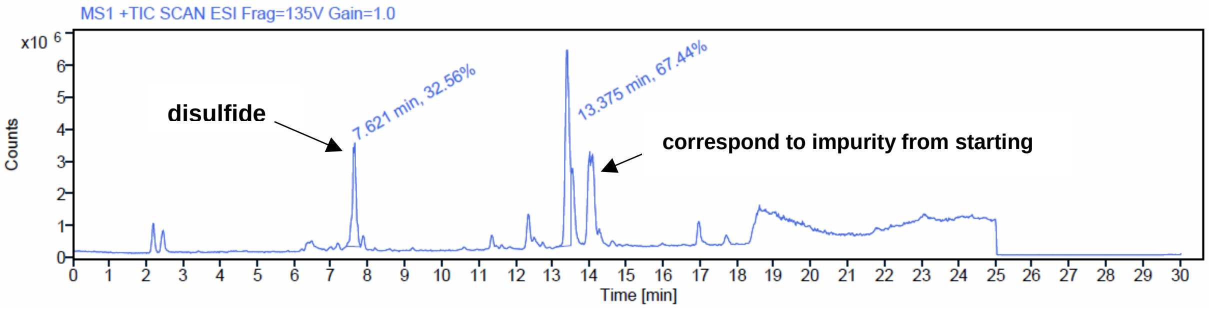

SUPPORTING INFORMATION

Alkynylated H-Phe-Cys-Gly-Pro-Ser-Leu-NH$_2$ (**15b**) and corresponding VBX (**15c**)

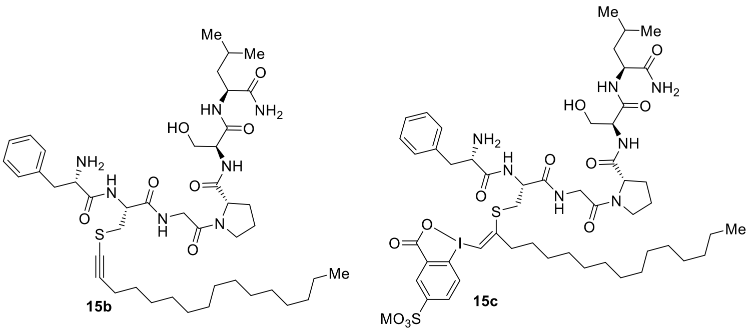

15 (0.50 mg, 0.80 µmol) and **4b** (0.56 mg, 0.82 µmol) in 80 µL 200 mM Tris pH 8.0. Adapted general reaction procedure for **4b.** Yield for **15b** 74% (retention time = 16.7), **15c** 22% (retention time = 15.4).

HRMS (ESI/QTOF) m/z: $[M + H]^+$ Calcd for **15b** $C_{44}H_{72}N_7O_7S^+$ 842.5208; Found 842.5201.

HRMS (ESI/QTOF) m/z: $[M]^-$ Calcd for **15c** $C_{51}H_{75}IN_7O_{12}S_2^-$ 1168.3965; Found 1168.3991.

HPLC gradient: Method 1.

HPLC-UV and HPLC-MS chromatogram

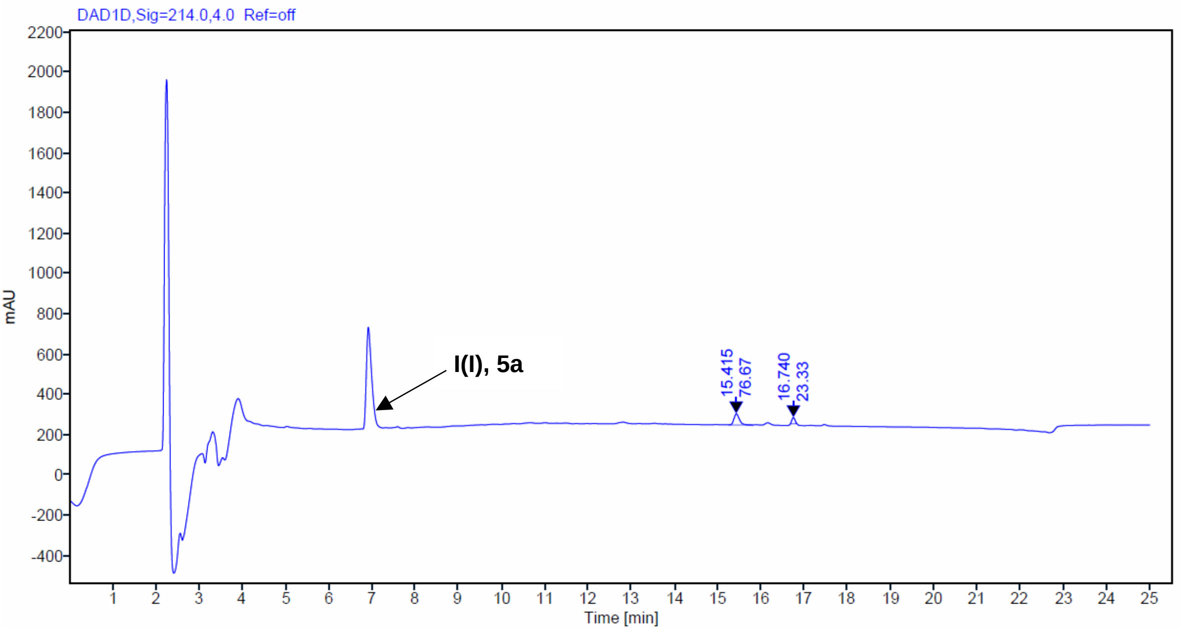

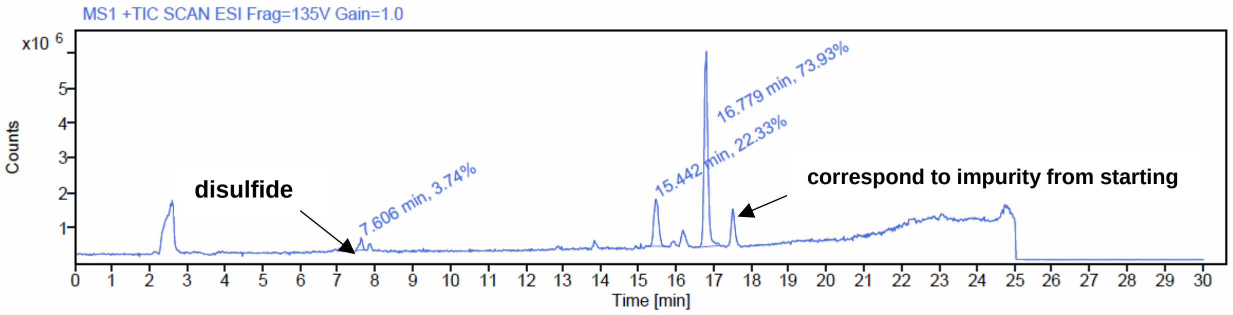

LC-MS/MS of **15b**

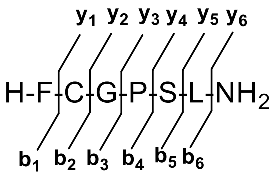

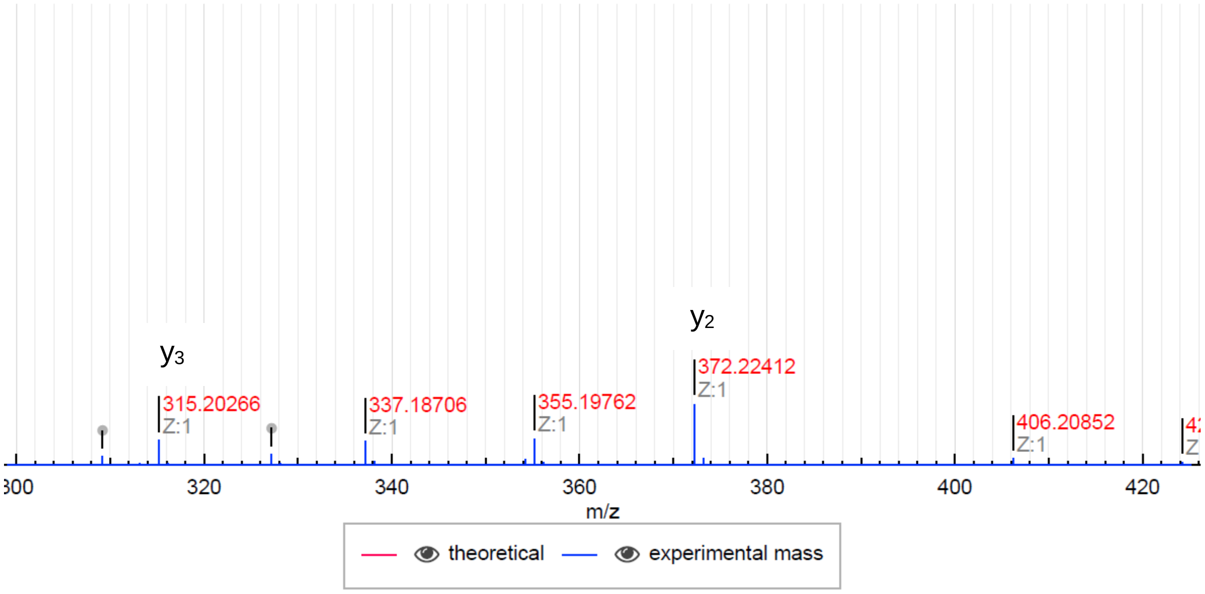

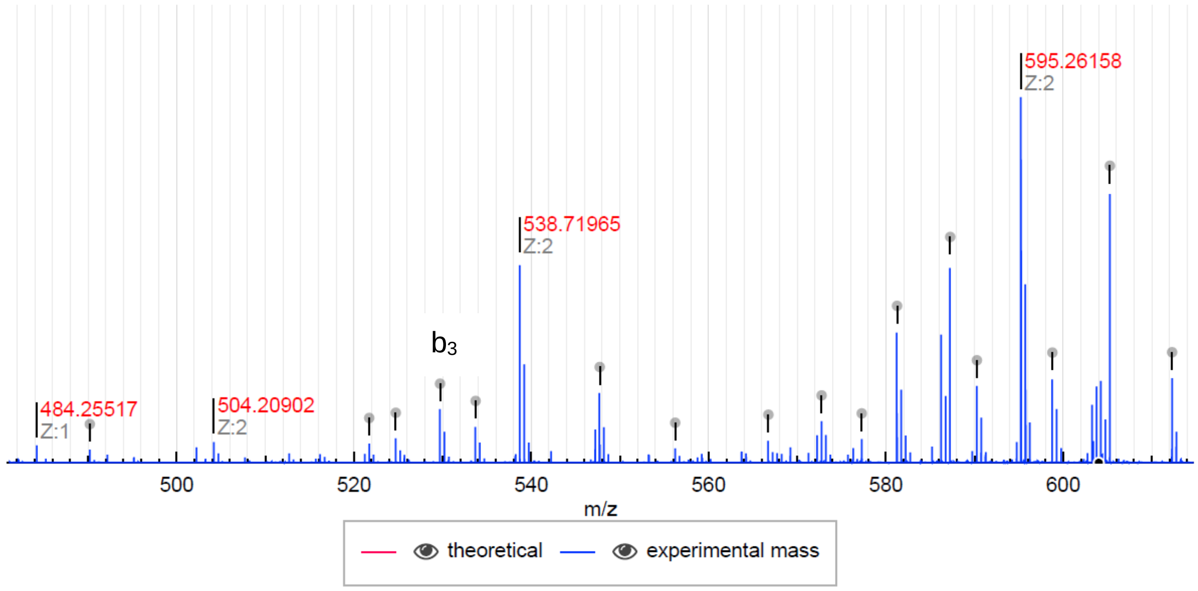

SUPPORTING INFORMATION

Alkynylated H-Gly-Cys-Ala-Leu-Asn-Thr-NH_2 (**16a**)

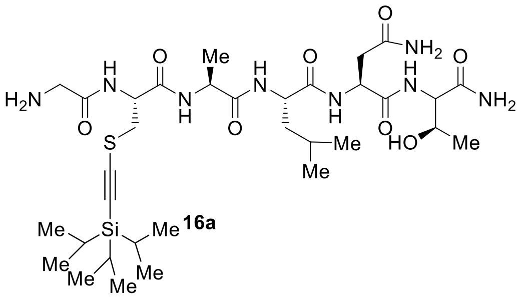

16a

16 (1.0 mg, 1.7 µmol) and **4a** (1.4 mg, 2.6 µmol) in 173 µL 10 mM Tris pH 7.4. Adapted general reaction procedure for **4a**. Yield for **16a** 86% (retention time = 12.1)

HRMS (ESI/QTOF) m/z: $[M + H]^+$ Calcd for **16a** $C_{33}H_{61}N_8O_8SSi^+$ 757.4097; Found 757.4130.

HPLC gradient: Method 1.

HPLC-UV and HPLC-MS chromatogram

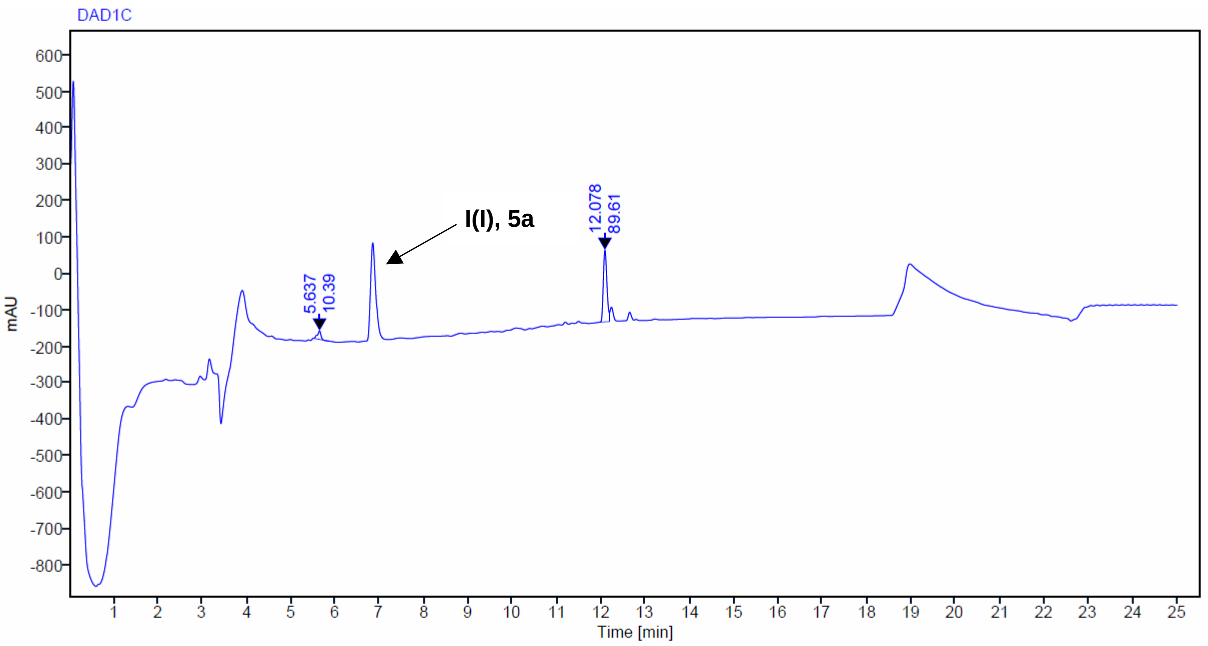

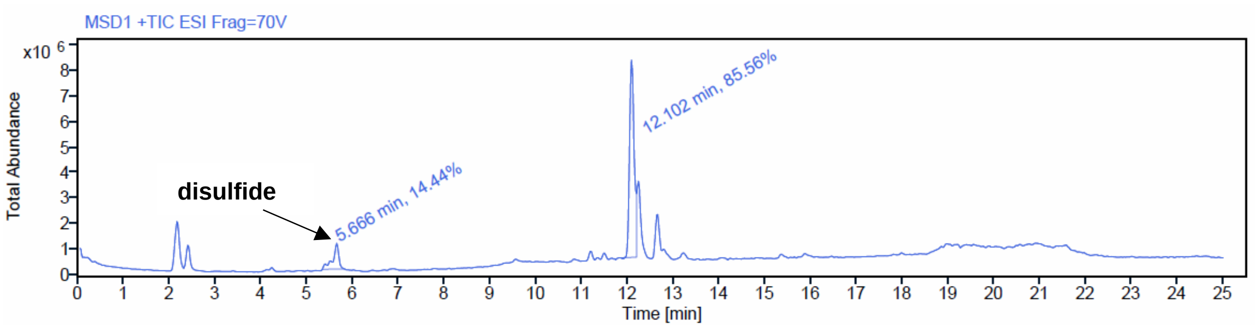

Alkynylated H-Gly-Cys-Ala-Leu-Asn-Thr-NH_2 (**16b**) and corresponding VBX (**16c**)

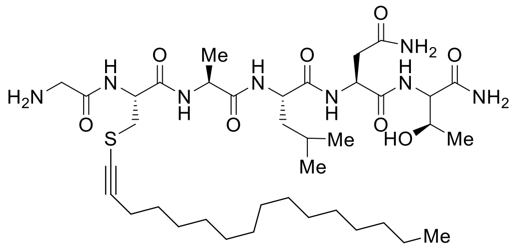

16b

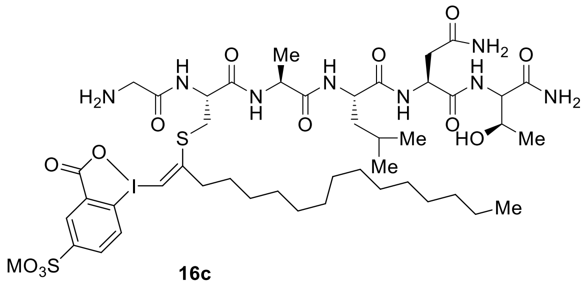

16c

16 (0.50 mg, 0.86 µmol) and **4b** (0.61 mg, 1.0 µmol) in 86 µL 200 mM Tris pH 8.0. Adapted general reaction procedure for **4b.** Yield for **16b** 84% (retention time = 15.3), **16c** 15% (retention time = 14.4).

HRMS (ESI/QTOF) m/z: $[M + H]^+$ Calcd for **16b** $C_{38}H_{69}N_8O_8S^+$ 797.4954; Found 797.4978.

HRMS (ESI/QTOF) m/z: $[M]^-$ Calcd for **16c** $C_{45}H_{72}IN_8O_{13}S_2^-$ 1123.3710; Found 1123.3750.

HPLC gradient: Method 1.

HPLC-UV and HPLC-MS chromatogram

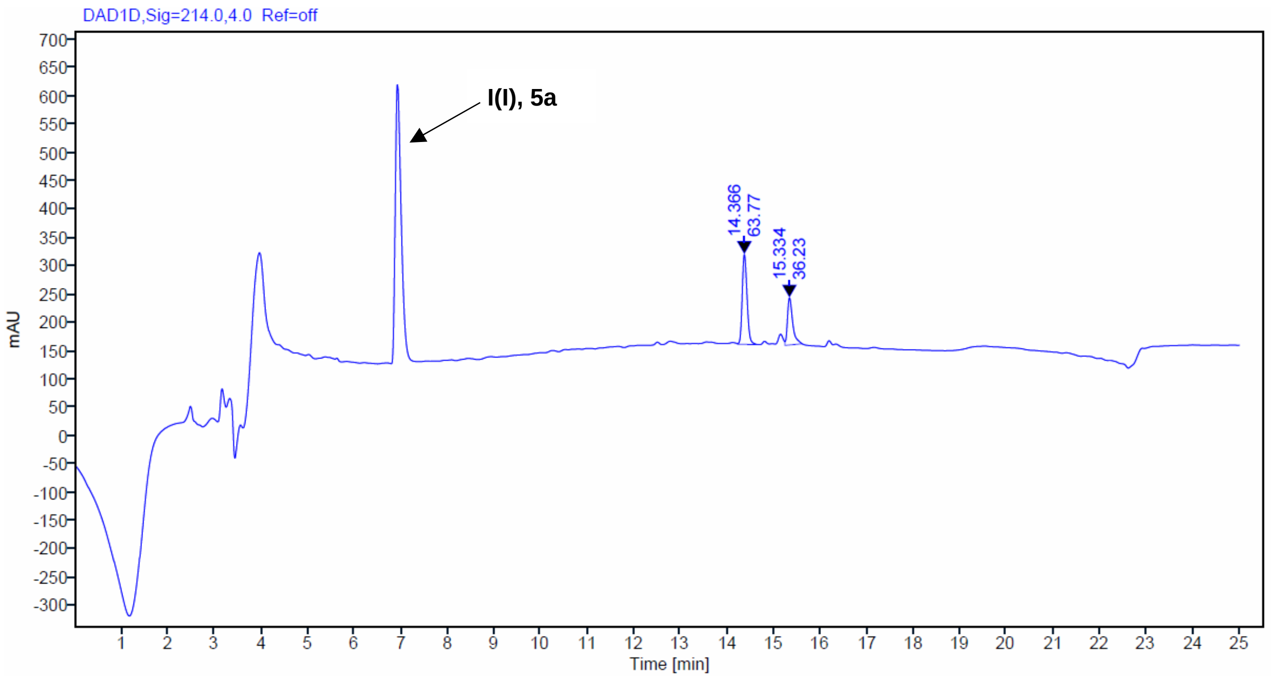

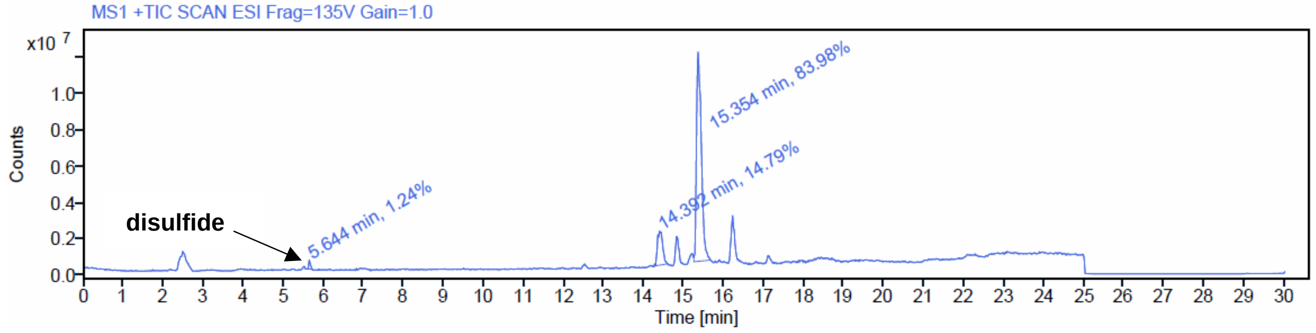

Alkynylated H-Gly-Cys-Ala-Phe-Lys-Thr-NH_2 (**17a**)

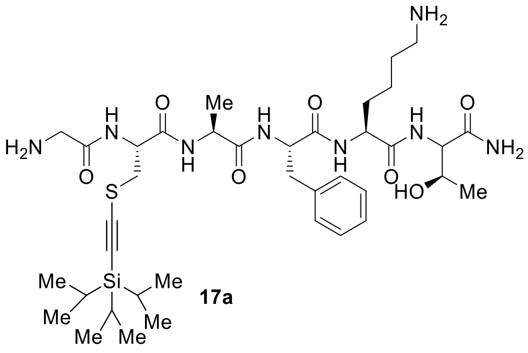

17a

17 (1.0 mg, 1.6 µmol) and **4a** (1.3 mg, 2.4 µmol) in 160 µL 10 mM Tris pH 7.4. Adapted general reaction procedure for **4a**. Yield for **17a** 69% (retention time = 10.7)

HRMS (ESI/QTOF) m/z: $[M+H]^+$ Calcd for **17a** $C_{38}H_{65}N_8O_7SSi^+$ 805.4461; Found 805.4462.

HPLC gradient: Method 1.

HPLC-UV and HPLC-MS chromatogram

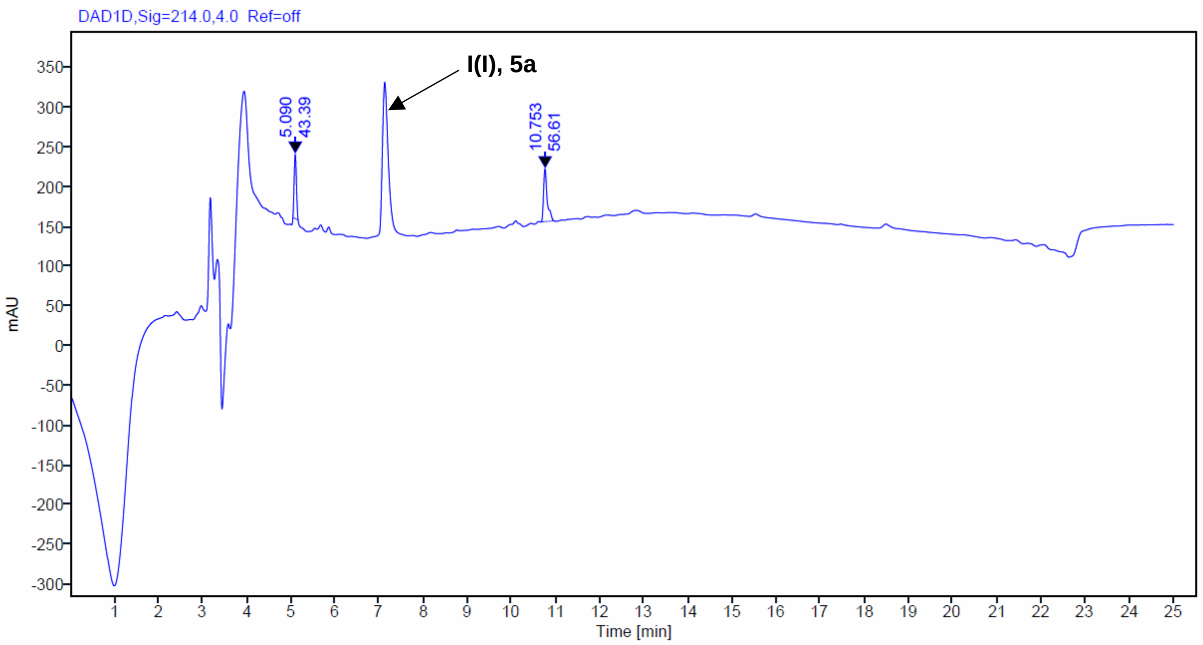

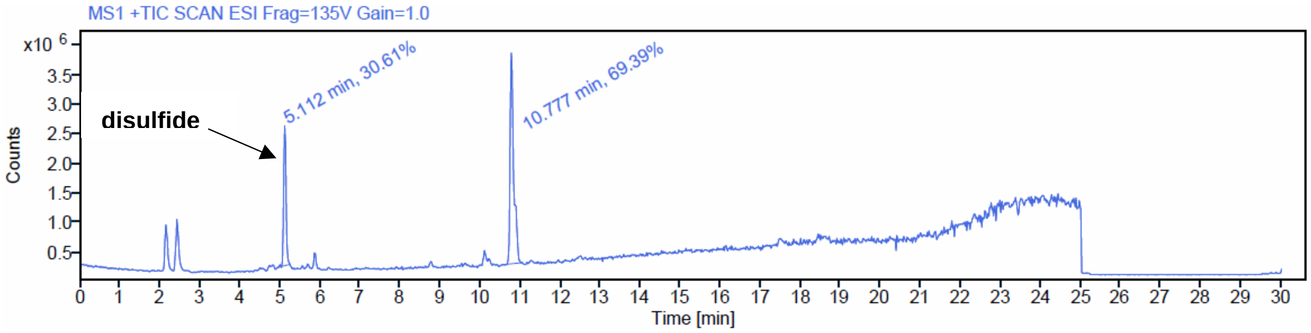

Alkynylated H-Gly-Cys-Ala-Phe-Lys-Thr-NH_2 (**17b**) and corresponding VBX (**17c**)

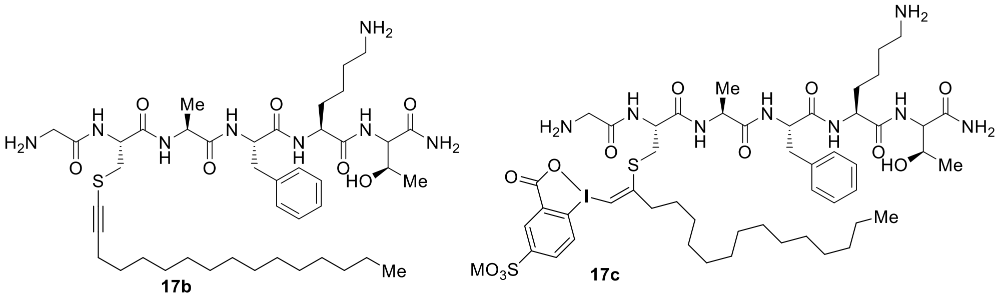

17b

17c

17 (0.50 mg, 0.80 µmol) and **4b** (0.56 mg, 0.96 µmol) in 80 µL 200 mM Tris pH 8.0. Adapted general reaction procedure for **4b.** Yield for **17b** 72% (retention time = 13.1), **17c** 25% (retention time = 12.5).

HRMS (QTOF) m/z: $[M + H]^+$ Calcd for **17b** $C_{43}H_{73}N_8O_7S^+$ 845.5317; Found 845.5336**.**

HRMS (ESI/QTOF) m/z: $[M]^-$ Calcd for **17c** $C_{50}H_{76}IN_8O_{12}S_2^-$ 1171.4074; Found 1171.4124.

HPLC gradient: Method 1.

HPLC-UV and HPLC-MS chromatogram

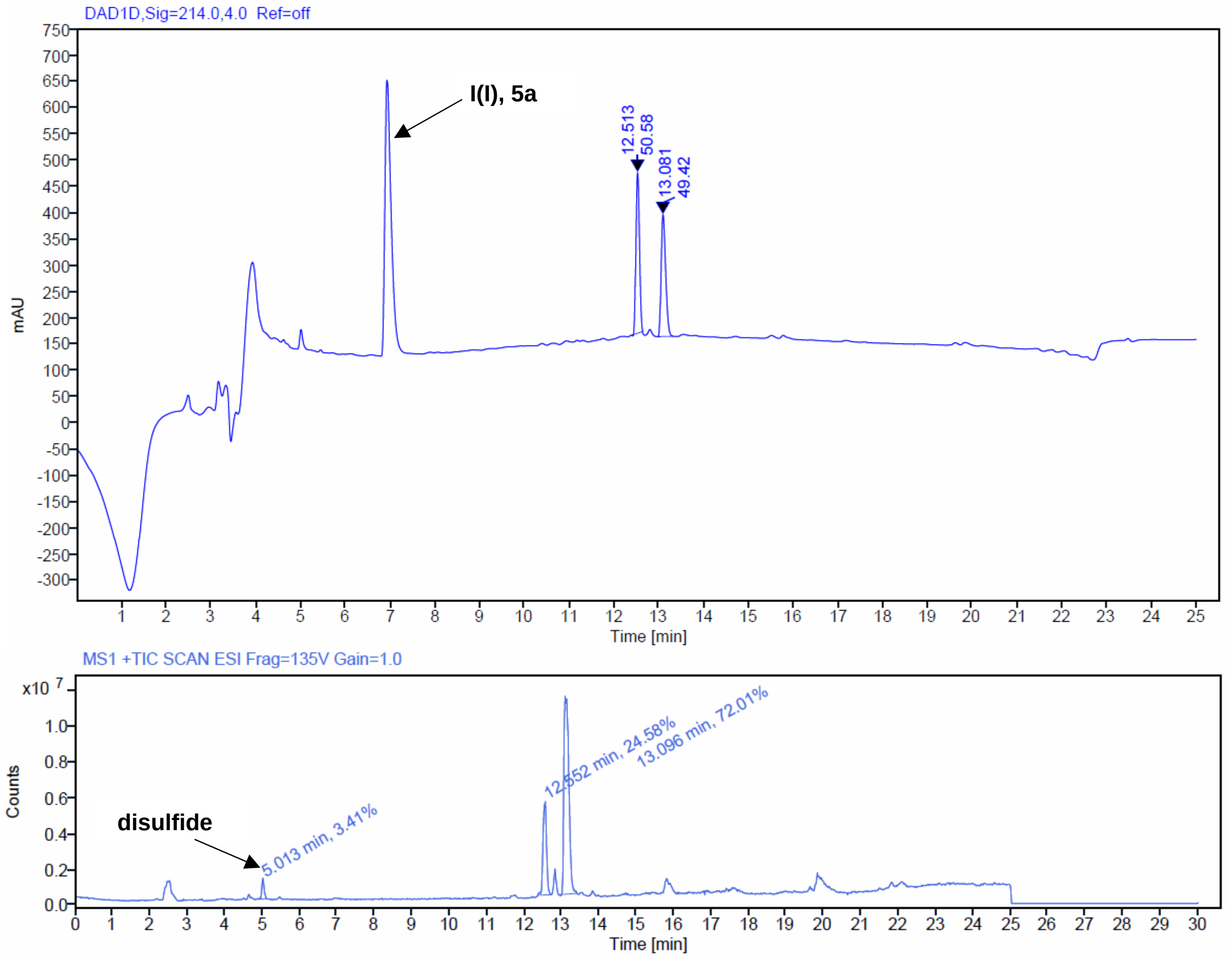

Alkynylated H-Ala-Cys-Ala-Phe-Lys-Asp-NH_2 (**18a**)

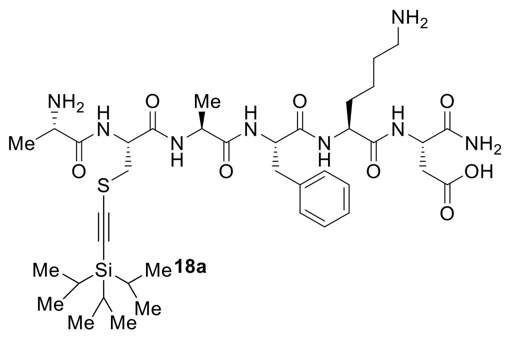

18a

18 (1.0 mg, 1.5 µmol) and **4a** (1.3 mg, 2.3 µmol) in 153 µL 10 mM Tris pH 7.4. Adapted general reaction procedure for **4a**. Yield for **18a** 89% (retention time = 10.9).

HRMS (ESI/QTOF) m/z: $[M + H]^+$ Calcd for **18a** $C_{39}H_{65}N_8O_8SSi^+$ 833.4410; Found 833.4400.

HPLC gradient: Method 1.

HPLC-UV and HPLC-MS chromatogram

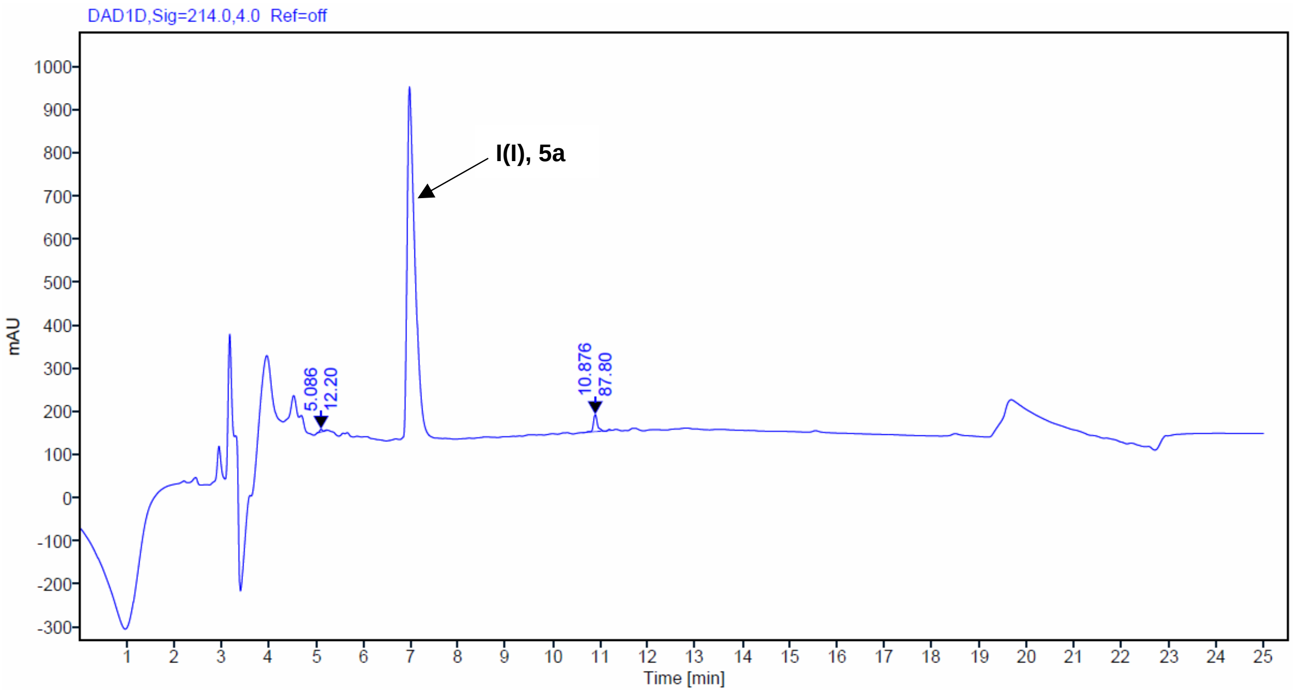

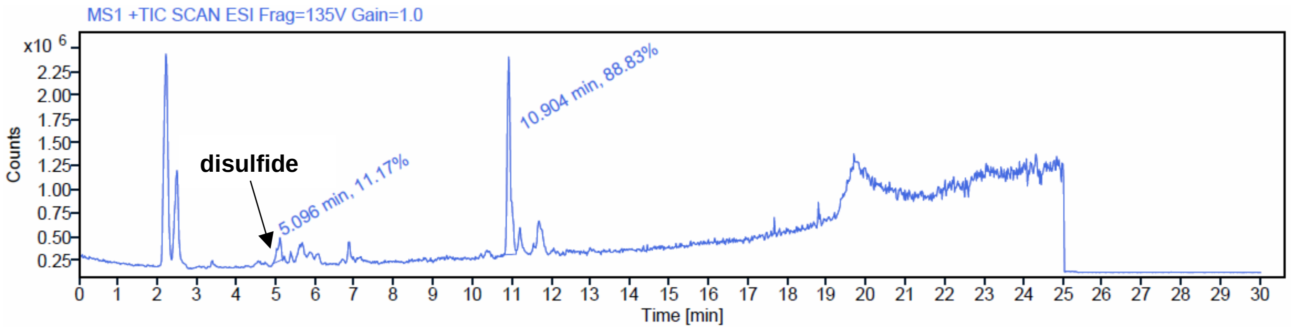

Alkynylated H-Ala-Cys-Ala-Phe-Lys-Asp-NH_2 (**18b**) and corresponding VBX (**18c**)

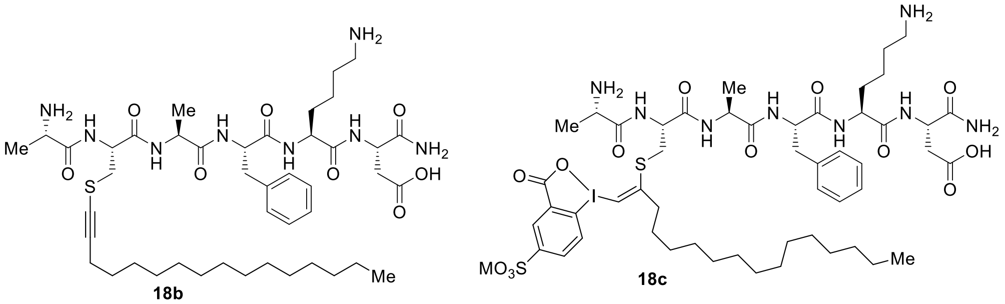

18b

18c

18 (0.50 mg, 0.76 µmol) and **4b** (0.54 mg, 0.91 µmol) in 77 µL 200 mM Tris pH 8.0. Adapted general reaction procedure for **4b**. Yield for **18b** 62% (retention time = 13.2), **18c** 28% (retention time = 12.3).

HRMS (ESI/QTOF) m/z: $[M + H]^+$ Calcd for **18b** $C_{44}H_{73}N_8O_8S^+$ 873.5267; Found 873.5278.

HRMS (ESI/QTOF) m/z: $[M]^-$ Calcd for **18c** $C_{51}H_{76}IN_8O_{13}S_2^-$ 1199.4023; Found 1199.4075.

HPLC gradient: Method 1.

HPLC-UV and HPLC-MS chromatogram

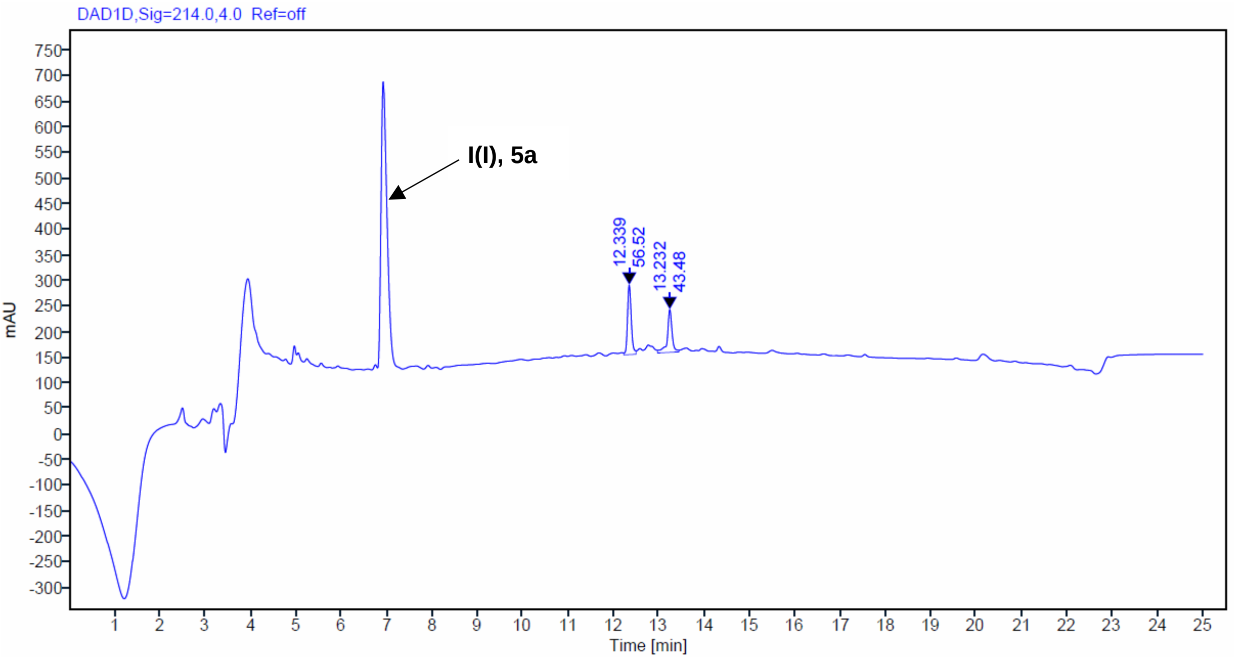

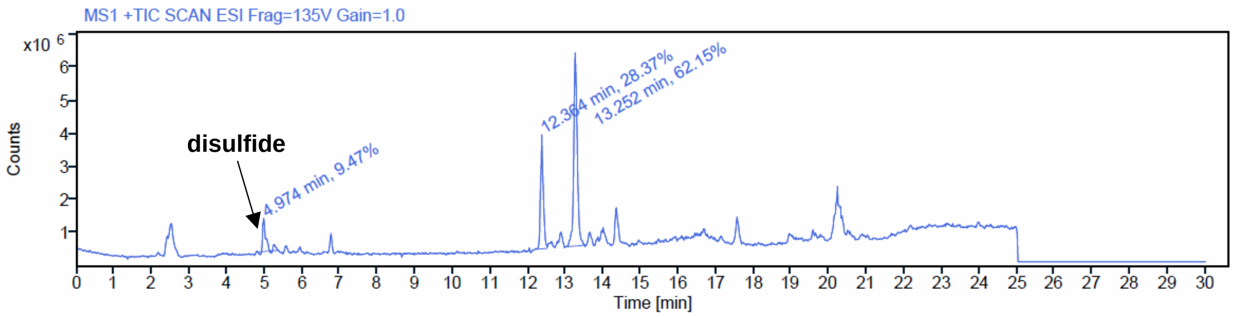

8. Substrate scope for long peptides

Alkynylated Ac-Met-Val-Arg-Gln-Val-His-Lys-Asp-Leu-Ile-Cys-Glu-Pro-Asn-Glu-NH_2 (**19a**)

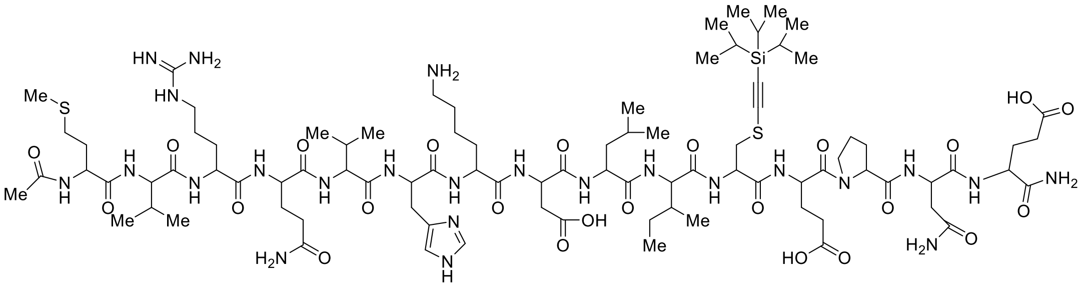

19a

19 (1.0 mg, 0.54 µmol) and **4a** (0.44 mg, 0.80 µmol) in 55 µL 10 mM Tris pH 7.4. Adapted general reaction procedure for **4a.** Yield for **19a** 76% (retention time = 10.5).

HRMS (ESI/QTOF) m/z: $[M + H_3]^{+3}$ Calcd for $C_{89}H_{153}N_{24}O_{24}S_2Si^{+3}$ 678.0228; Found 678.0254.

HPLC gradient: Method 1.

HPLC-UV and HPLC-MS chromatogram

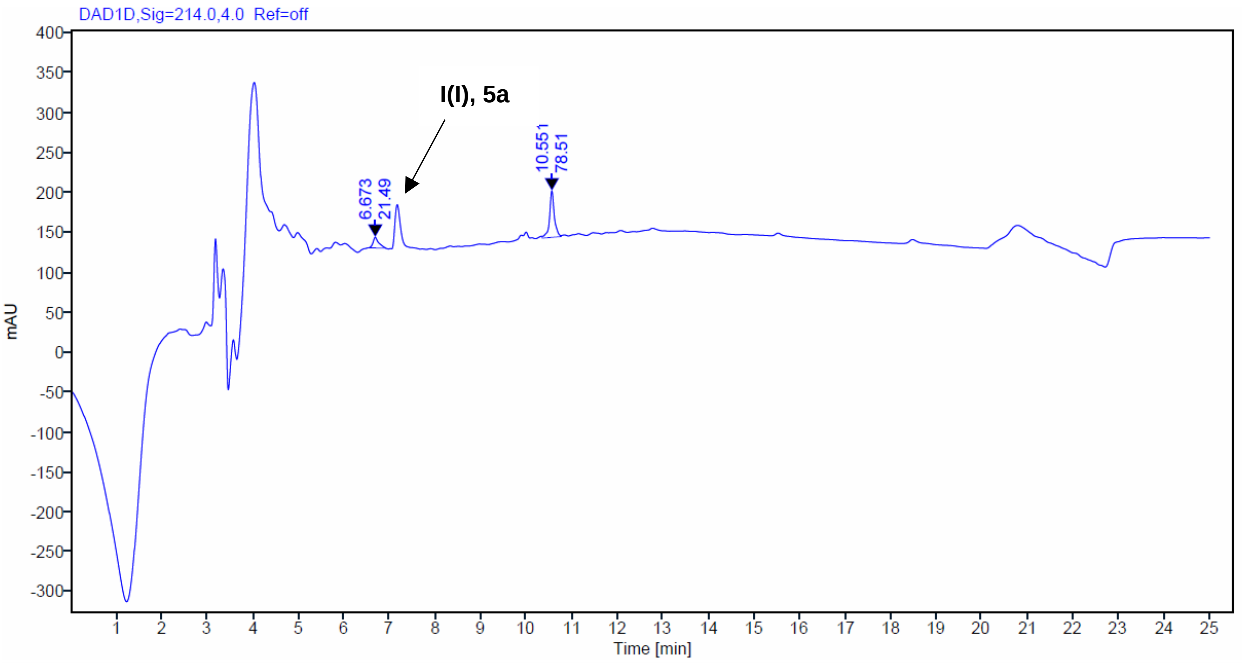

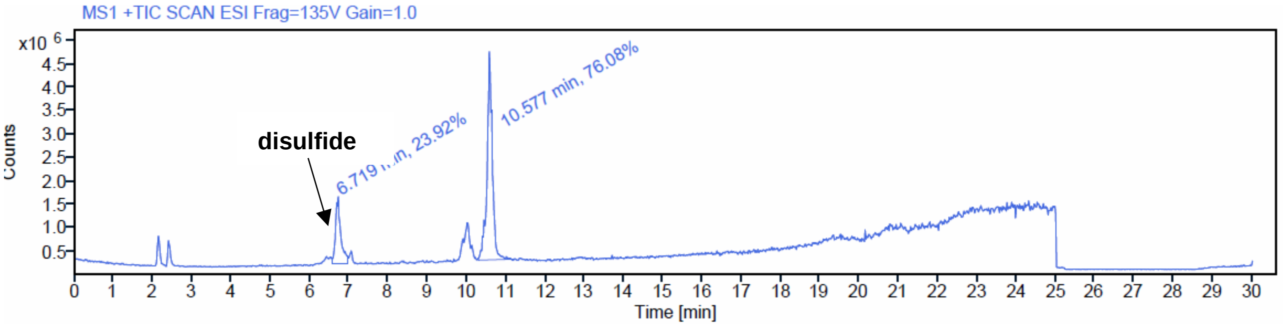

Alkynylated Ac-Met-Val-Arg-Gln-Val-His-Lys-Asp-Leu-Ile-Cys-Glu-Pro-Asn-Glu-NH_2 (**19b**) and corresponding VBX (**19c**)

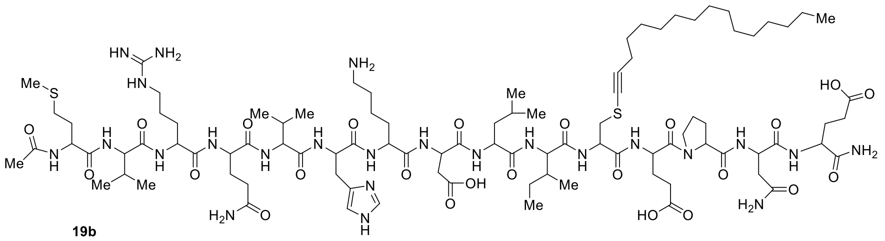

19b

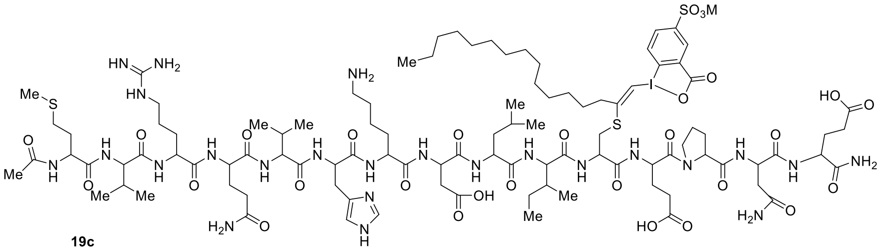

19c

19 (1.0 mg, 0.54 µmol) and **4b** (0.38 mg, 0.64 µmol) in 77 µL 100 mM PB pH 8.0. Adapted general reaction procedure for **4b**. Yield for **19b** 50% (retention time = 12.4), **19c** 15% (retention time = 11.6).

HRMS (ESI/QTOF) m/z: $[M + 2H]^{+2}$ Calcd for **19b** $C_{94}H_{160}N_{24}O_{24}S_2^{+2}$ 1036.5734; Found 1036.5774.

HRMS (ESI/QTOF) m/z: $[M + 2H]^{+2}$ Calcd for **19c** $C_{101}H_{165}IN_{24}O_{29}S_3^{+2}$ 1200.5190; Found 1200.5153.

HPLC gradient: Method 1.

HPLC-UV and HPLC-MS chromatogram

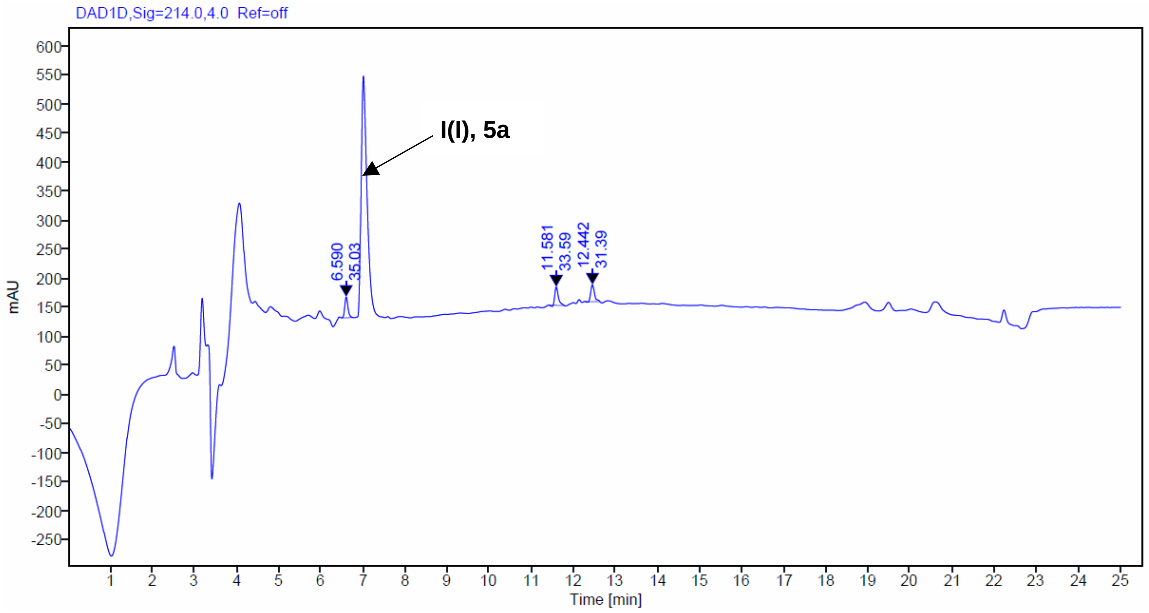

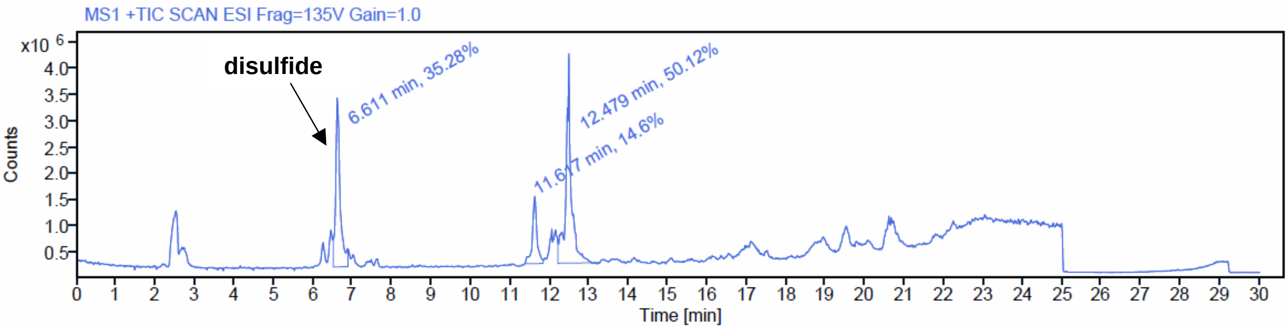

SUPPORTING INFORMATION

Alkynylated Ac-Glu-Arg-Ala-Ala-Lys-Glu-Arg-Ala-Cys-Ala-Glu-Arg-Ala-Ala-Glu-Gly-Gly-Tyr-NH_2 (**20a**)

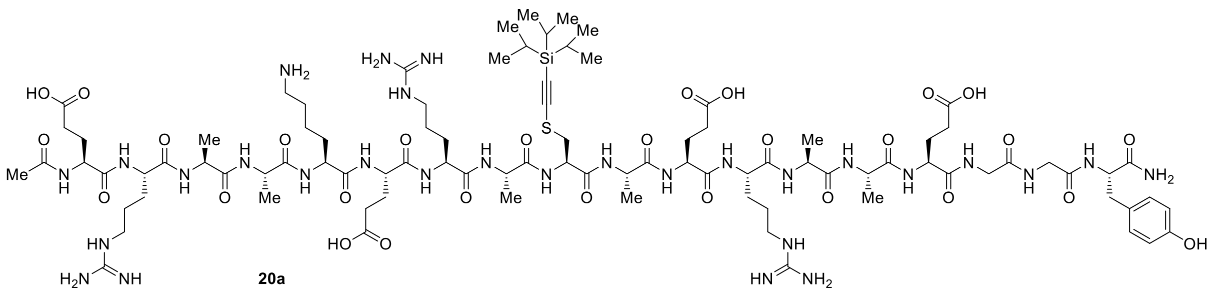

20a

20 (1.0 mg, 0.50 µmol) and **4a** (0.41 mg, 0.75 µmol) in 50 µL 10 mM Tris pH 7.4. Adapted general reaction procedure for **4a**. Yield for **20a** 81% (retention time = 8.1).

HRMS (ESI/QTOF) m/z: $[M + 3H]^{+3}$ Calcd for $C_{91}H_{154}N_{29}O_{28}SSi^{+3}$ 720.3664; Found 720.3661.

HPLC gradient: Method 1.

HPLC-UV and HPLC-MS chromatogram

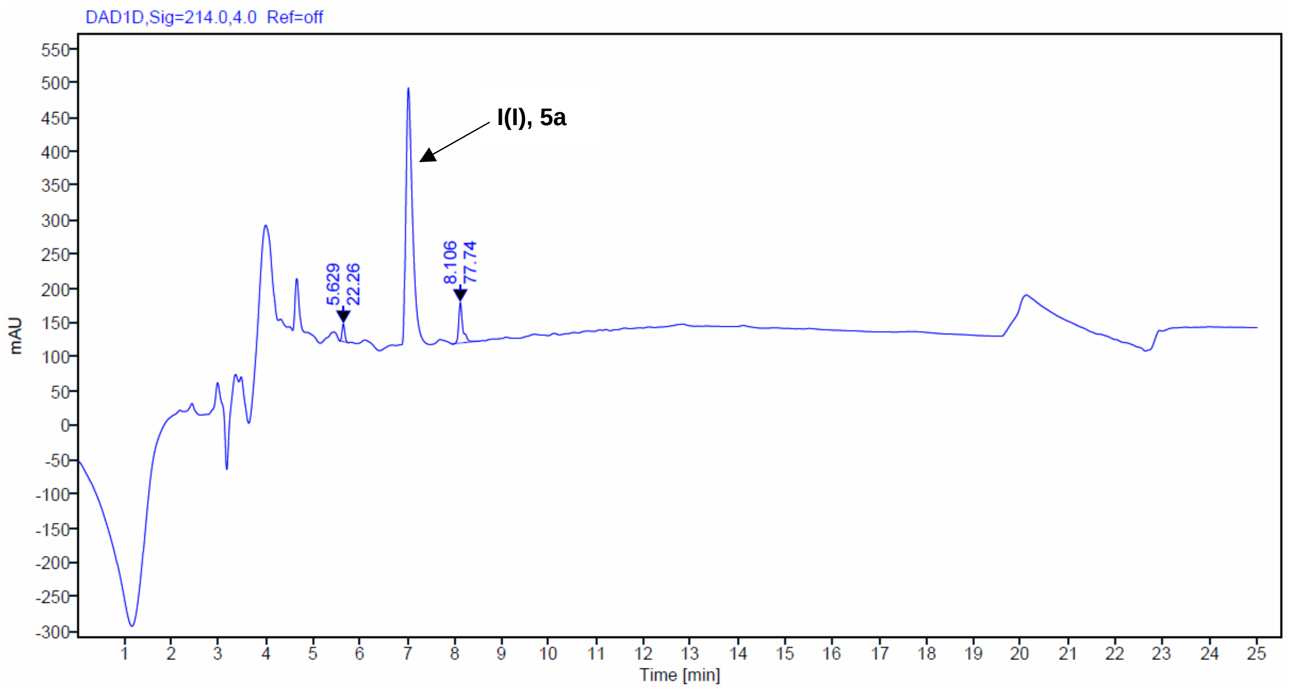

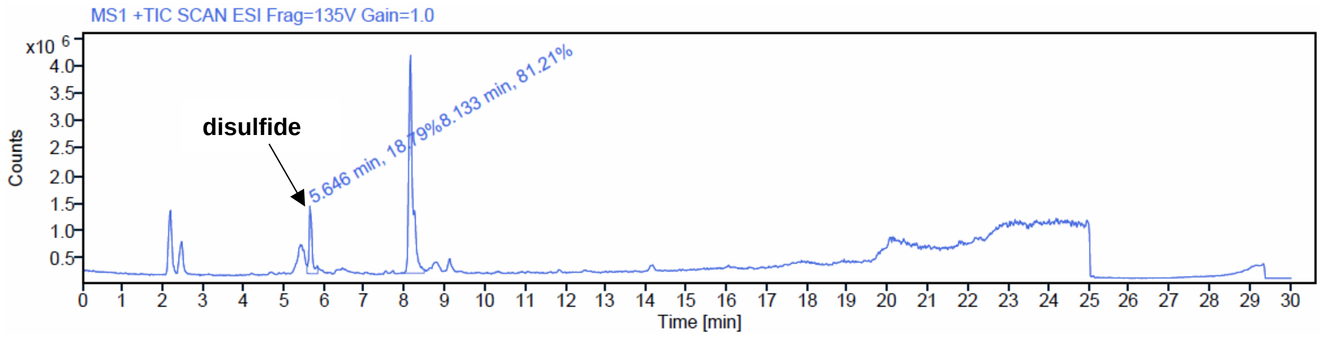

Alkynylated Ac-Glu-Arg-Ala-Ala-Lys-Glu-Arg-Ala-Cys-Ala-Glu-Arg-Ala-Ala-Glu-Gly-Gly-Tyr-NH_2 (**20b**) and corresponding VBX (**20c**)

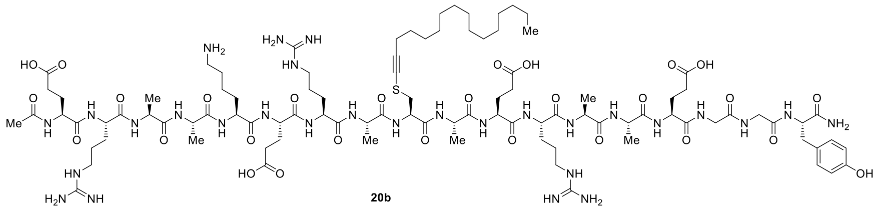

20b

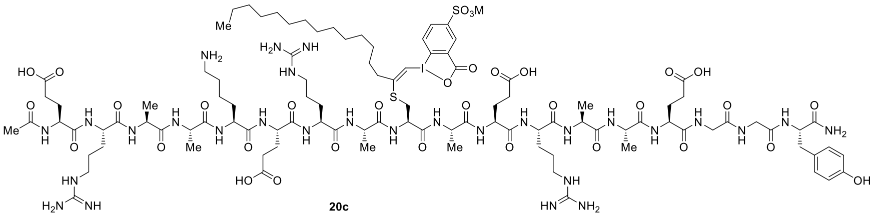

20c

20 (1.0 mg, 0.50 µmol) and **4b** (0.60 mg, 0.35 µmol) in 51 µL 200 mM Tris pH 8.0. Adapted general reaction procedure for **4b.** Yield for **20b** 47% retention time = 10.2), **20c** 28% (retention time = 9.8).

HRMS (ESI/QTOF) m/z: $[M + 2H]^{+2}$ Calcd for **20b** $C_{96}H_{161}N_{29}O_{28}S^{+2}$ 1100.0888; Found 1100.0937.

HRMS (ESI/QTOF) m/z: $[M]^{-2}$ Calcd for **20c** $C_{103}H_{162}IN_{29}O_{33}S_2^{-2}$ 1262.0193; Found 1262.0238.

HPLC gradient: Method 1.

HPLC-UV and HPLC-MS chromatogram

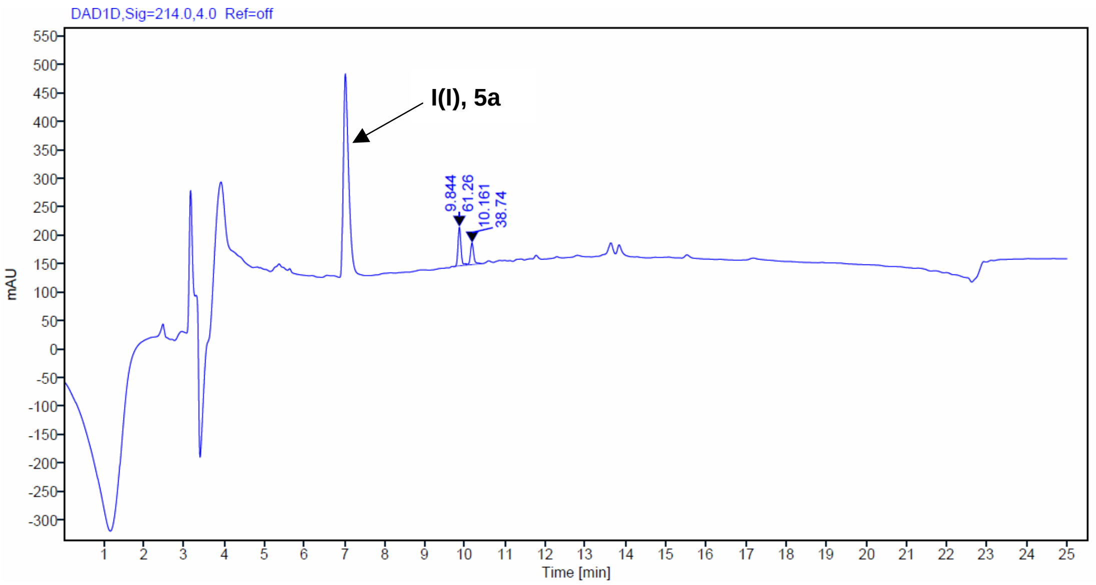

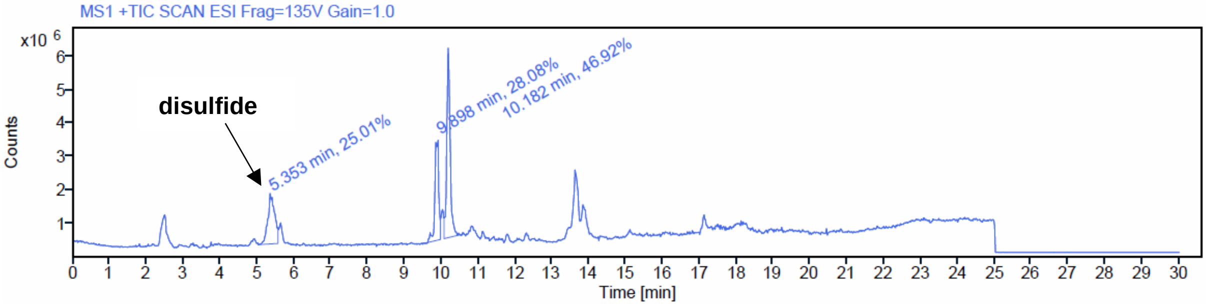

Alkynylated Ac-Asn-Gln-Lys-Leu-Leu-Arg-Trp-Leu-Asn-Cys-Phe-Thr-Gln-Gln-Ser-Gln-NH_2 (**21a**)

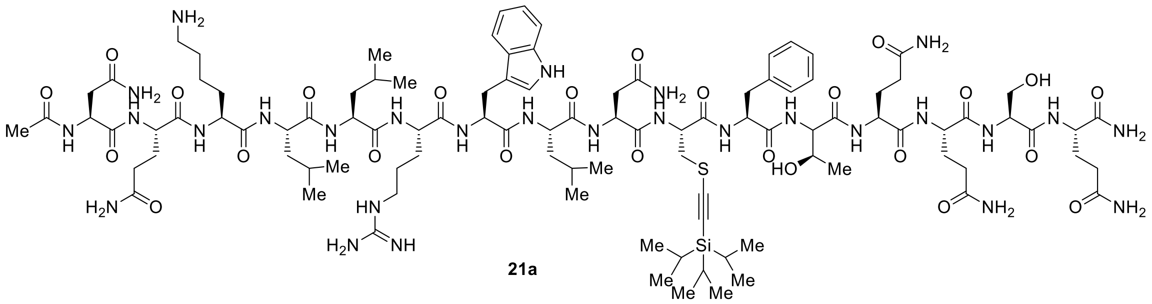

21a

21 (10.0 mg, 4.8 µmol) and **4a** (3.4 mg, 7.3 µmol) in 500 µL 10 mM Tris pH 7.4. Adapted general reaction procedure for **4a**. Yield for **21a** 95% (retention time = 11.6). Isolated yield 51% (5.5 mg, 2.5 µmol, retention time = 14-16 min).

HRMS (ESI/QTOF) m/z: $[M + 3H]^{+3}$ Calcd for **21a** $C_{101}H_{165}N_{28}O_{25}SSi^{+3}$ 743.3991; Found 743.3979.

HPLC gradient: Method 1. **Prep HPLC gradient**: Method 7.

HPLC-UV and HPLC-MS chromatogram

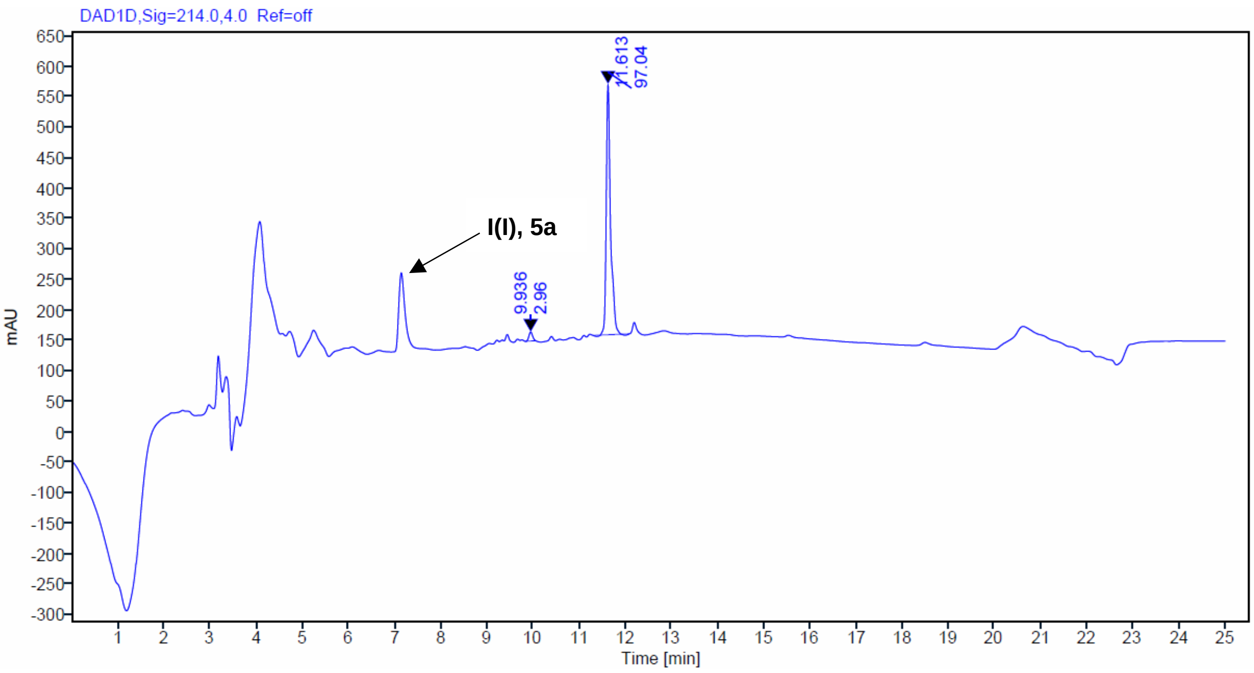

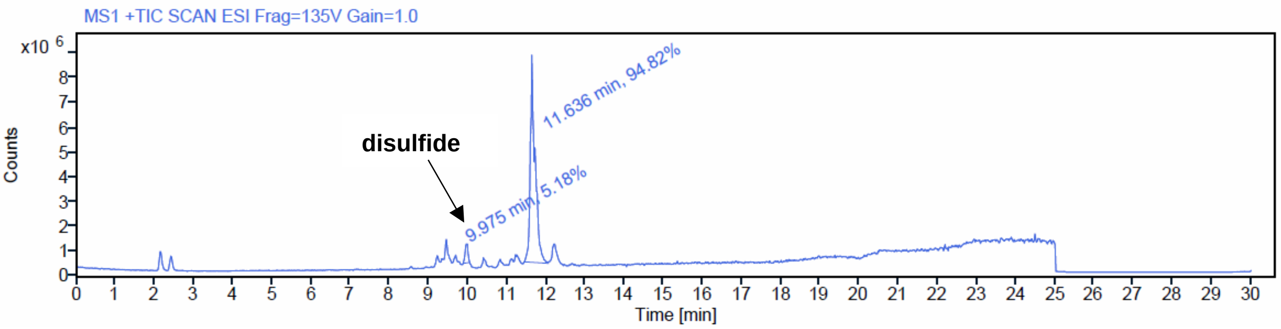

SUPPORTING INFORMATION

Calibration curve of **21a**

Preparation of stock solution. Pure **21a** (0.6 mg) dissolved in 400 uL of MeOH. Stock solution was diluted separately and submitted for RP-HPLC. Calibrated yield 77% (based on HPLC-UV), 38% (based on HPLC-MS).

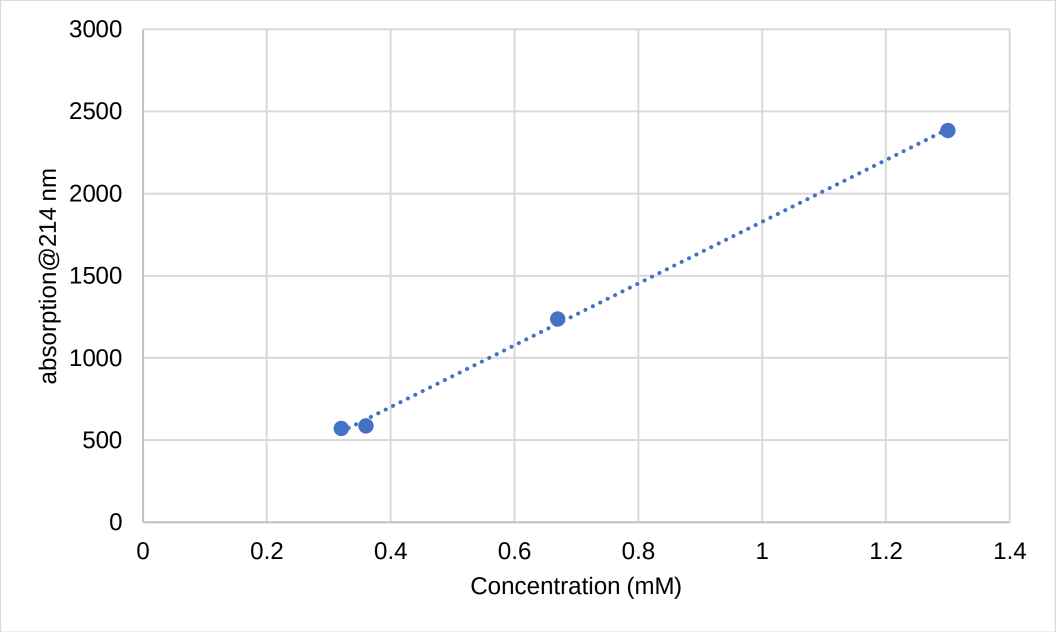

Figure S9. Calibration curve of **21a** based on HPLC-UV at 214nm.

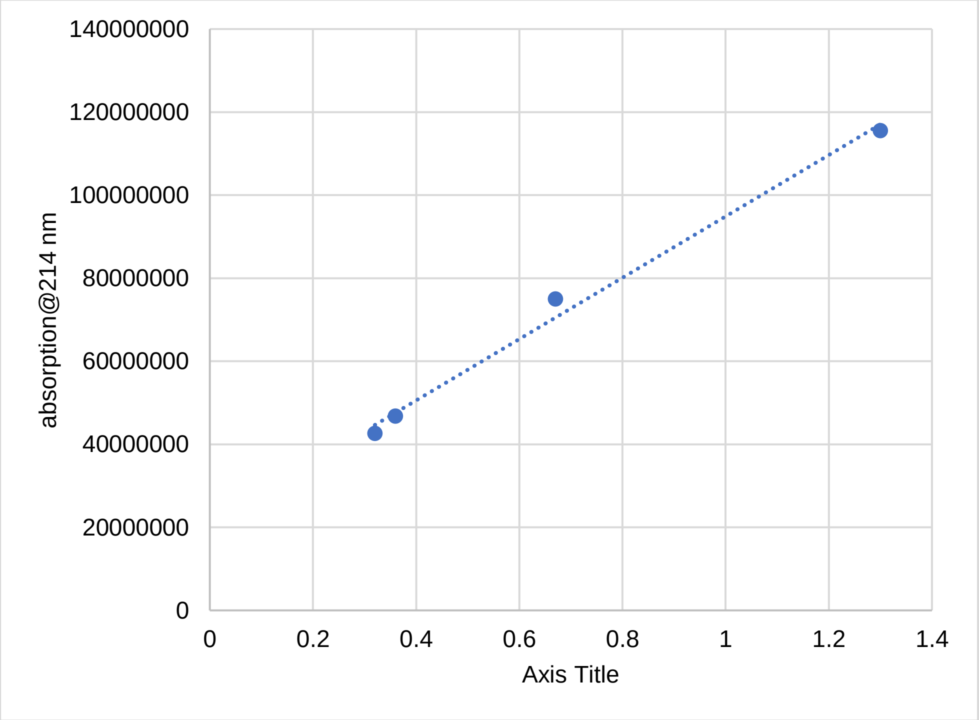

Figure S10. Calibration curve of **21a** based on HPLC-MS.

SUPPORTING INFORMATION

Alkynylated Ac-Asn-Gln-Lys-Leu-Leu-Arg-Trp-Leu-Asn-Cys-Phe-Thr-Gln-Gln-Ser-Gln-NH_2 (**21b**) and corresponding VBX (**21c**)

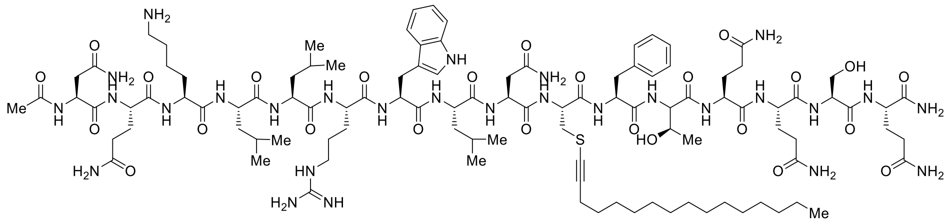

21b

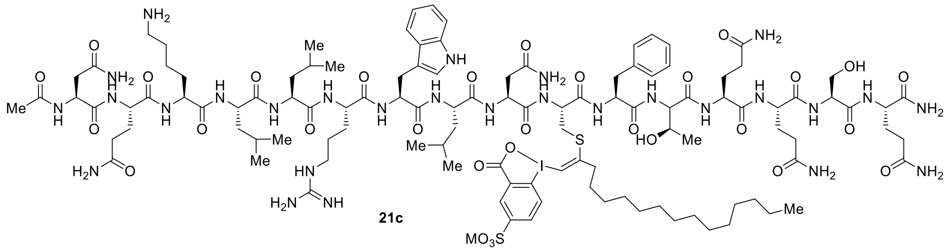

21c

21 (10.0 mg, 4.8 µmol) and **4b** (3.4 mg, 7.3 µmol) in 0.5 mL 200 mM Tris pH 8.0. Adapted general reaction procedure for **4b**. Yield for **21b** 69% (retention time = 13.6), **21c** 29% (retention time = 13.9). Isolated yield 51% (5.5 mg, retention time = 12-14 min.)

HRMS (QTOF) m/z: $[M + H_2]^{+2}$ Calcd for $C_{106}H_{172}N_{28}O_{25}S^{+2}$ 1134.6379; Found 1134.6402.

HPLC gradient: Method 1. **Pep-HPLC gradient**: Method 7.

HPLC-UV and HPLC-MS chromatogram

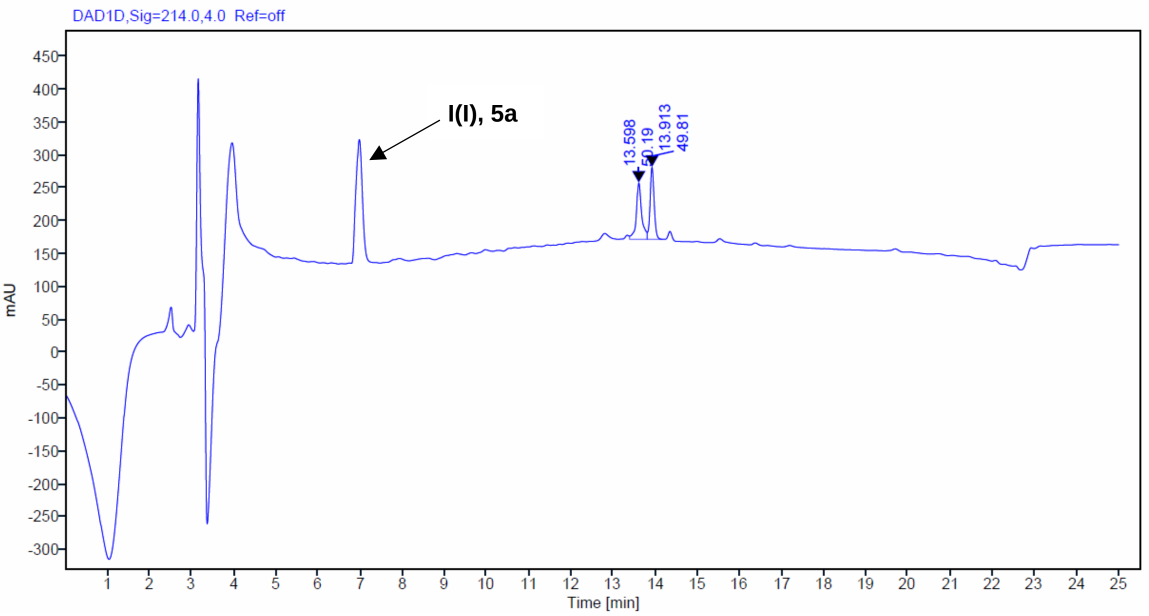

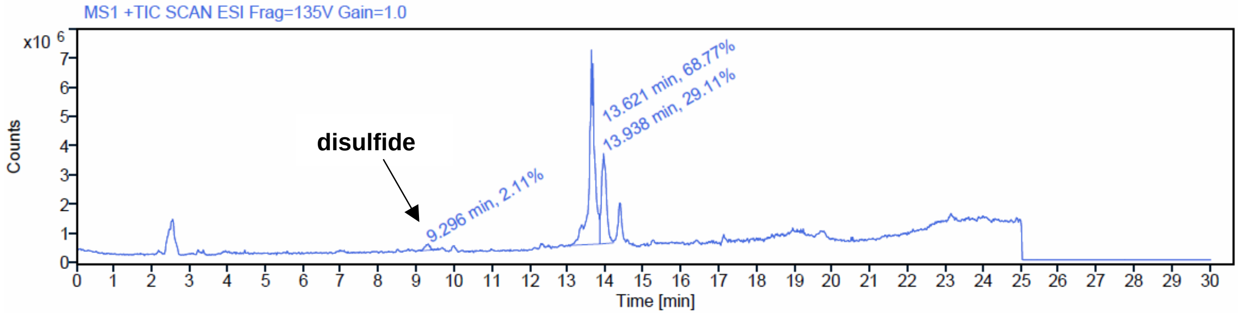

9. Substrate scope for bio-active fragments

Alkynylated Ac-Leu-Gln-Gln-Cys-Pro-Phe-Glu-Asp-His-NH_2 (**22a**)

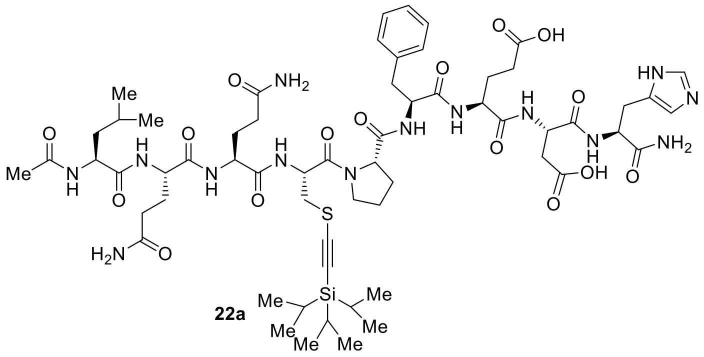

22a

22 (1.0 mg, 0.86 µmol) and **4a** (0.71 mg, 0.73 µmol) in 86 µL 10 mM Tris pH 7.4. Adapted general reaction procedure for **4a**. Yield for **22a** 78% (retention time = 12.5).

HRMS (ESI/QTOF) m/z: $[M + 2H]^{+2}$ Calcd for $C_{61}H_{94}N_{14}O_{16}SSi^{+2}$ 669.3226; Found 669.3252.

HPLC gradient: Method 1.

HPLC-UV and HPLC-MS chromatogram

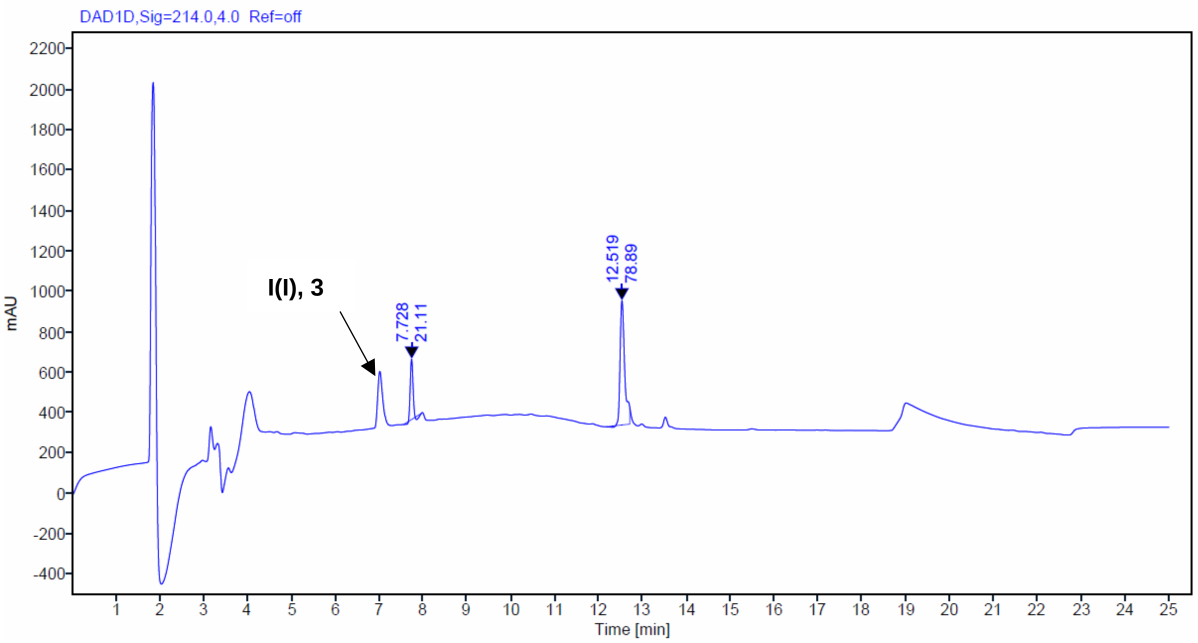

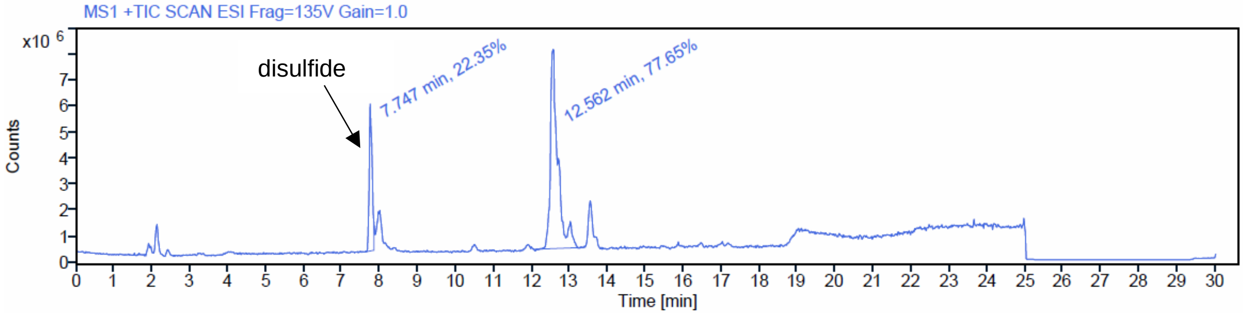

Alkynylated Ac-Leu-Gln-Gln-Cys-Pro-Phe-Glu-Asp-His-NH_2 (**22b**) and corresponding VBX (**22c**)

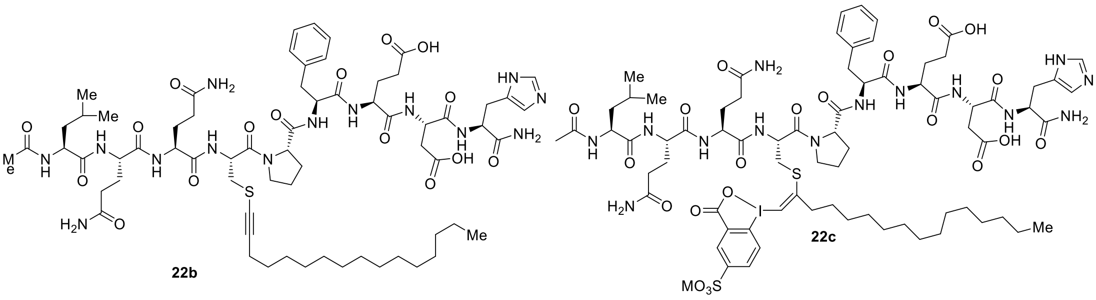

22 (1.0 mg, 0.86 µmol) and **4b** (0.61 mg, 1.0 µmol) in 86 µL 200 mM Tris pH 8.0. Adapted general reaction procedure for **4b**. Yield for **22b** 52% (retention time = 15.8), **22c** 43% (retention time = 14.1).

HRMS (ESI/QTOF) m/z: $[M + H]^+$ Calcd for **22b** $C_{66}H_{101}N_{14}O_{16}S^+$ 1377.7235; Found 1377.7200.

HRMS (ESI/QTOF) m/z: $[M]^-$ Calcd for **22c** $C_{73}H_{104}IN_{14}O_{21}S_2^-$ 1703.5992; Found 1703.5969.

HPLC gradient: Method 1.

HPLC-UV and HPLC-MS chromatogram

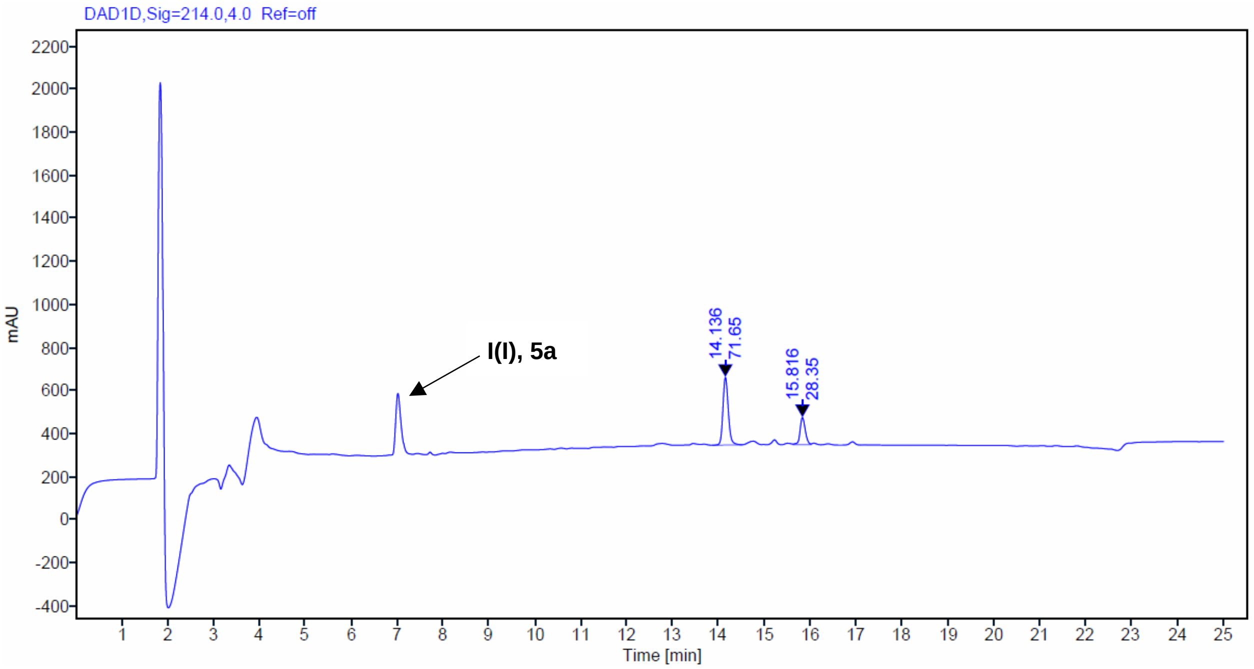

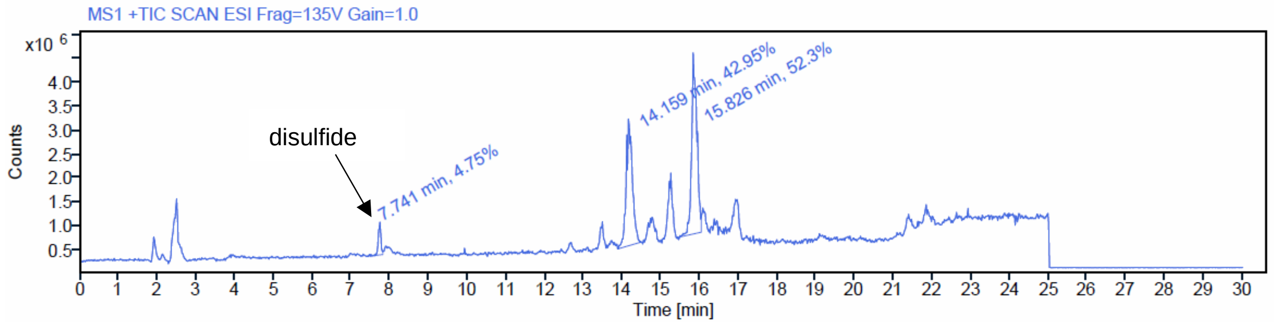

Alkynylated Ac-Trp-Met-Asn-Ser-Thr-Gly-Phe-Thr-Lys-Val-Cys-Gly-Ala-NH_2 **(23a)**

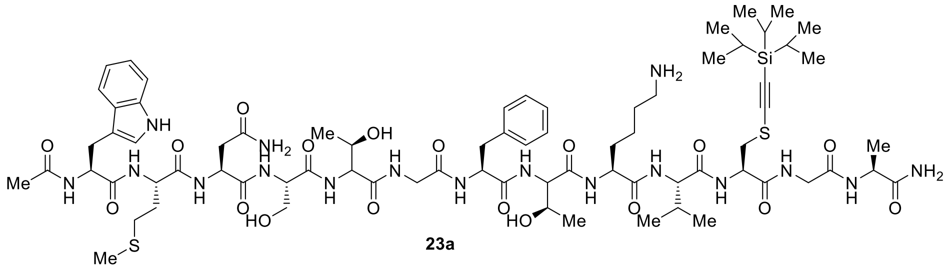

23a

23 (10.0 mg, 6.9 µmol) and **4a** (5.7 mg, 10.4 µmol) in 0.70 mL 10 mM Tris pH 7.4. Adapted general reaction procedure for **4a**. Yield for **23a** 77% (retention time = 13.7). Isolated yield 22% (2.5 mg, retention time = 15-17 min.)

HRMS (ESI/QTOF) m/z: $[M + 2H]^{+2}$ Calcd for $C_{74}H_{117}N_{17}O_{18}S_2Si^{+2}$ 811.8981; Found 811.8995.

HPLC gradient: Method 1. **Prep HPLC gradient**: Method 7.

HPLC-UV and HPLC-MS chromatogram

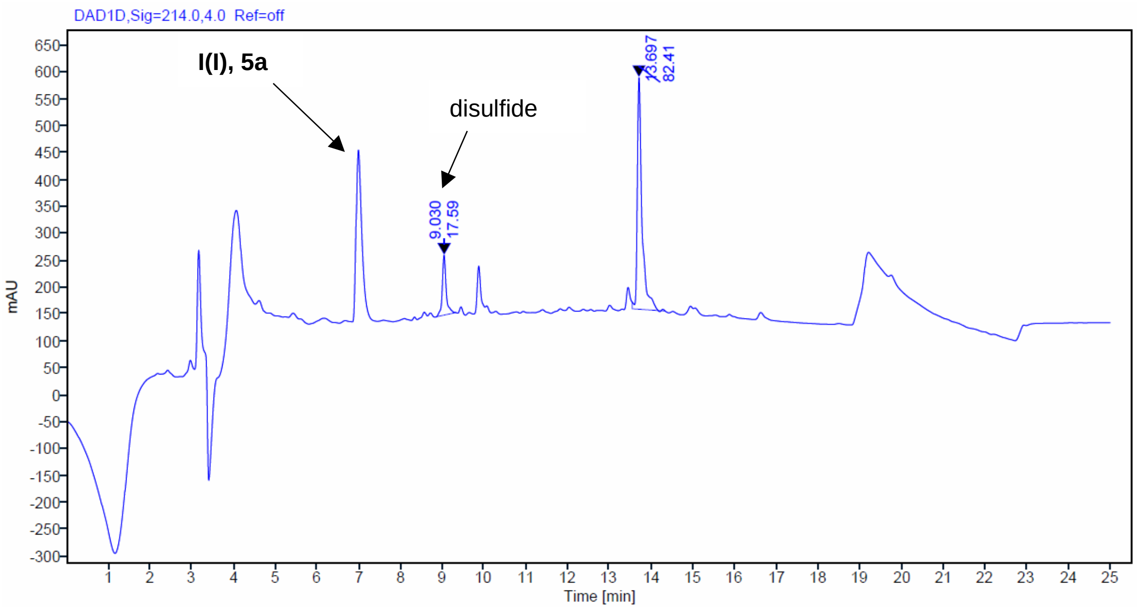

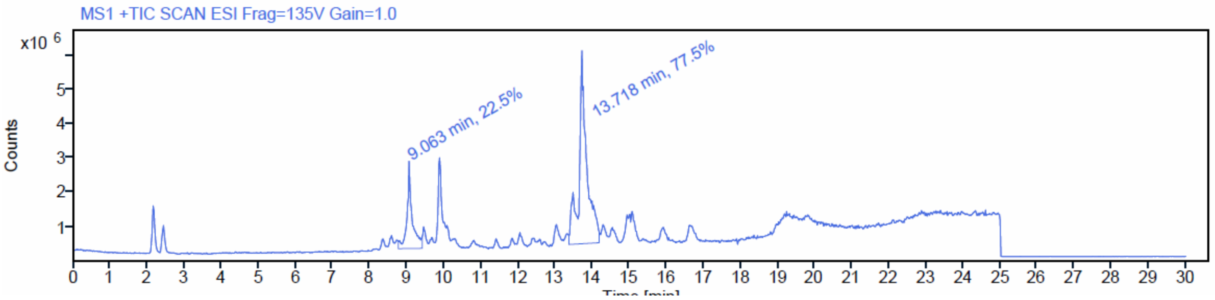

Calibration curve of **23a**

Preparation of stock solution. Pure **23a** (1.0 mg) dissolved in 90 uL of CH_3CN: H_2O (1:1). Stock solution was diluted separately and submitted for RP-HPLC. Calibrated yield, 80% (based on HPLC-UV), 83% (based on HPLC-MS).

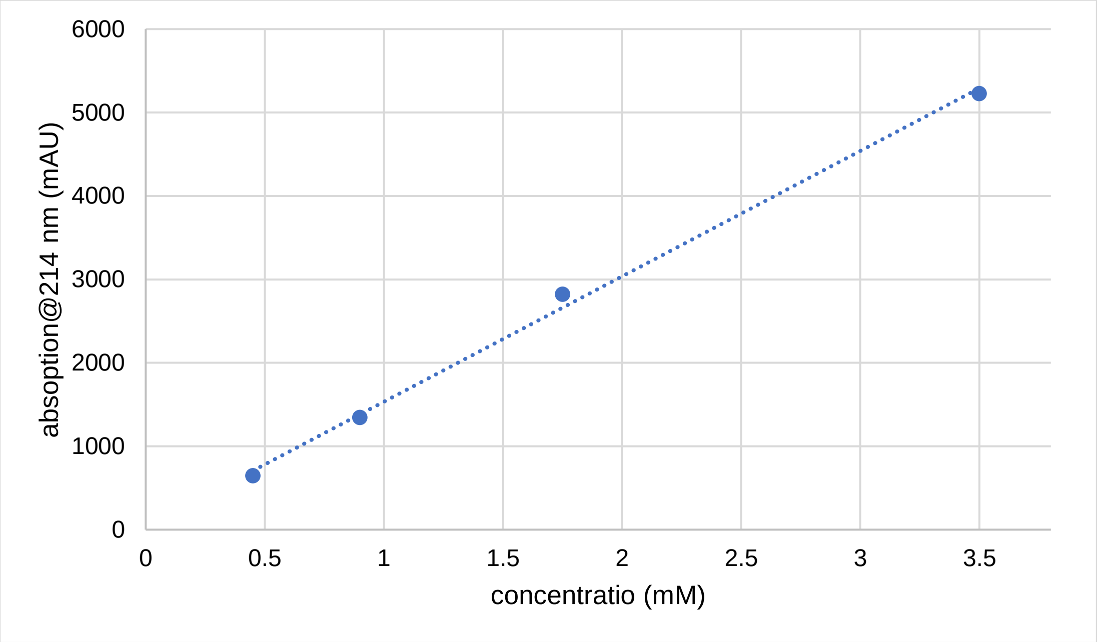

Figure S11. Calibration curve of **23a** based on HPLC-UV at 214nm.

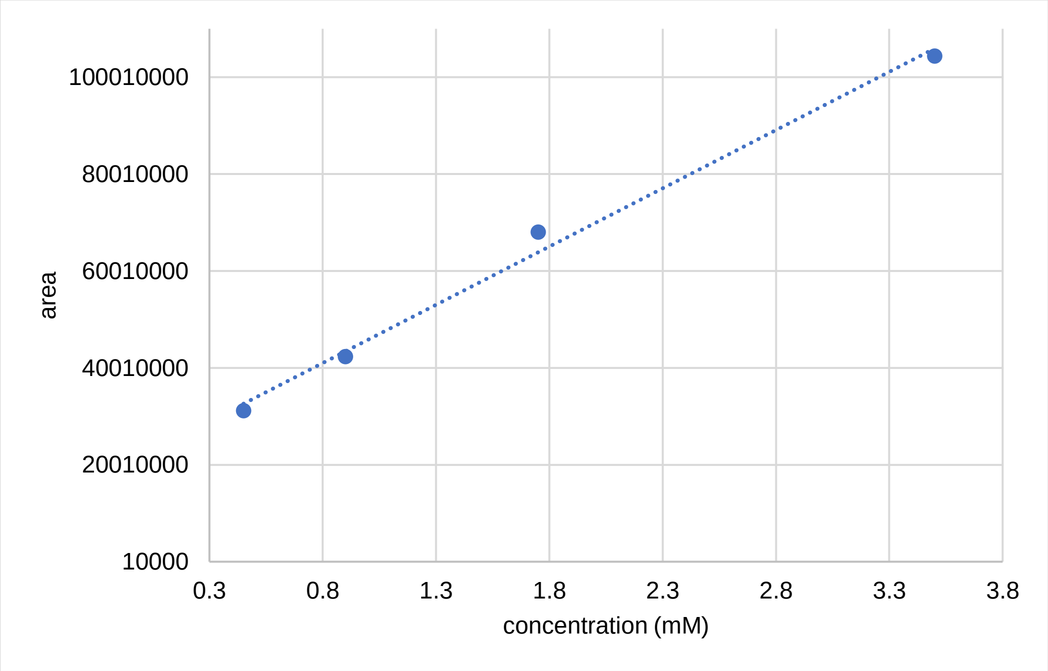

Figure S12. Calibration curve of **23a** based on HPLC-MS.

Alkynylated Ac-Trp-Met-Asn-Ser-Thr-Gly-Phe-Thr-Lys-Val-Cys-Gly-Ala-NH_2 (**23b**) and corresponding VBX (**23c**)

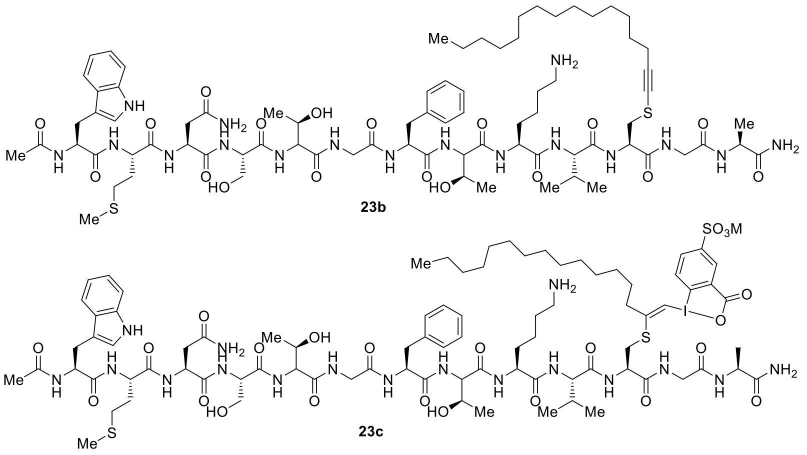

23 (1.0 mg, 0.69 µmol) and **4b** (0.48 mg, 0.83 µmol) in 86 µL 200 mM Tris pH 8.0. Adapted general reaction procedure for **4b**. Yield for **23b** 74% (retention time = 20.7), **23c** 17% (retention time = 14.8).

HRMS (ESI/QTOF) m/z: $[M + 2H]^{+2}$ Calcd for **23b** $C_{79}H_{125}N_{17}O_{18}S_2^{+2}$ 831.9409; Found 831.9423.

HRMS (ESI/QTOF) m/z: $[M]^-$ Calcd for **23c** $C_{86}H_{127}IN_{17}O_{23}S_3^-$ 1988.7503; Found 1988.7223.

HPLC gradient: Method 1.

HPLC-UV and HPLC-MS chromatogram

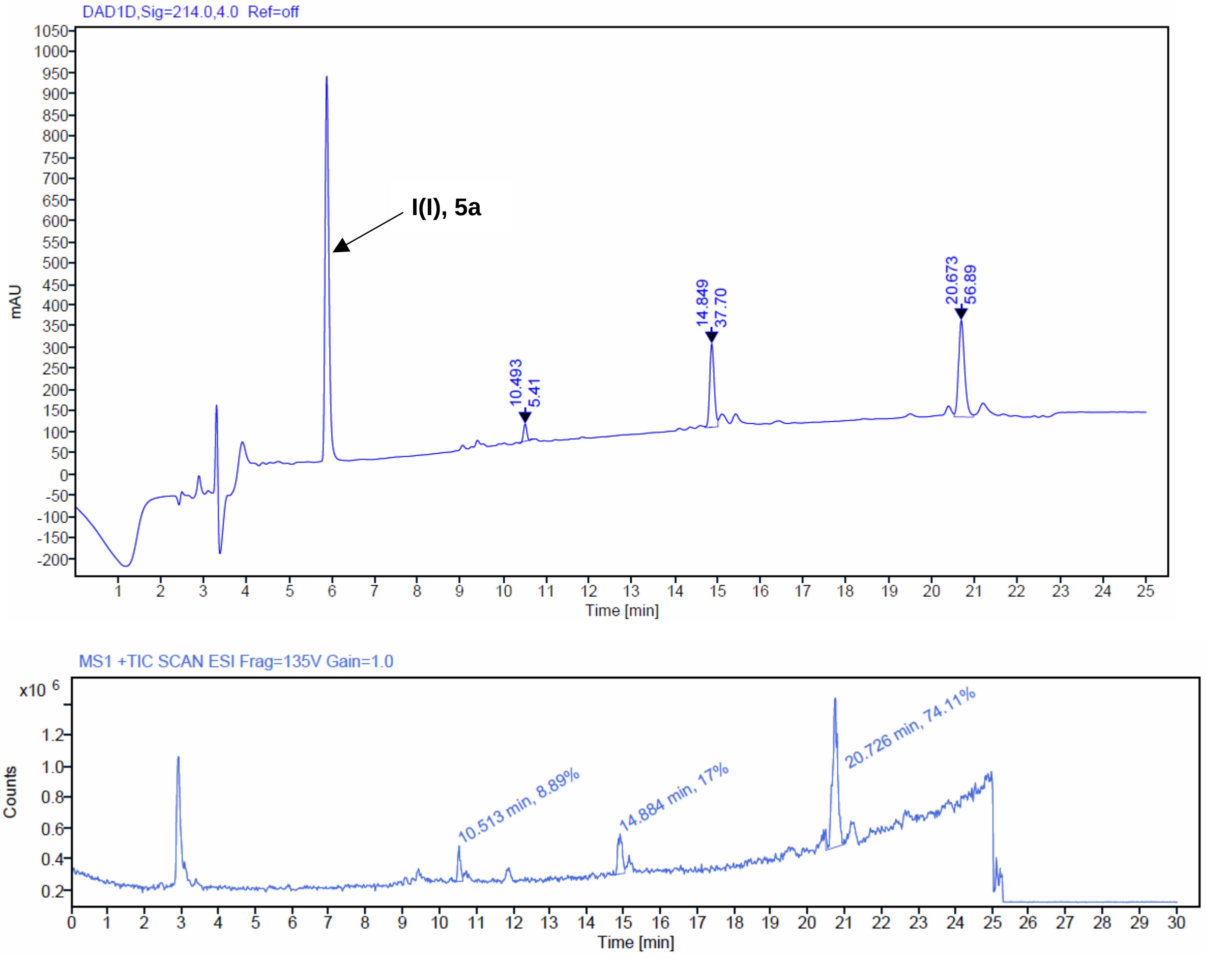

SUPPORTING INFORMATION

Alkynylated (Phe_{32}-Thr_{40}) Ac-Phe-His-Cys-Gln-Val-Cys-Phe-Ile-Thr-NH_2 (**24a**)

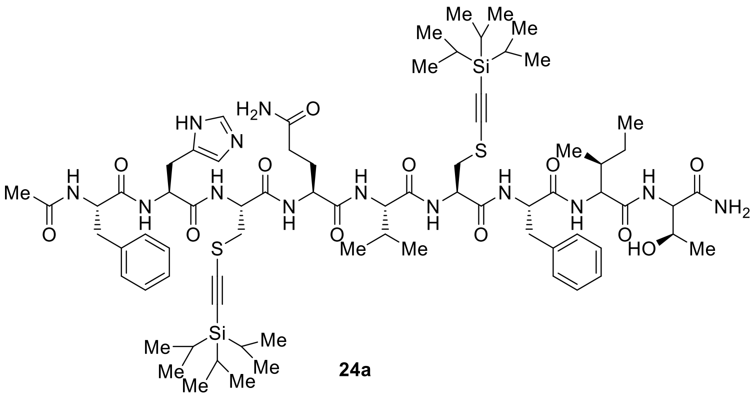

24a

24 (0.50 mg, 0.43 µmol) and **4a** (1.5 mg, 1.0 µmol) in 175 µL 10 mM Tris pH 7.4. Adapted general reaction procedure for **4a**. With 4.0 equiv. **4a,** Yield for **24a** 31% (retention time = 19.7), with 6.0 equiv. of **4a**, 57% for **24a**.

HRMS (ESI/QTOF) m/z: $[M + H]^+$ Calcd for $C_{74}H_{116}N_{13}O_{12}S_2Si_2^+$ 1498.7841; Found 1498.7869.

HPLC gradient: Method 1.

HPLC-MS chromatogram

After 14 h with 4.0 equivalent of **4a**

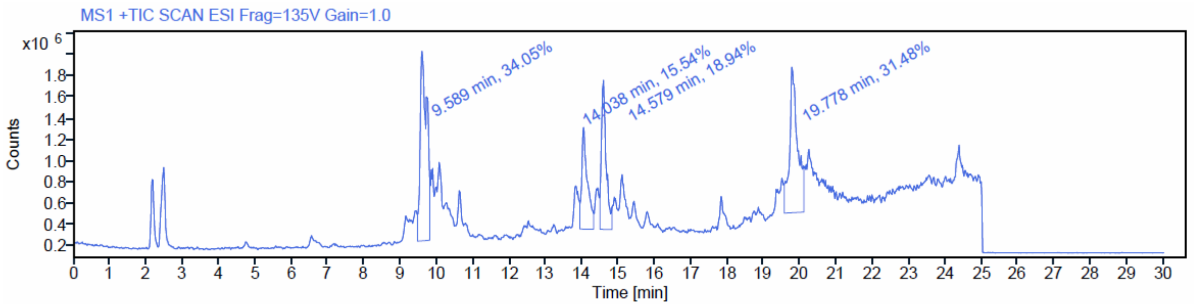

After 14 h with 6.0 equivalent of **4a**

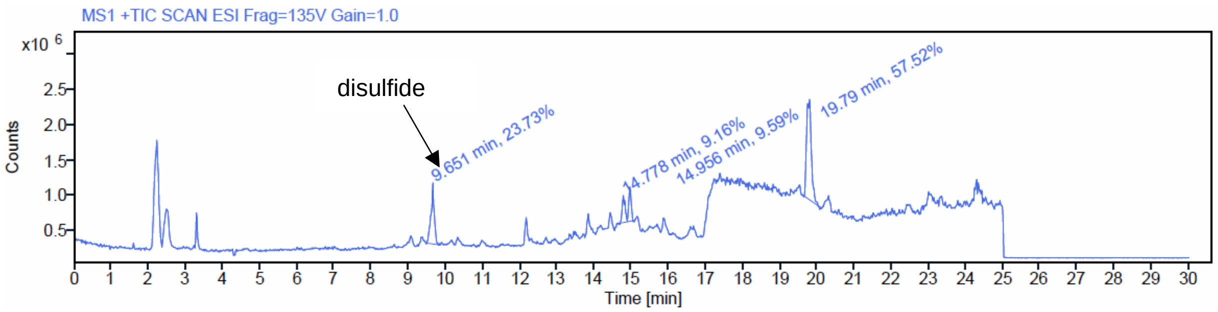

SUPPORTING INFORMATION

Alkynylated His_6-Cys-Ub (**25a**)

In 1.5 mL vial charged with **25** (0.4 mg, 0.04 µmol, 1.0 equiv.) and **4a** (0.20 mg, 0.36 µmol, 10 equiv.) dissolved in 200 µL 10 mM Tris pH 7.4 with small magnetic bar. Then reaction mixture was heated at 37 °C for 6 h. After 6 h an aliquot of the reaction mixture submitted for HPLC. Yield for **25a** 95% (retention time = 16.9).

ESI-MS m/z: $[M + 15H]^{+15}$ Calcd for $C_{74}H_{117}N_{17}O_{18}S_2Si^{+15}$ 726.3; Found 726.4**.**

HPLC gradient: Method 4.

HPLC-UV and HPLC-MS chromatogram

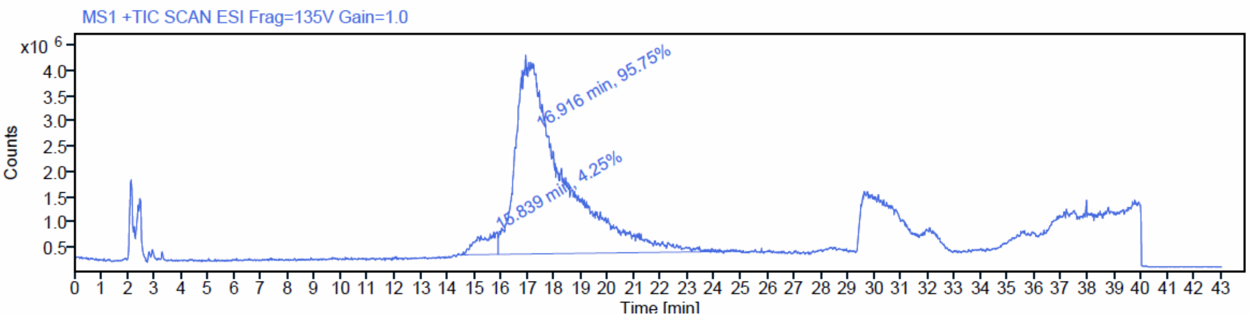

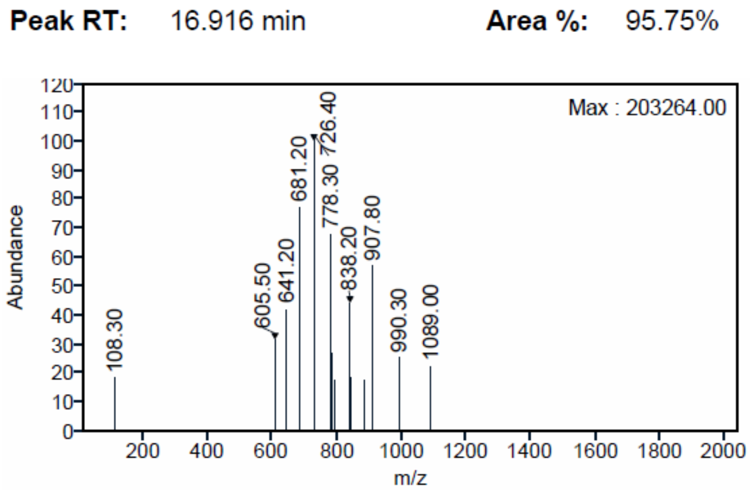

10. Synthesis of thioesters in one-pot

Reaction procedure for preparation of silylthio ester of Ac-Ala-Cys-Gly-Phe-NH_2 **11aa** in one-pot

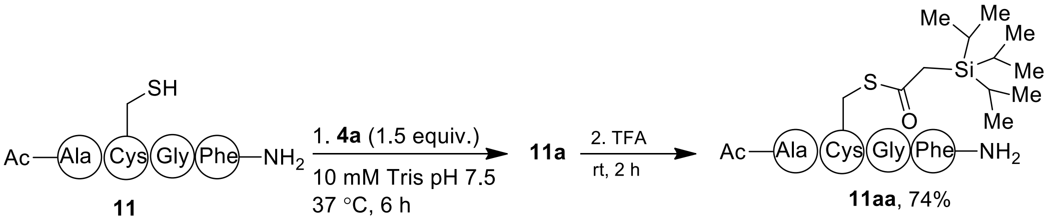

A 1.5 mL vial was charged with **11** (0.50 mg, 1.1 µmol, 1.0 equiv.) and **4a** (1.0 mg, 1.7 µmol, 1.5 equiv.) in 114 µL of 10 mM Tris buffer pH 7.4 with small magnetic stirring bar. The reaction mixture was stirred at 37 °C for 6 h. The buffer was used directly from a freshly prepared solution without degassing. After 6 h, an aliquot of 10 µL of the reaction mixture was diluted with 30 µL acetonitrile:water (1:1) to give a clear solution. The solution was submitted to HPLC. The HPLC revealed the complete consumption of **11**. TFA (50 µL, 0.65 mmol) was added and the reaction mixture was allowed to stir at rt for additional 2 h. After 2 h, 74% **11aa** (retention time = 12.5) and 17% disulfide (retention time = 6.3) were observed by HPLC-MS.

HRMS (nanochip-ESI/LTQ-Orbitrap) m/z: $[M + H]^+$ Calcd for $C_{30}H_{50}N_5O_6SSi^+$ 636.3246; Found 636.3258.

HPLC gradient: Method 3.

HPLC-UV and HPLC-MS chromatogram

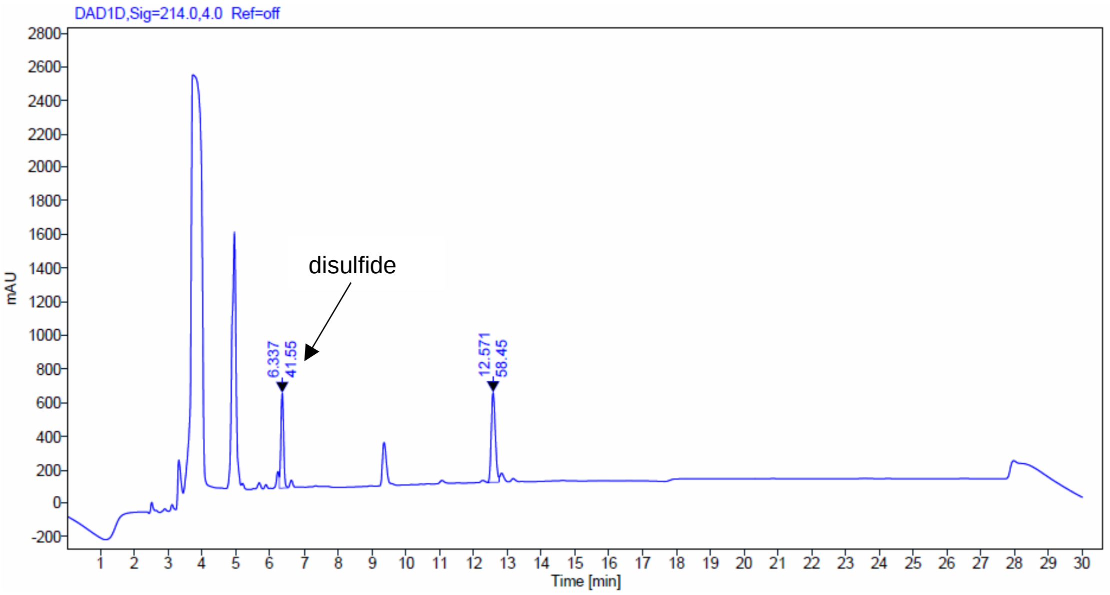

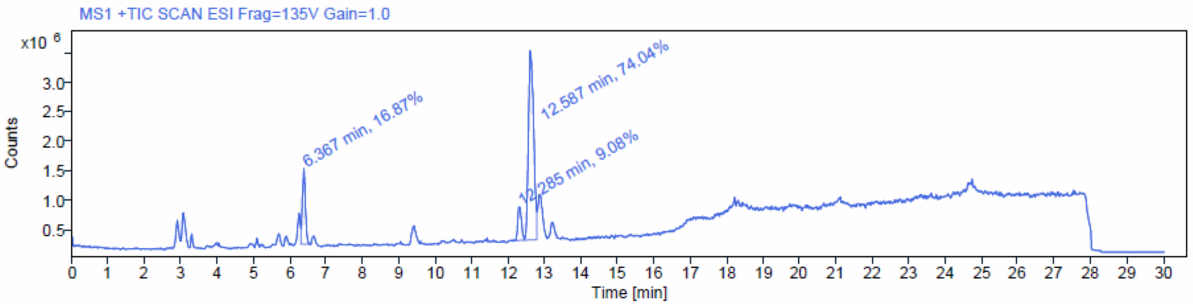

Reaction procedure for the preparation of the palmitolyl-ester of Ac-Ala-Cys-Gly-Phe-NH_2 **11bb** in one -pot

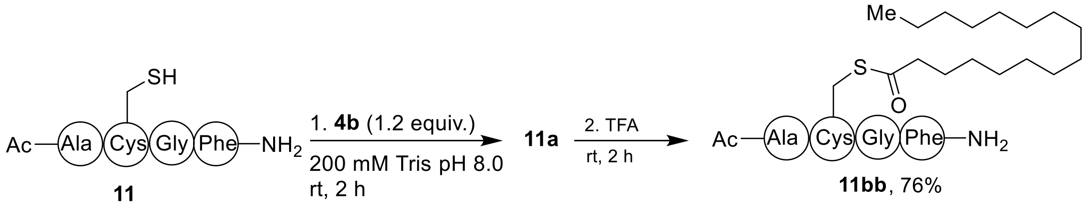

A 1.5 mL vial was charged with **11** (0.50 mg, 1.1 µmol,1.0 equiv.) and **4b** (0.80 mg, 1.4 µmol, 1.2 equiv.) in 114 µL of 200 mM Tris buffer pH 8.0 with a small magnetic stirring bar. The reaction mixture was stirred at rt for 2 h. The buffer was used directly from a freshly prepared solution without degassing. After 2 h, an aliquot of 10 µL of the reaction mixture was diluted with 30 µL acetonitrile:water (1:1) to give a clear solution. The solution was submitted to HPLC. The HPLC revealed the complete consumption of **11**. TFA (50 µL, 0.65 mmol) was added and the reaction mixture was allowed to stir at rt for additional 2 h. After 2 h, 76% **11bb** (retention time = 20.8) and 16% VBX (**11c**, retention time = 11.5) were observed by HPLC-MS.

HRMS (nanochip-ESI/LTQ-Orbitrap) m/z: $[M + H]^+$ Calcd for $C_{35}H_{58}N_5O_6S^+$ 676.4102; Found 676.4114.
HPLC gradient: Method 3.
HPLC-UV and HPLC-MS chromatogram

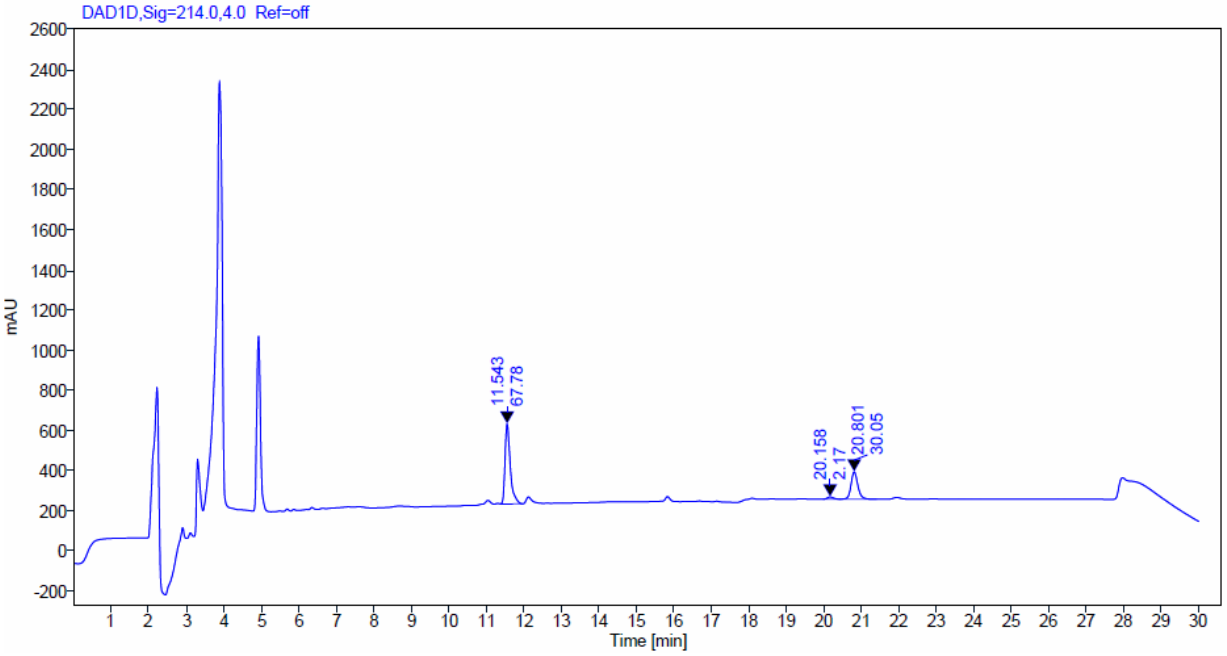

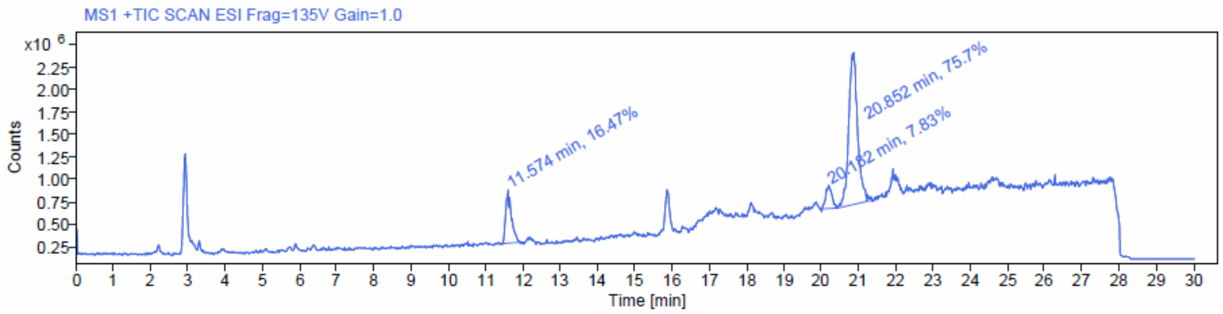

Preparation of the silylthioester of H-Gly-Cys-Ala-Phe-Lys-Thr-NH_2, **17aa**

A 5 mL vial was charged with **17** (10.0 mg, 15.7 µmol,1.0 equiv.) and **4a** (13.0 mg, 23.5 µmol, 1.5 equiv.) in 1.0 mL of 10 mM Tris buffer pH 7.4 with a small magnetic stirring bar. The reaction mixture was stirred at 37°C for 5 h. After 5 h, an aliquot of 10 µL of the reaction mixture was diluted with 80 µL acetonitrile: water (1:1). The solution was submitted to HPLC. The HPLC revealed the complete consumption of **17**. TFA (1.0 mL) was added and the reaction mixture was allowed to stir at rt for additional 2 h. After 2 h, an aliquot of 10 µL of the reaction mixture was diluted with 90 µL acetonitrile: water (1:1) to give a clear solution. The solution was submitted to HPLC. 58% **17aa** (retention time = 10.4) was observed by HPLC-MS.

HRMS (ESI/QTOF) m/z: $[M + H]^+$ Calcd for $C_{38}H_{67}N_8O_8SSi^+$ 823.4566; Found 823.4560.

HPLC gradient: Method 3.

HPLC-UV and HPLC-MS chromatogram

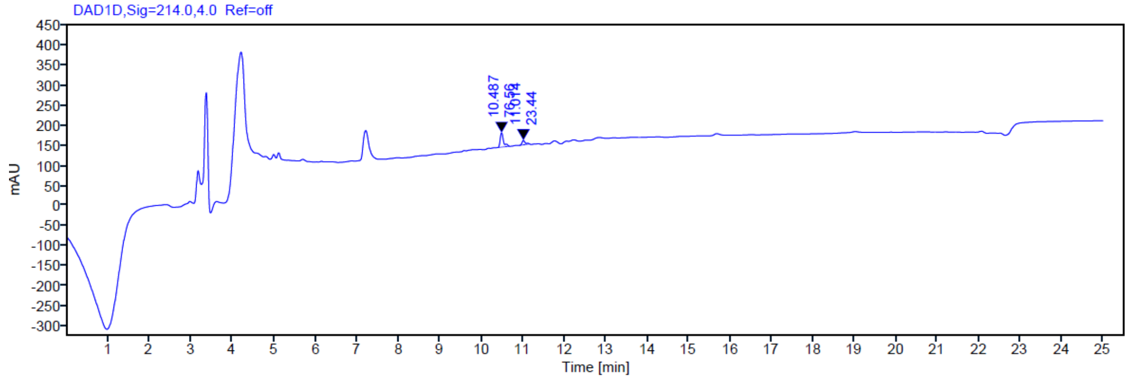

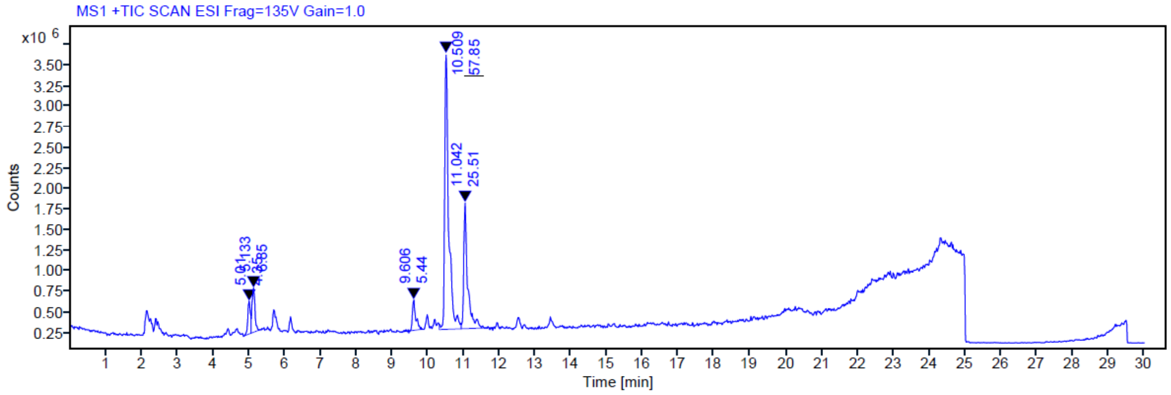

SUPPORTING INFORMATION

Preparation of the palmitoyl thioester of H-Gly-Cys-Ala-Phe-Lys-Thr-NH_2, **17bb**

A 5 mL vial was charged with **17** (15.0 mg, 23.5 µmol,1.0 equiv.) and **4b** (17.0 mg, 28.2 µmol, 1.2 equiv.) in 1.6 mL of 200 mM Tris buffer pH 8.0 with a small magnetic stirring bar. The reaction mixture was stirred at rt for 2 h. The buffer was used directly from a freshly prepared solution without degassing. After 2 h, an aliquot of 10 µL of the reaction mixture was diluted with 80 µL acetonitrile:water (1:1) to give a clear solution. The solution was submitted to HPLC. The HPLC revealed the complete consumption of **11**. TFA (1.5 mL) was added and the reaction mixture was allowed to stir at rt for additional 2 h. After 2 h, an aliquot of 10 µL of the reaction mixture was diluted with 90 µL acetonitrile:water (1:1) to give a clear solution. The solution was submitted to HPLC. 73% **17bb** (retention time = 13.4) and 21% VBX (**17c**, retention time = 12.8) were observed by HPLC-MS. Isolated Yield of **17bb**: 34% (7.0 mg, 8.1 µmol, retention time 12-15 min).

HRMS (ESI/QTOF) m/z: $[M + H]^+$ Calcd for $C_{43}H_{75}N_8O_8S^+$ 863.5423; Found 863.5425.

HPLC gradient: Method 1.

Prep HPLC Gradient: Method 5.

HPLC-UV and HPLC-MS chromatogram

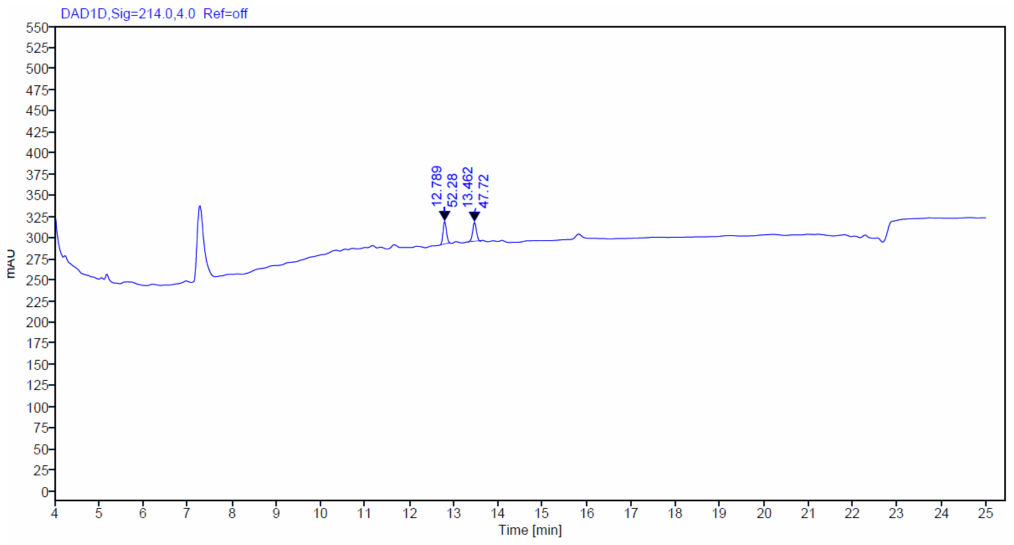

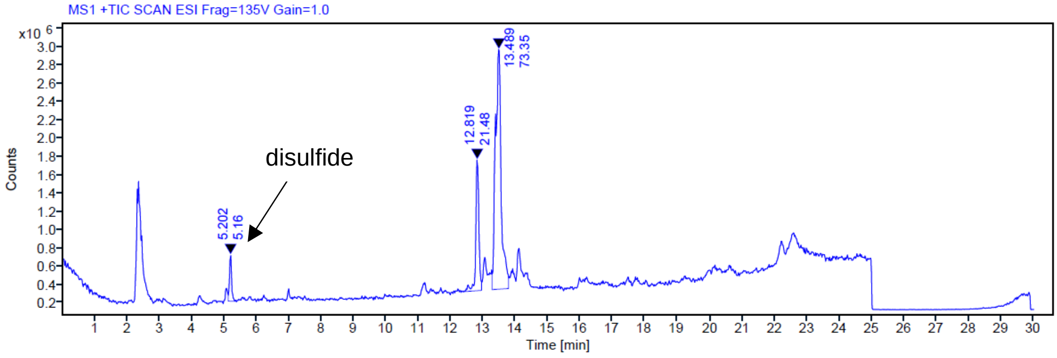

Calibration curve of **17bb**

Preparation of stock solution. Pure **17bb** (0.5 mg) dissolved in 116 μL of CH_3CN: H_2O (1:1). Stock solution was diluted separately and submitted for RP-HPLC. Calibrated yield 50% (based on HPLC-UV), 40% (based on HPLC-MS).

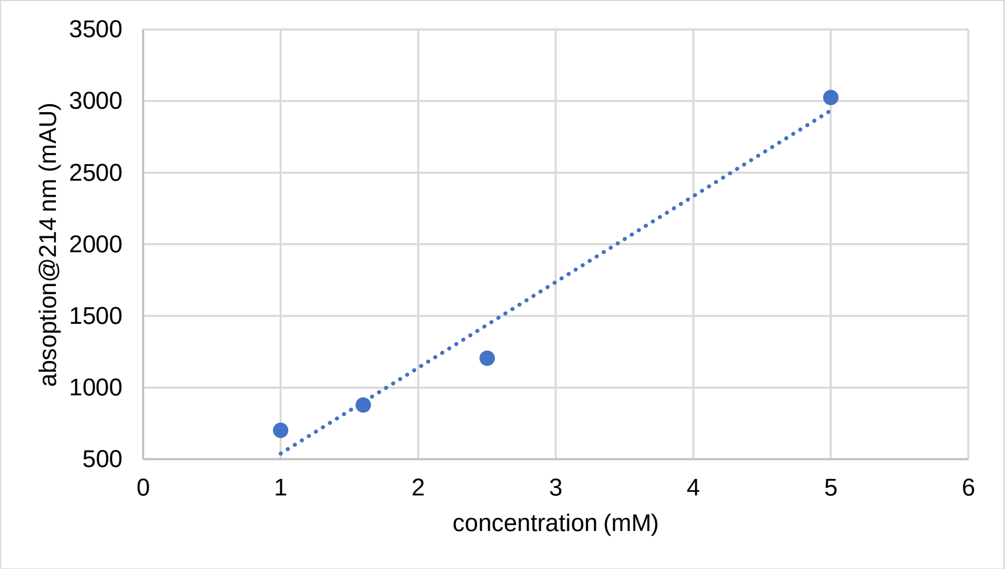

Figure S13. Calibration curve of **17bb** based on HPLC-UV absorption at 214 nm.

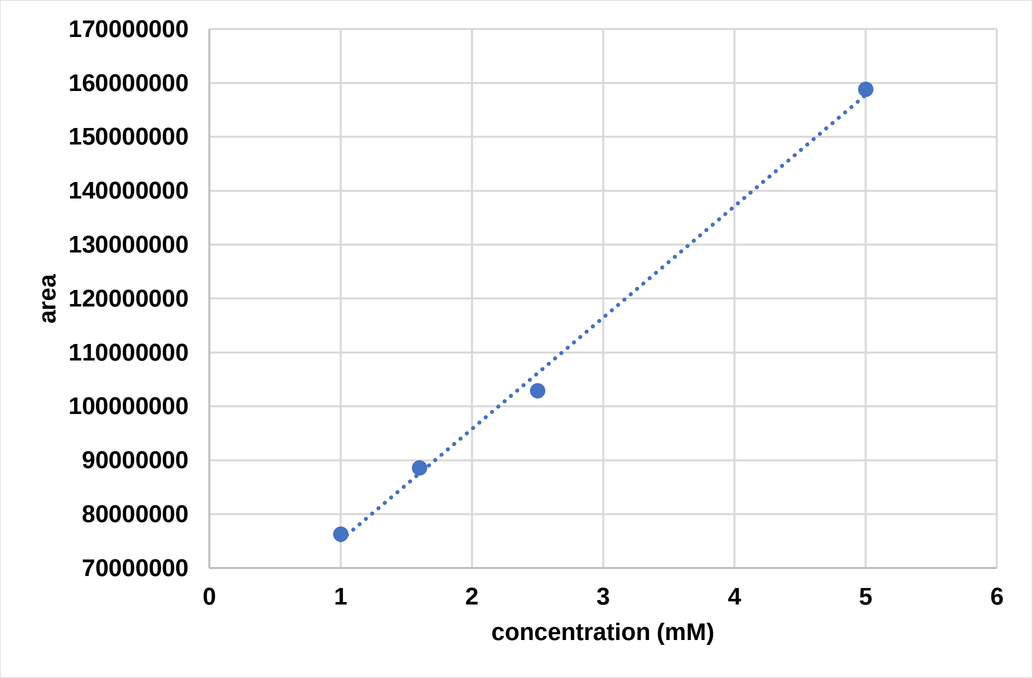

Figure S14. Calibration curve of **17bb** based on HPLC-MS.

SUPPORTING INFORMATION

Preparation of the silylthioester of H-Ala-Cys-Ala-Phe-Lys-Asp-NH_2, **18aa**

A 5 mL vial was charged with **17** (10.0 mg, 15.3 µmol,1.0 equiv.) and **4a** (9.8 mg, 22.9 µmol, 1.5 equiv.) in 1.0 mL of 10 mM Tris buffer pH 7.4 with a small magnetic stirring bar. The reaction mixture was stirred at 37°C for 5 h. The freshly prepared buffer was used without degassing. After 5 h, an aliquot of 10 µL of the reaction mixture was diluted with 80 µL acetonitrile: water (1:1). The solution was submitted to HPLC. The HPLC revealed the complete consumption of **17**. TFA (1.0 mL) was added and the reaction mixture was allowed to stir at rt for additional 2 h. After 2 h, an aliquot of 10 µL of the reaction mixture was diluted with 90 µL acetonitrile: water (1:1) to give a clear solution. The solution was submitted to HPLC. 58% **18aa** (retention time = 15.3) was observed by HPLC-MS. Isolated Yield of **18aa** 35% (4.5 mg, 5.4 µmol, retention time = 16-17 min)

HPLC gradient: Method 2.

HRMS (ESI/QTOF) m/z: $[M + H]^+$ Calcd for $C_{39}H_{67}N_8O_9SSi^+$ 851.4515; Found 851.4521.

Prep-HPLC gradient: Method 5.

HPLC-UV and HPLC-MS chromatogram

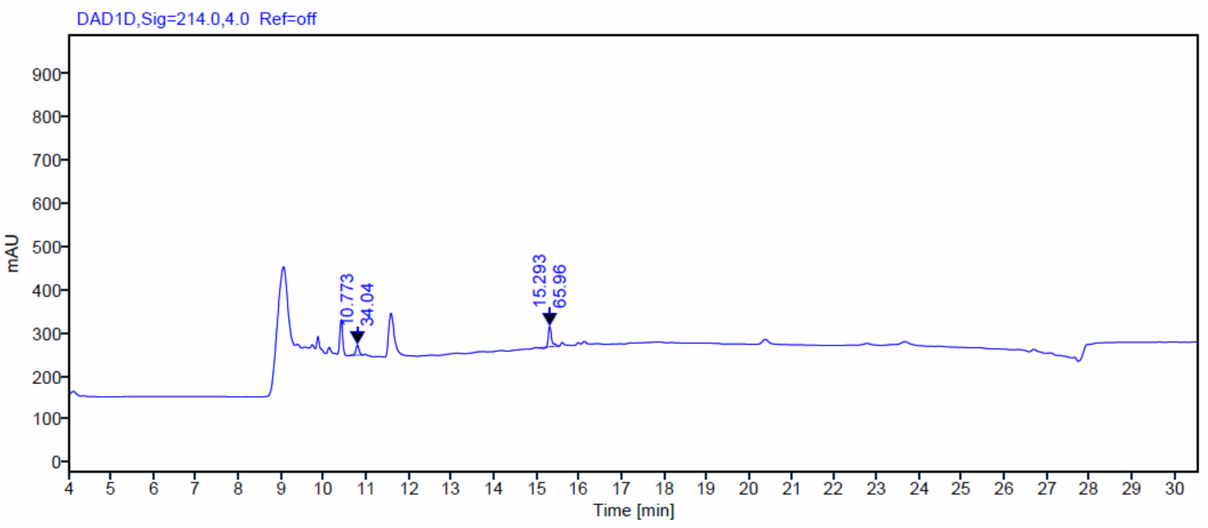

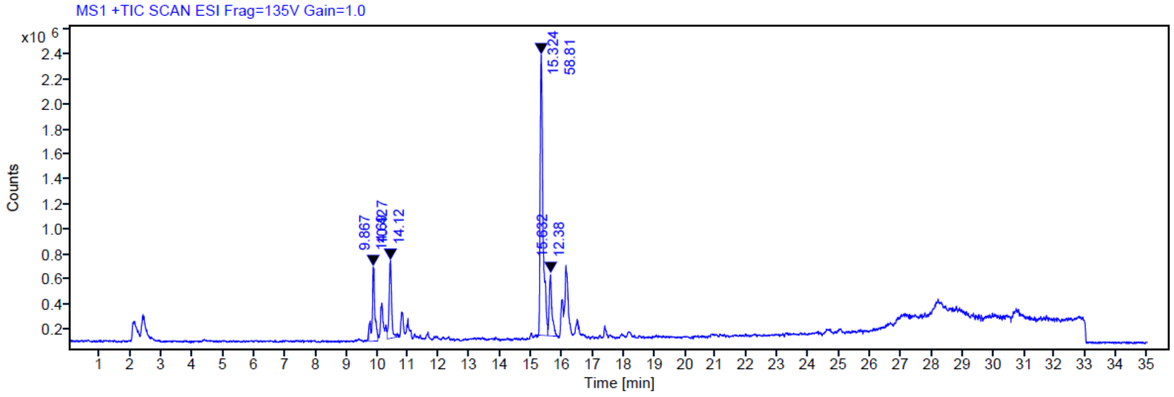

SUPPORTING INFORMATION

Preparation of the silylthioester of Ac-Trp-Met-Asn-Ser-Thr-Gly-Phe-Thr-Lys-Val-Cys-Gly-Ala-NH_2, **23aa**

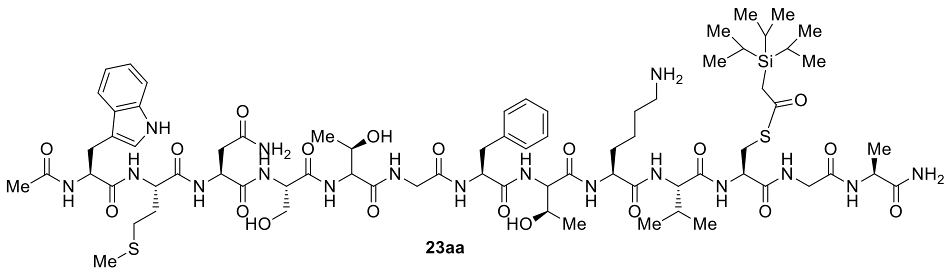

23aa

23 (1.0 mg, 0.69 µmol) and **4a** (0.60 mg, 1.0 µmol) were dissolved in 70 µL of 10 mM Tris buffer pH 7.4. Then 50 µL TFA was added. The same procedure was followed as for **11aa**. HPLC-MS yield for **23aa** was 82% (retention time = 14.5).

HRMS (nanochip-ESI/LTQ-Orbitrap) m/z: $[M + 2H]^{+2}$ Calcd for **23aa** $C_{74}H_{119}N_{17}O_{19}S_2Si^{+2}$ 820.9034; Found 820.9073.

HPLC gradient: Method 1.

HPLC-UV and HPLC-MS chromatogram

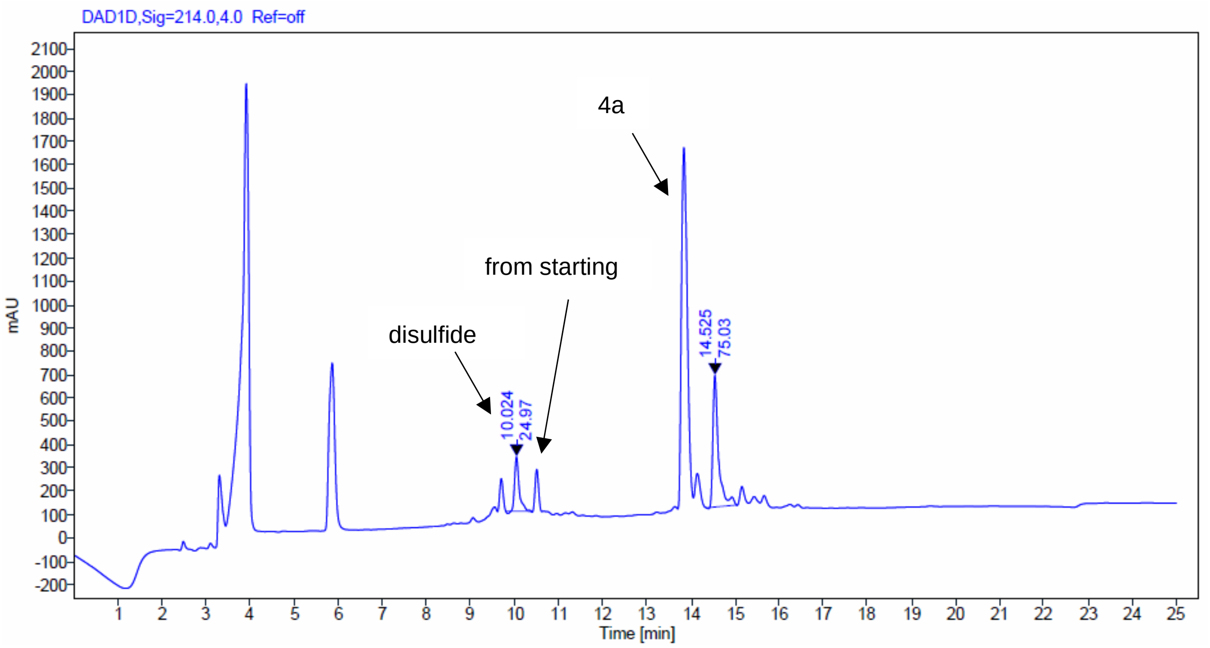

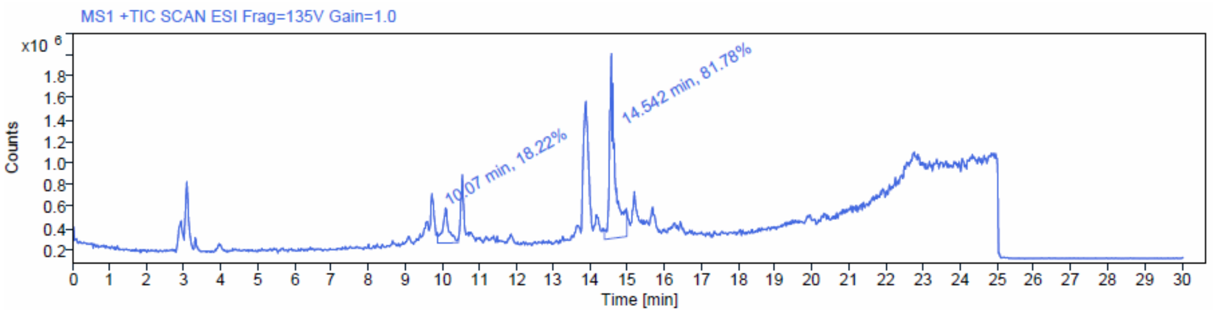

Preparation of the palmitoylthioester of Ac-Trp-Met-Asn-Ser-Thr-Gly-Phe-Thr-Lys-Val-Cys-Gly-Ala-NH_2 (**23bb)**

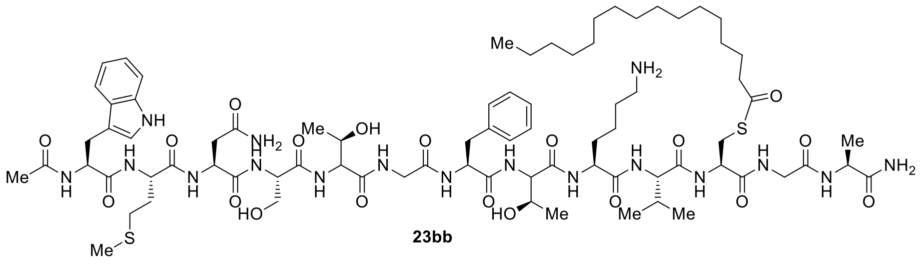

23bb

23 (1.0 mg, 0.69 µmol) and **4b** (0.48 mg, 0.83 µmol) were dissolved in 85 µL of 200 mM Tris buffer pH 8.0. The same procedure was followed as for **11bb**. Yield for **23bb** 67% (retention time = 20.6), **23c** 19% (retention time = 14.8).

HRMS (ESI/QTOF) m/z: $[M + 2H]^{+2}$ Calcd for **23b** $C_{79}H_{125}N_{17}O_{18}S_2^{+2}$ 831.9409; Found 831.9423.

HPLC gradient: Method 1.

HPLC-UV and HPLC-MS chromatogram

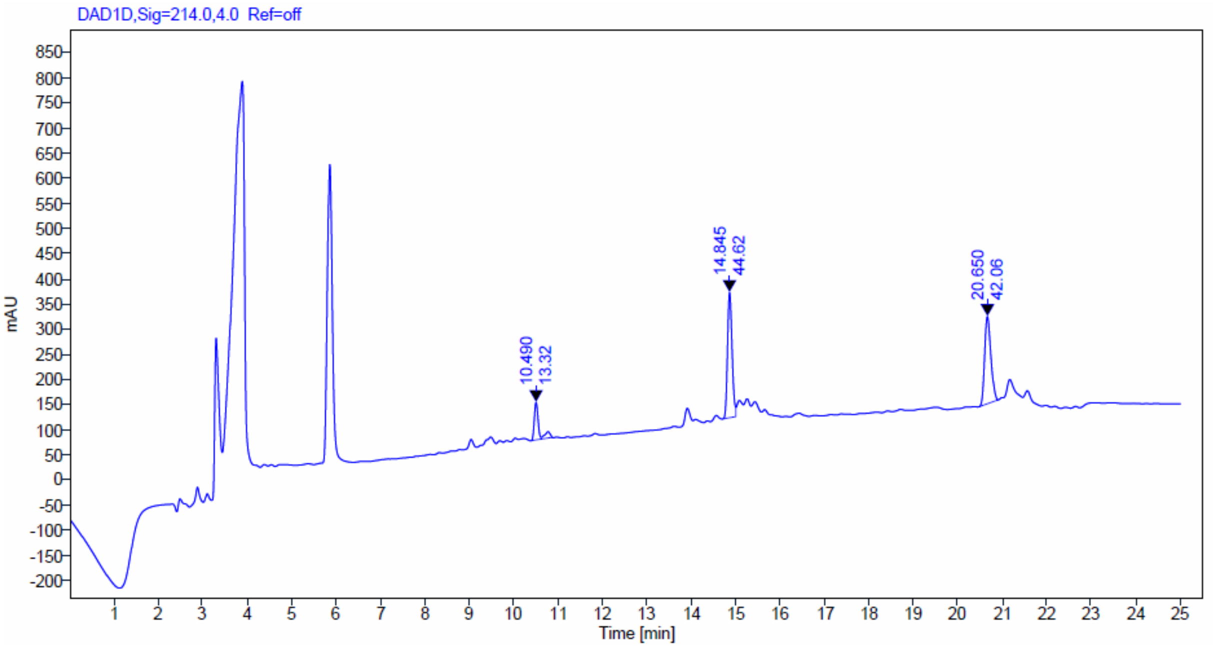

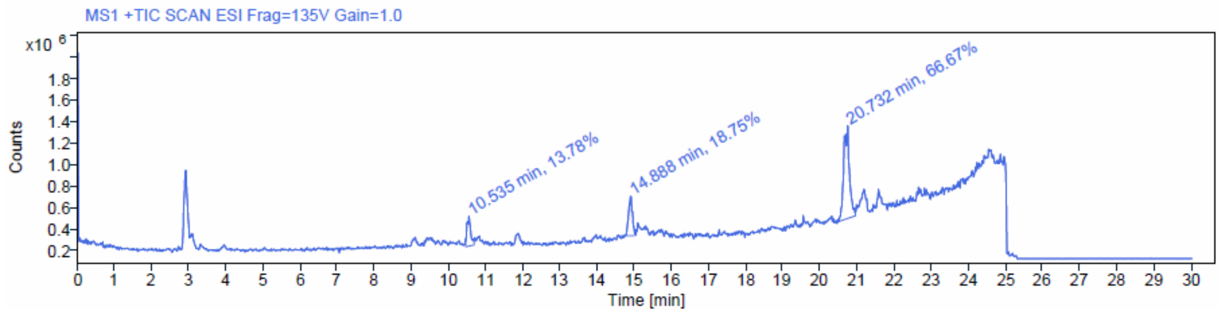

Cleavage of palmitoylthioester of Ac-Trp-Met-Asn-Ser-Thr-Gly-Phe-Thr-Lys-Val-Cys-Gly-Ala-NH_2 **(17bb)**

A 1.5 mL vial was charged with **17bb** (0.5 mg, 23.5 µmol,1.0 equiv.) 70 µL 1M NH_2OH with a small magnetic bar. The NH_2OH solution was freshly prepared in Milli Q water. Then the reaction mixture was stirred for 4 h at rt. After 4 h, an aliquot of 10 µL of the reaction mixture was diluted with 40 µL acetonitrile: water (1:1). The solution was submitted to HPLC. The HPLC revealed quantitative conversion. Yield for **17** and disulfide quantitative (retention time 5.0).

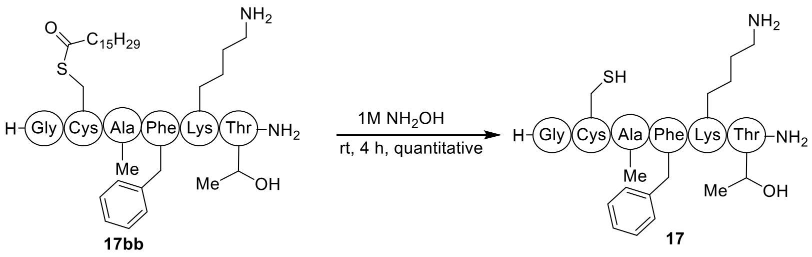

HRMS (ESI/QTOF) m/z: $[M + H]^+$ Calcd for $C_{27}H_{45}N_8O_7S^+$ 625.3126; Found 625.3143.

HPLC gradient: Method 1.

HPLC-UV chromatogram of starting material **17bb**

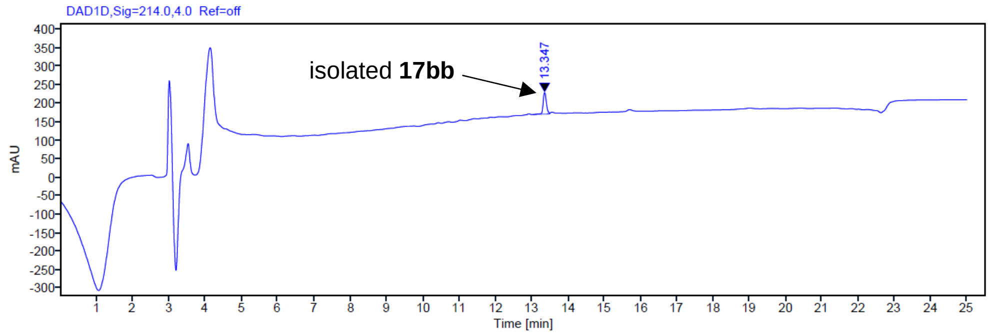

HPLC-UV and HPLC-MS chromatogram of reaction mixture

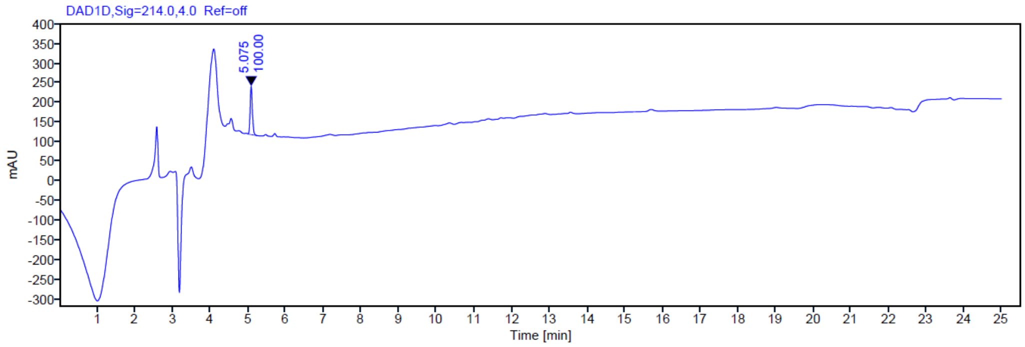

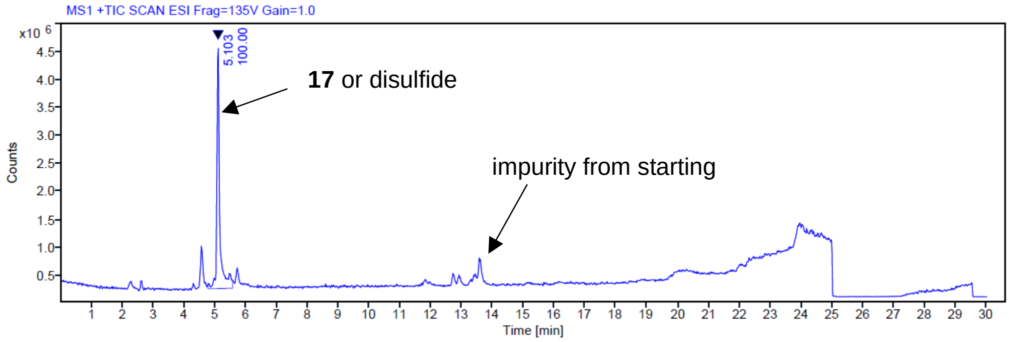

Cleavage of TIPS-thioester of Ac-Trp-Met-Asn-Ser-Thr-Gly-Phe-Thr-Lys-Val-Cys-Gly-Ala-NH_2 (**18aa**)

A 1.5 mL vial was charged with isolated **18aa** (0.5 mg, 0.6 µmol,1.0 equiv.) and KF (1.0 mg, 17.2 µmol, 28.7 equiv.) and 70 µL 1M NH_2OH with a small magnetic bar. The NH_2OH solution was freshly prepared in Milli Q water. Then the reaction mixture was stirred for 14 h at 37 °C. After 14 h, an aliquot of 10 µL of the reaction mixture was diluted with 40 µL water. The solution was submitted to HPLC. The HPLC revealed 90% conversion after 14 h.

HRMS (ESI/QTOF) m/z: $[M + H]^+$ Calcd for $C_{28}H_{45}N_8O_8S^+$ 653.3076; Found 653.309.

HPLC gradient: Method 1.

HPLC-UV chromatogram of starting material **18aa**

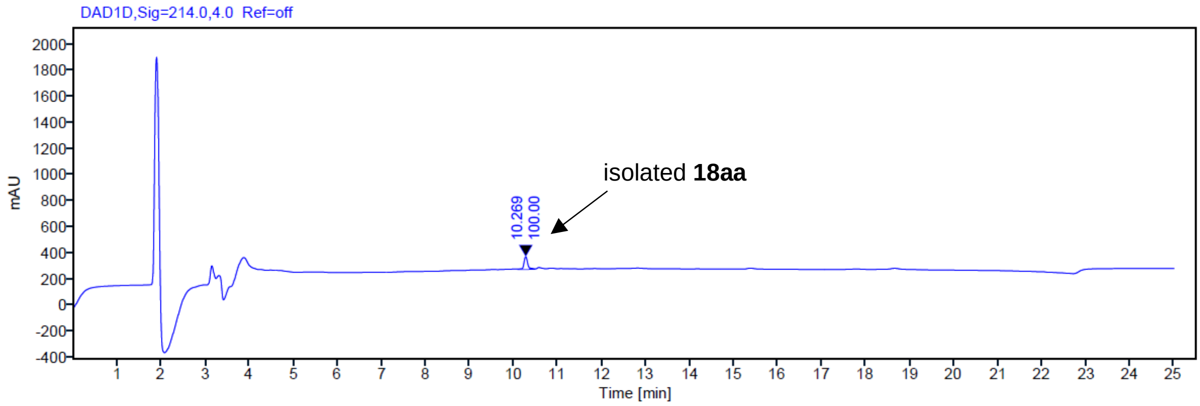

HPLC-UV and HPLC-MS chromatogram of reaction mixture

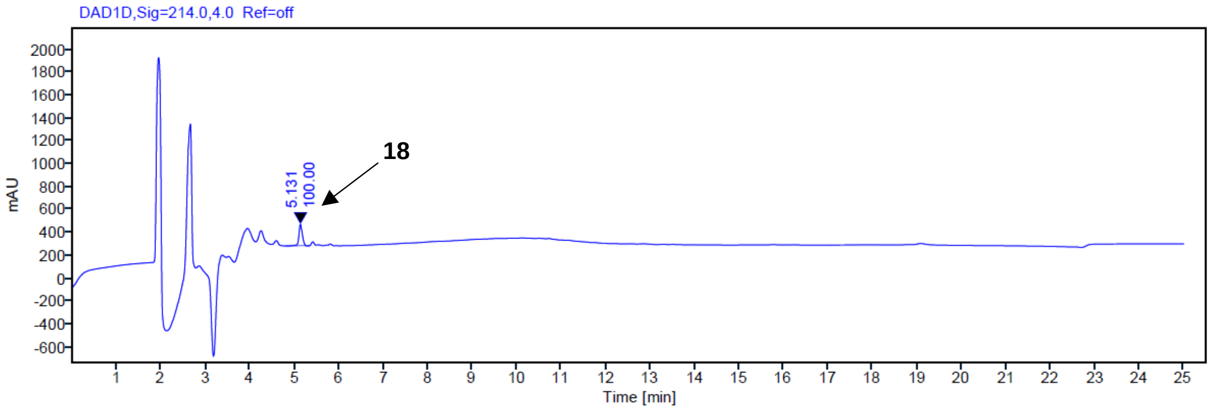

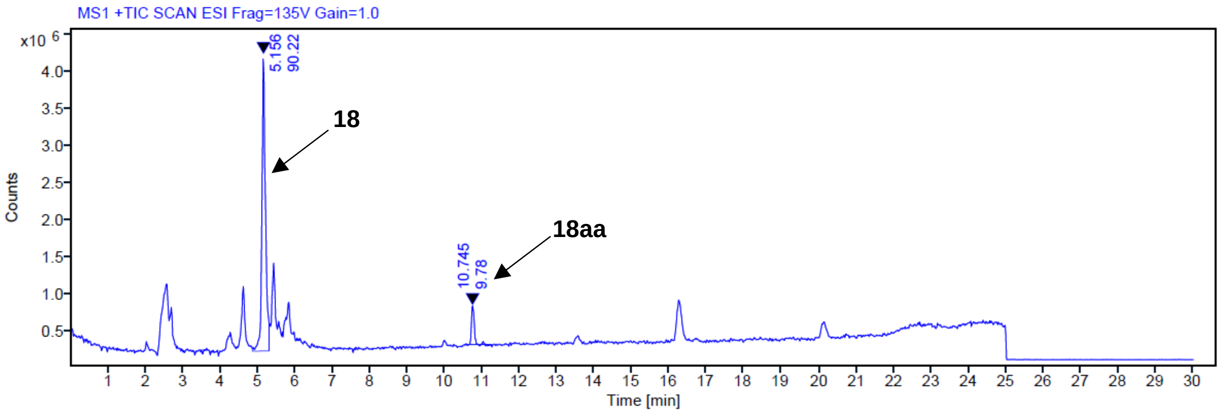

SUPPORTING INFORMATION

11. NMR spectra of isolated products

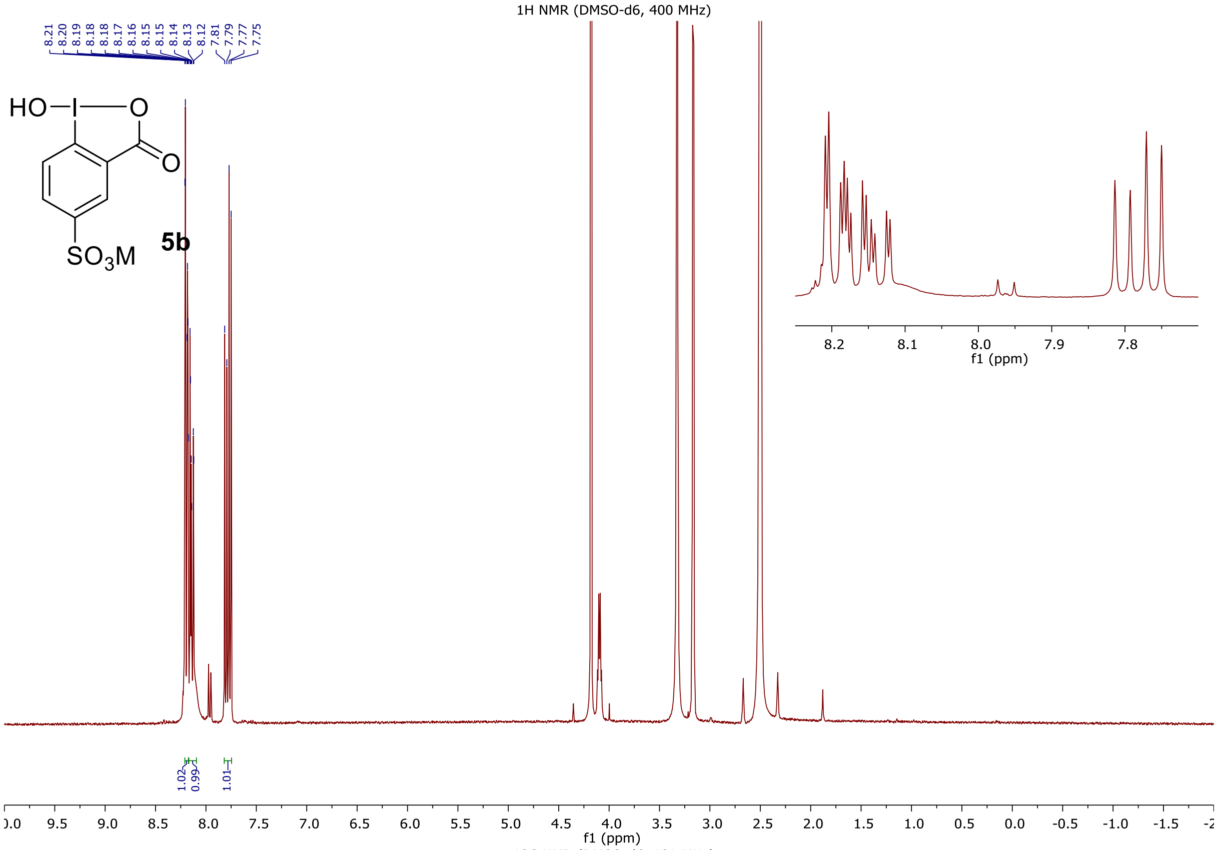

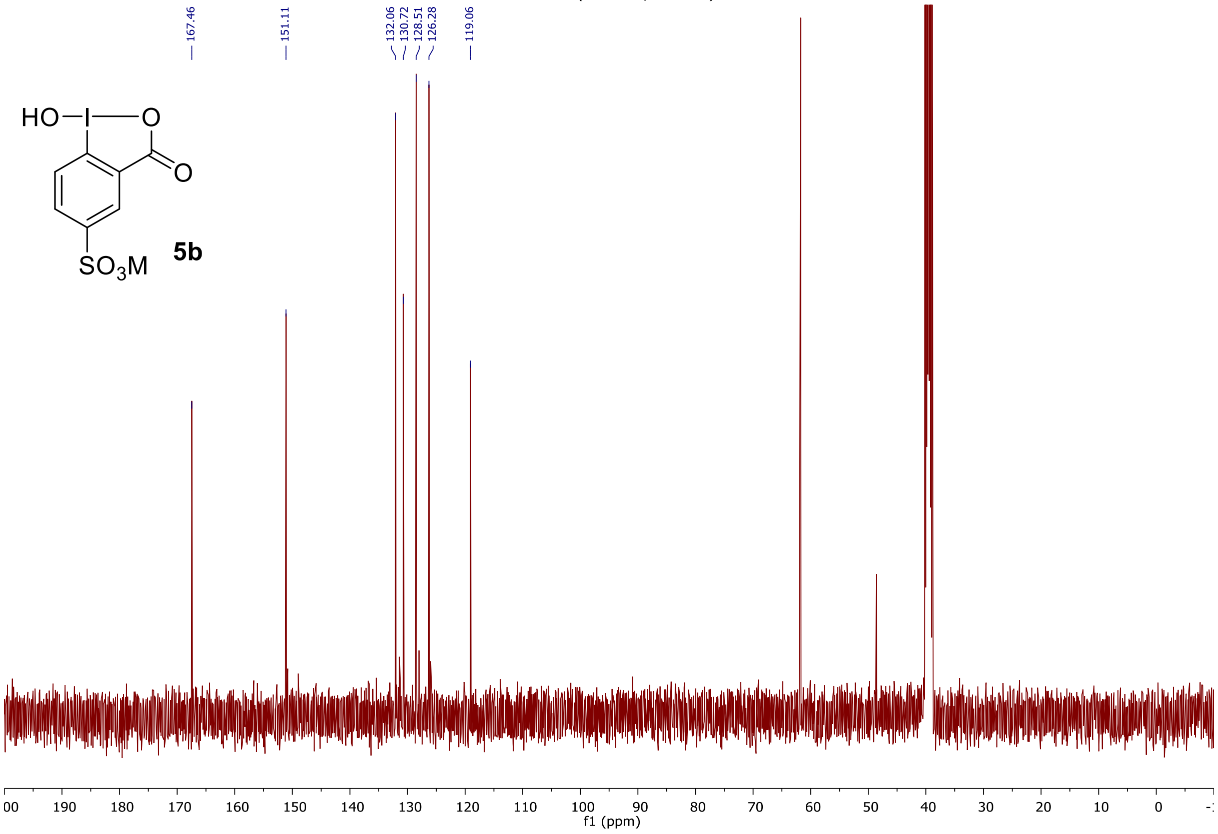

1H NMR (DMSO-d6, 400 MHz)

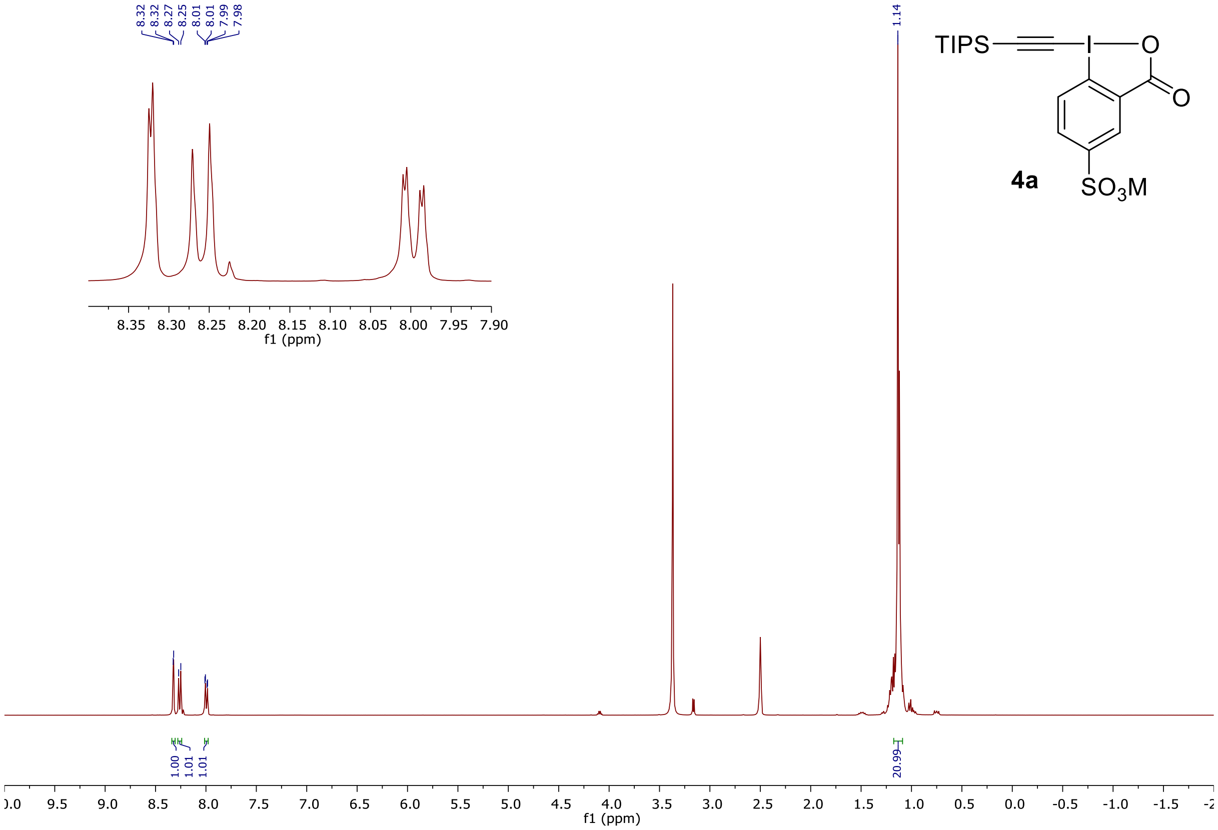

13C NMR (DMSO-d6, 101 MHz)

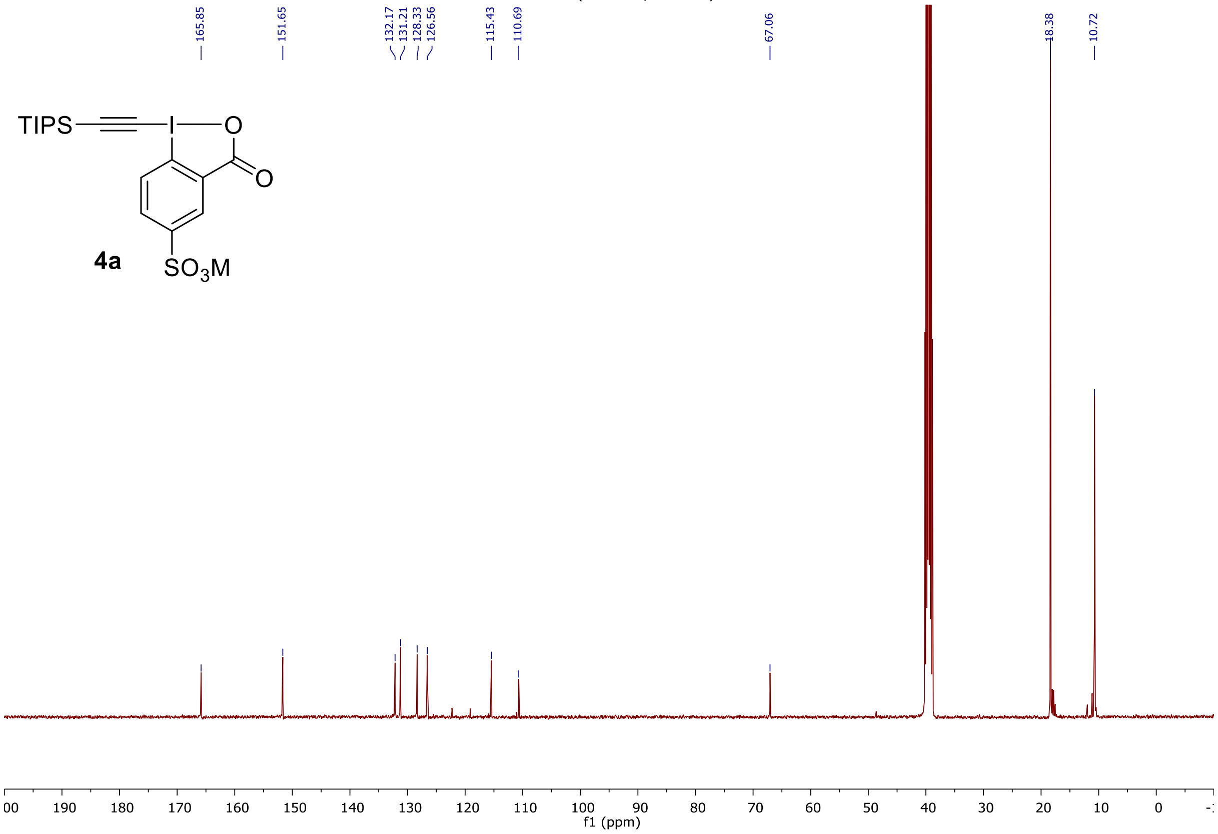

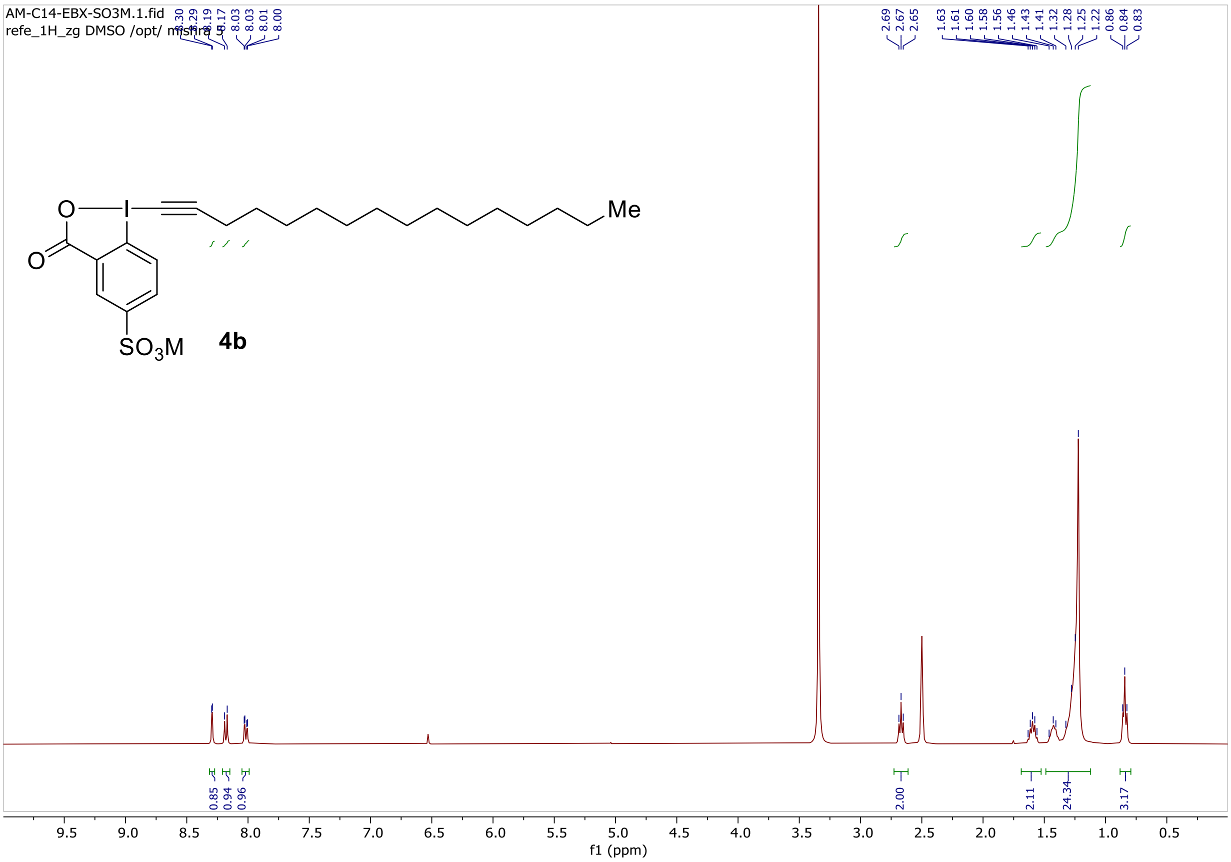
AM-C14-EBX-SO3M.1.fid
refe_1H_zg DMSO /opt/ mishra 5
4b
SO3M
Me
f1 (ppm)

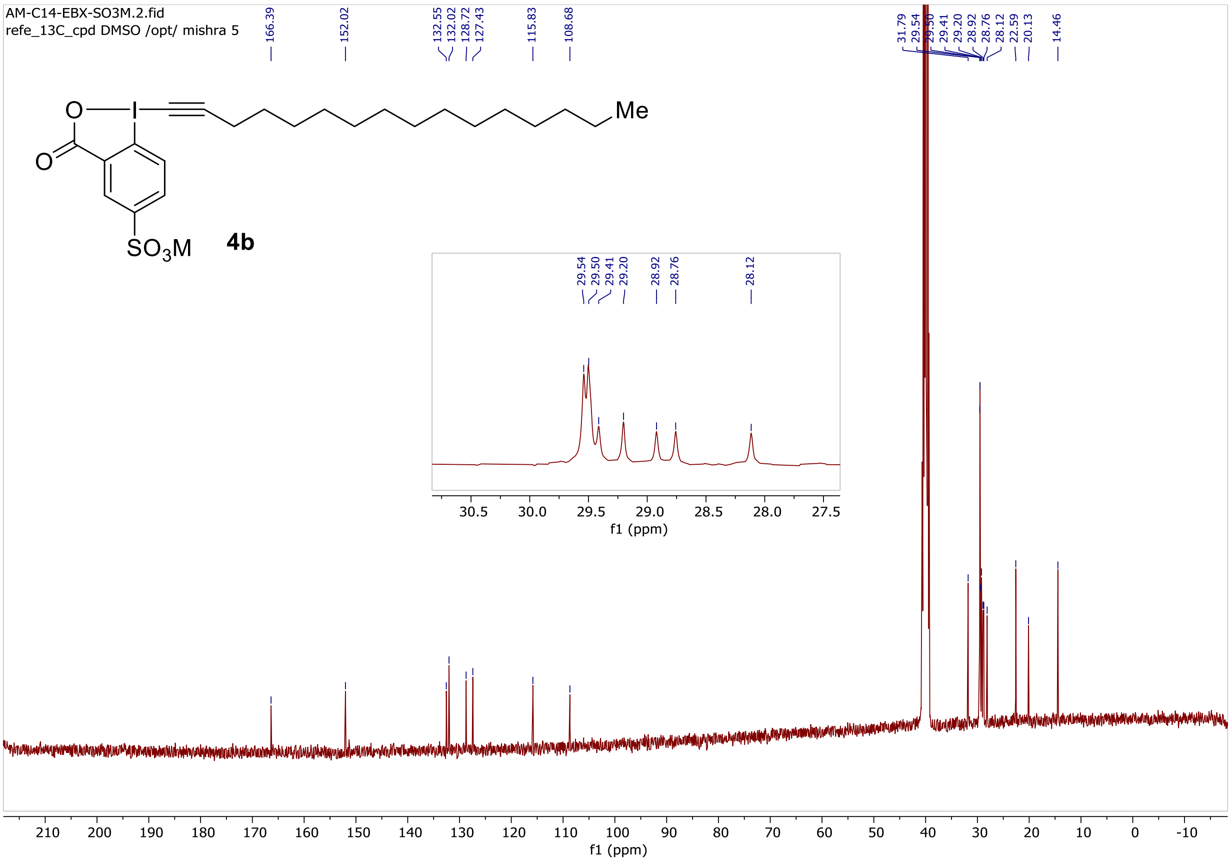
AM-C14-EBX-SO3M.2.fid
refe_13C_cpd DMSO /opt/ mishra 5
166.39
152.02
132.55
132.02
128.72
127.43
115.83
108.68
31.79
29.54
29.50
29.41
29.20
28.92
28.76
28.12
22.59
20.13
14.46
4b
SO3M
Me
f1 (ppm)

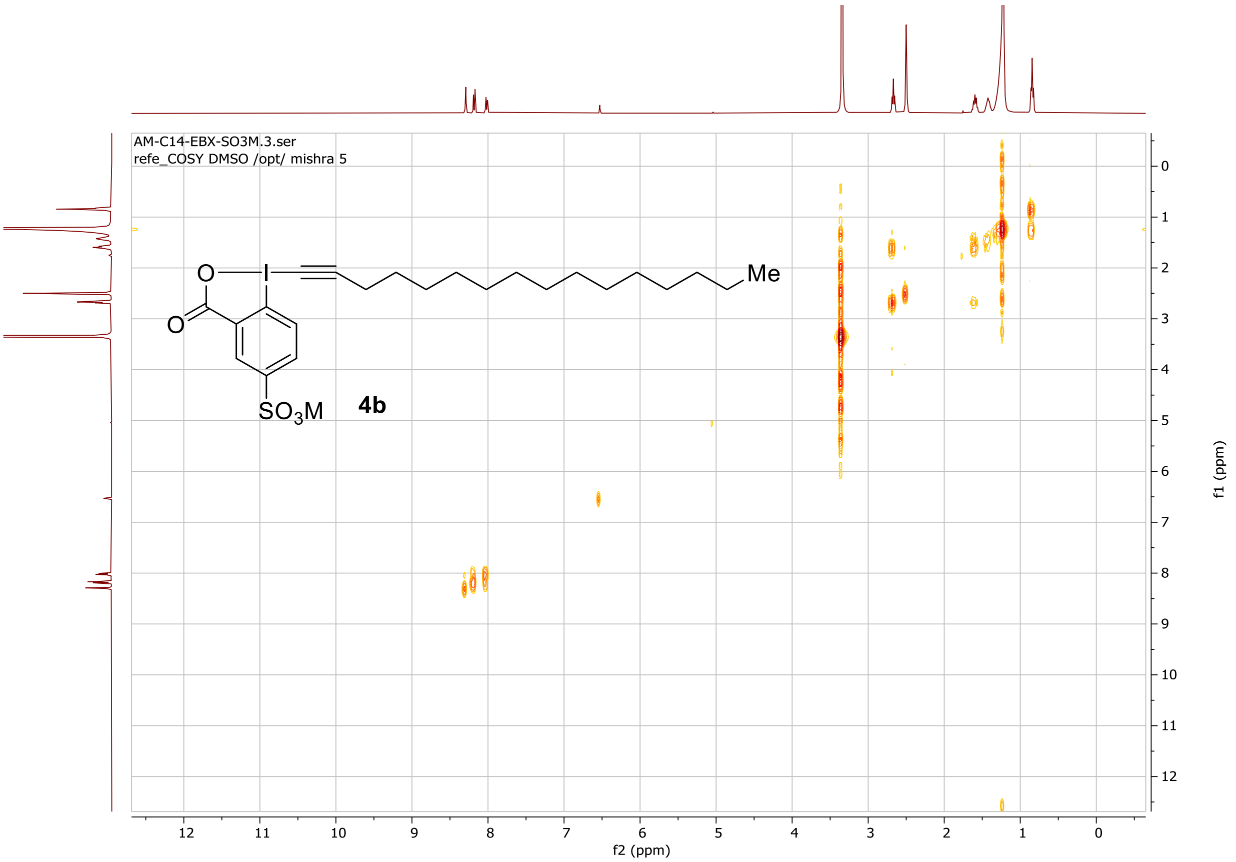
AM-C14-EBX-SO3M.3.ser
refe_COSY DMSO /opt/ mishra 5
O
I
Me
O
SO3M
4b
f2 (ppm)
f1 (ppm)

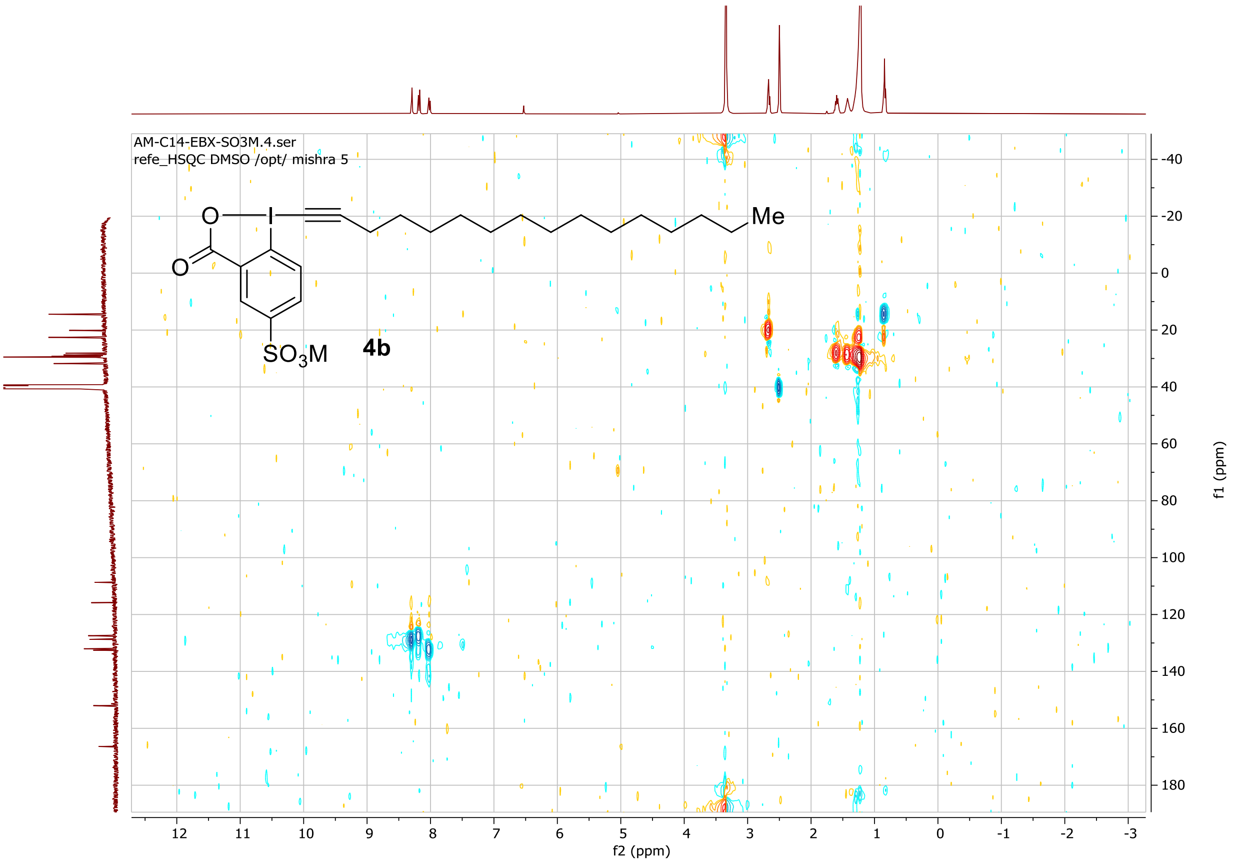
AM-C14-EBX-SO3M.4.ser
refe_HSQC DMSO /opt/ mishra 5
O
I
Me
O
SO3M
4b
f2 (ppm)
f1 (ppm)

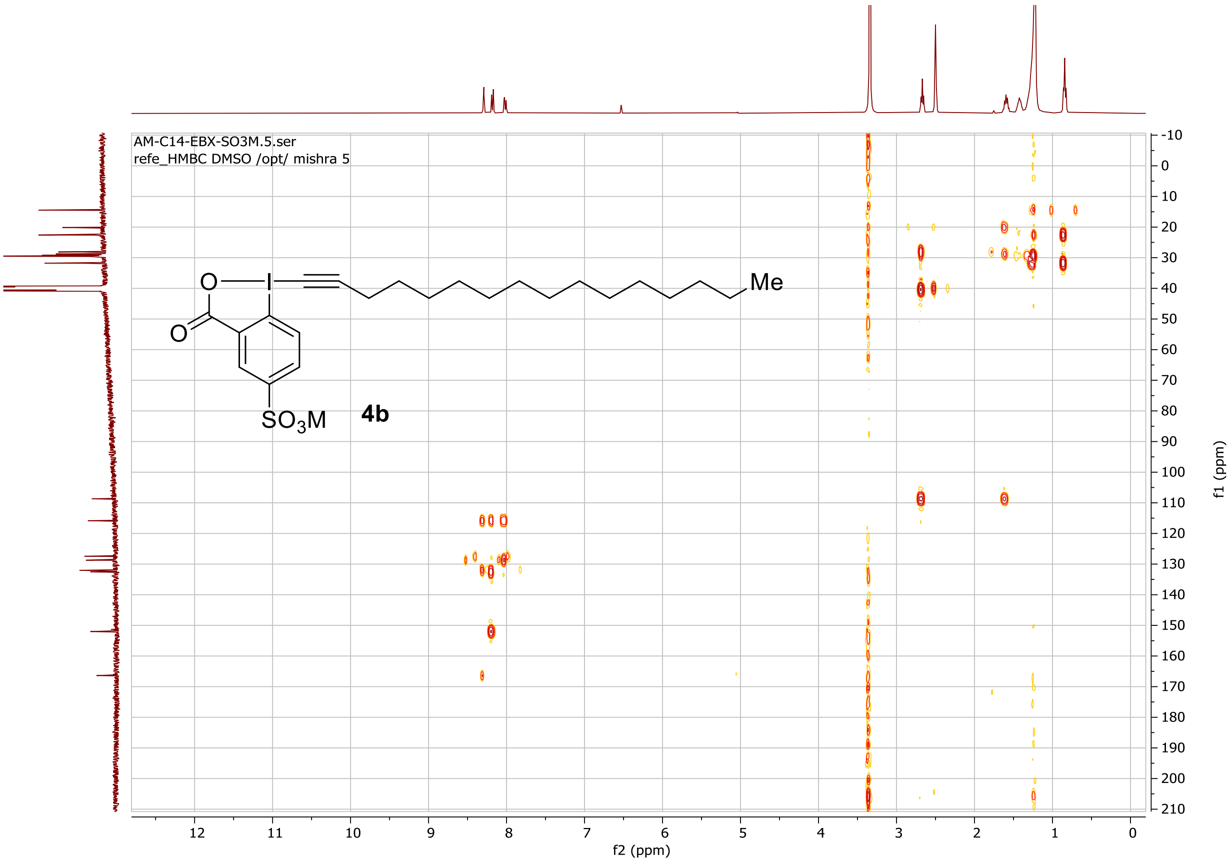
AM-C14-EBX-SO3M.5.ser
refe_HMBC DMSO /opt/ mishra 5
4b
f2 (ppm)
f1 (ppm)

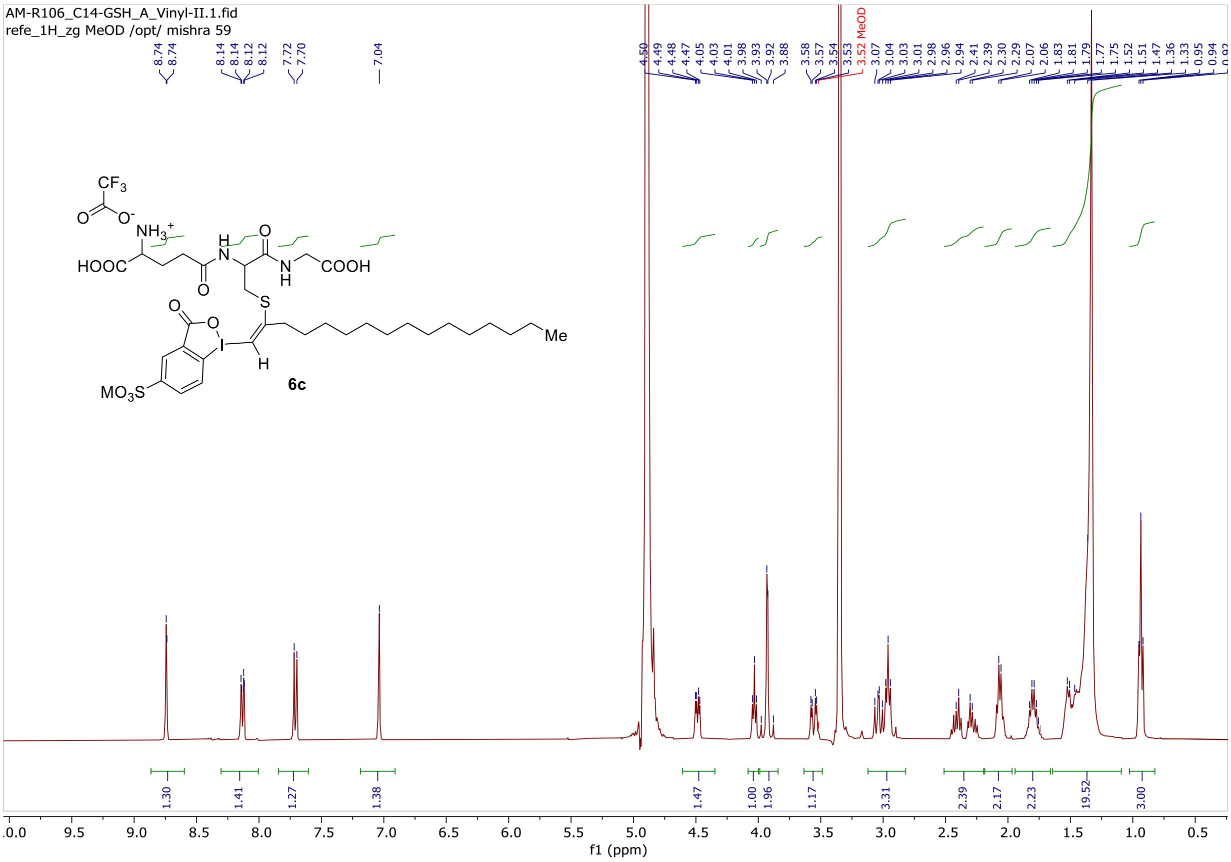
AM-R106_C14-GSH_A_Vinyl-II.1.fid
refe_1H_zg MeOD /opt/ mishra 59
6c
3.52 MeOD
f1 (ppm)

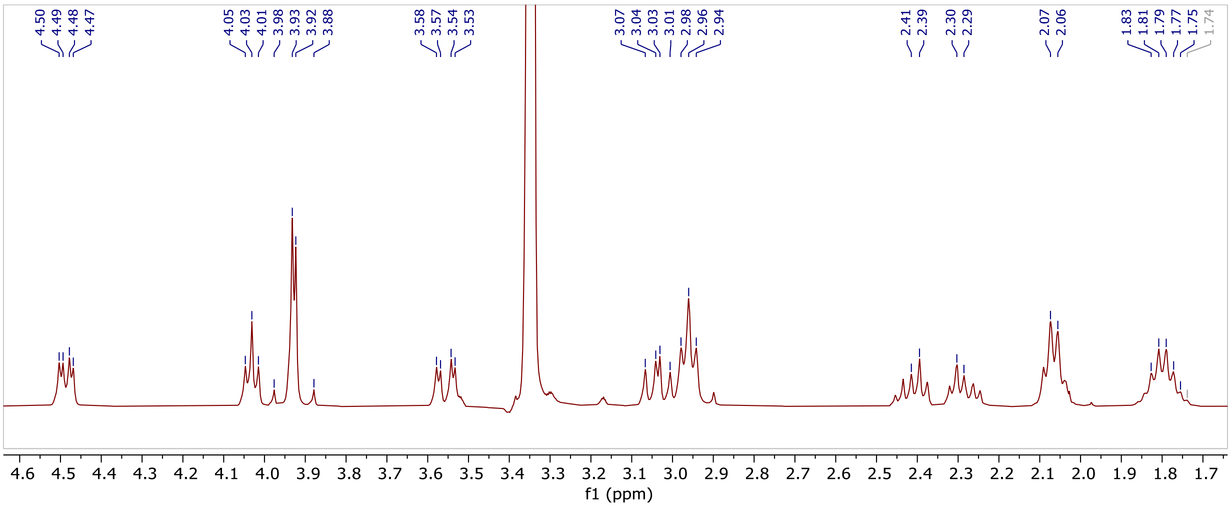
4.50
4.49
4.48
4.47
4.05
4.03
4.01
3.98
3.93
3.92
3.88
3.58
3.57
3.54
3.53
3.07
3.04
3.03
3.01
2.98
2.96
2.94
2.41
2.39
2.30
2.29
2.07
2.06
1.83
1.81
1.79
1.77
1.75
1.74
4.6 4.5 4.4 4.3 4.2 4.1 4.0 3.9 3.8 3.7 3.6 3.5 3.4 3.3 3.2 3.1 3.0 2.9 2.8 2.7 2.6 2.5 2.4 2.3 2.2 2.1 2.0 1.9 1.8 1.7
f1 (ppm)

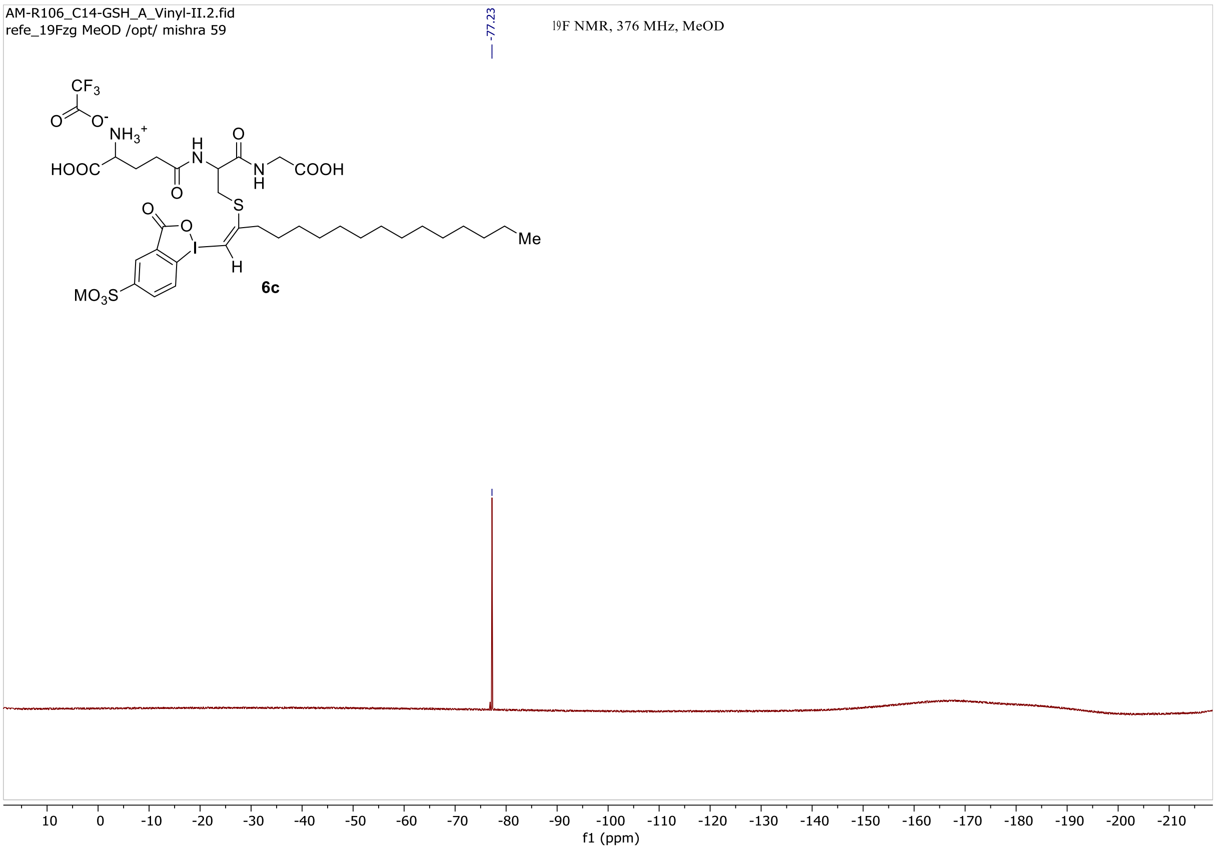
AM-R106_C14-GSH_A_Vinyl-II.2.fid
refe_19Fzg MeOD /opt/ mishra 59
-77.23
19F NMR, 376 MHz, MeOD
CF3
NH3+
HOOC
COOH
S
Me
MO3S
H
6c
10 0 -10 -20 -30 -40 -50 -60 -70 -80 -90 -100 -110 -120 -130 -140 -150 -160 -170 -180 -190 -200 -210
f1 (ppm)

SUPPORTING INFORMATION

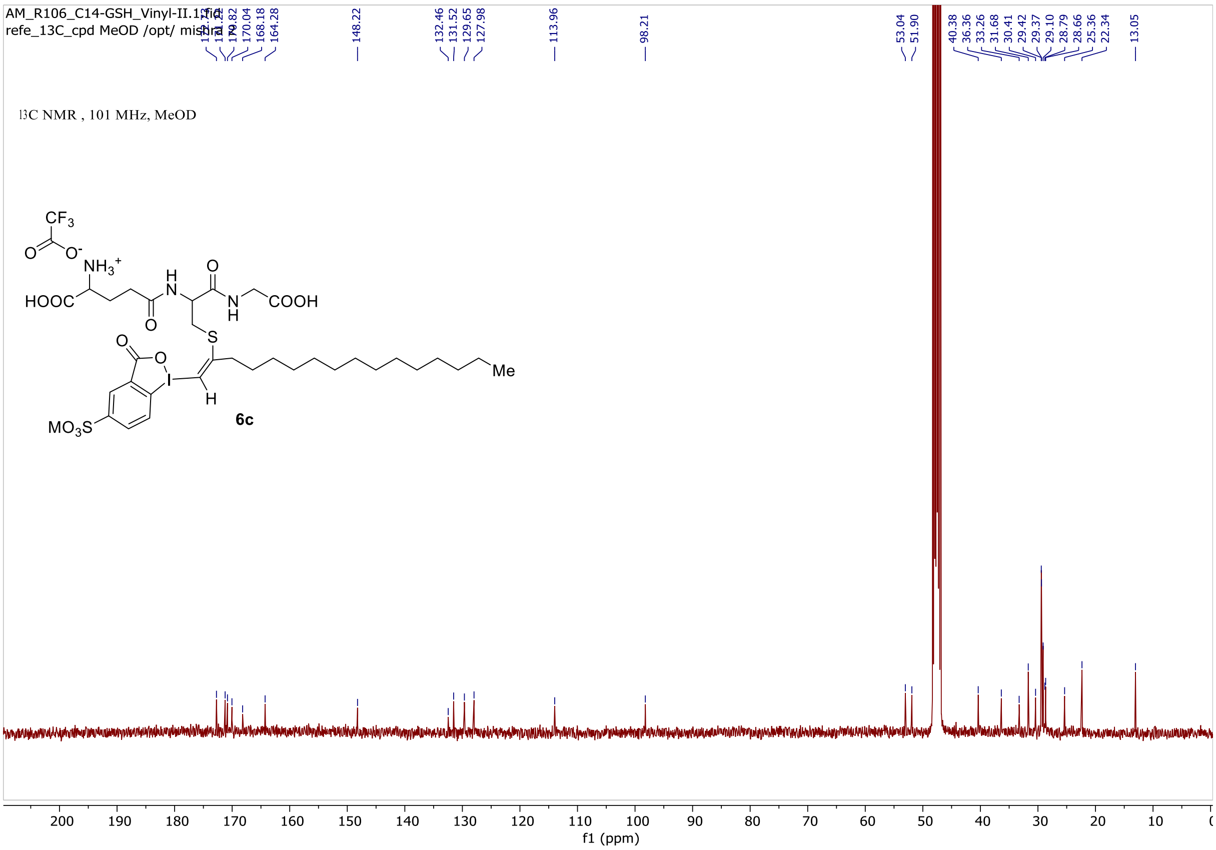

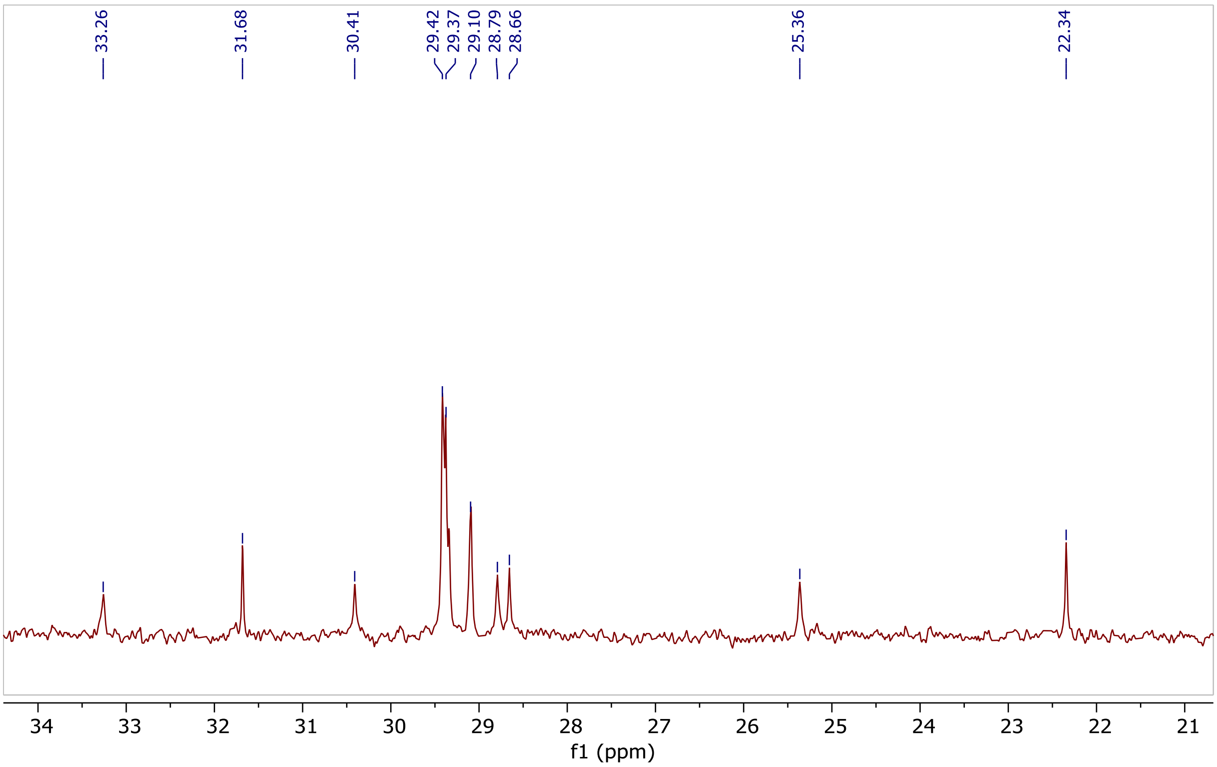

SUPPORTING INFORMATION

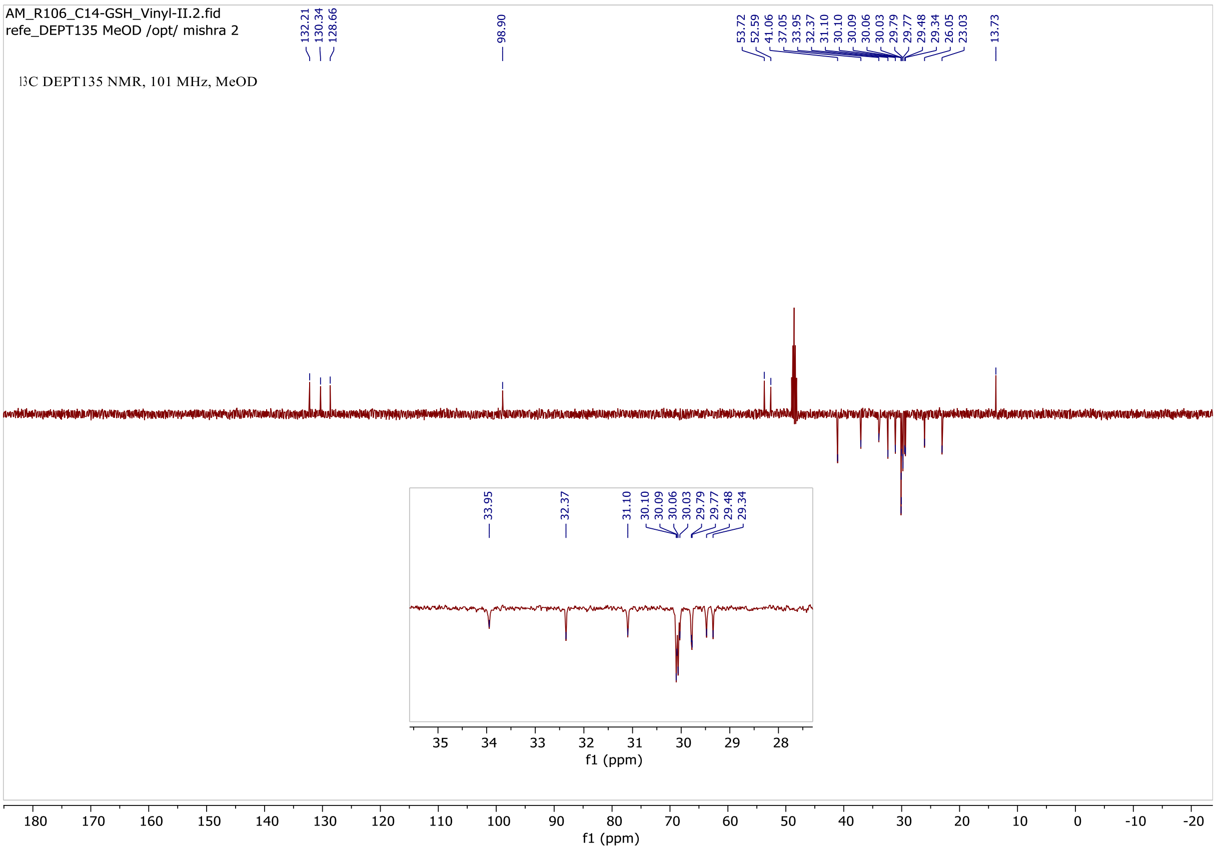

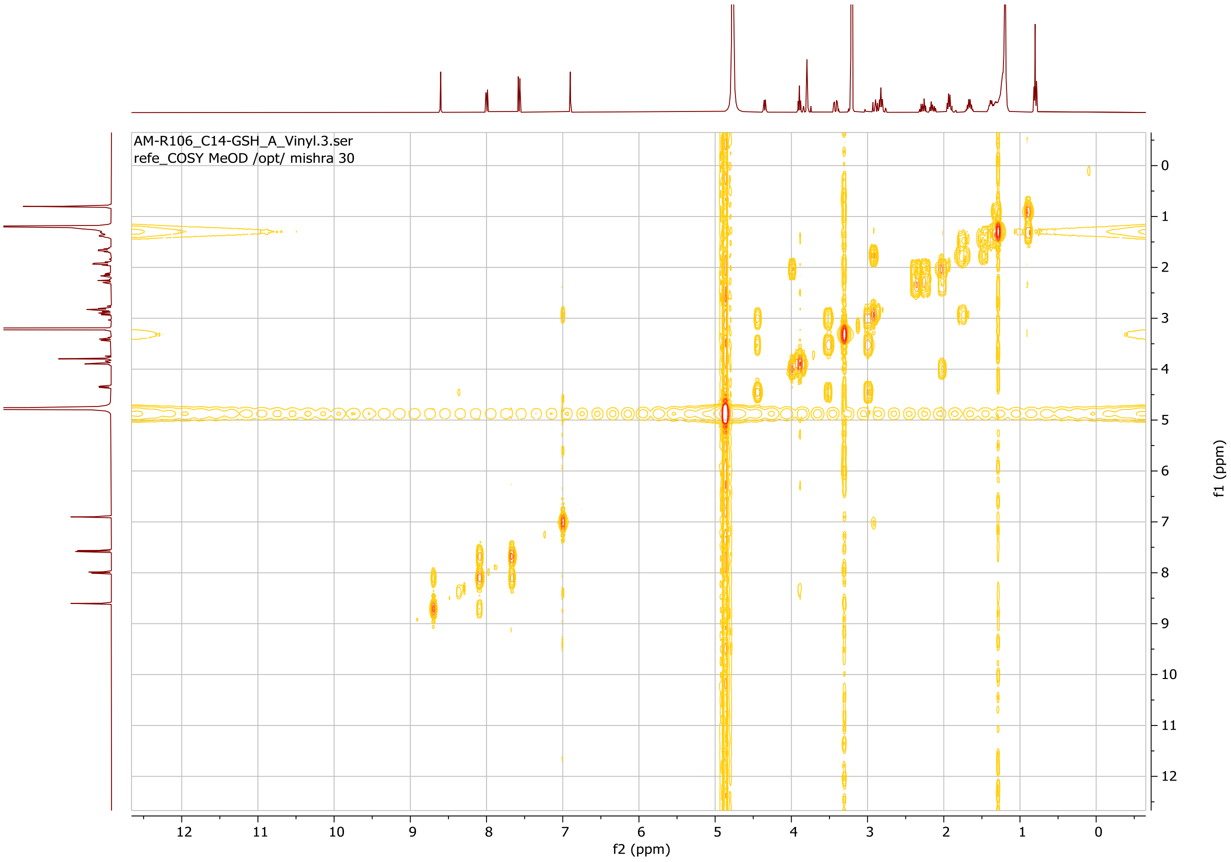

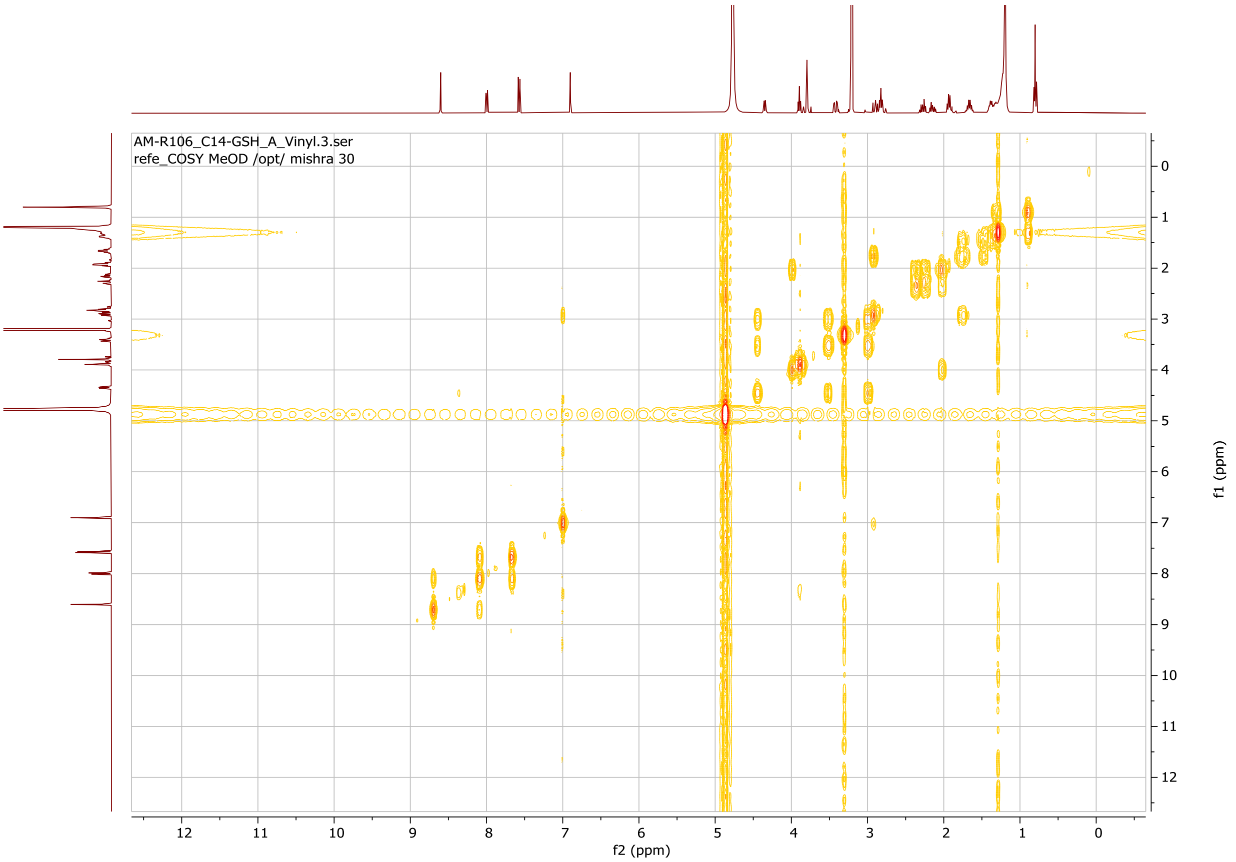
AM-R106_C14-GSH_A_Vinyl.3.ser
refe_COSY MeOD /opt/ mishra 30
f2 (ppm)
f1 (ppm)

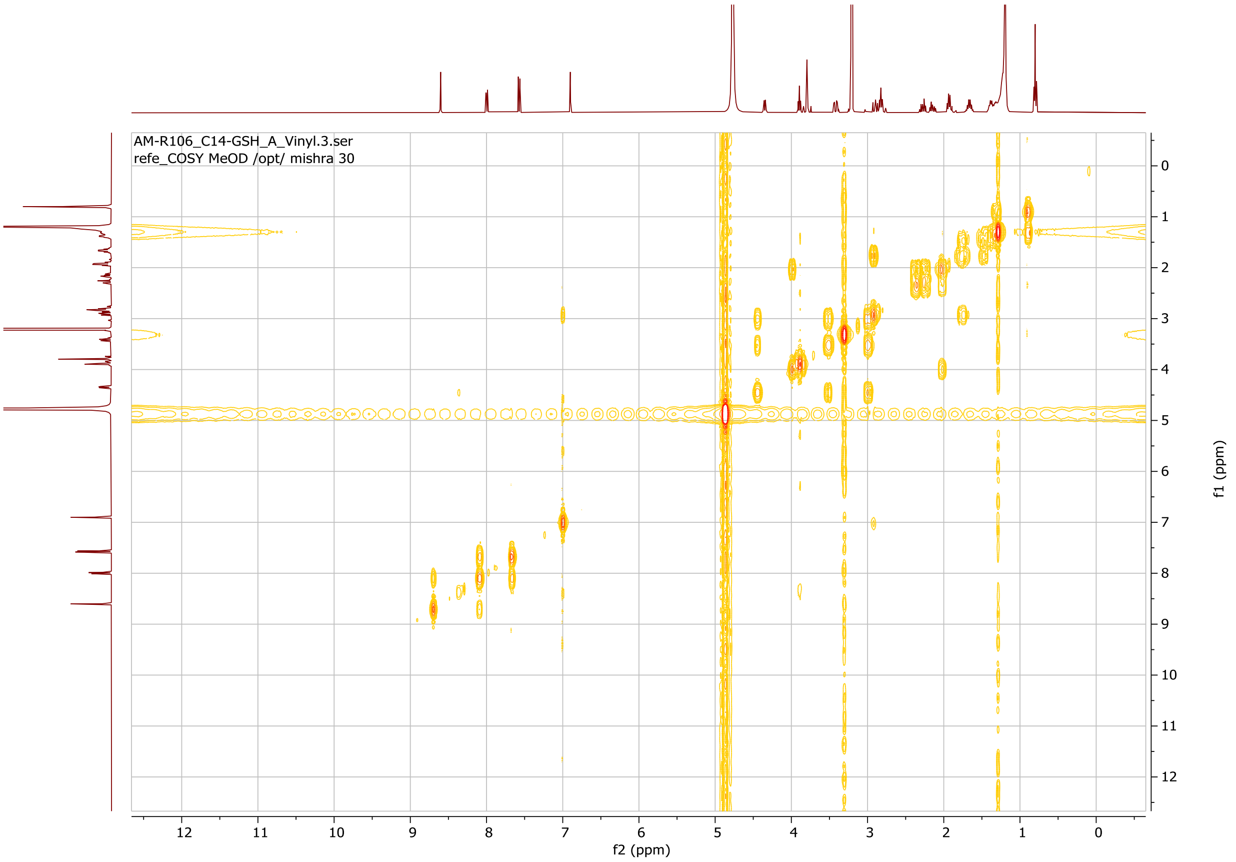
AM-R106_C14-GSH_A_Vinyl.3.ser
refe_COSY MeOD /opt/ mishra 30
f2 (ppm)
f1 (ppm)

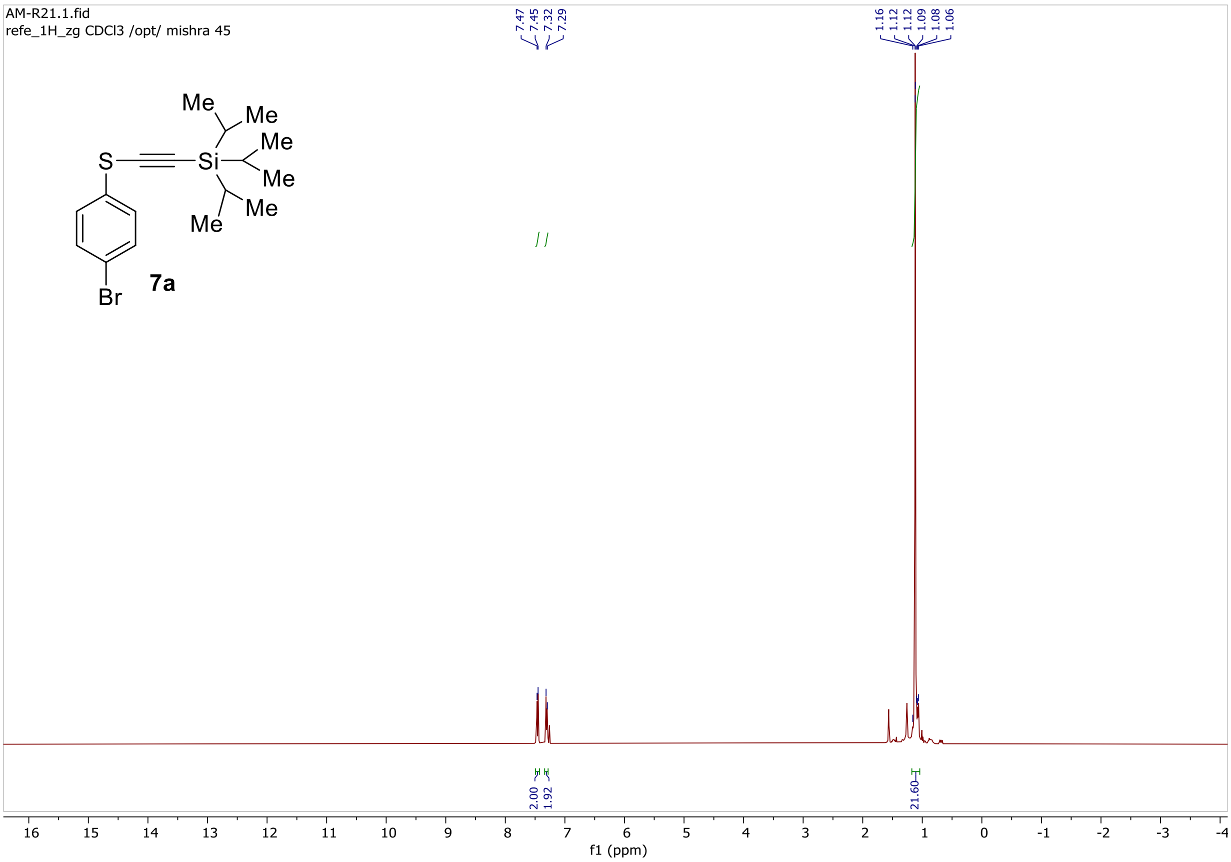
AM-R21.1.fid
refe_1H_zg CDCl3 /opt/ mishra 45
7.47
7.45
7.32
7.29
1.16
1.12
1.12
1.09
1.08
1.06
7a
2.00
1.92
21.60
f1 (ppm)

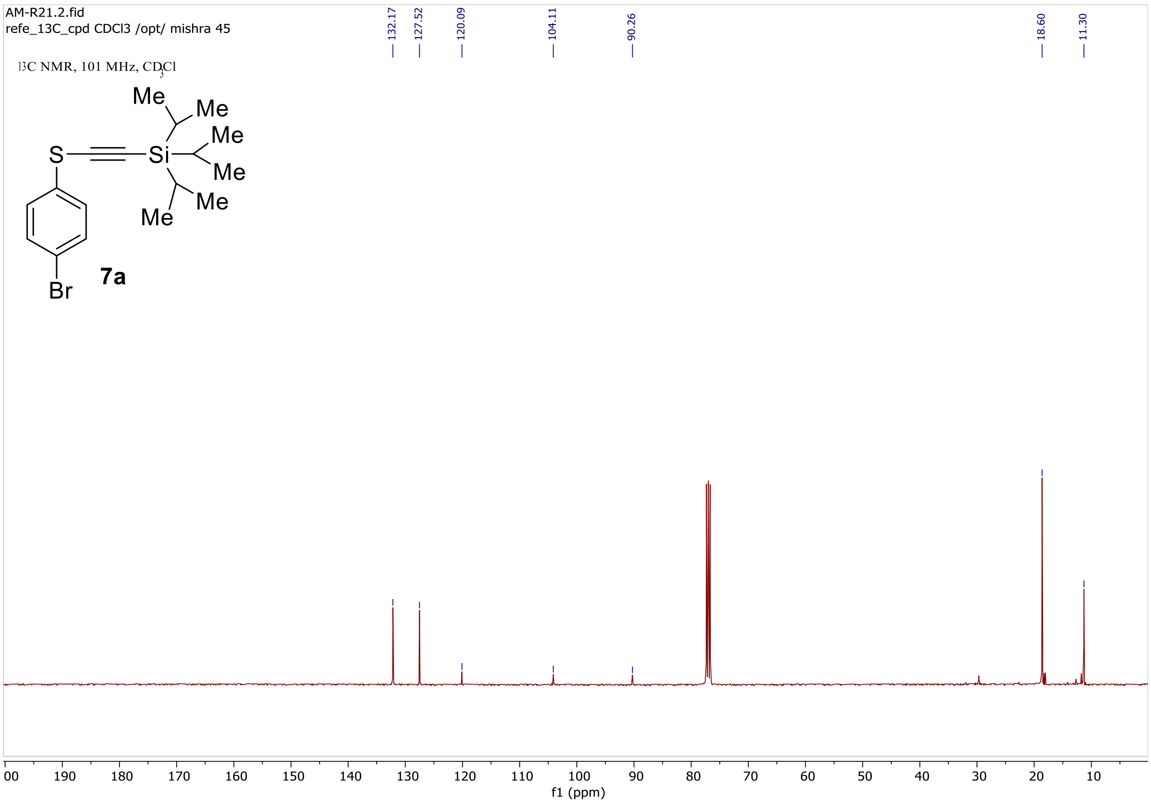
AM-R21.2.fid
refe_13C_cpd CDCl3 /opt/ mishra 45
132.17
127.52
120.09
104.11
90.26
18.60
11.30
13C NMR, 101 MHz, CDCl3
7a
f1 (ppm)

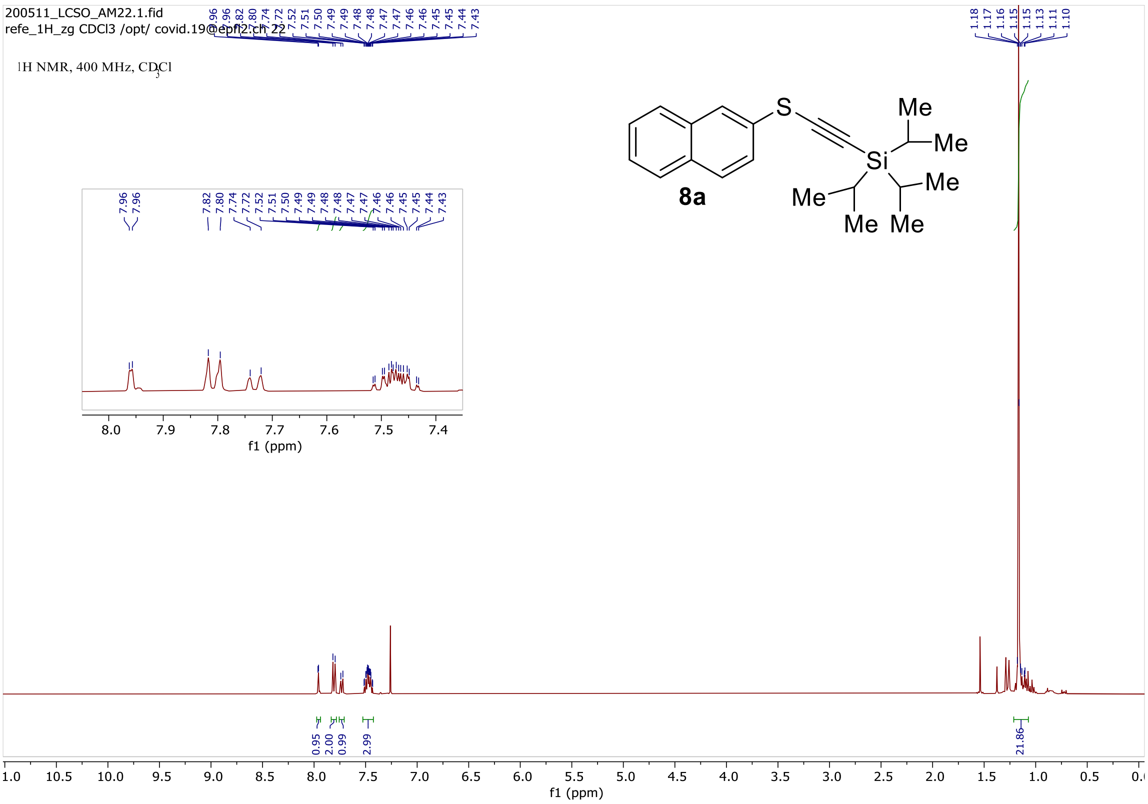
200511_LCSO_AM22.1.fid
refe_1H_zg CDCl3 /opt/ covid.19@epfl2.ch 22
1H NMR, 400 MHz, CDCl3
8a
S
Si
Me
f1 (ppm)
0.95
2.00
0.99
2.99
21.86

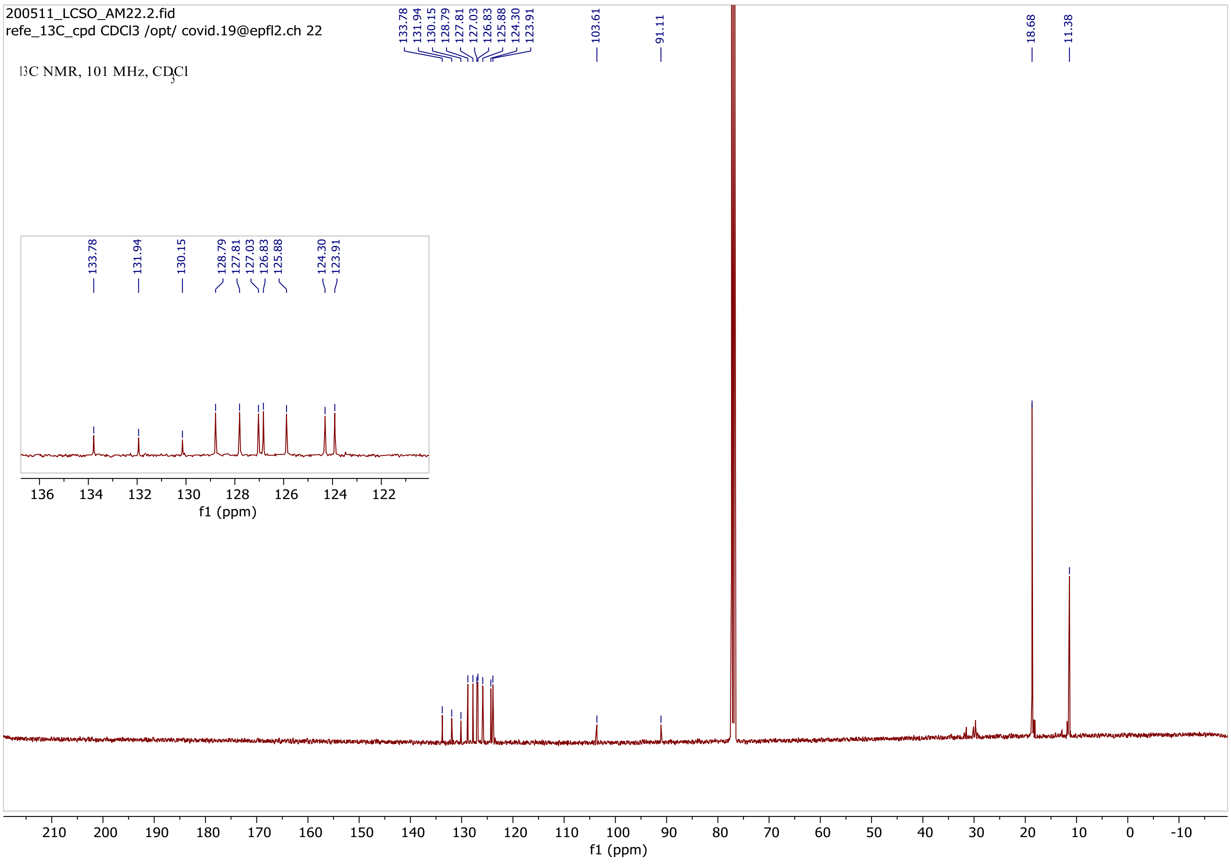
200511_LCSO_AM22.2.fid
refe_13C_cpd CDCl3 /opt/ covid.19@epfl2.ch 22
13C NMR, 101 MHz, CDCl3
133.78
131.94
130.15
128.79
127.81
127.03
126.83
125.88
124.30
123.91
103.61
91.11
18.68
11.38
f1 (ppm)

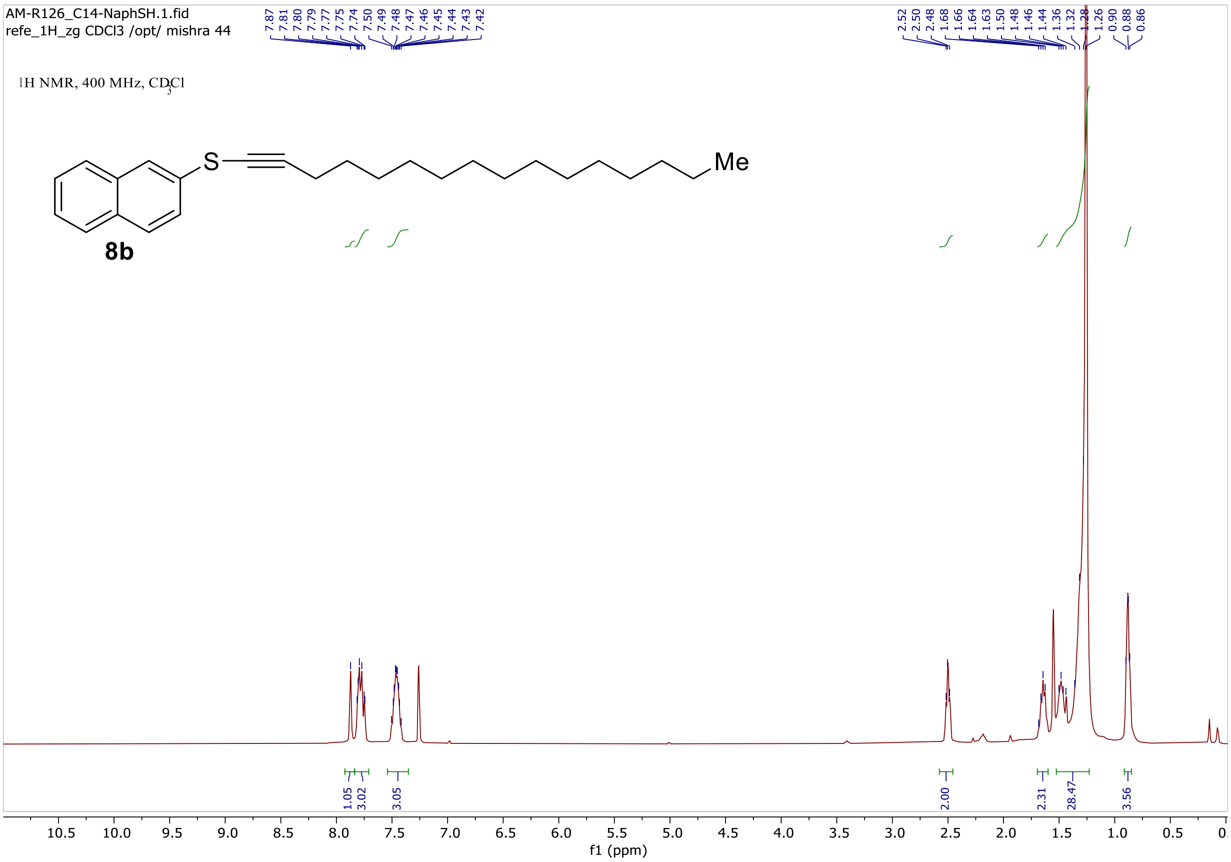
AM-R126_C14-NaphSH.1.fid
refe_1H_zg CDCl3 /opt/ mishra 44
1H NMR, 400 MHz, CDCl3
S
Me
8b
7.87
7.81
7.80
7.79
7.77
7.75
7.74
7.50
7.49
7.48
7.47
7.46
7.45
7.44
7.43
7.42
2.52
2.50
2.48
1.68
1.66
1.64
1.63
1.50
1.48
1.46
1.44
1.36
1.32
1.28
1.26
0.90
0.88
0.86
1.05
3.02
3.05
2.00
2.31
28.47
3.56
f1 (ppm)

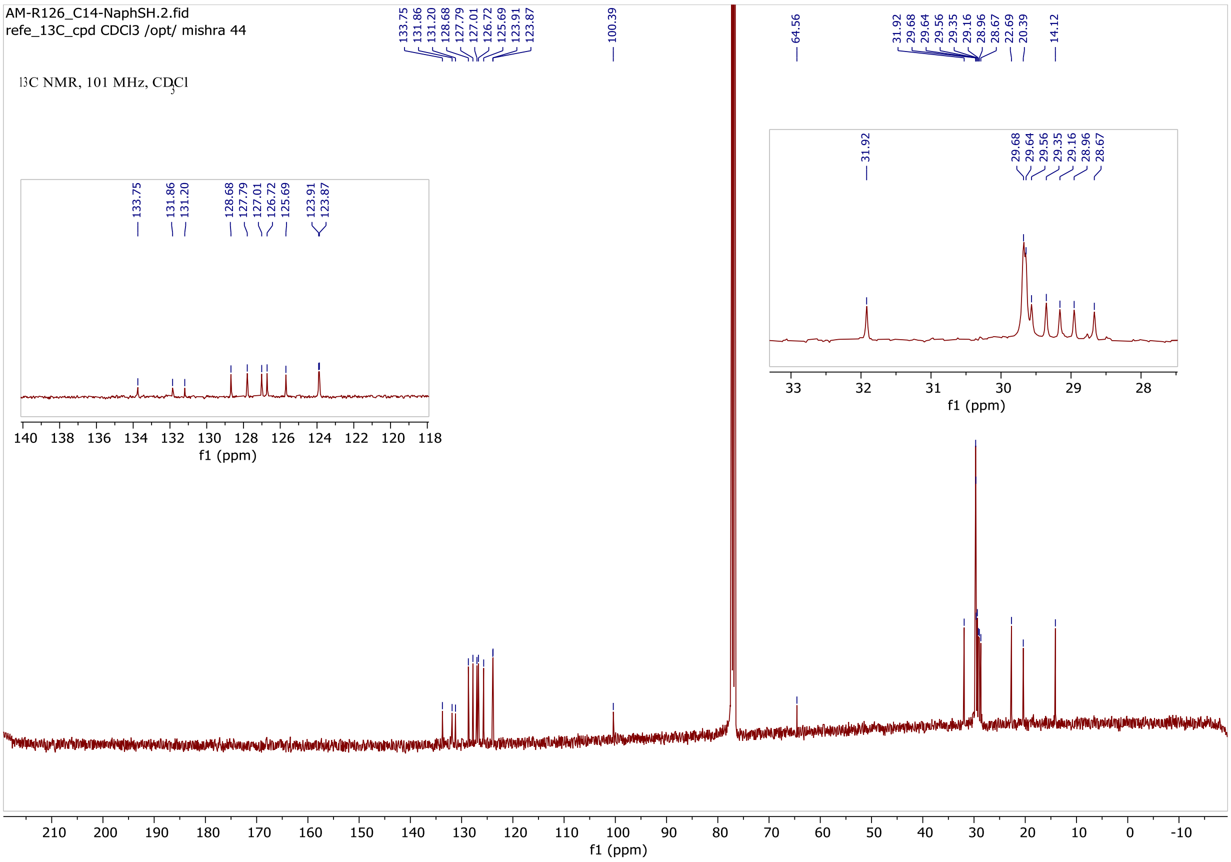
AM-R126_C14-NaphSH.2.fid
refe_13C_cpd CDCl3 /opt/ mishra 44
13C NMR, 101 MHz, CDCl3
133.75
131.86
131.20
128.68
127.79
127.01
126.72
125.69
123.91
123.87
100.39
64.56
31.92
29.68
29.64
29.56
29.35
29.16
28.96
28.67
22.69
20.39
14.12
f1 (ppm)

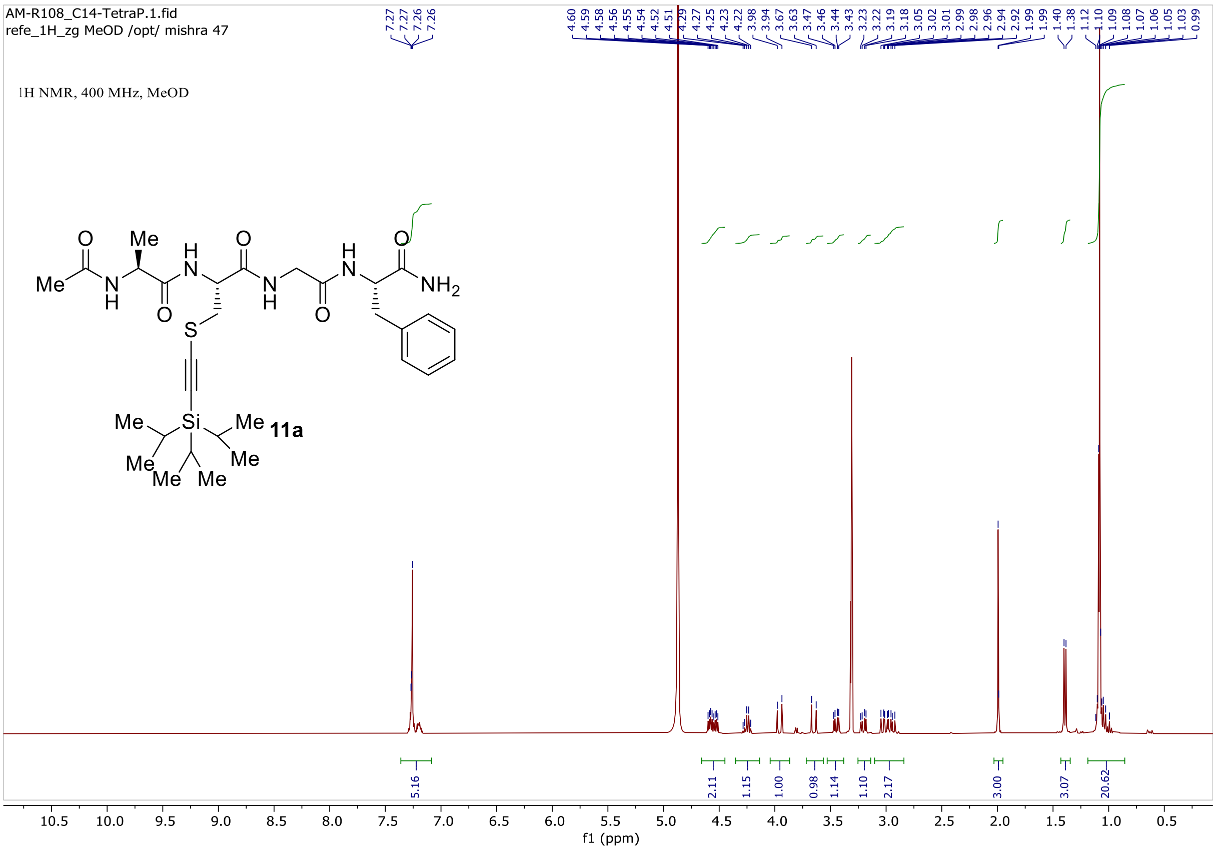
AM-R108_C14-TetraP.1.fid
refe_1H_zg MeOD /opt/ mishra 47
1H NMR, 400 MHz, MeOD
11a
f1 (ppm)

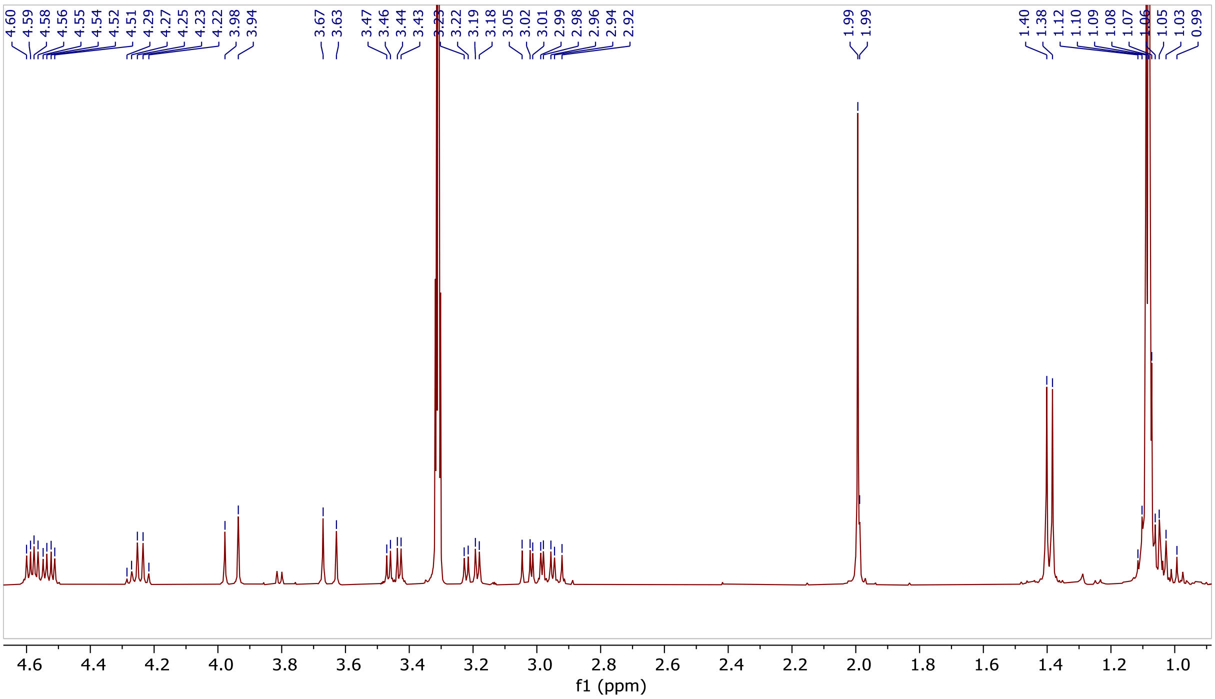
f1 (ppm)

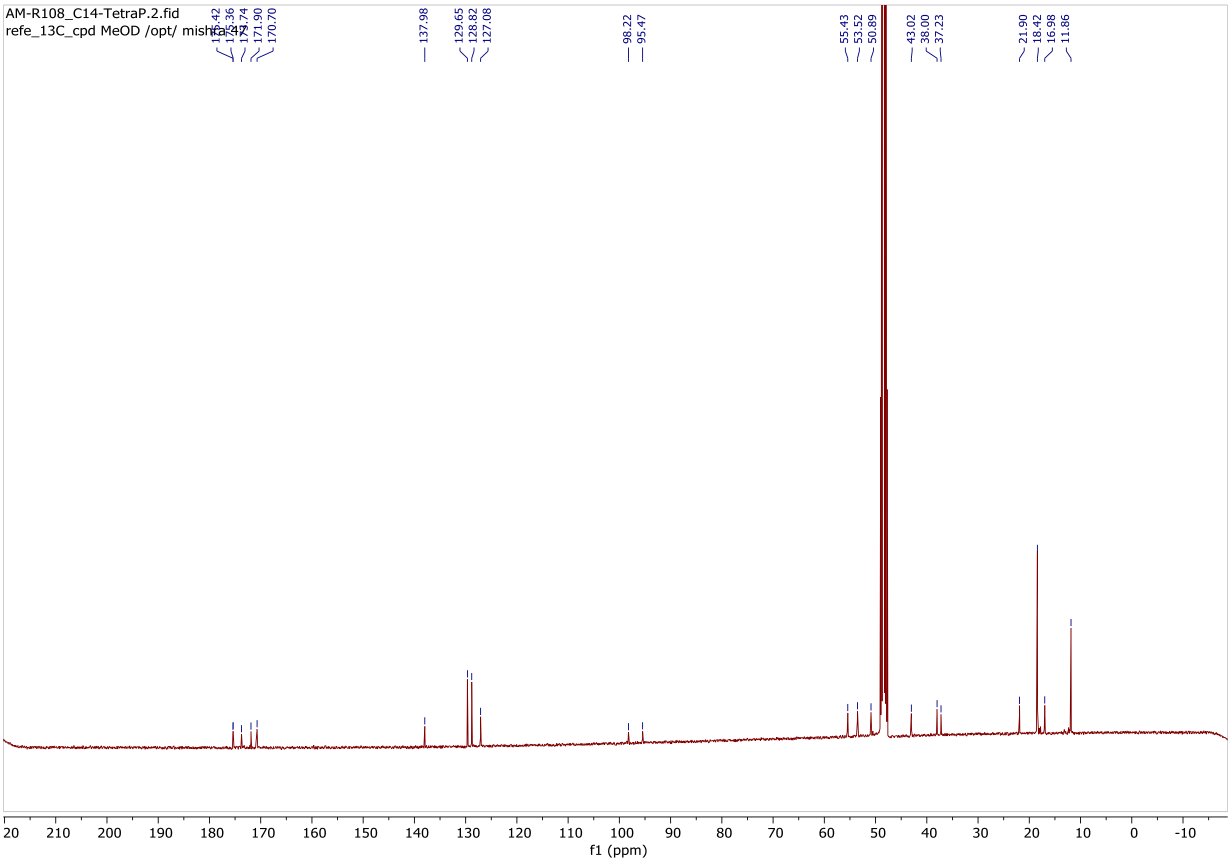
AM-R108_C14-TetraP.2.fid
refe_13C_cpd MeOD /opt/ mishra 47
171.90
170.70
137.98
129.65
128.82
127.08
98.22
95.47
55.43
53.52
50.89
43.02
38.00
37.23
21.90
18.42
16.98
11.86
20
210
200
190
180
170
160
150
140
130
120
110
100
90
80
70
60
50
40
30
20
10
0
-10
f1 (ppm)

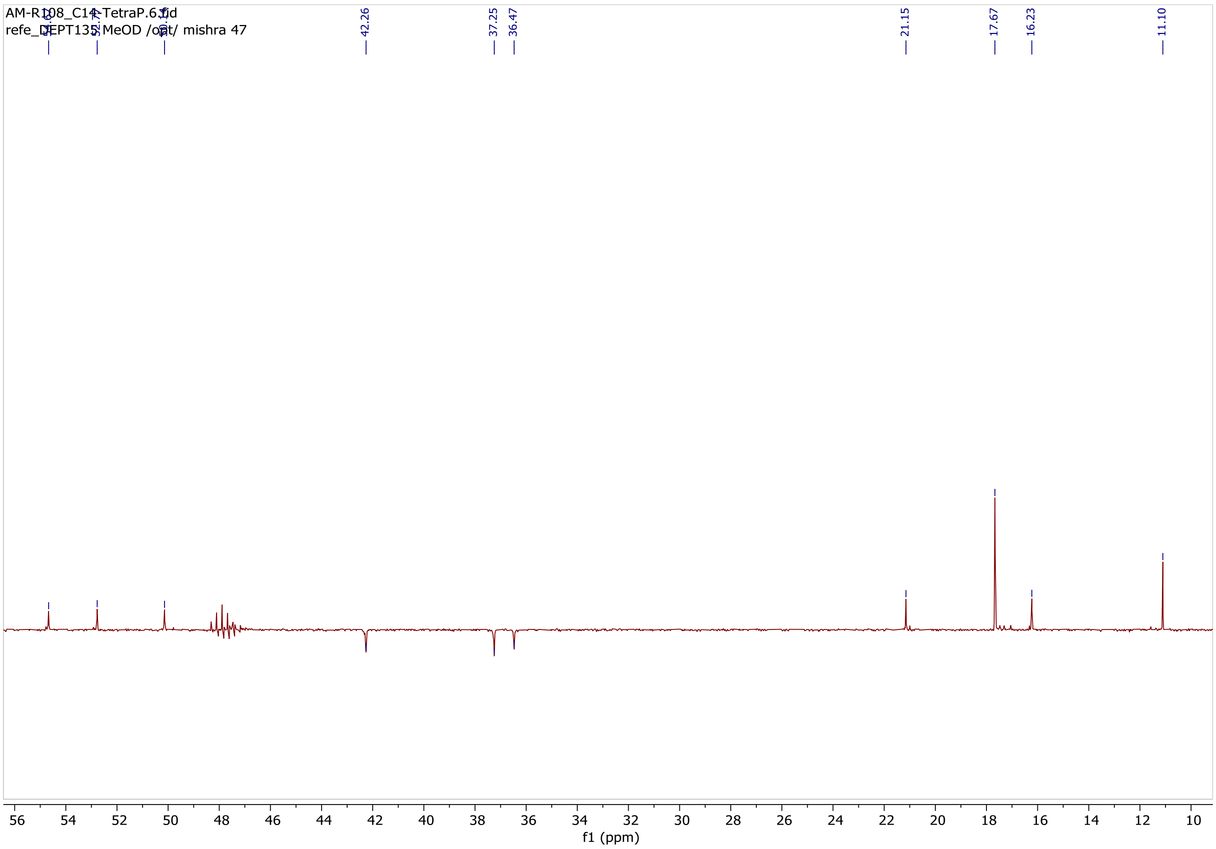
AM-R108_C14-TetraP.6.fid
refe_DEPT135 MeOD /opt/ mishra 47
42.26
37.25
36.47
21.15
17.67
16.23
11.10
56
54
52
50
48
46
44
42
40
38
36
34
32
30
28
26
24
22
20
18
16
14
12
10
f1 (ppm)

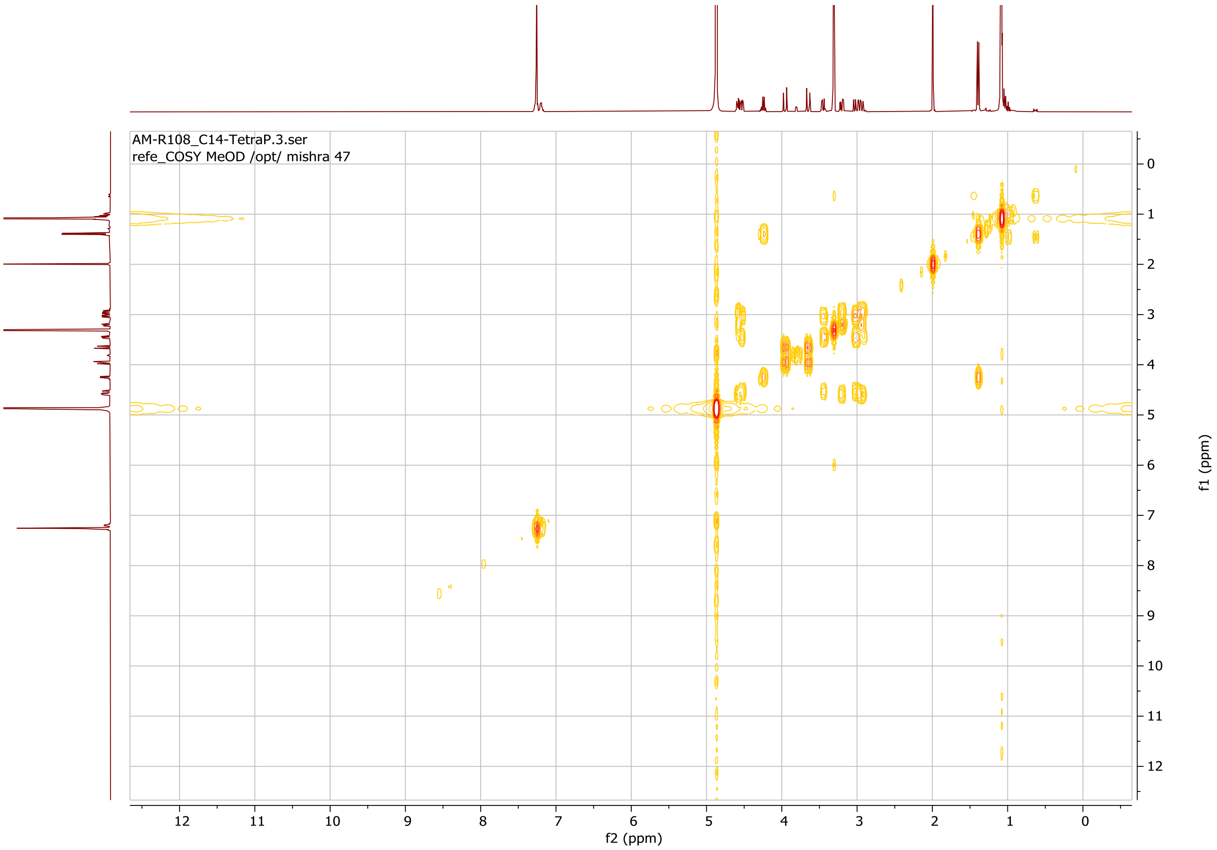
AM-R108_C14-TetraP.3.ser
refe_COSY MeOD /opt/ mishra 47
f2 (ppm)
f1 (ppm)

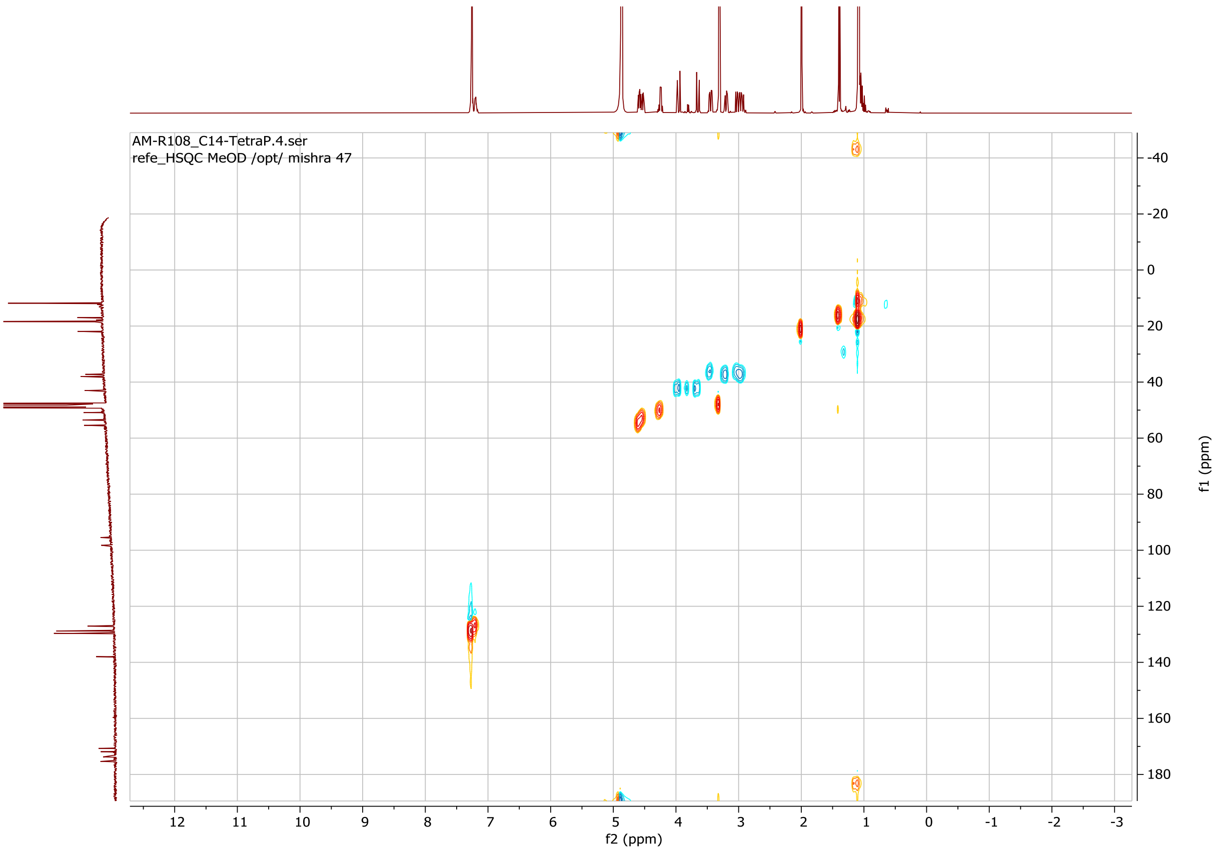
AM-R108_C14-TetraP.4.ser
refe_HSQC MeOD /opt/ mishra 47
f2 (ppm)
f1 (ppm)

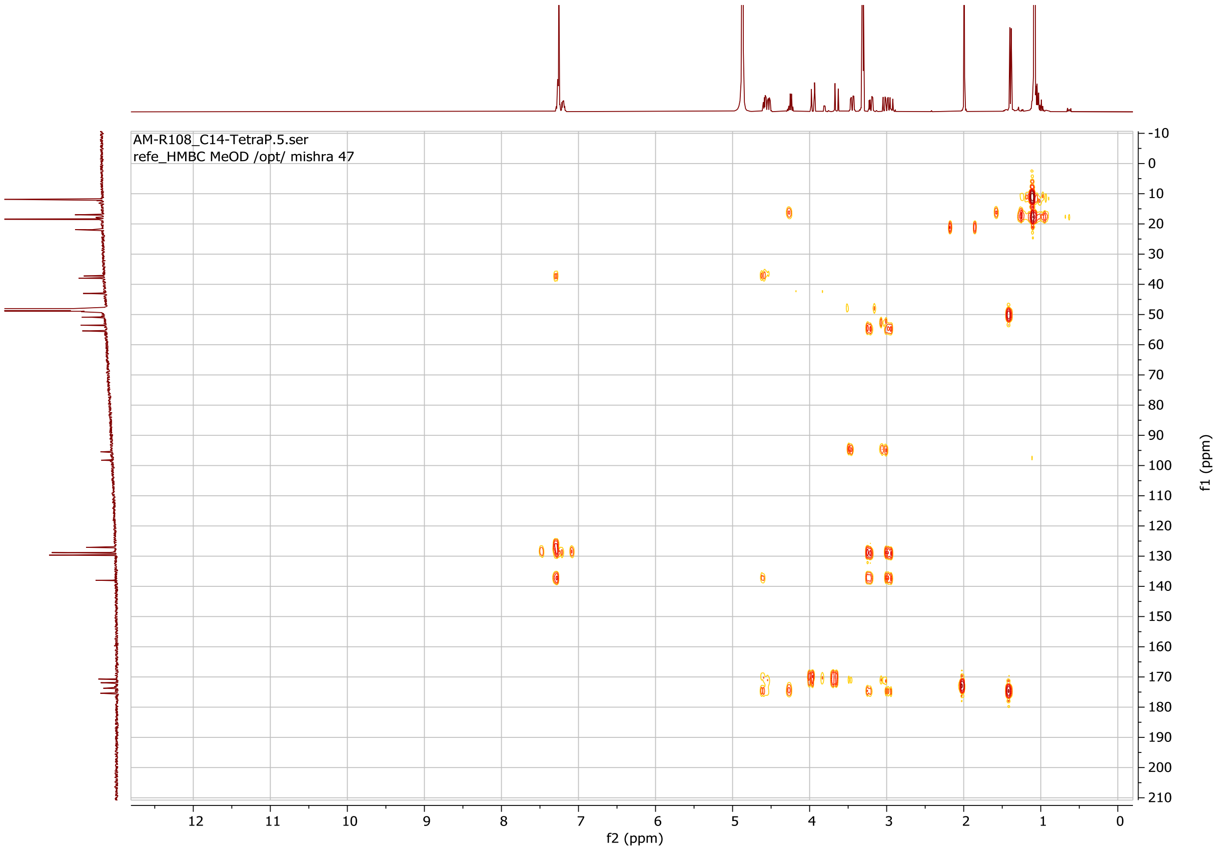
AM-R108_C14-TetraP.5.ser
refe_HMBC MeOD /opt/ mishra 47
f2 (ppm)
f1 (ppm)
